# Causal effects of gut microbiota on physical growth and cognitive performance via plasma metabolites: A Mendelian randomization study

**DOI:** 10.1097/MD.0000000000049865

**Published:** 2026-07-24

**Authors:** Fang-Fang Yang, Kai-Peng Luo, Zhi-Jie Liang, Zhuo-Xin Ou, Sheng-Lan Li, Jia-Mei Liang, Yu-Nan Lin

**Affiliations:** a The First Clinical College, Guangxi Medical University, Nanning, China; b Department of Anesthesiology, The First Affiliated Hospital of Guangxi Medical University, Nanning, China.

**Keywords:** cognitive performance, gut microbiota, Mendelian randomization, physical growth, plasma metabolites

## Abstract

Herein, we used plasma metabolites as potential mediators to determine the causal relationship between gut microbiota, cognitive performance, and physical growth. The relationship between gut microbiota and these 3 factors has not been accurately established in human experiments and studies; our study may provide some new insights. Two-sample Mendelian randomization was performed to evaluate causal associations between gut microbial composition and 3 core phenotypes (height, body weight, cognitive performance). Publicly available GWAS-identified significant SNPs were applied as instrumental variables, and mediation analyses were further conducted to quantify the proportional metabolite-mediated effects. Causal analyses confirmed 3 microbial taxa associated with height: Enterococcus B showed a positive correlation, while CAG-485 and Rhodococcus exhibited negative effects. Six microbial signatures were significantly linked to body weight, with four taxa displaying protective associations and 2 showing adverse impacts. Rhodanobacter and UBP9 were positively associated with cognitive performance. Mediation analyses verified that distinct microbial taxa regulated the 3 phenotypes through specific metabolites, with varied mediating percentages for growth and cognitive outcomes. The study findings highlight the metabolite pathways of the gut microbiota as potential targets for precise nutritional interventions, growth optimization, and cognitive health enhancement.

## 1. Introduction

Cognitive function is garnering increasing attention, as indicated by studies on dietary supplements to enhance cognitive impairment in Parkinson’s disease, and the effect of short-form videos on cognitive processes. Collectively, these efforts reflect the unwavering pursuit of researchers to enhance cognitive performance.^[1,2]^ Simultaneously, obesity (body mass index [BMI] > 30) has emerged as a contemporary global health concern that affects individuals of all ages and geographical locations.^[3]^ Height, body weight, and cognitive performance are fundamental markers for physical growth and neurodevelopment; this underscores their importance in assessing the physiological well-being of humans. Therefore, in this study, we suggest the identification of precision nutrition strategies based on the “gut microbiota–metabolite–phenotype” causal chain to optimize physical growth and cognitive performance, using height, body weight, and cognitive performance as key phenotypic markers. This goal is consistent with the preventive healthcare principle that prevention is better than treatment.

As the host’s “second genome,” the gut microbiota significantly affects immune regulation, metabolic homeostasis, and neural function by dynamically modulating physiological processes such as immune responses, energy metabolism, and neurotransmitter synthesis.^[4-7]^ Furthermore, specific microbial taxa and their metabolites, including bile acids, amino acid derivatives, and neurotransmitters, facilitate transkingdom signaling between the host and microbiota, serving as biological bridges that can link microbial ecosystems to complex phenotypes via plasma metabolite-mediated systemic effects.^[8]^ The gut microbiota modulates neurotransmitter production, transport, and function, affects Parkinson and Alzheimer diseases, and potentially affects obesity and cognition via estrogen-related pathways. Murine studies have further revealed links among gut microbiota, cognition, and obesity. Moreover, clinical trials have indicated that probiotics can enhance cognitive flexibility and decrease stress in healthy older adults.^[8-11]^ Emerging evidence suggests associations between gut microbiota abundance/diversity and pediatric development, including height deficits in children, and causal links to short stature.^[12,13]^ Fecal transplantation from undernourished Malawian donors into gnotobiotic mice recapitulates developmental defects such as skeletal deformities, growth impairment, and multitissue metabolic dysregulation.^[14]^ Current research on stunting and gut microbiota predominantly focuses on pediatric populations. Our study, based on adult data, addresses this gap by investigating the association between adult height and gut microbial composition.

Concurrently, lipid metabolites such as diacylglycerol and sphingolipids are abnormally accumulated in patients with obesity and metabolic syndrome with microbiota dysbiosis, suggesting that the microbiota disrupts energy homeostasis. Beyond metabolism, neurodevelopmental studies have revealed genus-level connections between autism spectrum disorder and *Ruminococcaceae UCG005* and *Prevotellaceae*, emphasizing the gut microbiota–brain pathway as a potential pathway linking microbial ecology to neuropsychiatric pathogenesis.^[15,16]^ Collectively, these results provide insight into the cross-systemic mechanisms behind the crosstalk between the microbiota and host by combining microbial ecology, plasma metabolomics, and host physiology across metabolic, skeletal, and neural domains.

In observational studies, Mendelian randomization (MR) helps effectively decrease confounding bias, providing strong causal evidence for the relationship between exposure and outcome. Notably, the combination of 2-sample MR and mediation models has helped clarify microbiota–metabolite–function pathways in metabolism and cognition.^[15,17-20]^ However, their role in regulating cognitive performance and physical growth in healthy populations remains unknown.

In the present study, we combined information from genome-wide association studies (GWAS) on human phenotypes (height, weight, and cognitive performance), gut microbiota, and plasma metabolites. Then, we addressed 3 primary questions using a 2-step MR approach: whether the gut microbiota exhibits genetic effects on cognitive and physical development; whether certain plasma metabolites mediate these relationships; and the biological mechanisms and potential for nutritional intervention for important genus–metabolite–phenotype pathways. Our study findings will advance translational applications of the diet–microbiota–phenotype axis in promoting overall health by providing a theoretical foundation for microbiota-targeted precision nutritional strategies.

## 2. Materials and methods

### 2.1. Study design

**Figure 1.**
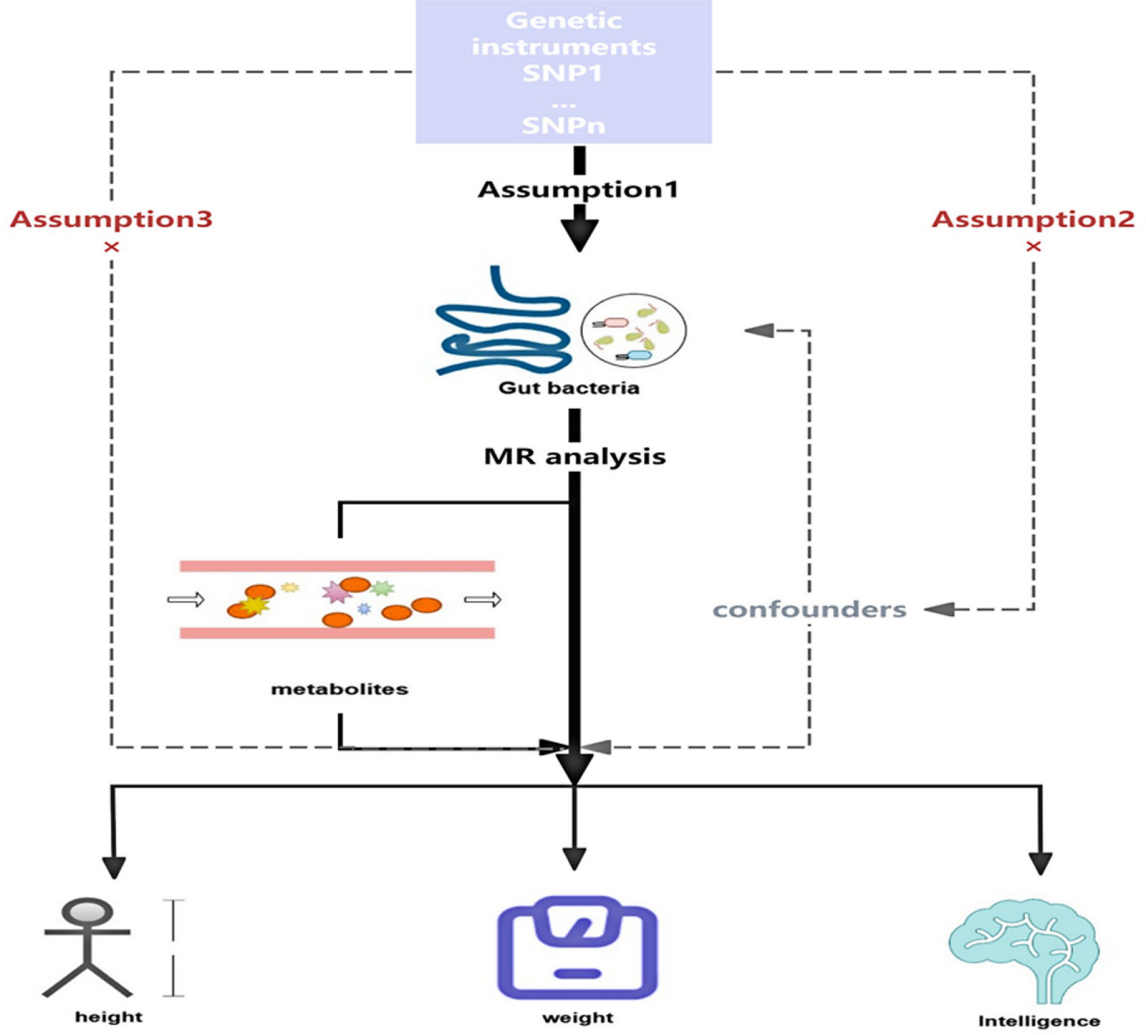
Study flowchart. Image adapted from: https://bioicons.com/. MR = Mendelian randomization, SNP = single-nucleotide polymorphism.

Figure 1 illustrates the study workflow. First, instrumental variables for univariate MR analysis were collected from specific single-nucleotide polymorphisms (SNPs) to determine causal associations among gut microbiota, plasma metabolites, and target phenotypes (height, body weight, and cognitive performance). Thereafter, a 2-step MR strategy was applied to identify candidate plasma metabolites that potentially mediate the causal effects of gut microbiota on host phenotypes.

### 2.2. Data sources

GWAS analyses were conducted in 5959 participants from the FR02 cohort. Associations between 7967,866 human genetic variants and 2801 microbial taxa were assessed.^[21]^ Metabolomic profiles were derived from 8299 participants of European descent in the Canadian Longitudinal Study on Aging, which included data on 1091 plasma metabolites and 309 metabolite-to-metabolite ratios.^[22]^ Outcome data were derived from the following sources: body weight: UKB-b-11842 (n = 4,61,632; SNPs = 98,51,867); cognitive performance: ebi-a-GCST006250 (n = 2,69,867; SNPs = 92,76,181; Supplementary Materials: Intelligence 1, Intelligence 2, Supplemental Digital Content 1); and height: UK Biobank analysis of 459,327 European samples with 23 highly heritable phenotypes and ~20 million imputed variants.^[23]^

### 2.3. Selection of instrument variables

To improve the reliability of causal inference in MR analyses, SNPs serving as instrumental variables for gut microbiota and plasma metabolite traits were only included if they exceeded a suggestive genome-wide significance threshold of *P* < 1 × 10^−5^.^[24]^ When very few genome-wide significant loci are identified in the original GWAS, SNPs with a relaxed threshold of *P* < 1 × 10^−5^ were included for subsequent analysis.^[17,24]^ To preserve independent variants (*R*^2^ < 0.001 within a 10 Mb window), linkage disequilibrium-based clumping was utilized. *F*-statistics were calculated to confirm the instrument’s strength. Only SNPs with values higher than the standard cutoff of 10 were retained for subsequent analyses.

## 3. Statistical analysis

The first MR analyses evaluated possible causal connections among gut microbiota, plasma metabolomes, and targeted phenotypes (height, body weight, and cognitive performance). Inverse variance weighting (IVW) was used as the primary analytical approach. To verify the robustness of the results, complementary methods such as weighted median, MR-Egger, simple mode, and weighted mode estimators were applied. Pleiotropy was measured using the MR-Egger intercept and PRESSO method, whereas heterogeneity was evaluated using Cochran test (Tables S2, S3 and S4, Supplemental Digital Content 2). Significance was determined when the IVW outcomes were *P* < .05, with no indications of heterogeneity or pleiotropy (*P* > .05), utilizing α = 0.05 as the cutoff. We performed false discovery rate (FDR) correction on all the results to mitigate the impact of multiple testing errors.

To determine metabolite-mediated pathways, a 2-step MR framework was applied to assess how plasma metabolites affect the causal effects of the gut microbiota on height, body weight, and cognitive performance. Odds ratios (ORs) and 95% confidence intervals (CIs) were used to characterize the effects associated with a 1 standard deviation (SD) change in exposure. The proportions for mediation were determined using the equation: (β_1_ × β_2_)/β, where β indicates the overall causal effect derived from the main analysis, and β_1_ and β_2_ reflect the effects of gut microbiota on mediators and mediators on associated phenotypes, respectively. The delta method was used to calculate CIs and standard errors (Table S1, Supplemental Digital Content 5).

## 4. Results

### 4.1. Effect of gut microbiota on height, body weight, and cognitive performance

**Figure 2.**
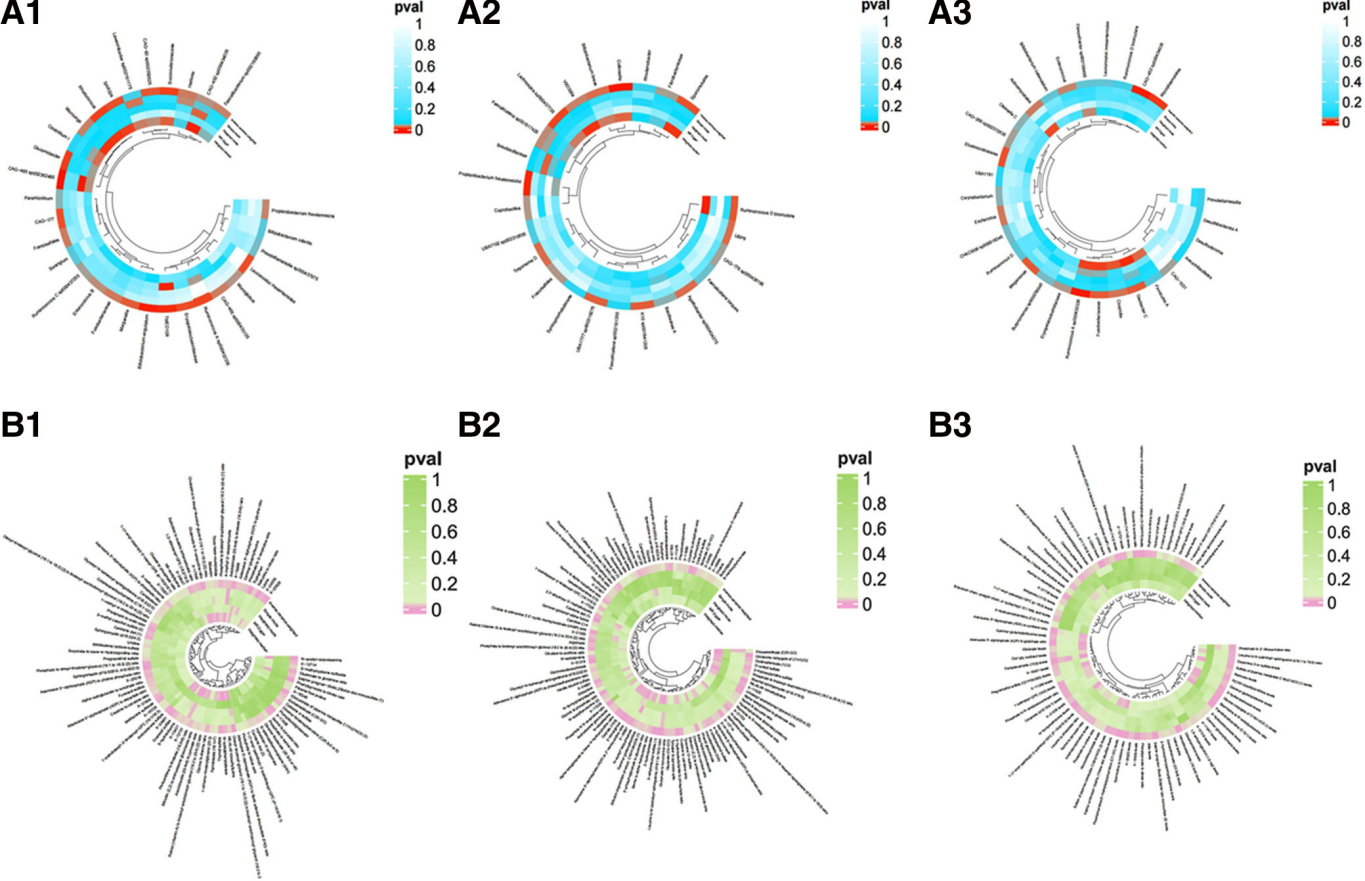
Causal effects of the gut microbiota and plasma metabolites on height, body weight, and cognitive performance. (A1) Gut microbiota–height, (A2) Gut microbiota–body weight, and (A3) Gut microbiota–cognitive performance. (B1) Plasma metabolites–height, (B2) Plasma metabolites–weight, and (B3) Plasma metabolites–cognitive performance.

Figure 2 (panels A1–A3 and B1–B3) illustrates the possible causal associations between gut microbiota and plasma metabolites for height, body weight, and cognitive performance. A 2-sample MR analysis revealed that many gut microbiota characteristics exhibited indicative associations (PIVW < 0.05) with these phenotypes. For height (Fig. 2A1), 16 taxa exhibited positive correlations, including *Enterococcus B* (OR: 1.037, 95% CI: 1.003–1.072, *P *= .028), whereas 13 taxa exhibited negative correlations, including *CAG-485 sp002362485* (OR: 0.972, 95% CI: 0.960–0.985, *P *= .000) and *Rhodococcus* (OR: 0.926, 95% CI: 0.875–0.980, *P *= .008). For body weight (Fig. 2A2), 21 taxa exhibited positive associations, including *Fusobacteriaceae* (OR: 1.049, 95% CI: 1.009–1.090, *P *= .015) and *Actinomycetales* (OR: 1.040, 95% CI: 1.005–1.077, *P *= .024), whereas 8 taxa exhibited negative correlations, including *CAG-1031* (OR: 0.981, 95% CI: 0.965–0.998, *P *= .034), *Flavonifractor sp900199495* (OR: 0.970, 95% CI: 0.942–0.999, *P *= .044), *CAG-269 sp002372935* (OR: 0.981, 95% CI: 0.965–0.998, *P *= .033), and *UBA1191* (OR: 0.967, 95% CI: 0.935–0.999, *P *= .048). For cognitive performance (Fig. 2A3), 11 taxa exhibited positive correlations, including *Rhodanobacter* (OR: 1.113, 95% CI: 1.001–1.237, *P *= .047) and *UBP9* (OR: 1.080, 95% CI: 1.012–1.152, *P *= .019), whereas 13 taxa exhibited negative correlations, including *Agathobacter sp000434275* (OR: 0.967, 95% CI: 0.941–0.995, *P *= .019) and *Coprobacillus* (OR: 0.967, 95% CI: 0.938–0.997, *P *= .031). Figure S1, Supplemental Digital Content 6 illustrates detailed scatter, forest, leave-one-out, and funnel plots used in the 2-sample MR investigation of the effects of the gut microbiota on height, body weight, and cognitive performance.

### 4.2. Metabolite-mediated effects on height, body weight, and cognitive performance

Two-sample MR analysis revealed that a subset of plasma metabolites exhibited suggestive causal associations with height, body weight, and cognitive performance (*P*IVW < 0.05). For height (Fig. 2B1), 40 metabolites exhibited positive correlations, whereas 51 exhibited negative correlations. For body weight (Fig. 2B2), 63 metabolites exhibited positive correlations, whereas 44 exhibited negative associations. For cognitive performance (Fig. 2B3), 31 metabolites exhibited positive correlations, whereas 50 exhibited negative correlations. Figure S1, Supplemental Digital Content 6 illustrates the detailed scatter, forest, leave-one-out, and funnel plot sensitivity analyses for the 2-sample MR analysis of the effects of plasma metabolites on these phenotypes.

### 4.3. Plasma metabolites as mediators of potential causal relationships

**Figure 3.**
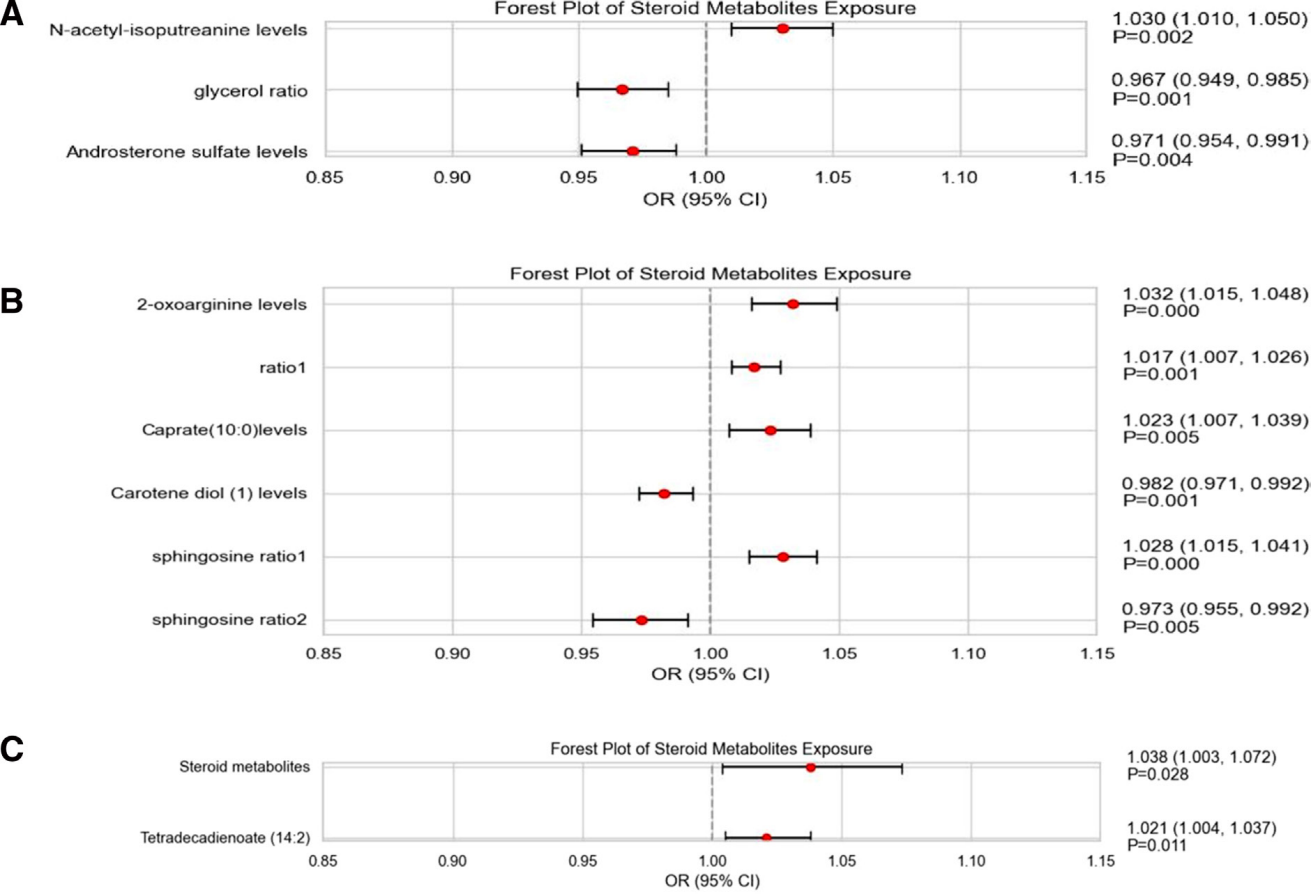
(A) Height, (B) body weight, and (C) cognitive performance. MR analysis (IVW method) of the mediator effects on height, body weight, and cognitive performance. *P* < .05. (A: Glycerol ratio: Oleoyl-linoleoyl-glycerol (18:1/18:2) [2] to linoleoyl-arachidonoyl-glycerol (18:2/20:4) [1] ratio, B: ratio1: 3-hydroxyisobutyrate to phosphate ratio, sphingosine ratio1: Leucine to *N*-palmitoyl-sphingosine (d18:1/16:0) ratio, sphingosine ratio2: *N*-palmitoyl-sphingosine (d18:1/16:0) to *N*-stearoyl-sphingosine (d18:1/18:0) ratio), and C: Steroid metabolites: 5α-Pregnan-3β,20α-diol monosulfate (2)). CI = confidence interval, IVW = inverse variance weighting, MR = Mendelian randomization, OR = odds ratio.

Figure 3 illustrates the estimated causal mediation effects of plasma metabolites on height, body weight, and cognitive performance. For height (Fig. 3A), 3 metabolites exhibited significant associations: *N-acetyl-isoputreanine* exhibited a positive correlation (OR: 1.030, 95% CI: 1.010–1.050, *P* = .003), whereas the *oleoyl-linoleoyl-glycerol (18:1/18:2) to linoleoyl-arachidonoyl-glycerol (18:2/20:4) ratio* (OR: 0.967, 95% CI: 0.949–0.985, *P* = .004) and *androsterone sulfate* (OR: 0.971, 95% CI: 0.954–0.991, *P* = .004) exhibited negative correlations. For body weight (Fig. 3B), 6 metabolites exhibited significance: *2-oxoarginine levels* (OR: 1.032, 95% CI: 1.015–1.048, *P* = .002), *3-hydroxyisobutyrate/phosphate ratio* (OR: 1.017, 95% CI: 1.007–1.026, *P* = .003), *caprate (10:0) levels* (OR: 1.023, 95% CI: 1.007–1.039, *P* = .004), and the *leucine/N-palmitoyl-sphingosine (d18:1/16:0) ratio* (OR: 1.028, 95% CI: 1.015–1.041, *P* = .004) exhibited positive associations. In contrast, *carotene diol (1) levels* (OR: 0.982, 95% CI: 0.971–0.992, *P* = .004) and the *N-palmitoyl-sphingosine (d18:1/16:0*) to *N-stearoyl-sphingosine (d18:1/18:0) ratio* (OR: 0.973, 95% CI: 0.955–0.992, *P* = .004) exhibited negative correlations. For cognitive performance (Fig. 3C), 2 plasma metabolites exhibited significant positive associations: *5α-pregnan-3β,20α-diol monosulfate (2*) (OR: 1.038, 95% CI: 1.003–1.072, *P* = .003) and *tetradecadienoate (14:2*) (OR: 1.021, 95% CI: 1.004–1.037, *P* = .004).

### 4.4. Exploratory analysis of plasma metabolites as mediators

**Figure 4.**
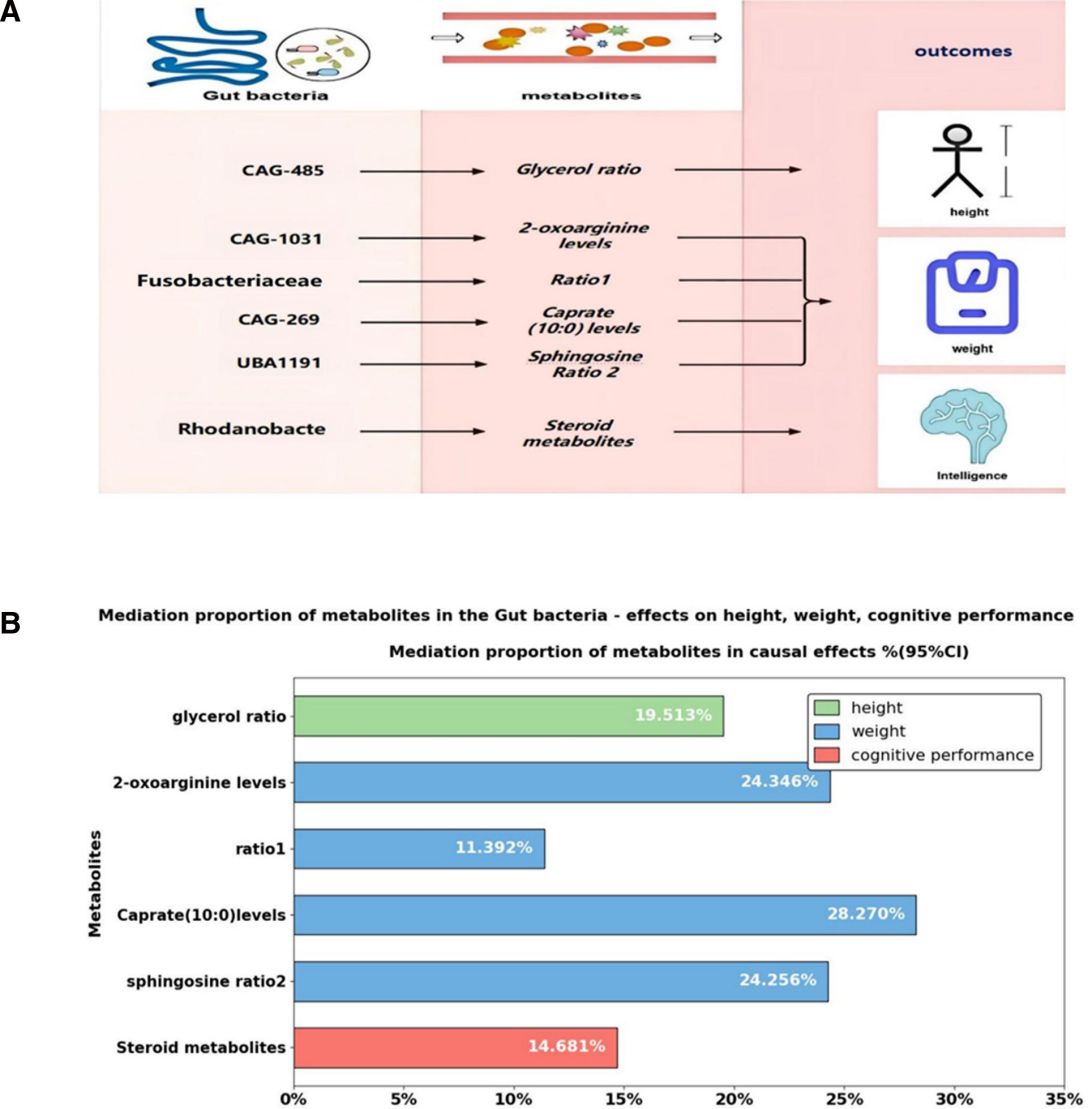
Pathway diagram and proportion of mediators by plasma metabolites. (A) Pathways by which gut microbiota influence plasma metabolite-mediated phenotypic outcomes. Image adapted from: https://bioicons.com/. (B) Quantified mediation effects of plasma metabolites in the associations between microbiota and outcomes. Height: Glycerol ratio – Oleoyl-linoleoyl-glycerol (18:1/18:2) to linoleoyl-arachidonoyl-glycerol (18:2/20:4) ratio. Body weight: ratio1: 3-hydroxyisobutyrate to phosphate ratio, sphingosine ratio2: *N*-palmitoyl-sphingosine (d18:1/16:0) to *N*-stearoyl-sphingosine (d18:1/18:0) ratio. Cognitive performance: Steroid metabolites: 5α-pregnan-3β,20α-diol monosulfate (2). CI =confidence interval.

Mediation analysis using 2-step MR was performed to evaluate whether plasma metabolites underlie gut microbiota’s effects on height, body weight, and cognitive performance. Six mediation pathways were identified (Fig. 4A). The glycerol ratio accounted for 19.513% of the mediation effect between *CAG-485* and height (Fig. 4B). Four pathways mediated body weight (Fig. 4A), including *2-oxoarginine levels* mediating *CAG-1031* (24.346%; Fig. 4B); the *3-hydroxyisobutyrate to phosphate ratio* mediating *Fusobacteriaceae* (11.392%; Fig. 4A, B); *caprate (10:0) levels* mediating *CAG-269 (sp002372935*) (28.270%; Fig. 4B); and the *N-palmitoyl-sphingosine (d18:1/16:0) to N-stearoyl-sphingosine (d18:1/18:0) ratio* mediating *UBA1191* (24.256%; Fig. 4A, B). *5α-Pregnan-3β,20α-diol monosulfate (2*) mediated the key mediating role of *Rhodobacter* in cognitive performance (14.681%; Fig. 4B). Collectively, these data support the mechanistic role of gut microbiota in influencing physical growth and neurocognitive performance via metabolite mediation.

### 4.5. Sensitivity analyses

To evaluate heterogeneity in effect estimates, Cochran *Q* statistic and its corresponding *P*-values were utilized. The significance of IVW estimates remained stable after adjusting for random effects, even with the observed heterogeneity in some analyses. We employed MR-Egger intercept-related p-values to identify potential pleiotropic effects (Tables S2, S3 and S4, Supplemental Digital Content 2). Furthermore, the robustness of the effects of the gut microbiota on height, body weight, and cognitive performance exhibited high consistency across methods when evaluated using the simple mode, MR-Egger, weighted mode, and weighted median methods. The agreement of sensitivity analyses supports the causal conclusions drawn from the main analyses.

## 5. Discussion

Herein, we employed a 2-sample, 2-step MR framework to investigate causal microbiota–host relationships and identify mediating pathways in physical growth and cognitive performance. We noted that the *oleoyl-linoleoyl-glycerol (18:1/18:2) to linoleoyl-arachidonoyl-glycerol (18:2/20:4) ratio* mediates a significant negative association between *CAG-485 (sp002362485*) and height. Furthermore, we identified four key metabolic mediators associated with body weight. Three pathways exhibited positive associations: *CAG-1031–2-oxoarginine*, *Flavonifractor (sp900199495*)–*3-hydroxyisobutyrate/phosphate ratio*, and *Fusobacteriaceae–caprate (10:0) level* pathways. In contrast, the *UBA1191–N-palmitoyl-sphingosine (d18:1/16:0) to N-stearoyl-sphingosine (d18:1/18:0) ratio* pathway exhibited a significant negative association. Among these, *caprate (10:0) levels* exerted the strongest effect on weight regulation. Furthermore, the *Rhodanobacter–5α-pregnan-3β,20α-diol monosulfate (2*) pathway was significantly associated with improved cognitive performance. Overall, the agreement between the IVW estimates and the sensitivity analysis results substantiates the robustness of the causal inference.

In the present study, we indicated that diacylglycerol may be essential for skeletal growth. Adequate bone mineral density (BMD) forms the basis for skeletal health and provides a stable structural foundation for height development. Previous studies have revealed the relationship between lipid metabolites and BMD. For example, a retrospective analysis of 710 elderly patients revealed an inverse correlation between high-density lipoprotein cholesterol (HDL-C) and BMD, potentially attributable to the role of oxysterols in osteogenic differentiation and oxysterol clearance from peripheral circulation by HDL-C. Furthermore, triglycerides exhibited a positive correlation with bone mass parameters.^[25]^ While height is primarily determined during childhood and adolescence, our dataset did not include pediatric populations; therefore, evidence from children, including the reported association between serum phospholipid DHA and BMD, serves only as a supplementary reference.^[26]^ Current bone‑related research primarily focuses on glycerolipid metabolites and BMD. However, our study provides novel insights by proposing a mechanism to directly link glycerolipid profiles to adult height. Previous studies on the associations among gut microbiota, metabolites, and obesity have primarily applied MR or clinical approaches.^[17,27]^ In contrast, research focusing on body weight in healthy populations remains scarce. Our study is the first to suggest that *CAG-1031* affects body weight by mediating *2-hydroxyarginine*. Research on branched-chain amino acids (including valine) and insulin resistance has revealed adverse effects on obesity-related weight, with the *3-hydroxyisobutyrate/phosphate ratio* identified as the key intermediate in valine catabolism that may affect energy metabolism.^[28]^ Furthermore, we observed that the flavonoid cleavage product sp900199495 mediates the relationship between this ratio and body weight. Interestingly, while mouse studies have revealed that *caprate (10:0*) can directly decrease adiposity or increase energy expenditure by activating the GPR84 receptor, the functional variability and precise in vivo mechanisms remain unclear. Our study findings suggest that *caprate (10:0) levels* affect body weight by mediating the *CAG-269* pathway.^[29,30]^ Ceramides differ in acyl-chain length from C14:0 to C30:0 and are synthesized by 6 ceramide synthases (CerS1-6). Therefore, CerS6 inhibition can serve as a targeted therapeutic strategy for obesity, with the suppression of ceramide production in obese rodents potentially reversing obesity.^[31]^ Consistent with the findings of experimental studies indicating a negative correlation with weight, our research further suggests that *UBA-1191* affects body weight by mediating the *N-palmitoyl-sphingosine (d18:1/16:0) to N-stearoyl-sphingosine (d18:1/18:0) ratio*. We noted a positive correlation between cognitive function and the *Rhodanobacter–5α-pregnan-3β,20α-diol monosulfate* pathway. As a pregnane derivative, *Rhodanobacter–5α-pregnan-3β,20α-diol monosulfate* is structurally associated with progesterone, which acts as a precursor for such compounds. In both wild-type and PRKO male mice, progesterone enhances cognitive performance, an effect that is potentially mediated by the increased expression of allopregnanolone and/or BDNF in the hippocampus, as well as increased allopregnanolone and GABA levels in the cortex.^[32]^ These findings offer novel mechanistic support for the gut–brain–microbiota axis theory.

Overall, these results highlight the particular goals for precision nutritional strategies. Diacylglycerol metabolism may be optimized to support skeletal development by adjusting dietary polyunsaturated fatty acid ratios, including the linoleic/arachidonic acid ratio. Based on the findings, the gut microbiota regulates host energy balance in weight management via various pathways, including the metabolism of amino acids (such as valine), medium-chain fatty acids (such as caprate, or through GPR84 receptor activation), and ceramide derivatives. Precision interventions may concentrate on adjusting caprate levels via microbiota-targeting strategies or dietary supplementation; controlling particular taxa (such as *Flavonifractor* or *Fusobacteriaceae*) and the pathways associated with them (ceramide synthesis or valine catabolism). Furthermore, *5α-pregnan-3β,20α-diol monosulfate* offers novel mechanistic proof of the gut–brain–microbiota axis. Nutritional interventions (such as prebiotics and particular dietary components) that target *Rhodanobacter* and neurosteroid metabolic pathways may serve as a promising avenue for improving cognitive health. Furthermore, we used European population cohorts to maximize the internal homogeneity of the study sample, thereby enhancing the validity of causal inference. However, our study has some limitations that should be acknowledged. First, caution is warranted when extrapolating the findings to non-European populations.

Our study has several notable strengths. First, metabolite-mediated pathways explained the effect of the microbiota on physical and neurocognitive development, providing mechanistic insights for future investigations. Second, by applying MR analysis, we mitigated confounding biases inherent in traditional observational studies and conducted multiple sensitivity analyses to confirm the consistency of the results. The current findings were exclusively derived from adult populations. To validate the age-dependent relationships across developmental stages, studies utilizing dynamic pediatric datasets are warranted in the future.

## 6. Conclusions

We provided genetic evidence for microbiota-targeted interventions, including probiotic supplementation or dietary fatty acid modulation, by methodically elucidating the mechanisms by which gut microbiota multidimensionally regulates human growth and cognitive performance via lipid, amino acid, and neurosteroid metabolic networks. To support the translation of customized dietary strategies to foster healthy development, future research should include longitudinal metabolomic and microbiomic analyses to clarify the causal temporal sequence of crucial metabolites and their potential in clinical translation. Prioritizing microbiota-targeted dietary interventions such as probiotics, prebiotics, and dietary fiber to modulate these lipid, amino acid, and neurosteroid pathways can be a promising strategy for preventing growth disorders and cognitive deficits while also optimizing weight control in overweight populations at risk of obesity and short stature.^[33-36]^

## Acknowledgments

The authors sincerely thank the related investigators for sharing the GWAS summary statistics included in this study.

## Author contributions

**Conceptualization:** Fang-Fang Yang, Kai-Peng Luo, Zhi-Jie Liang, Zhuo-Xin Ou, Sheng-Lan Li, Jia-Mei Liang, Yu-Nan Lin.

**Funding acquisition:** Yu-Nan Lin.

**Project administration:** Jia-Mei Liang, Yu-Nan Lin.

**Supervision:** Jia-Mei Liang, Yu-Nan Lin.

**Visualization:** Zhuo-xin Ou, Sheng-lan Li

**Writing – original draft:** Fang-Fang Yang, Kai-Peng Luo.

**Writing – review & editing:** Fang-Fang Yang, Kai-Peng Luo, Zhi-Jie Liang, Zhuo-Xin Ou, Sheng-Lan Li.














