# Supplementary material for: Causal effects of gut microbiota on physical growth and cognitive performance via plasma metabolites: A Mendelian randomization study
Source: Medicine (Baltimore). 2026 Jul 24;105(30):e49865. doi: 10.1097/MD.0000000000049865 (PMC13406128; doi:10.1097/MD.0000000000049865)
Supplement: Supplementary file 1 [file medi-105-e49865-s001.pdf]

LETTERS
https://doi.org/10.1038/s41588-018-0152-6

nature genetics

Genome-wide association meta-analysis in 269,867 individuals identifies new genetic and functional links to intelligence

Intelligence is highly heritable[1] and a major determinant of human health and well-being[2]. Recent genome-wide meta-analyses have identified 24 genomic loci linked to variation in intelligence[3-7], but much about its genetic underpinnings remains to be discovered. Here, we present a large-scale genetic association study of intelligence ($n = 269{,}867$), identifying 205 associated genomic loci (190 new) and 1,016 genes (939 new) via positional mapping, expression quantitative trait locus (eQTL) mapping, chromatin interaction mapping, and gene-based association analysis. We find enrichment of genetic effects in conserved and coding regions and associations with 146 nonsynonymous exonic variants. Associated genes are strongly expressed in the brain, specifically in striatal medium spiny neurons and hippocampal pyramidal neurons. Gene set analyses implicate pathways related to nervous system development and synaptic structure. We confirm previous strong genetic correlations with multiple health-related outcomes, and Mendelian randomization analysis results suggest protective effects of intelligence for Alzheimer's disease and ADHD and bidirectional causation with pleiotropic effects for schizophrenia. These results are a major step forward in understanding the neurobiology of cognitive function as well as genetically related neurological and psychiatric disorders.

We performed a genome-wide association study (GWAS) meta-analysis of 14 independent epidemiological cohorts of European ancestry and 9,295,118 genetic variants passing quality control (Table 1, Supplementary Fig. 1, and Supplementary Table 1). A flowchart of the study methodology is presented in Supplementary Fig. 2, and additional details of the methods and results are presented in the Supplementary Note.

Intelligence was assessed using various neurocognitive tests, primarily gauging fluid domains of cognitive functioning (Supplementary Note). Despite variation in form and content, cognitive test scores display a positive manifold of correlations, a robust empirical phenomenon that is observed in multiple populations[8]. Statistically, the variance common across cognitive tasks can be modeled as a latent factor denoted as *g* (the general factor of intelligence)[9,10]. In addition, twin and family studies show strong genetic correlations across diverse cognitive domains[11], suggesting pleiotropy, and across levels of ability[11], substantiating the view of general intelligence as an etiological continuum (with rare syndromic forms of severe intellectual disability being the exception[12]). Additionally, *g* factors extracted from different sets of cognitive tests correlate very strongly (>0.98[13,14]), supporting the universality of *g*[15,16]. In performing meta-analysis of cognitive scores obtained using a variety of tests, we aimed to boost the statistical power to detect genetic variants underlying *g*, which are likely to have pleiotropic effects across multiple domains of cognitive functioning.

Despite sample and methodological variations, genetic correlations (r_g) between cohorts were considerable (mean = 0.67), and there was no evidence of heterogeneity between cohorts in the SNP associations (Supplementary Table 2 and Supplementary Note). Age-stratified meta-analyses indicated high genetic correlations ($r_g > 0.62$) and comparable heritability across age groups, as captured by the SNPs included in the analysis ($h^2_{SNP} = 0.19$–0.22) (Supplementary Table 3 and Supplementary Note). The full-sample h^2_{SNP} was 0.19 (standard error (s.e.) = 0.01), in line with previous findings[4,5], and a linkage disequilibrium (LD) score intercept[17] of 1.08 (s.e. = 0.02) indicated that most of the inflation ($\lambda_{GC} = 1.92$) could be explained by polygenic signal[6] (Supplementary Fig. 3 and Supplementary Table 4).

In the meta-analysis, 12,110 variants indexed by 242 lead SNPs in approximate linkage equilibrium ($r^2 < 0.1$) reached genome-wide significance ($P < 5 \times 10^{-8}$) (Fig. 1a, Supplementary Figs. 4 and 5, and Supplementary Tables 5–7). These were located in 205 distinct genomic loci (Supplementary Note). We tested for replication using the proxy phenotype of educational attainment, which is correlated phenotypically (r ~0.40)[18] and genetically (r ~0.70)[19] with intelligence. We confirmed this high genetic correlation ($r_g = 0.73$) and observed sign concordance with educational attainment for 93% of genome-wide significant SNPs ($P < 1 \times 10^{-300}$), with replication for 48 loci (Supplementary Table 8 and Supplementary Note). Using polygenic score (PGS) prediction[20,21], the current results explain up to 5.2% of the variance in intelligence in four independent samples (Supplementary Table 9 and Supplementary Note).

We observed enrichment for heritability of SNPs in conserved regions ($P = 2.01 \times 10^{-12}$), coding regions ($P = 1.67 \times 10^{-6}$), and acetylated Lys9 of histone H3 (H3K9ac) histone regions/peaks ($P < 6.26 \times 10^{-5}$), and among common (minor allele frequency (MAF) > 0.3) variants (Fig. 1b, Supplementary Figs. 6 and 7, Supplementary Table 10, and Supplementary Note). Conserved and regulatory regions have previously been implicated in cognitive functioning[22], but coding regions have not.

Functional annotation of all candidate SNPs in the associated loci (SNPs with $r^2 \geq 0.6$ with one of the independent significant SNPs, a suggestive P value ($P < 1 \times 10^{-5}$), and MAF > 0.0001; $n = 21{,}368$) showed that these were mostly intronic or intergenic (Fig. 1 and Supplementary Table 6), yet 146 (81 genome-wide significant) SNPs were exonic nonsynonymous (ExNS) (Supplementary Table 11 and Supplementary Note). Convergent evidence of strong association ($z = 9.49$) and the highest observed probability of a deleterious protein effect (CADD[23] score = 34) were found for rs13107325. This missense mutation (MAF = 0.065, $P = 2.23 \times 10^{-21}$) in *SLC39A8* was the lead SNP in locus 71, and the ancestral C allele was associated with higher scores on intelligence measures. The effect sizes for ExNS SNPs were individually small, with each effect allele accounting for

A full list of authors and affiliations appears at the end of the paper.

© 2018 Nature America Inc., part of Springer Nature. All rights reserved.

Table 1 | Overview of cohorts included in a GWAS meta-analysis of general intelligence

Cohort	Cohort name	*n*	Age (years)	Phenotype
1	UKB	195,653	39–72	Verbal and mathematical reasoning
2	COGENT	35,289	8–96	One or more neuropsychological tests from three or more domains of cognitive performance
3	RS	6,182	45–98	Letter–digit substitution, Stroop, verbal fluency, delayed recall
4	GENR	1,929	5–9	SON-R (spatial visualization and abstract reasoning subsets)
5	STR	3,215	18	Logical, verbal, spatial, and technical ability subtests
6	S4S	2,818	17–18	SAT test scores
7	HiQ/HRS	9,410	NA[a]	High-IQ cases/unselected population controls
8	TEDS	3,414	12	WISC-III verbal and nonverbal reasoning; Raven's progressive matrices
9a	DTR-MADT	737	55–80	Verbal fluency, digit span, immediate and delayed recall tests
9b	DTR-LSADT	253	73–94	Verbal fluency, digit span, immediate and delayed recall tests
10	IMAGEN	1,343	14	WISC-IV, CANTAB factor score
11a	BLTS-Children	530	12–13	VSRT-C factor score
11b	BLTS-Adolescents	2,598	15–30	MAB-II IQ score
12	NESCOG	252	18–79	WAIS IQ score
13	GfG	5,084	15–91	ICAR verbal reasoning test
14a	STSA-SATSA + GENDER	703	50–94	Verbal, spatial, episodic memory, and processing speed tests
14b	STSA-HARMONY	448	65–96	Verbal, spatial, episodic memory, and processing speed tests

[a]The HiQ/HRS sample used a case–control design rather than a cognitive test score ascertained at a specific age; see the Methods and Supplementary Note.

a difference of 0.01 to 0.08 s.d. A detailed catalog of variants in the associated genomic loci is presented in Supplementary Tables 6 and 11 and in the Supplementary Note.

To link the associated variants to genes, we applied three gene-mapping strategies implemented in FUMA[24]. Positional gene mapping aligned SNPs to 522 genes by genomic location, eQTL gene mapping matched cis-eQTL SNPs to 684 genes whose expression levels they influence, and chromatin interaction mapping annotated SNPs to 227 genes on the basis of 3D DNA–DNA interactions (Fig. 2, Supplementary Figs. 8 and 9, Supplementary Tables 12–14, and Supplementary Note). This resulted in 859 unique mapped genes, 435 of which were implicated by at least two mapping strategies and 139 of which were implicated by all three (Fig. 3). Although not all of these genes are certain to have a role in intelligence, they point to potential functional links for the GWAS-associated variants and give higher credibility to genes with convergent evidence of association from multiple sources. The FUMA-mapped genes were enriched for brain tissue expression and several regulatory biological gene sets (Supplementary Note). Fifteen genes are particularly notable as they are implicated via chromatin interactions between two independent genomic risk loci (Fig. 2 and Supplementary Note). Cross-locus interactions implicated *ELAVL2*, *PTCH1*, *ATF4*, *FBXL17*, and *MAN2A1* in the left ventricle of the heart, *SATB2* in liver tissue, and *MEF2C* in five tissues. Multiple interactions in multiple tissue types were seen for a cluster of eight genes on chromosome 6 encoding zinc-finger proteins and histones.

We performed genome-wide gene-based association study (GWGAS) analysis using MAGMA[25] to estimate aggregate associations on the basis of all SNPs in a gene (whereas FUMA annotates individually significant SNPs to genes). GWGAS analysis identified 507 associated genes (Fig. 3a, Supplementary Table 15, and Supplementary Note), of which 350 were also mapped by FUMA (Fig. 3b). In total, 105 genes were implicated by all four strategies (Supplementary Table 16).

In gene set analysis, six Gene Ontology[26] gene sets were significantly associated with intelligence: neurogenesis ($P=4.78\times10^{-7}$), neuron differentiation ($P=4.82\times10^{-6}$), central nervous system neuron differentiation ($P=3.31\times10^{-6}$), regulation of nervous system development ($P=9.30\times10^{-7}$), positive regulation of nervous system development ($P=1.00\times10^{-6}$), and regulation of synapse structure or activity ($P=5.42\times10^{-6}$) (Supplementary Tables 17 and 18, and Supplementary Note). Conditional analysis indicated that there were three independent associations—regulation of nervous system development, central nervous system neuron differentiation, and regulation of synapse structure or activity—that together accounted for the associations of the other sets.

Linking gene-based *P* values to tissue-specific gene expression, we observed strong associations with gene expression across multiple brain areas (Fig. 3c, Supplementary Table 19, and Supplementary Note), particularly the frontal cortex ($P=3.10\times10^{-9}$). In brain single-cell expression gene set analyses, we found significant associations of striatal medium spiny neurons ($P=2.02\times10^{-14}$), hippocampal CA1 pyramidal neurons ($P=5.67\times10^{-11}$), and cortical somatosensory pyramidal neurons ($P=2.72\times10^{-9}$) (Fig. 3d, Supplementary Table 20, and Supplementary Note). Conditional analysis showed that the independent association signal in brain cells was driven by medium spiny neurons, neuroblasts, and pyramidal CA1 neurons.

Intelligence has been associated with a wide variety of human behaviors[15] and brain anatomy[27]. Confirming previous reports[5,6], we observed negative genetic correlations with attention deficit/hyperactivity disorder (ADHD; $r_g=-0.36$, $P=4.58\times10^{-23}$), depressive symptoms ($r_g=-0.27$, $P=6.20\times10^{-10}$), Alzheimer's disease ($r_g=-0.27$, $P=2.03\times10^{-5}$), and schizophrenia ($r_g=-0.21$, $P=3.82\times10^{-17}$) and positive correlations with longevity ($r_g=0.43$, $P=7.96\times10^{-8}$) and autism ($r_g=0.25$, $P=3.14\times10^{-7}$), among others (Supplementary Fig. 10 and Supplementary Table 21). Comparison with previous GWAS[28] supported these correlations, showing numerous shared genetic variants across phenotypes (Supplementary Tables 22 and 23, and Supplementary Note). Low enrichment (87 of 1,518 genes, $P=0.05$) was found for genes previously linked to intellectual disability or developmental delay, indicating largely distinct biological processes. However, our results extend previous genetic research on normal

© 2018 Nature America Inc., part of Springer Nature. All rights reserved.

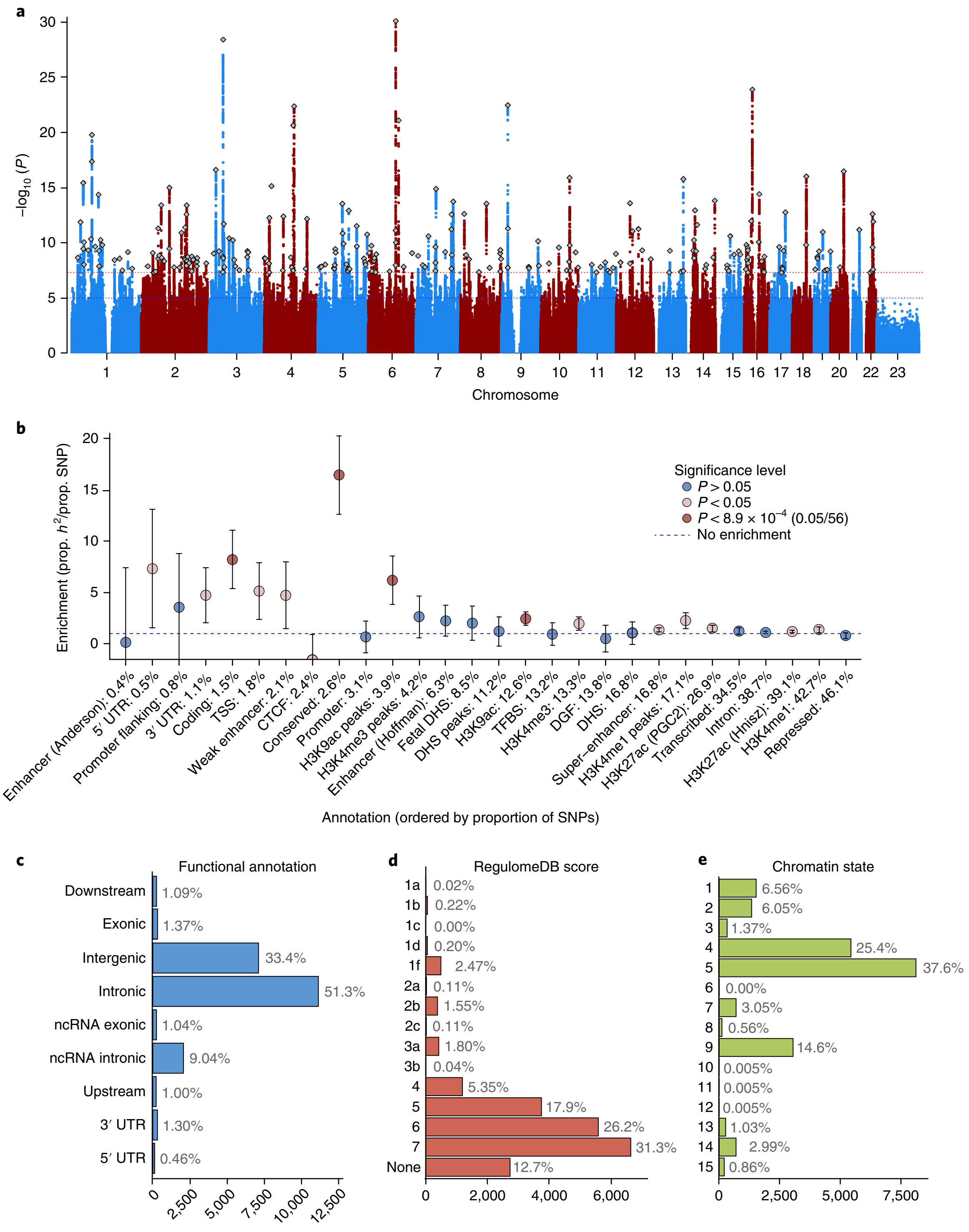

Fig. 1 | SNP-based associations with intelligence in the GWAS meta-analysis of $n = 269,867$ independent individuals. a, Manhattan plot showing the $-\log_{10}$-transformed two-tailed *P* value of each SNP from the GWAS meta-analysis (of linear and logistic regression statistics) on the *y* axis and base-pair positions along the chromosomes on the *x* axis. The dotted red line indicates Bonferroni-corrected genome-wide significance ($P < 5 \times 10^{-8}$); the dotted blue line indicates the threshold for suggestive association ($P < 1 \times 10^{-5}$). Independent lead SNPs are indicated by a diamond. **b**, Heritability enrichment of 28 functional annotation categories for SNPs in the meta-analysis, calculated with stratified LD Score regression. Error bars show 95% confidence intervals around the enrichment estimates. The dashed horizontal line indicates no enrichment of the annotation category. Red dots represent significant Bonferroni-corrected two-tailed *P* values, and beige dots represent suggestive ($P < 0.05$) values. TSS, transcription start site; CTCF, CCCTC-binding factor; DHS, DNase I–hypersensitive site; TFBS, transcription factor binding site; DGF, DNase I digital genomic footprint. **c**, Distribution of the functional consequences of SNPs in genomic risk loci in the meta-analysis. **d**, Distribution of RegulomeDB scores for SNPs in genomic risk loci, with a low score indicating a higher likelihood of the SNP having a regulatory function (Methods). **e**, The minimum chromatin state across 127 tissue and cell types for SNPs in genomic risk loci, with lower states indicating higher accessibility and states 1–7 referring to open chromatin states (Methods).

© 2018 Nature America Inc., part of Springer Nature. All rights reserved.

variation in general intelligence, as catalogued in Supplementary Tables 24 and 25.

We used Generalized Summary-data-based Mendelian randomization[29] to test for potential credible causal associations between intelligence and genetically correlated traits (Supplementary Figs. 11 and 12, Supplementary Table 26, and Supplementary Note). We observed a strong bidirectional effect of cognitive ability on educational attainment ($b_{xy}=0.549$, $P<1\times10^{-320}$) and of educational attainment on intelligence ($b_{yx}=0.480$, $P=6.85\times10^{-82}$). Such findings are consistent with previous studies implicating bidirectional causal effects[30,31]. There was also a bidirectional association showing a strong protective effect of intelligence on schizophrenia (odds ratio (OR) = 0.50, $b_{xy}=-0.685$, $P=2.02\times10^{-57}$) and a relatively smaller reverse effect ($b_{yx}=-0.214$, $P=4.19\times10^{-52}$), with additional evidence for pleiotropy (Supplementary Note). A number of previous reports support both a causal link and genetic overlap between these phenotypes[32,33]. Our results also suggested that higher intelligence had a protective effect on ADHD (OR = 0.48, $b_{xy}=-0.734$, $P=2.57\times10^{-46}$) and Alzheimer's disease (OR = 0.65, $b_{xy}=-0.435$, $P=3.59\times10^{-14}$), but was associated with higher risk of autism (OR = 1.38, $b_{xy}=0.321$, $P=1.12\times10^{-3}$).

In the present study, we have affirmed and expanded existing knowledge of the genetics of general intelligence, identifying 190 new loci and 939 new associated genes and replicating previous associations with 15 loci and 77 genes. The combined strategies of functional annotation and gene mapping using biological data resources provide extensive information on the likely consequences of relevant genetic variants and put forward a rich set of plausible gene targets and biological mechanisms for functional follow-up. Gene set analyses contribute novel insight into underlying neurobiological pathways, confirming the importance of brain-expressed genes and neurodevelopmental processes in fluid domains of intelligence and pointing toward the involvement of specific cell types. Our results indicate overlap in the genetic processes involved in both cognitive functioning and neurological and psychiatric traits and provide suggestive evidence of causal associations that may drive these correlations. These results are important for understanding the biological underpinnings of cognitive functioning and contribute to understanding of related neurological and psychiatric disorders.

URLs. UK Biobank website, http://www.ukbiobank.ac.uk/; Health and Retirement Study, http://hrsonline.isr.umich.edu/; Genes for Good, http://genesforgood.org/; International Cognitive Ability Resource measure (Genes for Good), https://icar-project.com/; Functional Mapping and Annotation (FUMA) software, http://fuma.ctglab.nl/; Multi-marker Analysis of Genomic Annotation (MAGMA) software, http://ctg.cncr.nl/software/magma; METAL software, http://genome.sph.umich.edu/wiki/METAL_Program; LD Score regression software, https://github.com/bulik/ldsc; LD Hub (GWAS summary statistics), http://ldsc.broadinstitute.org/; LD scores, https://data.broadinstitute.org/alkesgroup/LDSCORE/; GeneCards, http://www.genecards.org/; Psychiatric Genomics Consortium (GWAS summary statistics), http://www.med.unc.edu/pgc/results-and-downloads; MSigDB curated gene set database, http://software.broadinstitute.org/gsea/msigdb/collections.jsp; NHGRI GWAS catalog, https://www.ebi.ac.uk/gwas/; RegionAnnotator, https://github.com/ivankosmos/RegionAnnotator; GSMR software, http://cnsgenomics.com/software/gsmr/.

Methods

Methods, including statements of data availability and any associated accession codes and references, are available at https://doi.org/10.1038/s41588-018-0152-6.

Received: 18 September 2017; Accepted: 20 April 2018;
Published online: 25 June 2018

References

1. Polderman, T. J. et al. Meta-analysis of the heritability of human traits based on fifty years of twin studies. *Nat. Genet.* **47**, 702–709 (2015).
2. Wraw, C., Deary, I. J., Gale, C. R. & Der, G. Intelligence in youth and health at age 50. *Intelligence* **53**, 23–32 (2015).
3. Davies, G. et al. Genetic contributions to variation in general cognitive function: a meta-analysis of genome-wide association studies in the CHARGE consortium (*N* = 53949). *Mol. Psychiatry* **20**, 183–192 (2015).
4. Davies, G. et al. Genome-wide association study of cognitive functions and educational attainment in UK Biobank (*N* = 112 151). *Mol. Psychiatry* **21**, 758–767 (2016).
5. Sniekers, S. et al. Genome-wide association meta-analysis of 78,308 individuals identifies new loci and genes influencing human intelligence. *Nat. Genet.* **49**, 1107–1112 (2017).
6. Trampush, J. W. et al. GWAS meta-analysis reveals novel loci and genetic correlates for general cognitive function: a report from the COGENT consortium. *Mol. Psychiatry* **22**, 336–345 (2017).
7. Zabaneh, D. et al. A genome-wide association study for extremely high intelligence. *Mol. Psychiatry* **23**, 1226–1232 (2018).
8. Jensen, A.R. *The G Factor: The Science of Mental Ability* (Praeger, Westport, CT, US, 1998).
9. Carroll, J. B. *Human Cognitive Abilities: A Survey of Factor-Analytic Studies.* (Cambridge University Press, Cambridge, UK, 1993).
10. Spearman, C. "General intelligence," objectively determined and measured. *Am. J. Psychol.* **15**, 201–292 (1904).
11. Plomin, R. & Kovas, Y. Generalist genes and learning disabilities. *Psychol. Bull.* **131**, 592–617 (2005).
12. Plomin, R. & von Stumm, S. The new genetics of intelligence. *Nat. Rev. Genet.* **19**, 148–159 (2018).
13. Johnson, W., Bouchard, T. J., Krueger, R. F., McGue, M. & Gottesman, I. I. Just one g: consistent results from three test batteries. *Intelligence* **32**, 95–107 (2004).
14. Johnson, W., Nijenhuis, Jt & Bouchard, T. J. Still just 1 g: consistent results from five test batteries. *Intelligence* **36**, 81–95 (2008).
15. Deary, I. J., Penke, L. & Johnson, W. The neuroscience of human intelligence differences. *Nat. Rev. Neurosci.* **11**, 201–211 (2010).
16. Deary, I. J. Intelligence. *Annu. Rev. Psychol.* **63**, 453–482 (2012).
17. Bulik-Sullivan, B. K. et al. LD Score regression distinguishes confounding from polygenicity in genome-wide association studies. *Nat. Genet.* **47**, 291–295 (2015).
18. Deary, I. J., Strand, S., Smith, P. & Fernandes, C. Intelligence and educational achievement. *Intelligence* **35**, 13–21 (2007).
19. Rietveld, C. A. et al. GWAS of 126,559 individuals identifies genetic variants associated with educational attainment. *Science* **340**, 1467–1471 (2013).
20. Euesden, J., Lewis, C. M. & O'Reilly, P. F. PRSice: Polygenic Risk Score software. *Bioinformatics* **31**, 1466–1468 (2015).
21. Vilhjálmsson, B. J. et al. Modeling linkage disequilibrium increases accuracy of polygenic risk scores. *Am. J. Hum. Genet.* **97**, 576–592 (2015).
22. Hill, W. D. et al. Molecular genetic aetiology of general cognitive function is enriched in evolutionarily conserved regions. *Transl. Psychiatry* **6**, e980 (2016).
23. Kircher, M. et al. A general framework for estimating the relative pathogenicity of human genetic variants. *Nat. Genet.* **46**, 310–315 (2014).

Fig. 2 | Cross-locus interactions for genomic regions associated with intelligence in 269,867 independent individuals. a–e, Circos plots showing genes on chromosomes 2 (**a**), 5 (**b**), 6 (**c**), 9 (**d**), and 22 (**e**) that were linked to genomic risk loci in the GWAS meta-analysis (blue regions) by eQTL mapping (green lines connecting an eQTL SNP to its associated gene) and/or chromatin interactions (orange lines connecting two interacting regions) and showed evidence of interaction across two independent genomic risk loci. Genes implicated by eQTLs are in green, by chromatin interactions are in orange, and by both eQTLs and chromatin interactions are in red. The outer layer shows a Manhattan plot containing the $-\log_{10}$-transformed two-tailed P value of each SNP from the GWAS meta-analysis (of linear and logistic regression statistics), with genome-wide significant SNPs colored according to LD patterns with the lead SNP. Higher-resolution Circos plots for all chromosomes are provided in Supplementary Fig. 8.

© 2018 Nature America Inc., part of Springer Nature. All rights reserved.

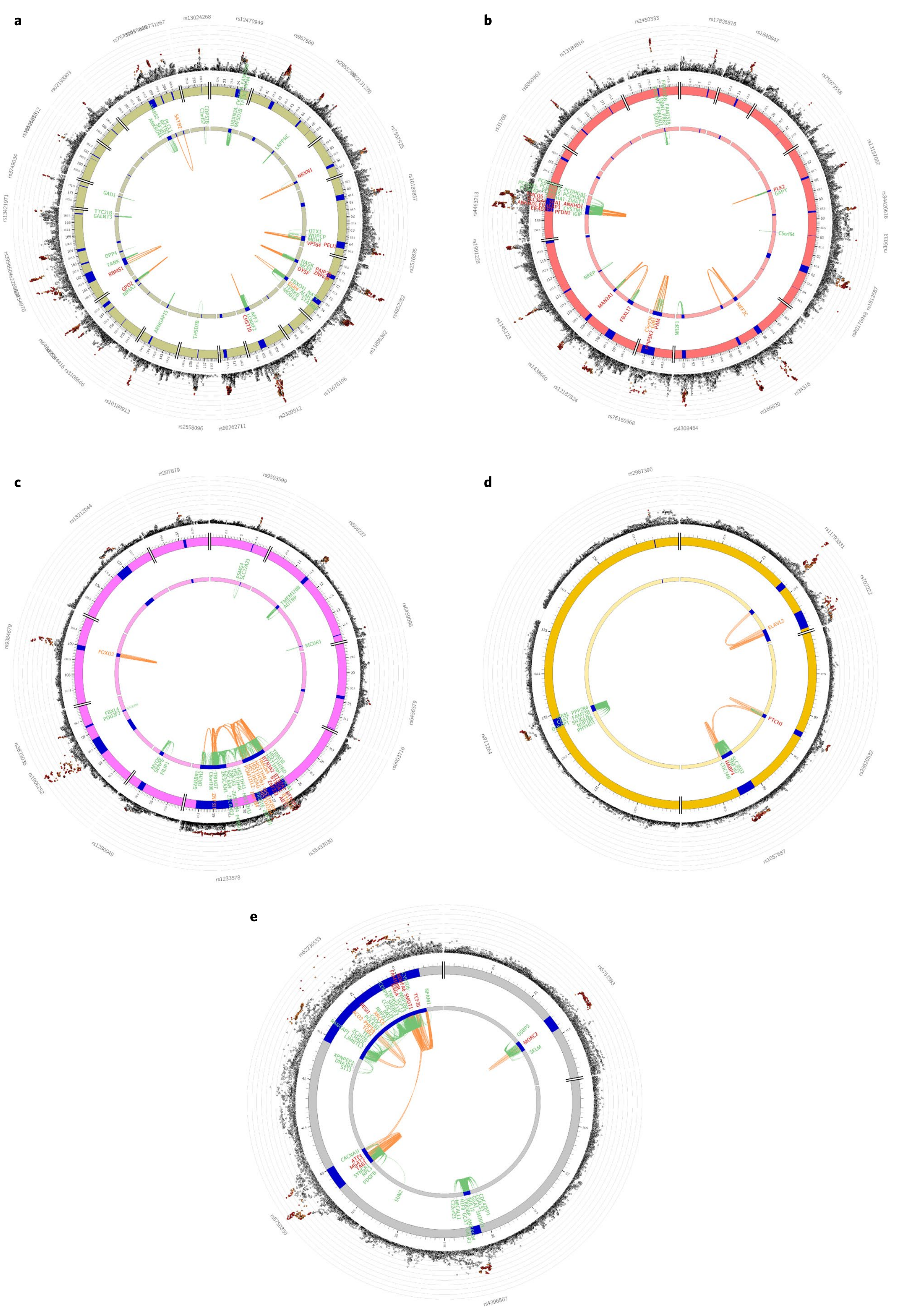

© 2018 Nature America Inc., part of Springer Nature. All rights reserved.

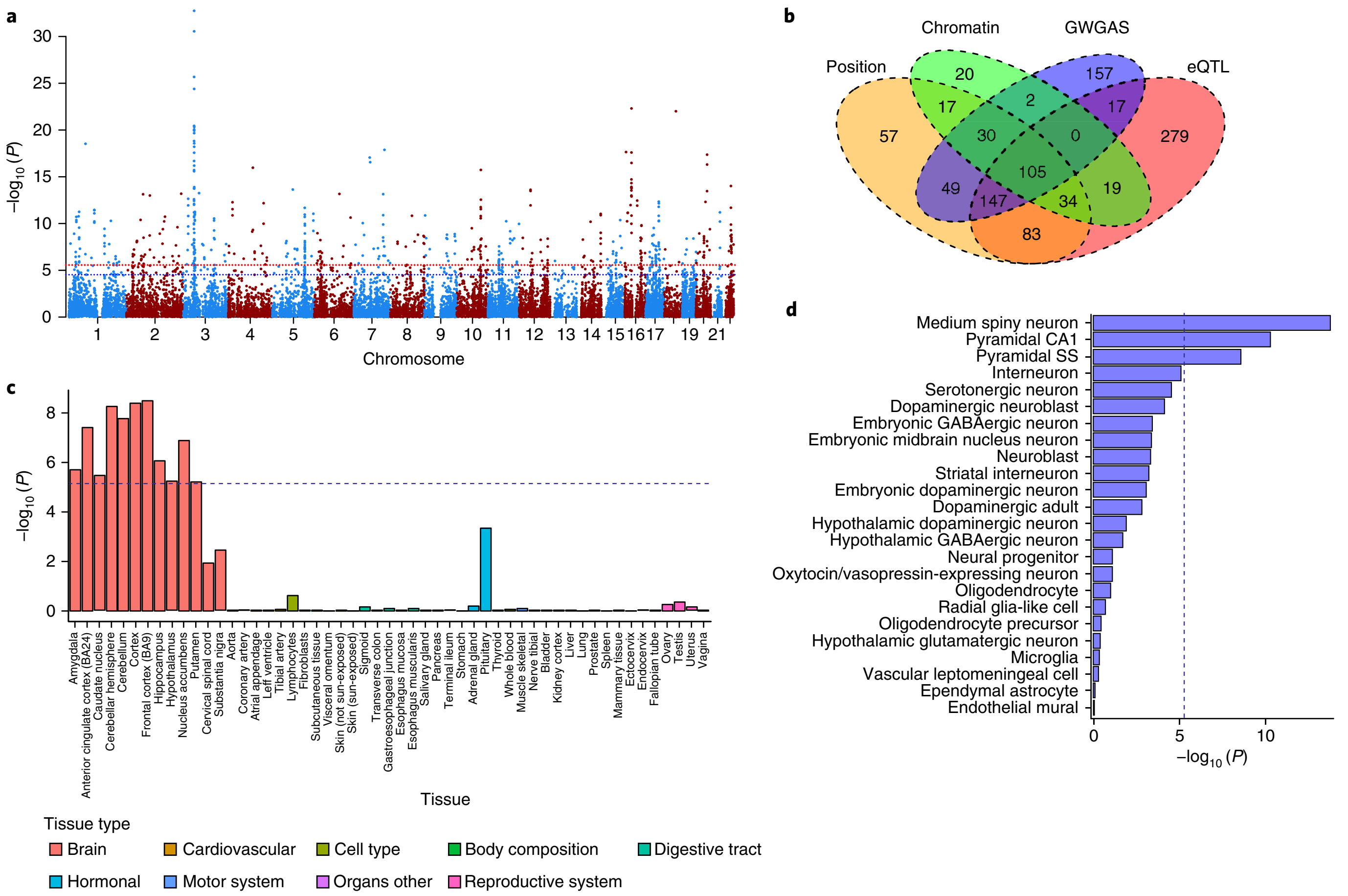

Fig. 3 | Implicated genes, pathways, and tissue and cell expression profiles for intelligence in 269,867 independent individuals. a, Manhattan plot of the GWGAS analysis. The *y* axis shows the $-\log_{10}$-transformed two-tailed *P* value of each gene from a linear model, and chromosomal position is shown on the *x* axis. The red dotted line indicates the Bonferroni-corrected threshold for genome-wide significance of the gene-based test ($P < 2.76 \times 10^{-6}$; 0.05/18,128 genes), and the blue dotted line indicates the suggestive threshold ($P < 2.76 \times 10^{-5}$; 0.5/18,128 genes). **b**, Venn diagram showing overlap of genes implicated by positional mapping, eQTL mapping, chromatin interaction mapping, and GWGAS. **c**, Gene expression profiles of associated genes in 53 tissue types. The *y* axis shows the $-\log_{10}$-transformed two-tailed *P* value of association of GWGAS test statistics with tissue-specific gene expression levels in a linear model. Expression data were extracted from the Genotype-Tissue Expression (GTEx) database. Expression values (RPKM) were $\log_2$ transformed with pseudocount 1 after Winsorization at 50 and averaged per tissue. The dashed blue line indicates the Bonferroni-corrected significance threshold ($P = 0.05/7{,}323$ gene sets $= 6.83 \times 10^{-6}$). **d**, Single-cell gene expression analysis of genes related to intelligence in 24 cell types. The *x* axis shows the $-\log_{10}$-transformed two-tailed *P* value of association of GWGAS test statistics with cell-specific gene expression levels in a linear model. The dashed blue line indicates the Bonferroni-corrected significance threshold ($P = 0.05/7{,}323$ gene sets $= 6.83 \times 10^{-6}$).

24. Watanabe, K., Taskesen, E., van Bochoven, A. & Posthuma, D. Functional mapping and annotation of genetic associations with FUMA. *Nat. Commun.* **8**, 1826 (2017).
25. de Leeuw, C. A., Mooij, J. M., Heskes, T. & Posthuma, D. MAGMA: generalized gene-set analysis of GWAS data. *PLoS Comput. Biol.* **11**, e1004219 (2015).
26. Ashburner, M. et al. Gene ontology: tool for the unification of biology. *Nat. Genet.* **25**, 25–29 (2000).
27. Posthuma, D. et al. The association between brain volume and intelligence is of genetic origin. *Nat. Neurosci.* **5**, 83–84 (2002).
28. MacArthur, J. et al. The new NHGRI-EBI Catalog of published genome-wide association studies (GWAS Catalog). *Nucleic Acids Res.* **45**(D1), D896–D901 (2017).
29. Zhu, Z. et al. Causal associations between risk factors and common diseases inferred from GWAS summary data. *Nat. Commun.* **9**, 224 (2018).
30. Johnson, W., Deary, I. J. & Iacono, W. G. Genetic and environmental transactions underlying educational attainment. *Intelligence* **37**, 466–478 (2009).
31. Richards, M. & Sacker, A. Is education causal? Yes. *Int. J. Epidemiol.* **40**, 516–518 (2011).
32. Kendler, K. S., Ohlsson, H., Sundquist, J. & Sundquist, K. IQ and schizophrenia in a Swedish national sample: their causal relationship and the interaction of IQ with genetic risk. *Am. J. Psychiatry* **172**, 259–265 (2015).
33. Le Hellard, S. et al. Identification of gene loci that overlap between schizophrenia and educational attainment. *Schizophr. Bull.* **43**, 654–664 (2017).

Acknowledgements

This work was funded by the Netherlands Organization for Scientific Research through the following grants: NWO Brain and Cognition 433-09-228 (D.P.), NWO MagW VIDI 452-12-014 (S.v.d.S.), NWO VICI 453-13-005 (D.P.), and 645-000-003 (D.P.). P.R.J. was funded by the Sophia Foundation for Scientific Research (SSWO, grant S14-27 to P.R.J.). The analyses were carried out on the Genetic Cluster Computer, which is financed by the Netherlands Scientific Organization (NWO: 480-05-003 to D.P.), Vrije Universiteit, Amsterdam, The Netherlands, and the Dutch Brain Foundation, and is hosted by the Dutch National Computing and Networking Services, SurfSARA. J.H.-L. was funded by the Swedish Research Council (Vetenskapsrådet, award 20143863), StratNeuro, the Wellcome Trust (108726/Z/15/Z), and the Swedish Brain Foundation (Hjärnfonden). N.G.S. was supported by the Wellcome Trust (108726/Z/15/Z). J.B. was funded by the Swiss National Science Foundation. This research has been conducted using the UK Biobank resource under application 16406. We thank the numerous participants, researchers, and staff from many studies who collected and contributed to the data. Additional acknowledgements can be found in the Supplementary Information.

© 2018 Nature America Inc., part of Springer Nature. All rights reserved.

Author contributions

D.P., J.E.S., and P.R.J. performed the analyses. D.P. conceived the idea of the study and supervised analyses. S. Stringer performed quality control on the UK Biobank data and wrote the analysis pipeline. K.W. constructed and applied the FUMA pipeline for performing follow-up analyses. J.B. conducted the single-cell enrichment analyses. C.A.d.L., M. Nagel, A.R. Hammerschlag, T.J.C.P., and S.v.d.S. assisted with pipeline development and data analysis. S.A., P.B.B., J.R.I.C., K.L.G., J.A.K., R.K., E. Krapohl, M.L., M. Nygaard, C.A.R., J.W.T., H.Y., D.Z., S.H., N.K.H., I.K.K., S.L., G.W.M., A.B.M.-M., E.B.Q., G.S., N.G.S., B.T.W., D.E.A., D.K., D.A., R.M.B., P.B., K.E.B., T.D.C., O.C.-F., A. Christoforou, E.T.C., E.C., A. Corvin, G. Davies, I.J.D., P.D., D.D., S.D., G. Donohoe, E.D.C., J.G.E., T.E., N.A.F., D.C.G., I.G., M.G., S.G., A.R. Hariri, A. Hatzimanolis, M.C.K., E. Knowles, B.K., J.L., S.L.-H., T.L., D.C.L., E.L., A.J.L., A.K.M., I.M., D.M., A.C.N., W.O., A. Palotie, A. Payton, N.P., R.A.P., K.R., I.R., P.R., D.R., F.W.S., M.A.S., O.B.S., N.S., J.M.S., V.M.S., N.C.S., R.E.S., K.S., A.N.V., D.R.W., E.W., J.Y., G.A., O.A.A., G.B., L.C., B.D., D.M.D., A. Heinz, J.H.-L., M.A.I., K.S.K., N.G.M., S.E.M., N.L.P., R.P., T.J.C.P., S.R., P.F.S., H.T., S.I.V., and M.J.W. contributed data. T.W. read and commented on the paper. D.P., J.E.S., and P.R.J. wrote the paper. All authors critically reviewed the paper.

Competing interests

P.F.S. reports the following potentially competing financial interests: Lundbeck (advisory committee), Pfizer (scientific advisory board member), and Roche (grant recipient, speaker reimbursement). G.B. reports consultancy and speaker fees from Eli Lilly and Illumina and grant funding from Eli Lilly. J.H.-L. reports interests from Cartana (scientific advisor) and Roche (grant recipient). T.D.C. is a consultant to Boehringer Ingelheim Pharmaceuticals and Lundbeck. All other authors declare no financial interests or potential conflicts of interest.

Additional information

Supplementary information is available for this paper at https://doi.org/10.1038/s41588-018-0152-6.

Reprints and permissions information is available at www.nature.com/reprints.

Correspondence and requests for materials should be addressed to D.P.

Publisher's note: Springer Nature remains neutral with regard to jurisdictional claims in published maps and institutional affiliations.

Jeanne E. Savage[1,89], Philip R. Jansen[1,2,89], Sven Stringer[1], Kyoko Watanabe[1], Julien Bryois[3], Christiaan A. de Leeuw[1], Mats Nagel[4], Swapnil Awasthi[5], Peter B. Barr[6], Jonathan R. I. Coleman[7,8], Katrina L. Grasby[9], Anke R. Hammerschlag[1], Jakob A. Kaminski[5,10], Robert Karlsson[3], Eva Krapohl[7], Max Lam[11], Marianne Nygaard[12,13], Chandra A. Reynolds[14], Joey W. Trampush[15,16], Hannah Young[17], Delilah Zabaneh[7], Sara Hägg[3], Narelle K. Hansell[18], Ida K. Karlsson[3], Sten Linnarsson[19], Grant W. Montgomery[9,20], Ana B. Muñoz-Manchado[19], Erin B. Quinlan[21], Gunter Schumann[21], Nathan G. Skene[19,22], Bradley T. Webb[23,24], Tonya White[2], Dan E. Arking[25], Dimitrios Avramopoulos[25,26], Robert M. Bilder[27], Panos Bitsios[28], Katherine E. Burdick[29,30,31], Tyrone D. Cannon[32], Ornit Chiba-Falek[33], Andrea Christoforou[34], Elizabeth T. Cirulli[35], Eliza Congdon[27], Aiden Corvin[36], Gail Davies[37,38], Ian J. Deary[37,38], Pamela DeRosse[39,40,41], Dwight Dickinson[42], Srdjan Djurovic[43,44], Gary Donohoe[45], Emily Drabant Conley[46], Johan G. Eriksson[47], Thomas Espeseth[48,49], Nelson A. Freimer[27], Stella Giakoumaki[50], Ina Giegling[51], Michael Gill[36], David C. Glahn[52], Ahmad R. Hariri[53], Alex Hatzimanolis[54,55,56], Matthew C. Keller[57], Emma Knowles[52], Deborah Koltai[58], Bettina Konte[51], Jari Lahti[59,60], Stephanie Le Hellard[34,44], Todd Lencz[39,40,41], David C. Liewald[38], Edythe London[27,61], Astri J. Lundervold[62,63], Anil K. Malhotra[39,40,41], Ingrid Melle[44,49], Derek Morris[45], Anna C. Need[64], William Ollier[65], Aarno Palotie[66,67,68], Antony Payton[69], Neil Pendleton[70], Russell A. Poldrack[71], Katri Räikkönen[72], Ivar Reinvang[48], Panos Roussos[29,30,73], Dan Rujescu[51], Fred W. Sabb[74], Matthew A. Scult[53], Olav B. Smeland[75], Nikolaos Smyrnis[54,55], John M. Starr[37,76], Vidar M. Steen[34,44], Nikos C. Stefanis[54,55,56], Richard E. Straub[77], Kjetil Sundet[48,49], Henning Tiemeier[2,78], Aristotle N. Voineskos[79], Daniel R. Weinberger[77], Elisabeth Widen[66], Jin Yu[39], Goncalo Abecasis[80,81], Ole A. Andreassen[49,75,82], Gerome Breen[7,8], Lene Christiansen[12,13], Birgit Debrabant[13], Danielle M. Dick[6,83,84], Andreas Heinz[5], Jens Hjerling-Leffler[19], M. Arfan Ikram[78], Kenneth S. Kendler[23,24,83], Nicholas G. Martin[9], Sarah E. Medland[9], Nancy L. Pedersen[3], Robert Plomin[7], Tinca J. C. Polderman[1], Stephan Ripke[5,85,86], Sophie van der Sluis[4], Patrick F. Sullivan[3,87], Scott I. Vrieze[17], Margaret J. Wright[18,88] and Danielle Posthuma[1,4]*

[1]Department of Complex Trait Genetics, Center for Neurogenomics and Cognitive Research, Amsterdam Neuroscience, Vrije Universiteit Amsterdam, Amsterdam, The Netherlands. [2]Department of Child and Adolescent Psychiatry, Erasmus Medical Center, Rotterdam, The Netherlands. [3]Department of Medical Epidemiology and Biostatistics, Karolinska Institutet, Stockholm, Sweden. [4]Department of Clinical Genetics, Section of Complex Trait Genetics, Neuroscience Campus Amsterdam, VU Medical Center, Amsterdam, The Netherlands. [5]Department of Psychiatry and Psychotherapy, Charité Universitätsmedizin Berlin, Campus Mitte, Berlin, Germany. [6]Department of Psychology, Virginia Commonwealth University, Richmond, VA, USA. [7]Social, Genetic, and Developmental Psychiatry Centre, Institute of Psychiatry, Psychology, and Neuroscience, King's College London, London, UK.

© 2018 Nature America Inc., part of Springer Nature. All rights reserved.

[8]NIHR Biomedical Research Centre for Mental Health, South London and Maudsley NHS Trust, London, UK. [9]QIMR Berghofer Medical Research Institute, Herston, Brisbane, Queensland, Australia. [10]Berlin Institute of Health (BIH), Berlin, Germany. [11]Institute of Mental Health, Singapore, Singapore. [12]The Danish Twin Registry and the Danish Aging Research Center, Department of Public Health, University of Southern Denmark, Odense, Denmark. [13]Epidemiology, Biostatistics, and Biodemography, Department of Public Health, University of Southern Denmark, Odense, Denmark. [14]Department of Psychology, University of California Riverside, Riverside, CA, USA. [15]BrainWorkup, LLC, Los Angeles, CA, USA. [16]Department of Psychiatry and the Behavioral Sciences, Keck School of Medicine, University of Southern California, Los Angeles, CA, USA. [17]Department of Psychology, University of Minnesota, St. Paul, MN, USA. [18]Queensland Brain Institute, University of Queensland, Brisbane, Queensland, Australia. [19]Laboratory of Molecular Neurobiology, Department of Medical Biochemistry and Biophysics, Karolinska Institutet, Stockholm, Sweden. [20]Institute for Molecular Bioscience, University of Queensland, Brisbane, Queensland, Australia. [21]Centre for Population Neuroscience and Precision Medicine (PONS), Institute of Psychiatry, Psychology, and Neuroscience, MRC-SGDP Centre, King's College London, London, UK. [22]UCL Institute of Neurology, Queen Square, London, UK. [23]Virginia Institute for Psychiatric and Behavioral Genetics, Virginia Commonwealth University, Richmond, VA, USA. [24]Department of Psychiatry, Virginia Commonwealth University, Richmond, VA, USA. [25]McKusick-Nathans Institute of Genetic Medicine, Johns Hopkins University School of Medicine, Baltimore, MD, USA. [26]Department of Psychiatry, Johns Hopkins University School of Medicine, Baltimore, MD, USA. [27]UCLA Semel Institute for Neuroscience and Human Behavior, Los Angeles, CA, USA. [28]Department of Psychiatry and Behavioral Sciences, Faculty of Medicine, University of Crete, Heraklion, Greece. [29]Department of Psychiatry, Icahn School of Medicine at Mount Sinai, New York, NY, USA. [30]Mental Illness Research, Education and Clinical Center (VISN 2), James J. Peters VA Medical Center, Bronx, NY, USA. [31]Department of Psychiatry, Brigham and Women's Hospital, Harvard Medical School, Boston, MA, USA. [32]Department of Psychology, Yale University, New Haven, CT, USA. [33]Department of Neurology, Bryan Alzheimer's Disease Research Center, and Center for Genomic and Computational Biology, Duke University Medical Center, Durham, NC, USA. [34]Dr. Einar Martens Research Group for Biological Psychiatry, Center for Medical Genetics and Molecular Medicine, Haukeland University Hospital, Bergen, Norway. [35]Human Longevity, Inc., Durham, NC, USA. [36]Neuropsychiatric Genetics Research Group, Department of Psychiatry and Trinity College Institute of Neuroscience, Trinity College Dublin, Dublin, Ireland. [37]Centre for Cognitive Ageing and Cognitive Epidemiology, University of Edinburgh, Edinburgh, UK. [38]Department of Psychology, University of Edinburgh, Edinburgh, UK. [39]Division of Psychiatry Research, Zucker Hillside Hospital, Glen Oaks, NY, USA. [40]Department of Psychiatry, Hofstra Northwell School of Medicine, Hempstead, NY, USA. [41]Center for Psychiatric Neuroscience, Feinstein Institute for Medical Research, Manhasset, NY, USA. [42]Clinical and Translational Neuroscience Branch, Intramural Research Program, National Institute of Mental Health, US National Institutes of Health, Bethesda, MD, USA. [43]Department of Medical Genetics, Oslo University Hospital, University of Bergen, Oslo, Norway. [44]NORMENT, K.G. Jebsen Centre for Psychosis Research, University of Bergen, Bergen, Norway. [45]Neuroimaging, Cognition, and Genomics (NICOG) Centre, School of Psychology and Discipline of Biochemistry, National University of Ireland, Galway, Ireland. [46]23andMe, Inc., Mountain View, CA, USA. [47]Department of General Practice and Primary Health Care, University of Helsinki and Helsinki University Hospital, Helsinki, Finland. [48]Department of Psychology, University of Oslo, Oslo, Norway. [49]Division of Mental Health and Addiction, Oslo University Hospital, Oslo, Norway. [50]Department of Psychology, University of Crete, Rethymno, Greece. [51]Department of Psychiatry, Martin Luther University of Halle-Wittenberg, Halle, Germany. [52]Department of Psychiatry, Yale University School of Medicine, New Haven, CT, USA. [53]Laboratory of NeuroGenetics, Department of Psychology and Neuroscience, Duke University, Durham, NC, USA. [54]Department of Psychiatry, National and Kapodistrian University of Athens Medical School, Eginition Hospital, Athens, Greece. [55]University Mental Health Research Institute, Athens, Greece. [56]Neurobiology Research Institute, Theodor-Theohari Cozzika Foundation, Athens, Greece. [57]Institute for Behavioral Genetics, University of Colorado, Boulder, CO, USA. [58]Psychiatry and Behavioral Sciences, Division of Medical Psychology and Department of Neurology, Duke University Medical Center, Durham, NC, USA. [59]Department of Psychology and Logopedics, Faculty of Medicine, University of Helsinki, Helsinki, Finland. [60]Helsinki Collegium for Advanced Studies, University of Helsinki, Helsinki, Finland. [61]Department of Psychiatry and Biobehavioral Sciences and Department of Molecular and Medical Pharmacology, University of California, Los Angeles, Los Angeles, CA, USA. [62]Department of Biological and Medical Psychology, University of Bergen, Bergen, Norway. [63]K.G. Jebsen Center for Research on Neuropsychiatric Disorders, University of Bergen, Bergen, Norway. [64]Division of Brain Sciences, Department of Medicine, Imperial College London, London, UK. [65]Centre for Integrated Genomic Medical Research, Institute of Population Health, University of Manchester, Manchester, UK. [66]Institute for Molecular Medicine Finland (FIMM), University of Helsinki, Helsinki, Finland. [67]Wellcome Trust Sanger Institute, Wellcome Trust Genome Campus, Cambridge, UK. [68]Center for Human Genetic Research, Psychiatric and Neurodevelopmental Genetics Unit, Massachusetts General Hospital, Boston, MA, USA. [69]Centre for Epidemiology, Division of Population Health, Health Services Research, and Primary Care, University of Manchester, Manchester, UK. [70]Division of Neuroscience and Experimental Psychology/School of Biological Sciences, Faculty of Biology Medicine and Health, University of Manchester, Manchester Academic Health Science Centre, Salford Royal NHS Foundation Trust, Manchester, UK. [71]Department of Psychology, Stanford University, Palo Alto, CA, USA. [72]Institute of Behavioural Sciences, University of Helsinki, Helsinki, Finland. [73]Department of Genetics and Genomic Science and Institute for Multiscale Biology, Icahn School of Medicine at Mount Sinai, New York, NY, USA. [74]Robert and Beverly Lewis Center for Neuroimaging, University of Oregon, Eugene, OR, USA. [75]NORMENT, K.G. Jebsen Centre for Psychosis Research, Institute of Clinical Medicine, University of Oslo and Division of Mental Health and Addiction, Oslo University Hospital, Oslo, Norway. [76]Alzheimer Scotland Dementia Research Centre, University of Edinburgh, Edinburgh, UK. [77]Lieber Institute for Brain Development, Johns Hopkins University Medical Campus, Baltimore, MD, USA. [78]Department of Epidemiology, Erasmus University Medical Center, Rotterdam, The Netherlands. [79]Campbell Family Mental Health Institute, Centre for Addiction and Mental Health, University of Toronto, Toronto, Ontario, Canada. [80]Department of Biostatistics, University of Michigan, Ann Arbor, MI, USA. [81]Center for Statistical Genetics, University of Michigan, Ann Arbor, MI, USA. [82]Institute of Clinical Medicine, University of Oslo, Oslo, Norway. [83]Department of Human and Molecular Genetics, Virginia Commonwealth University, Richmond, VA, USA. [84]College Behavioral and Emotional Health Institute, Virginia Commonwealth University, Richmond, VA, USA. [85]Analytic and Translational Genetics Unit, Massachusetts General Hospital, Boston, MA, USA. [86]Stanley Center for Psychiatric Research, Broad Institute of MIT and Harvard, Cambridge, MA, USA. [87]Department of Genetics, University of North Carolina, Chapel Hill, NC, USA. [88]Centre for Advanced Imaging, University of Queensland, Brisbane, Queensland, Australia. [89]These authors contributed equally: Jeanne E. Savage, Philip R. Jansen. *e-mail: d.posthuma@vu.nl

© 2018 Nature America Inc., part of Springer Nature. All rights reserved.

Methods

Study cohorts. The meta-analysis included new and previously reported GWAS summary statistics from 14 cohorts: UK Biobank (UKB), the Cognitive Genomics Consortium (COGENT), the Rotterdam Study (RS), the Generation R Study (GENR), the Swedish Twin Registry (STR), Spit for Science (S4S), the High-IQ/Health and Retirement Study (HiQ/HRS), the Twins Early Development Study (TEDS), the Danish Twin Registry (DTR), IMAGEN, the Brisbane Longitudinal Twin Study (BLTS), the Netherlands Study of Cognition, Environment, and Genes (NESCOG), Genes for Good (GfG), and the Swedish Twin Studies of Aging (STSA). All samples were obtained from epidemiological cohorts ascertained for research on a variety of physical and psychological outcomes. Participants ranged from children to older adults, with older samples being screened for cognitive decline to exclude the possibility of dementia affecting performance on cognitive tests.

Different measures of intelligence were assessed in each cohort but were all operationalized to index a common latent *g* factor underlying multiple dimensions of cognitive functioning. With the exception of HiQ/HRS, all cohorts extracted a single sum score, mean score, or factor score from a multidimensional set of cognitive performance tests and used this normally distributed score as the phenotype in a covariate-adjusted (for example, age, sex, ancestry principal components) GWAS using linear regression methods. For HiQ/HRS, a logistic regression GWAS was run with 'case' status reflecting whether participants were drawn from an extreme-sampled population of very high intelligence (i.e., at the upper ~0.03% of the tail of the normal distribution) versus an epidemiological sample of unselected population 'controls'. Detailed descriptions of the samples, measures, genotyping, quality control, and analysis procedures for each cohort are provided in the Supplementary Note, Supplementary Table 1, and in the Nature Research Reporting Summary.

Meta-analysis. Stringent quality control measures were applied to the summary statistics for each GWAS cohort before combining. All files were checked for data integrity and accuracy. SNPs were filtered from further analysis if they met any of the following criteria: imputation quality (INFO/R^2) score <0.6, Hardy–Weinberg equilibrium $P < 5\times10^{-6}$, study-specific minor allele frequency (MAF) corresponding to a minor allele count (MAC) <100, and mismatch of alleles or allele frequency difference greater than 20% from the Haplotype Reference Consortium (HRC) genome reference panel[16]. Some cohorts used more stringent criteria (Supplementary Note). Indels and SNPs that were duplicated, multiallelic, monomorphic, or ambiguous (A/T or C/G with MAF >0.4) were also excluded. Visual inspection of the distribution of the summary statistics was performed, and Manhattan plots and quantile–quantile plots were created for the cleaned summary statistics from each cohort (Supplementary Fig. 1).

The SNP association *P* values from the GWAS cohorts were subjected to meta-analysis with METAL[34] (see URLs) in two phases. First, we performed meta-analysis on all cohorts with quantitative phenotypes (all except HiQ/HRS) using a sample-size-weighted scheme. In the second phase, we added the HiQ/HRS study results to the results from the first phase, weighting each set of summary statistics by their respective non-centrality parameter (NCP). This method improves power when using an extreme case sampling design such as that in HiQ[35] and provides a comparable metric with which to combine information from different analytic designs while accounting for their differences in power/effective sample size. NCPs were estimated using the Genetic Power Calculator[36], as described by Coleman et al.[37]. After combining all data, meta-analysis results were further filtered to exclude any variants with $n < 50{,}000$. We additionally included a random-effects meta-analysis for each phase, as implemented in METAL, to evaluate potential heterogeneity in the SNP association statistics between cohorts.

The X chromosome was treated separately in the meta-analysis because imputed genotypes were not available for the X chromosome in the largest cohort (UKB), and there was little overlap between the UKB called genotypes and imputed data from other cohorts ($n_{\mathrm{SNPs}} < 500$). We therefore included only the called X-chromosome variants in UKB for these analyses after performing X-chromosome-specific quality-control steps[38].

We conducted a series of meta-analyses on subsets of the full sample using the same methods as above. Age-group-specific meta-analyses were run in the cohorts of children (age <17 years; GENR, TEDS, IMAGEN, BLTS; $n = 9{,}814$), young adults (age ~17–18 years; S4S, STR; $n = 6{,}033$), adults (age >18 years, primarily middle-aged or older: UKB, RS, DTR, NESCOG, STSA; $n = 204{,}228$), and older adults (mean age >60 years, RS, DTR, STSA; $n = 8{,}323$), excluding studies whose samples overlapped children/young adult and adult groups (COGENT, HiQ/HRS, GfG; $n = 49{,}792$). To create independent discovery samples for use in polygenic score validation, we also conducted meta-analyses with a 'leave-one-out' strategy in which summary statistics from four validation datasets were each excluded from the meta-analysis (see "Polygenic scoring").

Cohort heritability and genetic correlation. LD Score regression[17] was used to estimate genomic inflation and heritability of the intelligence phenotypes in each of the 14 cohorts using their post-quality-control summary statistics and to estimate the cross-cohort genetic correlations[39]. Precalculated LD scores from the 1000 Genomes European reference population were obtained online (see URLs). Genetic correlations were calculated on HapMap 3 SNPs only. LD Score regression was also used on the age-subgroup meta-analyses to estimate heritability and cross-age-group genetic correlations.

Genomic risk locus definition. Independently associated loci from the meta-analysis were defined using FUMA[24] (see URLs), an online platform for functional mapping of genetic variants. We first identified 'independent significant SNPs', which had a Bonferroni-corrected genome-wide significant two-tailed *P* value ($P < 5\times10^{-8}$) and represented signals that were independent from each other at $r^2 < 0.6$. These SNPs were further represented by 'lead SNPs', which are a subset of the independent significant SNPs that are in approximate linkage equilibrium with each other at $r^2 < 0.1$. We then defined associated 'genomic loci' by merging any physically overlapping lead SNPs (LD blocks <250 kb apart). Borders of the associated genomic loci were defined by identifying all SNPs in LD ($r^2 \geq 0.6$) with one of the independent significant SNPs in the locus, and the region containing all of these 'candidate SNPs' was considered to be a single independent genomic locus. All LD information was calculated from UKB genotype data.

Proxy replication with educational attainment. We conducted GWAS of educational attainment, an outcome with a high genetic correlation with intelligence[5], in a non-overlapping European subset of the UKB sample ($n = 188{,}435$) who did not complete the intelligence measure. Educational attainment was coded as maximum years of education completed, using the same methods as earlier analyses[40], and GWAS was conducted using the same quality-control and analytic procedures as described for the UKB intelligence phenotype (Supplementary Note). To test replication of the SNPs with this proxy phenotype, we performed a sign concordance test for all genome-wide significant SNPs from the meta-analysis using the two-tailed exact binomial test. For each independent genomic locus, we considered it to be evidence for replication if the lead SNP or another correlated SNP in the region was sign concordant with the corresponding SNP in the intelligence meta-analysis and had a two-tailed *P* value of association with educational attainment smaller than 0.05/242 independent tests = 0.0002.

Polygenic scoring. We calculated polygenic scores (PGSs) based on the SNP effect sizes of the leave-one-out meta-analyses, from which four cohorts were (separately) excluded and reserved for score validation. These included child (GENR), young adult (S4S), and adult (RS) samples. We also included the UKB-wb sample to test for validation in a very large ($n = 53{,}576$) cohort with the greatest phenotypic similarity to the largest contributor to the meta-analysis statistics (UKB-ts), to maximize potential predictive power. PGSs were calculated on the genotype data using LDpred[21], a Bayesian PGS method that uses a prior on effect size distribution to remodel the SNP effect size and account for LD, and PRSice[20], a PLINK[41]-based program that automates optimization of the set of SNPs included in the PGS based on high-resolution filtering of the GWAS *P*-value threshold. LDpred PGSs were applied to the called, cleaned, genotyped variants in each of the validation cohorts with UKB as the LD reference panel. PRSice PGSs were calculated on hard-called imputed genotypes using *P*-value thresholds from 0.0 to 0.5 in steps of 0.001. The explained variance (ΔR^2) was derived from a linear model in which the GWAS intelligence phenotype was regressed on each PGS while controlling for the same covariates as in each cohort-specific GWAS, compared to a linear model with GWAS covariates only.

Stratified heritability. We partitioned SNP heritability using stratified LD Score regression[42] in three ways: (i) by functional annotation category, (ii) by MAF in six percentile bins, and (iii) by chromosome. Annotations for 28 binary categories of putative functional genomic characteristics (for example, coding or regulatory regions) were obtained from the LD score website (see URLs). With this method, enrichment/depletion of heritability in each category is calculated as the proportion of heritability attributable to SNPs in the specified category divided by the proportion of total SNPs annotated to that category. The Bonferroni-corrected significance threshold was 0.05/56 annotations = 0.0009.

Functional annotation of SNPs. Functional annotation of SNPs implicated in the meta-analysis was performed using FUMA[24] (see URLs). We selected all candidate SNPs in associated genomic loci having $r^2 \geq 0.6$ with one of the independent significant SNPs, a suggestive *P* value ($P < 1\times10^{-5}$), and MAF >0.0001 for annotations. Predicted functional consequences for these SNPs were obtained by matching SNPs' chromosome, base-pair position, and reference and alternate alleles to databases containing known functional annotations, including ANNOVAR[43] categories, combined annotation-dependent depletion (CADD) scores[23], RegulomeDB[44] (RDB) scores, and chromatin states[45,46]. ANNOVAR categories identify the SNP's genic position (for example, intron, exon, intergenic) and associated function. CADD scores predict how deleterious the effect of a SNP is likely to be for protein structure/function, with higher scores referring to higher deleteriousness. A CADD score above 12.37 is the threshold to be potentially pathogenic[23]. The RegulomeDB score is a categorical score based on information from eQTLs and chromatin marks, ranging from 1a to 7, with lower scores indicating an increased likelihood of having a regulatory function. Scores are as follows: 1a, eQTL + transcription factor (TF) binding + matched TF

© 2018 Nature America Inc., part of Springer Nature. All rights reserved.

motif + matched DNase footprint + DNase peak; 1b, eQTL + TF binding + any motif + DNase footprint + DNase peak; 1c, eQTL + TF binding + matched TF motif + DNase peak; 1d, eQTL + TF binding + any motif + DNase peak; 1e, eQTL + TF binding + matched TF motif; 1f, eQTL + TF binding/DNase peak; 2a, TF binding + matched TF motif + matched DNase footprint + DNase peak; 2b, TF binding + any motif + DNase footprint + DNase peak; 2c, TF binding + matched TF motif + DNase peak; 3a, TF binding + any motif + DNase peak; 3b, TF binding + matched TF motif; 4, TF binding + DNase peak; 5, TF binding or DNase peak; 6, other; 7, not available. The chromatin state represents the accessibility of genomic regions (every 200 bp) with 15 categorical states predicted by a hidden Markov model based on 5 chromatin marks for 127 epigenomes in the Roadmap Epigenomics Project[46]. A lower state indicates higher accessibility, with states 1–7 referring to open chromatin states. We annotated the minimum chromatin state across tissues to SNPs. The 15 core chromatin states as suggested by Roadmap are as follows: 1, active transcription start site (TSS); 2, flanking active TSS; 3, transcription at gene 5′ and 3′ ends; 4, strong transcription; 5, weak transcription; 6, genic enhancer; 7, enhancers; 8, zinc-finger gene and repeats; 9, heterochromatic; 10, bivalent/poised TSS; 11, flanking bivalent/poised TSS/enhancer; 12, bivalent enhancer; 13, repressed Polycomb; 14, weak repressed Polycomb; 15, quiescent/low. Standardized SNP effect sizes were calculated for the SNPs with the greatest impact by transforming the sample-size-weighted meta-analysis z score, as described by Zhu et al.[47].

Gene mapping. Genome-wide significant loci obtained by the GWAS meta-analysis were mapped to genes in FUMA[24] using three strategies:

1. Positional mapping maps SNPs to genes based on physical distance (within a 10-kb window) from known protein-coding genes in the human reference assembly (GRCh37/hg19);
2. eQTL mapping maps SNPs to genes with which they show a significant eQTL association (i.e., allelic variation at the SNP is associated with the expression level of that gene). eQTL mapping uses information from 45 tissue types in 3 data repositories (GTEx[48], Blood eQTL browser[49], BIOS QTL browser[50]) and is based on cis-eQTLs that can map SNPs to genes up to 1 Mb away. We used a false discovery rate (FDR) of 0.05 to define significant eQTL associations;
3. Chromatin interaction mapping was performed to map SNPs to genes when there was a 3D DNA–DNA interaction between the SNP region and a gene region. Chromatin interaction mapping can involve long-range interactions, as it does not have a distance boundary. FUMA currently contains Hi-C data for 14 tissue types from the study of Schmitt et al.[51]. Because chromatin interactions are often defined in a certain resolution, such as 40 kb, an interacting region can span multiple genes. If a SNP is located in a region that interacts with a region containing multiple genes, it will be mapped to each of those genes. To further prioritize candidate genes, we selected only interaction-mapped genes in which one region involved in the interaction overlapped with a predicted enhancer region in any of the 111 tissue/cell types from the Roadmap Epigenomics project[46] and the other region was located in a gene promoter region (from 250 bp upstream to 500 bp downstream of the TSS and also predicted by Roadmap to be a promoter region). This reduced the number of genes mapped but increased the likelihood that those identified would have a plausible biological function. We used an FDR of 1×10^{-5} to define significant interactions, based on previous recommendations[51] modified to account for the differences in cell lines used here.

Functional annotation of mapped genes. Genes implicated by mapping of significant GWAS SNPs were further investigated using the GENE2FUNC procedure in FUMA[24], which provides hypergeometric tests of enrichment of the list of mapped genes in 53 GTEx[48] tissue-specific gene expression sets, 7,246 MSigDB gene sets[52], and 2,195 GWAS catalog gene sets[28]. The Bonferroni-corrected significance threshold was 0.05/9,494 gene sets = 5.27×10^{-6}.

Gene-based analysis. SNP-based P values from the meta-analysis were used as input for GWGAS. 18,128 protein-coding genes (each containing at least 1 GWAS SNP) from the NCBI 37.3 gene definitions were used as the basis for GWGAS in MAGMA[25] (see URLs). The Bonferroni-corrected genome-wide significance threshold was 0.05/18,128 genes = 2.76×10^{-6}.

Gene set analysis. Results from the GWGAS analyses were used to test for association in three types of predefined gene sets:

1. 7,246 curated gene sets representing known biological and metabolic pathways were derived from 9 data resources, catalogued by and obtained from MSigDB version 5.2[29] (see URLs);
2. Gene expression values from 53 tissues obtained from GTEx[48], $\log_2$ transformed with pseudocount 1 after Winsorization at 50 and averaged per tissue;
3. Cell-type-specific gene expression in 24 types of brain cells, which were calculated following the method described in Skene et al.[53] and Coleman et al.[37]. Briefly, brain-cell-type expression data were drawn from single-cell RNA-seq data from mouse brains. For each gene, the value for each cell type was calculated by dividing the mean unique molecular identifier (UMI) counts for the given cell type by the summed mean UMI counts across all cell types. Single-cell gene sets were derived by grouping genes into 40 equal bins by specificity of expression.

These gene sets were tested for association with the GWGAS gene-based test statistics using MAGMA. We computed competitive P values, which represent the test of association for a specific gene set in comparison to other gene sets. This method is more robust to type I error than self-contained tests that only test for association of a gene set against the null hypothesis of no association[25]. The Bonferroni-corrected significance threshold was 0.05/7,323 gene sets = 6.83×10^{-6}. Conditional analyses were performed as a follow-up using MAGMA to test whether each significant association observed was independent of all others. The association between each gene set was tested conditional on the most strongly associated set, and then—if any substantial ($P < 0.05$/number of gene sets) associations remained—by conditioning on the first and second most strongly associated set, and so on until no associations remained. Gene sets that retained their association after correcting for other sets were considered to be independent signals. We note that this is not a test of association per se, but rather a strategy to identify, among gene sets with known significant associations whose defining genes may overlap, which set(s) are responsible for driving the observed association.

Cross-trait genetic correlation. Genetic correlations (r_g) between intelligence and 38 phenotypes were computed using LD Score regression[39], as described above, based on GWAS summary statistics obtained from publicly available databases (see URLs; Supplementary Table 18). The Bonferroni-corrected significance threshold was 0.05/38 traits = 1.32×10^{-3}.

GWAS catalog lookup. We used FUMA to identify SNPs with previously reported ($P < 5 \times 10^{-5}$) phenotypic associations in published GWAS listed in the NHGRI-EBI catalog[28] that overlapped with the genomic risk loci identified in the meta-analysis. As an additional relevant phenotype of interest, we examined whether the genes associated with intelligence in this study (by FUMA mapping or GWGAS) were over-represented in a set of 1,518 genes linked to intellectual disability and/or developmental delay, as compiled by RegionAnnotater (see URLs). Many of these have been identified by non-GWAS sources and are not represented in the NHGRI catalog. We tested for enrichment using a hypergeometric test with a background set of 19,283 genomic protein-coding genes, as in FUMA. Manual lookups were also performed to identify overlapping loci/genes with known previous GWAS of intelligence.

Mendelian randomization. To infer credible causal associations between intelligence and traits that are genetically correlated with intelligence, we performed generalized summary-data-based Mendelian randomization[29] (GSMR; see URLs). This method uses summary-level data to test for causal associations between a putative risk factor (exposure) and an outcome by using independent genome-wide significant SNPs as instrumental variables. HEIDI outlier detection was used to filter genetic instruments that showed clear pleiotropic effects on both the exposure phenotype and the outcome phenotype. We used a threshold P value of 0.01 for the outlier detection analysis in HEIDI, which removes 1% of SNPs by chance if there is no pleiotropic effect. To test for a potential causal effect of intelligence on various outcomes, we selected traits in non-overlapping samples that showed significant genetic correlations (r_g) with intelligence. We tested for bidirectional causation by repeating the analyses while switching the role of each correlated phenotype as an exposure and intelligence as the outcome. For each trait, we selected independent ($r^2 \leq 0.1$), genome-wide significant lead SNPs as instrumental variables in the analyses. For traits with fewer than ten genome-wide significant lead SNPs (i.e., the minimum number of SNPs on which GSMR can perform a reliable analysis), the genome-wide significance threshold was lowered to 1×10^{-5}, allowing a sufficient number of SNPs to conduct the reverse GSMR analysis for former smoker status, autism, intracranial volume, and ADHD.

The method estimates a putative causal effect of the exposure on the outcome (b_{xy}) as a function of the relationship between the SNPs' effects on the exposure (b_{zx}) and the SNPs' effects on the outcome (b_{zy}), given the assumption that the effect of non-pleiotropic SNPs on an exposure (x) should be related to their effect on the outcome (y) in an independent sample only via mediation through the phenotypic causal pathway (b_{xy}). The estimated causal effect coefficients (b_{xy}) are approximately equal to the natural log odds ratio (OR) for a case–control trait[29]. An OR of 2 can be interpreted as a doubled risk in comparison to the population prevalence of a binary trait for every s.d. increase in the exposure trait. For quantitative traits, b_{xy} can be interpreted as a 1 s.d. increase explained in the outcome trait for every s.d. increase in the exposure trait. This method can help differentiate the likely causal direction of association between two traits but cannot make any statement about the intermediate mechanisms involved in any potential causal process.

Reporting Summary. Further information on experimental design is available in the Nature Research Reporting Summary linked to this article.

Data availability. Summary statistics are available for download from https://ctg.cncr.nl/.

© 2018 Nature America Inc., part of Springer Nature. All rights reserved.

References

34. Willer, C. J., Li, Y. & Abecasis, G. R. METAL: fast and efficient meta-analysis of genomewide association scans. *Bioinformatics* **26**, 2190–2191 (2010).
35. Peloso, G. M. et al. Phenotypic extremes in rare variant study designs. *Eur. J. Hum. Genet.* **24**, 924–930 (2016).
36. Purcell, S., Cherny, S. S. & Sham, P. C. Genetic Power Calculator: design of linkage and association genetic mapping studies of complex traits. *Bioinformatics* **19**, 149–150 (2003).
37. Coleman, J.R.I. *et al.* Biological annotation of genetic loci associated with intelligence in a meta-analysis of 87,740 individuals. *Mol. Psychiatry* https://doi.org/10.1038/s41380-018-0040-6 (2018).
38. König, I. R., Loley, C., Erdmann, J. & Ziegler, A. How to include chromosome X in your genome-wide association study. *Genet. Epidemiol.* **38**, 97–103 (2014).
39. Bulik-Sullivan, B. et al. An atlas of genetic correlations across human diseases and traits. *Nat. Genet.* **47**, 1236–1241 (2015).
40. Okbay, A. et al. Genome-wide association study identifies 74 loci associated with educational attainment. *Nature* **533**, 539–542 (2016).
41. Chang, C. C. et al. Second-generation PLINK: rising to the challenge of larger and richer datasets. *Gigascience* **4**, 7 (2015).
42. Finucane, H. K. et al. Partitioning heritability by functional annotation using genome-wide association summary statistics. *Nat. Genet.* **47**, 1228–1235 (2015).
43. Wang, K., Li, M. & Hakonarson, H. ANNOVAR: functional annotation of genetic variants from high-throughput sequencing data. *Nucleic Acids Res.* **38**, e164 (2010).
44. Boyle, A. P. et al. Annotation of functional variation in personal genomes using RegulomeDB. *Genome Res.* **22**, 1790–1797 (2012).
45. Ernst, J. & Kellis, M. ChromHMM: automating chromatin-state discovery and characterization. *Nat. Methods* **9**, 215–216 (2012).
46. Kundaje, A. et al. Integrative analysis of 111 reference human epigenomes. *Nature* **518**, 317–330 (2015).
47. Zhu, Z. et al. Integration of summary data from GWAS and eQTL studies predicts complex trait gene targets. *Nat. Genet.* **48**, 481–487 (2016).
48. GTEx Consortium. The Genotype-Tissue Expression (GTEx) pilot analysis: multitissue gene regulation in humans. *Science* **348**, 648–660 (2015).
49. Westra, H. J. et al. Systematic identification of trans eQTLs as putative drivers of known disease associations. *Nat. Genet.* **45**, 1238–1243 (2013).
50. Zhernakova, D. V. et al. Identification of context-dependent expression quantitative trait loci in whole blood. *Nat. Genet.* **49**, 139–145 (2017).
51. Schmitt, A. D. et al. A compendium of chromatin contact maps reveals spatially active regions in the human genome. *Cell Rep.* **17**, 2042–2059 (2016).
52. Liberzon, A. et al. Molecular signatures database (MSigDB) 3.0. *Bioinformatics* **27**, 1739–1740 (2011).
53. Skene, N. G. et al. Genetic identification of brain cell types underlying schizophrenia. *Nat. Genet.* **50**, 825–833 (2018).

© 2018 Nature America Inc., part of Springer Nature. All rights reserved.

nature research

Corresponding author(s): Danielle Posthuma

☐ Initial submission ☐ Revised version ☒ Final submission

Life Sciences Reporting Summary

Nature Research wishes to improve the reproducibility of the work that we publish. This form is intended for publication with all accepted life science papers and provides structure for consistency and transparency in reporting. Every life science submission will use this form; some list items might not apply to an individual manuscript, but all fields must be completed for clarity.

For further information on the points included in this form, see Reporting Life Sciences Research. For further information on Nature Research policies, including our data availability policy, see Authors & Referees and the Editorial Policy Checklist.

Experimental design

1. Sample size

Describe how sample size was determined.

Data used in this study was collected by external sources and used for secondary analysis. Sample size was not pre-determined and was chosen based on all known available cohorts with relevant data collected to date, after quality control steps were performed in each cohort (described in detail in Online Methods section "Study Cohorts" and Supplementary Information section 1.1). Power calculations using the Genetic Power Calculator indicated that we had virtually 100% power to detect SNPs accounting for >0.05% of trait variance in our sample size of N=269867.

2. Data exclusions

Describe any data exclusions.

Each cohort in the meta-analysis applied their own quality control criteria (described in Supplementary Information section 1.1), with samples being excluded for study design purposes (i.e. based on age), criteria that would affect normal intelligence scores such as the presence of dementia, poor quality DNA sample, or if consent was withdrawn after enrollment. Genetic variants were excluded using standard quality control metrics. At the meta-analysis level, we applied a set of pre-determined quality control filters for the removal of SNPs based on minor allele frequency, imputation quality, mismatch from a known reference panel, etc. as described fully in the Online Methods (section "Meta-analysis").

3. Replication

Describe whether the experimental findings were reliably reproduced.

The meta-analysis strategy includes replication by default, as it weights the reported test statistics by the evidence of association across multiple samples. Further, SNP-based replication was carried out using a proxy phenotype of educational attainment in an independent sample. Aggregate genomic associations were replicated using polygenic score validation in four holdout samples.

4. Randomization

Describe how samples/organisms/participants were allocated into experimental groups.

Not relevant; no experimental procedures were performed as this was a study of genetic association between genotypes and non-manipulated phenotypes.

5. Blinding

Describe whether the investigators were blinded to group allocation during data collection and/or analysis.

Not relevant; there were no experimental groups.

Note: all studies involving animals and/or human research participants must disclose whether blinding and randomization were used.

6. Statistical parameters

For all figures and tables that use statistical methods, confirm that the following items are present in relevant figure legends (or in the Methods section if additional space is needed).

n/a	Confirmed	
☐	☒	The exact sample size (*n*) for each experimental group/condition, given as a discrete number and unit of measurement (animals, litters, cultures, etc.)
☐	☒	A description of how samples were collected, noting whether measurements were taken from distinct samples or whether the same sample was measured repeatedly
☒	☐	A statement indicating how many times each experiment was replicated
☐	☒	The statistical test(s) used and whether they are one- or two-sided (note: only common tests should be described solely by name; more complex techniques should be described in the Methods section)
☐	☒	A description of any assumptions or corrections, such as an adjustment for multiple comparisons
☐	☒	The test results (e.g. *P* values) given as exact values whenever possible and with confidence intervals noted
☐	☒	A clear description of statistics including central tendency (e.g. median, mean) and variation (e.g. standard deviation, interquartile range)
☐	☒	Clearly defined error bars

See the web collection on statistics for biologists for further resources and guidance.

Software

Policy information about availability of computer code

7. Software

Describe the software used to analyze the data in this study.	We used standard, publicly available statistical genetics software packages, which are described and linked to in the Online Methods. The packages we used included:<br><br>R - Data management and statistical analyses (R Core Team, 2016)<br>SHAPEIT2/IMPUTE2 - Genotype imputation (Howie et al., 2009)<br>PLINK - Genetic association testing (Purcell et al., 2007; Chang et al., 2015)<br>SNPTEST - Genetic association testing (Marchini et al., 2007)<br>RAREMETALWORKER - Genetic association testing in related individuals (Liu et al., 2014)<br>METAL - GWAS meta-analysis (Willer et al., 2010)<br>FUMA - Online platform for functional annotation of GWAS results (Watanabe et al., 2017)<br>MAGMA - Gene-based association testing (de Leeuw et al., 2015)<br>LD score regression - SNP-based heritability and genetic correlations from GWAS summary statistics (Bulik-Sullivan et al., 2015)<br>PRSice - Polygenic score analysis (Eusden et al., 2015)<br>LDpred - Polygenic score analysis (Vilhjalmsson et al., 2015)

For manuscripts utilizing custom algorithms or software that are central to the paper but not yet described in the published literature, software must be made available to editors and reviewers upon request. We strongly encourage code deposition in a community repository (e.g. GitHub). *Nature Methods* guidance for providing algorithms and software for publication provides further information on this topic.

Materials and reagents

Policy information about availability of materials

8. Materials availability

Indicate whether there are restrictions on availability of unique materials or if these materials are only available for distribution by a for-profit company.	Summary statistics from the GWAS meta-analysis will be made freely available for download at https://ctg.cncr.nl/.

9. Antibodies

Describe the antibodies used and how they were validated for use in the system under study (i.e. assay and species).	No antibodies were used in this study.

10. Eukaryotic cell lines

a. State the source of each eukaryotic cell line used.

No eukaryotic cell lines were used in this study.

b. Describe the method of cell line authentication used.

No eukaryotic cell lines were used in this study.

c. Report whether the cell lines were tested for mycoplasma contamination.

No eukaryotic cell lines were used in this study.

d. If any of the cell lines used are listed in the database of commonly misidentified cell lines maintained by ICLAC, provide a scientific rationale for their use.

No eukaryotic cell lines were used in this study.

Animals and human research participants

Policy information about studies involving animals; when reporting animal research, follow the ARRIVE guidelines

11. Description of research animals

Provide details on animals and/or animal-derived materials used in the study.

No animals were used in this study.

Policy information about studies involving human research participants

12. Description of human research participants

Describe the covariate-relevant population characteristics of the human research participants.

Human research participants of all ages (approximately 6 too 100 years old) were included, with approximately equal proportions of males and females. Participants came from 14 independent cohorts previously collected by external sources, which were defined by different criteria based on the original study goals but were generally population-based cohorts of healthy individuals. Participants were not included/excluded for specific criteria except the presence of Alzheimer's disease, dementia, or other cognitive impairments. Population characteristics of the sample are described extensively in the Supplementary Information section 1.1 and summarized in Supplementary Table 1.
