# Supplementary figures and images for: Causal effects of gut microbiota on physical growth and cognitive performance via plasma metabolites: A Mendelian randomization study
Source: Medicine (Baltimore). 2026 Jul 24;105(30):e49865. doi: 10.1097/MD.0000000000049865 (PMC13406128; doi:10.1097/MD.0000000000049865)

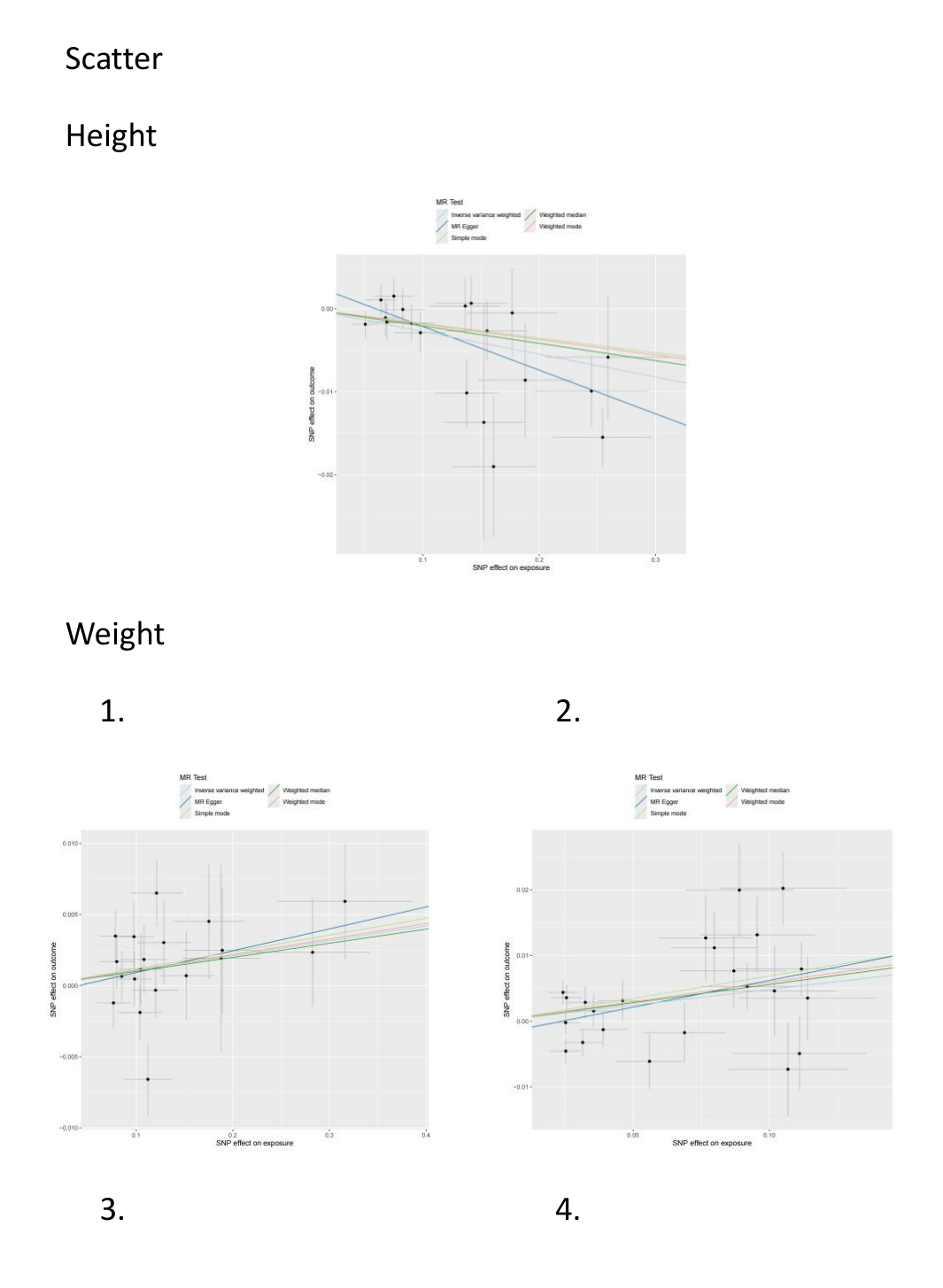

Supplement: Supplementary file 6 [file medi-105-e49865-s006.tiff]
