# Supplementary material for: Causal effects of gut microbiota on physical growth and cognitive performance via plasma metabolites: A Mendelian randomization study
Source: Medicine (Baltimore). 2026 Jul 24;105(30):e49865. doi: 10.1097/MD.0000000000049865 (PMC13406128; doi:10.1097/MD.0000000000049865)
Supplement: Supplementary file 7 [file medi-105-e49865-s007.pdf]

nature genetics
SUPPLEMENTARY INFORMATION
https://doi.org/10.1038/s41588-018-0152-6
In the format provided by the authors and unedited.

Genome-wide association meta-analysis in 269,867 individuals identifies new genetic and functional links to intelligence

Jeanne E. Savage[1,89], Philip R. Jansen[1,2,89], Sven Stringer[1], Kyoko Watanabe[1], Julien Bryois[3], Christiaan A. de Leeuw[1], Mats Nagel[4], Swapnil Awasthi[5], Peter B. Barr[6], Jonathan R. I. Coleman[7,8], Katrina L. Grasby[9], Anke R. Hammerschlag[1], Jakob A. Kaminski[5,10], Robert Karlsson[3], Eva Krapohl[7], Max Lam[11], Marianne Nygaard[12,13], Chandra A. Reynolds[14], Joey W. Trampush[15,16], Hannah Young[17], Delilah Zabaneh[7], Sara Hägg[3], Narelle K. Hansell[18], Ida K. Karlsson[3], Sten Linnarsson[19], Grant W. Montgomery[9,20], Ana B. Muñoz-Manchado[19], Erin B. Quinlan[21], Gunter Schumann[21], Nathan G. Skene[19,22], Bradley T. Webb[23,24], Tonya White[2], Dan E. Arking[25], Dimitrios Avramopoulos[25,26], Robert M. Bilder[27], Panos Bitsios[28], Katherine E. Burdick[29,30,31], Tyrone D. Cannon[32], Ornit Chiba-Falek[33], Andrea Christoforou[34], Elizabeth T. Cirulli[35], Eliza Congdon[27], Aiden Corvin[36], Gail Davies[37,38], Ian J. Deary[37,38], Pamela DeRosse[39,40,41], Dwight Dickinson[42], Srdjan Djurovic[43,44], Gary Donohoe[45], Emily Drabant Conley[46], Johan G. Eriksson[47], Thomas Espeseth[48,49], Nelson A. Freimer[27], Stella Giakoumaki[50], Ina Giegling[51], Michael Gill[36], David C. Glahn[52], Ahmad R. Hariri[53], Alex Hatzimanolis[54,55,56], Matthew C. Keller[57], Emma Knowles[52], Deborah Koltai[58], Bettina Konte[51], Jari Lahti[59,60], Stephanie Le Hellard[34,44], Todd Lencz[39,40,41], David C. Liewald[38], Edythe London[27,61], Astri J. Lundervold[62,63], Anil K. Malhotra[39,40,41], Ingrid Melle[44,49], Derek Morris[45], Anna C. Need[64], William Ollier[65], Aarno Palotie[66,67,68], Antony Payton[69], Neil Pendleton[70], Russell A. Poldrack[71], Katri Räikkönen[72], Ivar Reinvang[48], Panos Roussos[29,30,73], Dan Rujescu[51], Fred W. Sabb[74], Matthew A. Scult[53], Olav B. Smeland[75], Nikolaos Smyrnis[54,55], John M. Starr[37,76], Vidar M. Steen[34,44], Nikos C. Stefanis[54,55,56], Richard E. Straub[77], Kjetil Sundet[48,49], Henning Tiemeier[2,78], Aristotle N. Voineskos[79], Daniel R. Weinberger[77], Elisabeth Widen[66], Jin Yu[39], Goncalo Abecasis[80,81], Ole A. Andreassen[49,75,82], Gerome Breen[7,8], Lene Christiansen[12,13], Birgit Debrabant[13], Danielle M. Dick[6,83,84], Andreas Heinz[5], Jens Hjerling-Leffler[19], M. Arfan Ikram[78], Kenneth S. Kendler[23,24,83], Nicholas G. Martin[9], Sarah E. Medland[9], Nancy L. Pedersen[3], Robert Plomin[7], Tinca J. C. Polderman[1], Stephan Ripke[5,85,86], Sophie van der Sluis[4], Patrick F. Sullivan[3,87], Scott I. Vrieze[17], Margaret J. Wright[18,88] and Danielle Posthuma[1,4]*

[1]Department of Complex Trait Genetics, Center for Neurogenomics and Cognitive Research, Amsterdam Neuroscience, Vrije Universiteit Amsterdam, Amsterdam, The Netherlands. [2]Department of Child and Adolescent Psychiatry, Erasmus Medical Center, Rotterdam, The Netherlands. [3]Department of Medical Epidemiology and Biostatistics, Karolinska Institutet, Stockholm, Sweden. [4]Department of Clinical Genetics, Section of Complex Trait Genetics, Neuroscience Campus Amsterdam, VU Medical Center, Amsterdam, The Netherlands. [5]Department of Psychiatry and Psychotherapy, Charité Universitätsmedizin Berlin, Campus Mitte, Berlin, Germany. [6]Department of Psychology, Virginia Commonwealth University, Richmond, VA, USA. [7]Social, Genetic, and Developmental Psychiatry Centre, Institute of Psychiatry, Psychology, and Neuroscience, King's College London, London, UK. [8]NIHR Biomedical Research Centre for Mental Health, South London and Maudsley NHS Trust, London, UK. [9]QIMR Berghofer Medical Research Institute, Herston, Brisbane, Queensland, Australia. [10]Berlin Institute of Health (BIH), Berlin, Germany. [11]Institute of Mental Health, Singapore, Singapore. [12]The Danish Twin Registry and the Danish Aging Research Center, Department of Public Health, University of Southern Denmark, Odense, Denmark. [13]Epidemiology, Biostatistics, and Biodemography, Department of Public Health, University of Southern Denmark, Odense, Denmark. [14]Department of Psychology, University of California Riverside, Riverside, CA, USA. [15]BrainWorkup, LLC, Los Angeles, CA, USA. [16]Department of Psychiatry and the

© 2018 Nature America Inc., part of Springer Nature. All rights reserved.

Behavioral Sciences, Keck School of Medicine, University of Southern California, Los Angeles, CA, USA. [17]Department of Psychology, University of Minnesota, St. Paul, MN, USA. [18]Queensland Brain Institute, University of Queensland, Brisbane, Queensland, Australia. [19]Laboratory of Molecular Neurobiology, Department of Medical Biochemistry and Biophysics, Karolinska Institutet, Stockholm, Sweden. [20]Institute for Molecular Bioscience, University of Queensland, Brisbane, Queensland, Australia. [21]Centre for Population Neuroscience and Precision Medicine (PONS), Institute of Psychiatry, Psychology, and Neuroscience, MRC-SGDP Centre, King's College London, London, UK. [22]UCL Institute of Neurology, Queen Square, London, UK. [23]Virginia Institute for Psychiatric and Behavioral Genetics, Virginia Commonwealth University, Richmond, VA, USA. [24]Department of Psychiatry, Virginia Commonwealth University, Richmond, VA, USA. [25]McKusick-Nathans Institute of Genetic Medicine, Johns Hopkins University School of Medicine, Baltimore, MD, USA. [26]Department of Psychiatry, Johns Hopkins University School of Medicine, Baltimore, MD, USA. [27]UCLA Semel Institute for Neuroscience and Human Behavior, Los Angeles, CA, USA. [28]Department of Psychiatry and Behavioral Sciences, Faculty of Medicine, University of Crete, Heraklion, Greece. [29]Department of Psychiatry, Icahn School of Medicine at Mount Sinai, New York, NY, USA. [30]Mental Illness Research, Education and Clinical Center (VISN 2), James J. Peters VA Medical Center, Bronx, NY, USA. [31]Department of Psychiatry, Brigham and Women's Hospital, Harvard Medical School, Boston, MA, USA. [32]Department of Psychology, Yale University, New Haven, CT, USA. [33]Department of Neurology, Bryan Alzheimer's Disease Research Center, and Center for Genomic and Computational Biology, Duke University Medical Center, Durham, NC, USA. [34]Dr. Einar Martens Research Group for Biological Psychiatry, Center for Medical Genetics and Molecular Medicine, Haukeland University Hospital, Bergen, Norway. [35]Human Longevity, Inc., Durham, NC, USA. [36]Neuropsychiatric Genetics Research Group, Department of Psychiatry and Trinity College Institute of Neuroscience, Trinity College Dublin, Dublin, Ireland. [37]Centre for Cognitive Ageing and Cognitive Epidemiology, University of Edinburgh, Edinburgh, UK. [38]Department of Psychology, University of Edinburgh, Edinburgh, UK. [39]Division of Psychiatry Research, Zucker Hillside Hospital, Glen Oaks, NY, USA. [40]Department of Psychiatry, Hofstra Northwell School of Medicine, Hempstead, NY, USA. [41]Center for Psychiatric Neuroscience, Feinstein Institute for Medical Research, Manhasset, NY, USA. [42]Clinical and Translational Neuroscience Branch, Intramural Research Program, National Institute of Mental Health, US National Institutes of Health, Bethesda, MD, USA. [43]Department of Medical Genetics, Oslo University Hospital, University of Bergen, Oslo, Norway. [44]NORMENT, K.G. Jebsen Centre for Psychosis Research, University of Bergen, Bergen, Norway. [45]Neuroimaging, Cognition, and Genomics (NICOG) Centre, School of Psychology and Discipline of Biochemistry, National University of Ireland, Galway, Ireland. [46]23andMe, Inc., Mountain View, CA, USA. [47]Department of General Practice and Primary Health Care, University of Helsinki and Helsinki University Hospital, Helsinki, Finland. [48]Department of Psychology, University of Oslo, Oslo, Norway. [49]Division of Mental Health and Addiction, Oslo University Hospital, Oslo, Norway. [50]Department of Psychology, University of Crete, Rethymno, Greece. [51]Department of Psychiatry, Martin Luther University of Halle-Wittenberg, Halle, Germany. [52]Department of Psychiatry, Yale University School of Medicine, New Haven, CT, USA. [53]Laboratory of NeuroGenetics, Department of Psychology and Neuroscience, Duke University, Durham, NC, USA. [54]Department of Psychiatry, National and Kapodistrian University of Athens Medical School, Eginition Hospital, Athens, Greece. [55]University Mental Health Research Institute, Athens, Greece. [56]Neurobiology Research Institute, Theodor-Theohari Cozzika Foundation, Athens, Greece. [57]Institute for Behavioral Genetics, University of Colorado, Boulder, CO, USA. [58]Psychiatry and Behavioral Sciences, Division of Medical Psychology and Department of Neurology, Duke University Medical Center, Durham, NC, USA. [59]Department of Psychology and Logopedics, Faculty of Medicine, University of Helsinki, Helsinki, Finland. [60]Helsinki Collegium for Advanced Studies, University of Helsinki, Helsinki, Finland. [61]Department of Psychiatry and Biobehavioral Sciences and Department of Molecular and Medical Pharmacology, University of California, Los Angeles, Los Angeles, CA, USA. [62]Department of Biological and Medical Psychology, University of Bergen, Bergen, Norway. [63]K.G. Jebsen Center for Research on Neuropsychiatric Disorders, University of Bergen, Bergen, Norway. [64]Division of Brain Sciences, Department of Medicine, Imperial College London, London, UK. [65]Centre for Integrated Genomic Medical Research, Institute of Population Health, University of Manchester, Manchester, UK. [66]Institute for Molecular Medicine Finland (FIMM), University of Helsinki, Helsinki, Finland. [67]Wellcome Trust Sanger Institute, Wellcome Trust Genome Campus, Cambridge, UK. [68]Center for Human Genetic Research, Psychiatric and Neurodevelopmental Genetics Unit, Massachusetts General Hospital, Boston, MA, USA. [69]Centre for Epidemiology, Division of Population Health, Health Services Research, and Primary Care, University of Manchester, Manchester, UK. [70]Division of Neuroscience and Experimental Psychology/School of Biological Sciences, Faculty of Biology Medicine and Health, University of Manchester, Manchester Academic Health Science Centre, Salford Royal NHS Foundation Trust, Manchester, UK. [71]Department of Psychology, Stanford University, Palo Alto, CA, USA. [72]Institute of Behavioural Sciences, University of Helsinki, Helsinki, Finland. [73]Department of Genetics and Genomic Science and Institute for Multiscale Biology, Icahn School of Medicine at Mount Sinai, New York, NY, USA. [74]Robert and Beverly Lewis Center for Neuroimaging, University of Oregon, Eugene, OR, USA. [75]NORMENT, K.G. Jebsen Centre for Psychosis Research, Institute of Clinical Medicine, University of Oslo and Division of Mental Health and Addiction, Oslo University Hospital, Oslo, Norway. [76]Alzheimer Scotland Dementia Research Centre, University of Edinburgh, Edinburgh, UK. [77]Lieber Institute for Brain Development, Johns Hopkins University Medical Campus, Baltimore, MD, USA. [78]Department of Epidemiology, Erasmus University Medical Center, Rotterdam, The Netherlands. [79]Campbell Family Mental Health Institute, Centre for Addiction and Mental Health, University of Toronto, Toronto, Ontario, Canada. [80]Department of Biostatistics, University of Michigan, Ann Arbor, MI, USA. [81]Center for Statistical Genetics, University of Michigan, Ann Arbor, MI, USA. [82]Institute of Clinical Medicine, University of Oslo, Oslo, Norway. [83]Department of Human and Molecular Genetics, Virginia Commonwealth University, Richmond, VA, USA. [84]College Behavioral and Emotional Health Institute, Virginia Commonwealth University, Richmond, VA, USA. [85]Analytic and Translational Genetics Unit, Massachusetts General Hospital, Boston, MA, USA. [86]Stanley Center for Psychiatric Research, Broad Institute of MIT and Harvard, Cambridge, MA, USA. [87]Department of Genetics, University of North Carolina, Chapel Hill, NC, USA. [88]Centre for Advanced Imaging, University of Queensland, Brisbane, Queensland, Australia. [89]These authors contributed equally: Jeanne E. Savage, Philip R. Jansen. *e-mail: d.posthuma@vu.nl

© 2018 Nature America Inc., part of Springer Nature. All rights reserved.

Supplementary Note for

Genome-wide association meta-analysis in 269,867 individuals identifies new genetic and functional links to intelligence

Jeanne E Savage[1#], Philip R Jansen[1,2#], Sven Stringer[1], Kyoko Watanabe[1], Julien Bryois[3], Christiaan A de Leeuw[1], Mats Nagel[4], Swapnil Awasthi[5], Peter B Barr[6], Jonathan R I Coleman[7,8], Katrina L Grasby[9], Anke R Hammerschlag[1], Jakob A Kaminski[5,10], Robert Karlsson[3], Eva Krapohl[7], Max Lam[11], Marianne Nygaard[12,13], Chandra A Reynolds[14], Joey W Trampush[15,16], Hannah Young[17], Delilah Zabaneh[7], Sara Hägg[3], Narelle K Hansell[18], Ida K Karlsson[3], Sten Linnarsson[19], Grant W Montgomery[9,20], Ana B Muñoz-Manchado[19], Erin B Quinlan[21], Gunter Schumann[21], Nathan G Skene[19,22], Bradley T Webb[23,24], Tonya White[2], Dan E Arking[25], Dimitrios Avramopoulos[25,26], Robert M Bilder[27], Panos Bitsios[28], Katherine E Burdick[29,30,31], Tyrone D Cannon[32], Ornit Chiba-Falek[33], Andrea Christoforou[34], Elizabeth T Cirulli[35], Eliza Congdon[27], Aiden Corvin[36], Gail Davies[37,38], Ian J Deary[37,38], Pamela DeRosse[39,40,41], Dwight Dickinson[42], Srdjan Djurovic[43,44], Gary Donohoe[45], Emily Drabant Conley[46], Johan G Eriksson[47,48], Thomas Espeseth[49,50], Nelson A Freimer[27], Stella Giakoumaki[51], Ina Giegling[52], Michael Gill[36], David C Glahn[53,] Ahmad R Hariri[54], Alex Hatzimanolis[55,56,57], Matthew C Keller[58], Emma Knowles[53], Deborah Koltai[59], Bettina Konte[52], Jari Lahti[60,61], Stephanie Le Hellard[34,44], Todd Lencz[39,40,41], David C Liewald[38], Edythe London[27,62], Astri J Lundervold[63,64], Anil K Malhotra[39,40,41], Ingrid Melle[44,50], Derek Morris[45], Anna C Need[65], William Ollier[66], Aarno Palotie[67,68,69], Antony Payton[70], Neil Pendleton[71], Russell A Poldrack[72], Katri Räikkönen[73], Ivar Reinvang[49], Panos Roussos[29,30,74], Dan Rujescu[52], Fred W Sabb[75], Matthew A Scult[54], Olav B Smeland[76], Nikolaos Smyrnis[55,56], John M Starr[37,77], Vidar M Steen[34,44], Nikos C Stefanis[55,56,57], Richard E Straub[78], Kjetil Sundet[49,50], Henning Tiemeier[2,79], Aristotle N Voineskos[80], Daniel R Weinberger[78], Elisabeth Widen[67], Jin Yu[39], Goncalo Abecasis[81,82], Ole A Andreassen[50,76,83], Gerome Breen[7,8], Lene Christiansen[12,13], Birgit Debrabant[13], Danielle M Dick[6,84,85], Andreas Heinz[5], Jens Hjerling-Leffler[19], M Arfan Ikram[79], Kenneth S Kendler[23,24,84], Nicholas G Martin[9], Sarah E Medland[9], Nancy L Pedersen[3], Robert Plomin[7], Tinca JC Polderman[1], Stephan Ripke[5,86,87], Sophie van der Sluis[4], Patrick F Sullivan[3,88], Scott I Vrieze[17], Margaret J Wright[18,89], Danielle Posthuma[1,4]*

Affiliations:

1. Department of Complex Trait Genetics, Center for Neurogenomics and Cognitive Research, Amsterdam Neuroscience, Vrije Universiteit Amsterdam, Amsterdam, The Netherlands
2. Department of Child and Adolescent Psychiatry, Erasmus Medical Center, Rotterdam, The Netherlands
3. Department of Medical Epidemiology and Biostatistics, Karolinska Institutet, Stockholm, Sweden
4. Department of Clinical Genetics, section Complex Trait Genetics, Neuroscience Campus Amsterdam, VU Medical Center, Amsterdam, the Netherlands
5. Department of Psychiatry and Psychotherapy, Charité Universitätsmedizin Berlin, Campus Mitte, Berlin, Germany
6. Department of Psychology, Virginia Commonwealth University, Richmond, VA, USA
7. Social, Genetic and Developmental Psychiatry Centre, Institute of Psychiatry, Psychology and Neuroscience, King's College London, London, UK
8. NIHR Biomedical Research Centre for Mental Health, South London and Maudsley NHS Trust, London, UK
9. QIMR Berghofer Medical Research Institute, Herston, Brisbane, Australia
10. Berlin Institute of Health (BIH), 10178 Berlin, Germany

11. Institute of Mental Health, Singapore
12. The Danish Twin Registry and the Danish Aging Research Center, Department of Public Health, University of Southern Denmark, Odense, Denmark
13. Epidemiology, Biostatistics and Biodemography, Department of Public Health, University of Southern Denmark, Odense, Denmark
14. Department of Psychology, University of California Riverside, Riverside, California, USA
15. BrainWorkup, LLC, Los Angeles, California, USA
16. Department of Psychiatry and the Behavioral Sciences, Keck School of Medicine, University of Southern California, Los Angeles, California, USA
17. Department of Psychology, University of Minnesota, Saint Paul, Minnesota, USA
18. Queensland Brain Institute, University of Queensland, Brisbane, Australia
19. Laboratory of Molecular Neurobiology, Department of Medical Biochemistry and Biophysics, Karolinska Institutet, Stockholm, Sweden
20. Institute for Molecular Bioscience, University of Queensland, Brisbane, Australia
21. Centre for Population Neuroscience and Precision Medicine (PONS), Institute of Psychiatry, Psychology and Neuroscience, MRC-SGDP Centre, King's College London, London, UK
22. UCL Institute of Neurology, Queen Square, London, UK
23. Virginia Institute for Psychiatric and Behavioral Genetics, Virginia Commonwealth University, Richmond, Virginia, USA
24. Department of Psychiatry, Virginia Commonwealth University, Richmond, Virginia, USA
25. McKusick-Nathans Institute of Genetic Medicine, Johns Hopkins University School of Medicine, Baltimore, Maryland, USA
26. Department of Psychiatry, Johns Hopkins University School of Medicine, Baltimore, Maryland, USA
27. UCLA Semel Institute for Neuroscience and Human Behavior, Los Angeles, California, USA
28. Department of Psychiatry and Behavioral Sciences, Faculty of Medicine, University of Crete, Heraklion, Crete, Greece
29. Department of Psychiatry, Icahn School of Medicine at Mount Sinai, New York, New York, USA
30. Mental Illness Research, Education, and Clinical Center (VISN 2), James J. Peters VA Medical Center, Bronx, New York, USA
31. Department of Psychiatry - Brigham and Women's Hospital; Harvard Medical School; Boston MA
32. Department of Psychology, Yale University, New Haven, Connecticut, USA
33. Department of Neurology, Bryan Alzheimer's Disease Research Center, and Center for Genomic and Computational Biology, Duke University Medical Center, Durham, North Carolina, USA
34. Dr. Einar Martens Research Group for Biological Psychiatry, Center for Medical Genetics and Molecular Medicine, Haukeland University Hospital, Bergen, Norway
35. Human Longevity Inc, Durham, North Carolina, USA
36. Neuropsychiatric Genetics Research Group, Department of Psychiatry and Trinity College Institute of Neuroscience, Trinity College Dublin, Dublin, Ireland
37. Centre for Cognitive Ageing and Cognitive Epidemiology, University of Edinburgh, Edinburgh, UK
38. Department of Psychology, University of Edinburgh, Edinburgh, UK
39. Division of Psychiatry Research, The Zucker Hillside Hospital, Glen Oaks, New York, USA
40. Department of Psychiatry, Hofstra Northwell School of Medicine, Hempstead, New York, USA
41. Center for Psychiatric Neuroscience, Feinstein Institute for Medical Research, Manhasset, New York, USA
42. Clinical and Translational Neuroscience Branch, Intramural Research Program, National Institute of Mental Health, National Institute of Health, Bethesda, Maryland, USA
43. Department of Medical Genetics, Oslo University Hospital, University of Bergen, Oslo, Norway
44. NORMENT, K.G. Jebsen Centre for Psychosis Research, University of Bergen, Bergen, Norway
45. Neuroimaging, Cognition & Genomics (NICOG) Centre, School of Psychology and Discipline of Biochemistry, National University of Ireland, Galway, Ireland
46. 23andMe, Inc., Mountain View, California, USA
47. Department of General Practice and Primary Health Care, University of Helsinki, Helsinki, Finland
48. Helsinki University Hospital, Helsinki, Finland
49. Department of Psychology, University of Oslo, Oslo, Norway

50. Division of Mental Health and Addiction, Oslo University Hospital, Oslo, Norway
51. Department of Psychology, University of Crete, Greece
52. Department of Psychiatry, Martin Luther University of Halle-Wittenberg, Halle, Germany
53. Department of Psychiatry, Yale University School of Medicine, New Haven, Connecticut, USA
54. Laboratory of NeuroGenetics, Department of Psychology & Neuroscience, Duke University, Durham, North Carolina, USA
55. Department of Psychiatry, National and Kapodistrian University of Athens Medical School, Eginition Hospital, Athens, Greece
56. University Mental Health Research Institute, Athens, Greece
57. Neurobiology Research Institute, Theodor-Theohari Cozzika Foundation, Athens, Greece
58. Institute for Behavioral Genetics, University of Colorado, Boulder, Colorado, USA
59. Psychiatry and Behavioral Sciences, Division of Medical Psychology, and Department of Neurology, Duke University Medical Center, Durham, North Carolina, USA
60. Department of Psychology and Logopedics, Faculty of Medicine, University of Helsinki, Helsinki, Finland
61. Helsinki Collegium for Advanced Studies, University of Helsinki, Helsinki, Finland
62. Department of Psychiatry and Biobehavioral Sciences and Department of Molecular and Medical Pharmacology, University of California Los Angeles, Los Angeles, Carolina, USA
63. Department of Biological and Medical Psychology, University of Bergen, Norway
64. K.G. Jebsen Center for Research on Neuropsychiatric Disorders, University of Bergen, Norway
65. Division of Brain Sciences, Department of Medicine, Imperial College, London, UK
66. Centre for Integrated Genomic Medical Research, Institute of Population Health, University of Manchester, Manchester, UK
67. Institute for Molecular Medicine Finland (FIMM), University of Helsinki, Finland
68. Wellcome Trust Sanger Institute, Wellcome Trust Genome Campus, Cambridge, UK
69. Massachusetts General Hospital, Center for Human Genetic Research, Psychiatric and Neurodevelopmental Genetics Unit, Boston, Massachusetts, USA
70. Centre for Epidemiology, Division of Population Health, Health Services Research & Primary Care, The University of Manchester, Manchester, UK
71. Division of Neuroscience and Experimental Psychology/ School of Biological Sciences, Faculty of Biology Medicine and Health, University of Manchester, Manchester Academic Health Science Centre, Salford Royal NHS Foundation Trust, Manchester, UK
72. Department of Psychology, Stanford University, Palo Alto, California, USA
73. Institute of Behavioural Sciences, University of Helsinki, Helsinki, Finland
74. Department of Genetics and Genomic Science and Institute for Multiscale Biology, Icahn School of Medicine at Mount Sinai, New York, New York, USA
75. Robert and Beverly Lewis Center for Neuroimaging, University of Oregon, Eugene, Oregon, USA
76. NORMENT, K.G. Jebsen Centre for Psychosis Research, Institute of Clinical Medicine, University of Oslo and Division of Mental Health and Addiction, Oslo University Hospital, Oslo, Norway
77. Alzheimer Scotland Dementia Research Centre, University of Edinburgh, Edinburgh, UK
78. Lieber Institute for Brain Development, Johns Hopkins University Medical Campus, Baltimore, Maryland, USA
79. Department of Epidemiology, Erasmus University Medical Center, Rotterdam, The Netherlands
80. Campbell Family Mental Health Institute, Centre for Addiction and Mental Health, University of Toronto, Toronto, Canada
81. Department of Biostatistics, University of Michigan, Ann Arbor, Michigan, USA
82. Center for Statistical Genetics, University of Michigan, Ann Arbor, Michigan, USA
83. Institute of Clinical Medicine, University of Oslo, Oslo, Norway
84. Department of Human and Molecular Genetics, Virginia Commonwealth University, Richmond, Virginia, USA
85. College Behavioral and Emotional Health Institute, Virginia Commonwealth University, Richmond, Virginia, USA
86. Analytic and Translational Genetics Unit, Massachusetts General Hospital, Boston, Massachusetts, USA
87. Stanley Center for Psychiatric Research, Broad Institute of MIT and Harvard, Cambridge, Massachusetts, USA
88. Department of Genetics, University of North Carolina, Chapel Hill, North Carolina, USA
89. Centre for Advanced Imaging, University of Queensland, Brisbane, Australia

# These authors contributed equally

*Correspondence to: Danielle Posthuma: Department of Complex Trait Genetics, Vrije Universiteit Amsterdam, De Boelelaan 1085, 1081 HV, Amsterdam, The Netherlands. Phone: +31 20 598 2823, Fax: +31 20 5986926, d.posthuma@vu.nl

Table of Contents

Affiliations: ... **1**

1. Supplementary Information ... **6**

1.1 Study Cohort Descriptions and Methodology ... **6**

1.1.1 UK Biobank (UKB) ... 6

1.1.2 Cognitive Genomics Consortium (COGENT) ... 9

1.1.3 Rotterdam Study (RS) ... 10

1.1.4 Generation R Study (GENR) ... 11

1.1.5 Swedish Twin Registry (STR) ... 12

1.1.6 Spit for Science (S4S) ... 13

1.1.7 High IQ / Health and Retirement Study (HiQ/HRS) ... 14

1.1.8 Twin Early Development Study (TEDS) ... 15

1.1.9 Danish Twin Registry (DTR) ... 16

1.1.10 IMAGEN ... 18

1.1.11 Brisbane Longitudinal Twin Study (BLTS) ... 19

1.1.12 Netherlands Study of Cognition, Environment and Genes (NESCOG) ... 20

1.1.13 Genes for Good (GfG) ... 20

1.1.14 Swedish Twin Studies of Aging (STSA) ... 21

1.2 Operationalization of intelligence ... **22**

2. Supplementary Results ... **25**

2.1 Cohort-Specific Analyses ... **25**

2.1.1 GWAS results per cohort ... 25

2.1.2 Genetic correlations between cohorts ... 26

2.1.3 SNP heterogeneity between cohorts ... 27

2.2 Age Group Comparisons ... **28**

2.3 Meta-analysis Results ... **28**

2.3.1 Genomic risk loci ... 29

2.3.2 Proxy replication ... 32

2.3.3 Polygenic score validation ... 33

2.3.4 Stratified heritability ... 34

2.3.5 Description and functional annotation of genomic risk loci ... 35

2.3.6 Gene mapping of GWS SNPs in genomic risk loci ... 37

2.4 Gene-based (GWGAS) and Gene-set Association ... **40**

2.4.1 Genome-wide gene-based association (GWGAS) ... 40

2.4.2 Gene-set association ... 41

2.5 Associations Between Intelligence and Other Phenotypes ... **44**

2.5.1 Genetic correlations ... 44

2.5.2 Overlap of intelligence risk loci with risk loci implicated in other phenotypes ... 45

2.5.3 Mendelian randomization with correlated traits ... 45

3. Supplementary Figures ... **52**

4. List of Supplementary Tables (separate Excel file) ... **96**

5. Additional Acknowledgements ... **98**

6. References ... **111**

1. Supplementary Information

1.1 Study Cohort Descriptions and Methodology

We meta-analyzed genome-wide association studies of intelligence measures in 14 cohorts, which are described in detail below.

1.1.1 UK Biobank (UKB)

The UK Biobank Study (http://www.ukbiobank.ac.uk) is a large population-based cohort that includes over 500,000 participants[1]. The aim of the UK Biobank is to study health-related determinants and outcomes by large-scale data-collection within the general population. The study protocol was approved by the National Research Ethics Service Committee North West–Haydock (reference 11/NW/0382), and all procedures were performed in accordance with the ethical principles for medical research declared in the World Medical Association Declaration of Helsinki. Access to the UK Biobank data was obtained under the UK Biobank application number 16406. Participants were recruited by invitation letters that were sent out to approximately 9.2 million individuals between 37-72 years who were living within 25 miles distance from one of the 22 study assessment centers and were registered with the National Health Service (NHS). In total, 503,325 participants were subsequently recruited into the study[1]. Large-scale data collection was performed that included a wide array of phenotypic data, such as registry-based phenotypic information, extensive self-reported baseline data collected by questionnaire, brain imaging, and anthropometric assessments, in addition to genotype data ascertained from blood samples.

We analyzed imputed genotype data from the second release by UK Biobank (July 2017), which includes 92,693,895 genetic variants in 487,442 individuals[2]. Genotyping was divided over 106 batches using two custom Affymetrix genotyping platforms (UK BiLEVE Axiom array n~=50,000; UK Biobank Axiom array n~=450,000). Quality control of the genotype data was performed locally by UK Biobank (details available at http://www.biorxiv.org/content/early/2017/07/20/166298). Genotypes were imputed using a combination of two reference panels. The first panel consisted of a merged reference panel that included the UK10K haplotype panel and the 1000 Genomes reference panel. In addition, the genotype data was imputed using the Haplotype Reference Consortium[3] (HRC) reference panel. If variants were imputed in both panels, the HRC imputation was retained. Following recommendations from UK Biobank, we excluded variants imputed based on the combined UK10K/1000 genomes panel.

For the current GWAS, we only included individuals of European ancestry, defined by projecting ancestry principal components from the 1000 Genomes reference populations[4] onto the called genotypes available in UK Biobank, and grouping individuals into their closest ancestral population identified by the minimum Mahalanobis distance from the projected principal component scores[5]. We excluded subjects with a Mahalanobis distance >6 SD from their empirically assigned population. Additional filtering of individuals was based on UKB-provided information on genomic relatedness (subjects with most inferred relatives, 3rd degree or closer, were removed until no related subjects were present), discordant sex, sex aneuploidy, missing phenotype or covariate data, and withdrawn consent.

Imputed variants were converted to hard calls at a certainty threshold of 0.9, filtering by an imputation INFO threshold of <0.9 and excluding multi-allelic SNPs, indels, SNPs without a unique rsID, and SNPs with minor allele frequency (MAF) <0.0001, resulting in a total of 10,847,151 remaining SNPs for analysis. To correct for population stratification, we computed European-specific principal components based on a set of 145,432 independent (r^2<0.1) autosomal SNPs with MAF >0.01 and INFO=1 using FlashPCA2[6].

Genome-wide analysis was performed on two fluid intelligence scores available in UK Biobank, that were collected by questionnaire either on a touch-screen device at the assessment center ('touchscreen', field 20016) or by an online questionnaire from home ('web-based', field 20191). The measures indicate the number of correct answers out of 13 questions of verbal and mathematical fluid intelligence. We used the test score that was collected during the first research visit (2006-2010), or during subsequent assessments in case data was missing at the first visit (N=19,831). Test results were available for N=185,317 (touch-screen; ts) and N=123,665 (web-based; wb) individuals. In case individuals had completed both the touch-screen and the web-based test (N=57,450), only the touchscreen test scores were used. In total, 251,532 individuals completed either the web-based (UKB-wb) or touchscreen (UKB-ts) version of the fluid intelligence score and 195,653 European ancestry individuals (142,077 ts/53,576 wb) with available genotype and phenotype data after quality control were included in the analysis (**Supplementary Table 1**).

GWAS was conducted separately for the two phenotypes in PLINK v1.9[7] using an additive linear regression model and controlling for covariates of age, sex, twenty European-based ancestry

principal components, genotyping array, socioeconomic status assessed by the Townsend deprivation index[8], and, for UKB-ts, assessment center where the measure was collected.

1.1.2 Cognitive Genomics Consortium (COGENT)

The Cognitive Genomics Consortium (COGENT) data comes from a large consortium that meta-analyzed GWAS results on general cognitive functioning from 24 independent studies (ACPRC, ADNI, ASPIS, CAMH, CHS, CNP, DCC, DNS, DUBLIN, FHS, GCAP, GENADA, HBCS, IBG, LBC1936, LOAD, LOGOS, MCTFR, MUNICH NCNG, PNC, TOP, ZHH). Full descriptions of these cohorts and the analytic methods are provided in Trampush et al[9], from which the reported GWAS summary stats were obtained. The samples were primarily adults (age range 8-96, *M*=46) and all of European ancestry, from the United States, United Kingdom, and Europe. All studies were approved by their relevant institutional ethics review board. Participants were excluded based on presence of dementia or other neuropsychiatric disorder, where relevant to the study sample. To be included in the COGENT meta-analysis, participants had to have at least one neuropsychological measure available across at least three domains of cognitive performance (e.g. working memory, verbal memory, processing speed, executive function) or a validated *g* factor measure such as a full-scale IQ test. Common neuropsychological measures used by the studies included the digit symbol, digit span, verbal memory for words/stories, semantic/phonemic fluency, vocabulary, and trail-making tests, as described in Trampush et al[9]. GWAS meta-analysis was performed on a score derived from the first unrotated principal component of the neurocognitive measures. This principal component explained on average 42% (SD=11%) of the variance in overall cognitive test performance. Participants in the

individual samples were genotyped on either Affymetrix or Illumina SNP arrays. After quality control steps including SNP filtering on MAF <0.01, call rate <98%, Hardy-Weinberg Equilibrium (HWE) $P<10^{-6}$; and individual filtering on call rate <98%, ancestry outliers, sex mismatch, and cryptic relatedness, samples were imputed to the HRC reference panel. Association analyses were conducted in each cohort using either PLINK v1.9 or BOLT-LMM[10] (for studies whose design included related individuals), using a linear allelic association test with imputed dosages and adjusting for age, sex, and ancestry principal components. Cohort results were meta-analyzed in METAL[11] and filtered for minimum imputation INFO score <0.6, MAF <0.1, and minimum N <10,000.

1.1.3 Rotterdam Study (RS)

The Rotterdam Study is a population-based cohort in the area of Rotterdam, the Netherlands including individuals of 45 years and older[12]. The current study included all participants for whom genotypic data was available and who had undergone cognitive testing at the study center from 2002 onwards. The Rotterdam Study protocol has been approved by the medical ethics committee according to the Population Study Act Rotterdam Study, executed by the Ministry of Health, Welfare and Sports of the Netherlands, and written informed consent was obtained from all participants. Participants underwent extensive cognitive testing with a neuropsychological battery comprising of the letter-digit substitution task (number of correct digits in one minute), the verbal fluency test (animal categories), the Stroop test (error-adjusted time in seconds for Stroop reading and interference tasks), and a 15-word learning test (delayed recall). Principal component analysis was performed on the test results to estimate a

g-factor, which explained 66.0% of the variation in cognitive test scores. Genotyping was performed on either Illumina 550, Illumina 550 duo or Illumina 610 quad SNP arrays. Variants were filtered on call rate <95%, MAF<0.01, and HWE $P<10^{-6}$. Individuals were filtered based on individual call rate <95%, gender mismatch, outlying heterozygosity, non-European ancestry and relatedness (pairwise IBD >0.185). Genotypes were imputed to the HRC reference panel. Genome-wide analysis of the g-factor was performed in PLINK v1.9 in 6,182 individuals, while correcting for age, sex, genotype array and ancestry principal components.

1.1.4 Generation R Study (GENR)

The Generation R Study is a large birth cohort in the area of Rotterdam, the Netherlands, that investigated approximately 10,000 children born between 2002 and 2006[13]. The study was approved by The Medical Ethical Committee of the Erasmus Medical Center, and parents provided informed consent for their enrolled children. Cognitive testing was done when children were around the age of 6 using the two subtests of the Snijders-Oomen Non-Verbal Intelligence test revised (SON-R)[14]. The subsets 'mosaics' and 'categories' were used, which assess spatial visualization abilities and abstract reasoning abilities, respectively. The correlation between the total score derived from these two subsets and IQ scores of the complete test was 0.86 in a subsample of children (N=626). A nonverbal IQ score was derived from the cognitive test scores using normal values tailored to exact age. DNA was extracted either from cord blood at birth or from venipuncture during a later visit to the research center. Genotyping was performed on Illumina 550K or Illumina 610K SNP arrays. An extensive description of the calling procedures and subsequent quality control have been described previously[15]. SNP-level

filtering included genotype call rate <95%, MAF <0.01, and HWE $P<10^{-6}$. Individuals were filtered on based on individual call rate <95%, outlying heterozygosity, non-European ethnicity, missing phenotype, and relatedness (pairwise IBD >0.185). Genotype data were subsequently imputed to the HRC reference panel. Genome-wide analysis of the derived IQ score in 1,929 individuals was performed using linear regression in PLINK v1.9, while correcting for age, gender, genotype array and ancestry principal components.

1.1.5 Swedish Twin Registry (STR)

The Swedish Twin Registry (STR) is a large epidemiological resource established in 1950s to study smoking and alcohol consumption and disease risk[16]. In a subset of this sample (n = 3,125), records from the Military Archives of Sweden were matched to participant data to obtain a measure of intelligence that was assessed during military conscription at age 18, as described in Rietveld et al[17]. Participants provided written informed consent and the study was approved by the local ethics review board. The assessment included 4 subtests of logical, verbal, spatial, and technical ability, from which the first principal component was extracted for analysis. Participants were genotyped on the Illumina HumanOmniExpress-12v1_A, and genotypes were imputed to the HapMap2 CEU reference population using IMPUTE2[18] after excluding variants with call rate <95%, MAF <0.01, and HWE $P<10^{-6}$, and individuals with phenotypic/genotypic sex discordance, outlying heterozygosity (>5 S.D.), relatedness, or non-European ancestry. Genetic analysis with a linear model was conducted using Merlin-offline[19] to account for the relatedness of the twin sample, using an age/sex standardized cognitive

performance score and ancestry principal components as covariates. GWAS summary statistics for this analysis were obtained from previous reports[17,20].

1.1.6 Spit for Science (S4S)

The Spit for Science study aims to study genetic, environmental, and developmental influences on substance use and emotional health in a representative sample of college students at a large, urban, public university in the United States[21]. All participants provided written informed consent and the study was approved by the local institutional review board. The study enrolled four cohorts of incoming freshman students to participate in an online self-report survey. Participant data was also matched to university records, from which a measure of intelligence was obtained from scores on the SAT, a standardized test that is widely used for college admission and includes two primary domains of fluid intelligence: critical reading/writing and mathematics. Scores on the SAT are highly correlated with other intelligence tests and provide a good index of *g*[22]. The sample has little variation in age at enrollment (*M*=18.5), although exact age at SAT assessment was not available. However, the SAT is a standard prerequisite for college admissions in the United States and is nearly always taken in the year prior to college enrollment, when participants would have been aged 17-18. Participants were required to be at least age 18 to enroll in the study, and 98% were between 18.0-19.5 at the initial freshman fall assessment.

DNA was isolated from saliva and subsequent genotyping was performed on the Axiom Biobank 650k V2 array. Following removal of poor quality SNPs with call rates <95%, HWE $P<10^{-6}$, and samples with call rates <98%, sex discordance, outlying heterozygosity, or relatedness,

genotypes were imputed to the 1000 Genomes phase 1 v.3 reference panel. Individuals from this multi-ethnic sample were each assigned to continental super-population groups best reflecting their genetic ancestry by projecting ancestry principal components from the 1000 Genomes reference populations onto the S4S samples and identifying the most closely-related population based on Mahalanobis distances (as described in section 1.1.1; see also Webb et al.[5]). Genetic association analyses were then conducted separately within each of five ancestral super-population groups to avoid spurious association results due to population stratification. Included here are results from the European subset (N=2,818). Analyses were conducted in SNPTESTv2[23] using an additive linear model with sex and within-ancestry principal components (selected via step-wise linear regression association with the phenotype) as covariates.

1.1.7 High IQ / Health and Retirement Study (HiQ/HRS)

This study used an extreme sampling design to compare individuals with high intelligence with unascertained population controls[24]. The HiQ sample included individuals with exceptionally high IQ (top 0.03% of the IQ distribution) that were identified by the Duke University Talent Identification Program (TIP), a program that identifies high-achieving youth who take the SAT at age 12 instead of the usual age 18. The project received ethical approval from the King's College London Research Ethics Committee (PNM/11/12–51) and the European Research Council Executive Agency ((2012)56321) and informed consent was obtained from all subjects. DNA was derived from buccal swabs and subsequent genotyping was done on the Illumina Omni Express genotyping array. In total, 1,238 high-IQ individuals of European descent were genotyped and passed quality control.

As a population comparison group, individuals were obtained from the publically available University of Michigan Health and Retirement Study (HRS) sample, a representative population-based sample of individuals in the United States aged 50 years and older. Given that these participants come from an epidemiological birth cohort unselected for cognitive or other health-related outcomes, it is presumed that intelligence follows a typical normal distribution in this sample. Genotype data was based on saliva, and genotyping was performed on Illumina Human Omni-2.5 Quad Beadchip. These data were obtained through dbGaP (accession: phs000428.v2.p2, https://www.ncbi.nlm.nih.gov/projects/gap/cgi-bin/study.cgi?study_id=phs000428.v2.p2). After quality control and ancestry-matching to the HiQ participants, genotypes were available for 8,172 European ancestry individuals.

Quality control steps, performed separately in the two samples, included filtering SNPs with call rate <98%, HWE $P<10^{-6}$, or MAF <0.005 and samples with call rate <98%, outlying heterozygosity (>4 SD), relatedness, or outlying ancestry. Samples from both studies were imputed to the HRC reference panel. High IQ individuals were used as cases and population comparisons as controls in a logistic regression model in SNPTESTv2 using imputed dosages of SNPs with MAF >0.01 and INFO >0.9, controlling for sex and ancestry principal components. Therefore, unlike the rest of the cohorts, the phenotype for this cohort was status as a high-IQ or unselected individual, rather than a measured intelligence score.

1.1.8 Twin Early Development Study (TEDS)

The Twins Early Development Study (TEDS)[25] is a longitudinal cohort study investigating over 11,000 twin pairs from England and Wales between 1994 and 1996. The study was approved by

the ethics committee of the Institute of Psychiatry (05/Q0706/228), and consent was obtained from parents of the participants. Cognitive testing was performed at the age of 12, using the WISC-III-PI Multiple Choice Information (General Knowledge) and Vocabulary Multiple Choice subtests[26], and two nonverbal reasoning tests, the WISC-III-UK Picture Completion[26] and Raven's Standard and Advanced Progressive Matrices[27,28], which were all administered online[29]. g-scores were derived as the arithmetic mean of the four standardized test scores. DNA was obtained from saliva and buccal cheek swabs. Genotyping was performed on either an Affymetrix GeneChip 6.0 (N=2,241) or Illumina HumanOmniExpressExome SNP array (N=1,173), which were imputed and analyzed separately (referred to as TEDS1 and TEDS2, respectively, throughout the manuscript). Individuals were filtered from subsequent analysis based on call rate <0.99%, non-European ancestry, outlying heterozygosity, poor array signal intensity, and relatedness. SNPs were filtered on MAF <0.05, and genotype call rate of <99%, and HWE $P<10^{-5}$. Genotype data were imputed to the HRC reference panel. GWAS was conducted with a linear regression model using hard-called imputed dosage data in PLINK v1.9, correcting for sex, age at assessment and ancestry principal components.

1.1.9 Danish Twin Registry (DTR)

The Danish Twin Registry sample (DTR) included 990 participants that were collected as part of two studies: the study of Middle-Aged Danish Twins (MADT, n=737) and the Longitudinal Study of Aging Danish Twins (LSADT, n=253). Written informed consent was obtained from all participants. Collection and use of biological material and survey information were approved by the Regional Scientific Ethical Committees for Southern Denmark, and the study was approved

by the Danish Data Protection Agency. MADT was initiated in 1998 and includes 4,314 twins randomly chosen from the birth years 1931-1952[30]. Surviving participants were revisited from 2008 to 2011[31], where blood samples and cognitive test data were collected. LSADT was initiated in 1995 and includes twins aged 70 years and older. Follow-up assessments were conducted every second year through 2005[32]. The individuals included in the present study all participated in the 1997 assessment, where blood samples and cognitive data were collected from same sex twin pairs. The phenotype in both MADT and LSADT was a composite cognitive score that included tests of verbal fluency, forward and backward digit span, and immediate and delayed recall[33]. In both studies, independent samples (excluding co-twins within a pair) were used for analysis.

Samples from MADT (N=737) were genotyped using the Illumina Infinium PsychArray (Illumina San Diego, CA, USA). Genotyping was conducted by the SNP&SEQ Technology Platform, Science for Life Laboratory, Uppsala, Sweden (http://snpseq.medsci.uu.se/genotyping/snp-services/). Pre-imputation quality control included filtering SNPs on genotype call rate <98%, HWE $P<10^{-6}$, and MAF=0, and individuals on sample call rate <99%, relatedness and gender mismatch. Pre-phasing and imputation to the 1000 Genomes phase I v.3 reference panel was performed using IMPUTE2 and a chunk-size of 1 MB. GWAS was conducted using hard-called imputed dosage data in PLINK v1.9 using age and sex as covariates in a linear regression model. Samples from LSADT (N=253) were genotyped on the Illumina Infinium PsychArray BeadChip and underwent quality control procedures including filtering SNPs on call rate of <98%, HWE $P<10^{-6}$, and filtering individuals based on individual call rate <99%, relatedness and gender mismatch, before imputation to the 1000 Genomes phase I v.3 reference panel. GWAS was conducted

using a linear regression model with hard-called imputed dosage data in PLINK v1.9 using age, sex, and ancestry principal components as covariates.

1.1.10 IMAGEN

The IMAGEN study is an international collaboration that studies mental health in adolescents from the UK, France, Ireland and Germany[34] and collects data from self-report questionnaires, behavioral assessment, interviews, brain imaging and genetic data. The study protocol was approved by the local ethics committees and all participants and their parents provided written informed consent/assent. The study conducted a neurocognitive test battery in subjects from three subsamples. Principal component analysis was performed to obtain a general factor of cognition based on WISC IV[26] subtests (matrix reasoning, block design, digit span backward and forward, similarities and vocabulary) and the CANTAB battery[35] (pattern recognition, rapid visual information processing and spatial working memory). The first principal component explained more than twice as much variance as compared to the following components, and was used as a general factor of IQ (g-factor). DNA was collected from blood obtained by venipuncture and genotyping was performed using Illumina Quad 610 chips. Data was collected from eight different study sites and genotyped in three batches, for which quality control, imputation, and analysis were performed separately. In each batch, SNPs were filtered based on call rate <0.98 and HWE $P<10^{-6}$ and individuals were filtered based on individual call rate <98%, outlying heterozygosity rate (<0.2), pairwise IBD >0.2, and non-European ancestry. Genotype imputation was performed using the pre-phasing/imputation stepwise approach implemented in IMPUTE2/SHAPEIT2[36] (chunk size of 3 Mb and default parameters) with the

1000 Genomes Project reference panel phase I v3. Post-imputation, SNPs were additionally filtered for INFO > 0.8 and missingness < .01. GWAS was conducted on each dataset using an additive linear model in PLINK with ancestry principal components as covariates and results were meta-analyzed across the three subsamples (N=1,343 individuals) using METAL with an inverse variance-weighted fixed effects scheme.

1.1.11 Brisbane Longitudinal Twin Study (BLTS)

The Brisbane Longitudinal Twin Study (BLTS) was initiated in 1992 at the Queensland Institute of Medical Research (QIMR)[37]. Twelve year old twins and their non-twin siblings were recruited from primary and secondary schools, and were followed over time, including cognitive testing at the age of 12 and 16. Cognitive phenotypes included IQ scores from the Multi-dimensional Aptitude Battery II (MAB)[38] at age 16 and the first factor score from the Verbal and Spatial Reasoning Test for Children (VSRT-C)[39] at age 12. Participants were genotyped using Illumina 610-Quadv1 or Illumina HumanCoreExome-12v1 chips using DNA extracted from blood samples in the majority of the cases. Data was screened for genotyping quality (GenCall < .7 from the 610k chip), SNP call rates (< 0.95% or < 0.99% for exome markers), HWE $P < 10^{-6}$, MAF < 0.01, individual call rate <0.95%, non-European ancestry, and pedigree, sex, and Mendelian errors. Data from the two genotype arrays were phased separately using SHAPEIT2 and imputed to the HRC reference panel. The GWAS was performed separately in children (QIMR-C, N=530) and adolescents (QIMR-A; N=2,598) using RareMetalWorker[40] software to account for relatedness, with sex, age, ancestry principal components and imputation run included as covariates in a linear model.

1.1.12 Netherlands Study of Cognition, Environment and Genes (NESCOG)

The Netherlands Study of Cognition, Environment and Genes (NESCOG), is a population-based study of adults that investigates genetic and environmental determinants of cognitive function[41]. For the current study a population-based family sample of NESCOG was used including 560 participants (330 females) that participated in a gene-environment interaction study on cognition. The study was approved by the institutional review board of Vrije Universiteit Amsterdam and participants provided informed consent. General cognition was measured using the full Wechsler Adult Intelligence Scale (WAIS)[42]. Genotype data was generated on the Illumina PsychChip array with HumanCore, Human Exome, and custom content. Pre-imputation SNP filtering included MAF <0.05, and genotype call rate of <99% and HWE $P<10^{-6}$. Individuals were filtered on outlying heterozygosity, genotype call rate <95% and non-European ancestry. Genotypes were subsequently imputed to the HRC reference panel. GWAS was conducted on the age- and sex-standardized scores using hard-called dosages in a linear model in PLINK v1.9 with ancestry principal components as covariates for 252 individuals with available phenotypic and genotypic data.

1.1.13 Genes for Good (GfG)

Genes for Good (GfG) is an online genetic study (http://genesforgood.org) hosted at the University of Michigan investigating health-related traits through the Facebook App Platform. The study is open to anyone in the US over the age of 18. The study methods were approved by the University of Michigan institutional review board and all participants provided written

informed consent. Participants were asked to fill out various health-related surveys including the ICAR 16 item Verbal Reasoning Test[43] (https://icar-project.com/), consisting of questions related to vocabulary, general knowledge, and logical deduction. The final phenotype is a factor extracted from a 2-parameter item response theory model on participant responses to the Verbal Reasoning Test. Genotyping on saliva samples was conducted on a standard Illumina HumanCoreExome BeadChip and Illumina HumanCoreExome BeadChip with custom content. Pre-imputation quality control included filtering SNPs with MAF <0.05, genotype call rate <99%, ambiguous SNPs with MAF >0.4, HWE $P<10^{-3}$, and individuals with sex discordance, individual call rate <99%, and non-European ancestry based on principal component analysis. Genotypes were imputed to the HRC reference panel. Genome-wide analysis was run on 5,084 individuals using a linear regression model in PLINK v1.9, and results were corrected for age, sex and ancestry principal components.

1.1.14 Swedish Twin Studies of Aging (STSA)

The Swedish Twin Studies of Aging include three studies grouped into two analytic cohorts, all samples of older adults with no history of stroke or dementia. All participants provided informed consent and the study was approved by the Regional Ethics Board in Stockholm and the Institutional Review Board at the University of Southern California. The Swedish Adoption/Twin Study of Aging (SATSA) and the "Sex differences in health and aging" (GENDER) studies include twins born, respectively, between 1886-1958 (SATSA)[44] and 1906-1925 (GENDER)[45]. The SATSA sample of like-sex twins and the GENDER sample of unlike sex twins were assessed at an in-person visit on a neurocognitive battery. The first principal component

was extracted from four ability tests available in 703 participants from the combined SATSA and GENDER samples, spanning verbal (Synonyms), spatial (Block Design), episodic memory (Thurstone Picture Memory), and processing speed (Digit Symbol) domains. This factor accounted for 60.79% of the variance in the four tests. DNA was extracted from blood samples and genotyped using the Illumina Infinium PsychArray. Data was cleaned for sample call rate <99%, SNP call rate <98%, HWE $P < 10^{-6}$, and non-European ancestry samples. Samples were phased and imputed to the 1000 Genomes phase 1 v.3 reference panel using SHAPEIT2+IMPUTE2. Association analysis was conducted using a linear model in PLINK v1.07[46] with age, sex, and ancestry principal components as covariates and cluster-robust standard errors to correct for twin pair dependency.

The Study of Dementia in Swedish Twins (HARMONY) includes twin pairs of like and unlike sex born before 1935 who completed a full clinical workup[47]. The first principal component was extracted from five intelligence tests in 448 participants, spanning verbal (WAIS Information subtest), executive functioning (Verbal Fluency), spatial (WAIS Block Design), episodic memory (Word List Learning-Delayed Recall), and processing speed (Digit Symbol) domains, and this factor accounted for 55.6% of the variance in task performance. Genotyping, quality control, and data analysis followed the same procedures as in the SATSA+GENDER cohort but were conducted separately.

1.2 Operationalization of intelligence

The various cohorts used different measures to assess intelligence (see **Supplementary Figure 2**). However, decades of research have firmly established that virtually all aspects of cognitive

functioning – verbal and mathematical ability, abstract reasoning, processing speed, executive functioning, spatial reasoning, memory, etc. – are captured primarily by a single underlying latent factor, labeled general intelligence or Spearman's *g*[48]. This is known as the positive manifold of cognitive ability or intelligence. Cognitive tests may comprise a single complex task that simultaneously taps into facets from each of these domains, or may be a constellation of multiple sub-tests each from a single domain. Regardless of the specific construction, correlations between performance on all such validated cognitive tests are high[49]. Further, the *g* factor underlying multiple different test batteries has been shown to be identical[50], indicating that different tests or different configurations of sub-tests index the same construct. While there are specific genetic and environmental influences on sub-domains of intelligence (for example, mathematics versus vocabulary), the covariance between these domains is best captured by a higher-order *g* factor, and this *g* factor accounts for most of the genetic influences on specific domains[51]. Phenotypes which extract the commonalities across multiple domains of cognitive functioning will thus be most informative for genetic association analyses. Meta-analysis of such phenotypes will improve power to detect variants that either have direct effects on individual variation in the *g* factor (i.e., a single system underlying global cognitive functioning), or variants which have pleiotropic effects across multiple domains of cognitive functioning that are indirectly captured by *g.* Unfortunately, as GWAS is simply a statistical association technique, it can only point to which genetic variants demonstrate a significant association and not what the mechanism for this association might be, requiring follow-up with functional testing and phenotypic refinement to determine how these variants and genes are linked to intelligence.

In the present study, all cohorts except HiQ/HRS used a quantitative measure of intelligence consisting of either a principal component extracted from multiple tests from different cognitive sub-domains (*g* factor), or a score from a composite measure with multiple sub-domain tests included by design (full-scale IQ scores from the WISC, WAIS, MAB, or SON-R; fluid intelligence measure summed over verbal and mathematical reasoning items; or the SAT college entrance exam which has both verbal and mathematical reasoning components). The GfG cohort is slightly more limited than the others in that its *g* factor was derived primarily from the verbal reasoning domain, and may capture somewhat less (but still a substantial proportion[49]) of the influence of the general intelligence factor. As the HiQ/HRS study sampled individuals from the extremely high and normal ranges of the Gaussian IQ distribution, it can be expected that the two sub-samples of this study differ on mean levels of the same *g* factor that underlies IQ scores. With the exception of severe intellectual disability caused by rare, usually Mendelian, mutations, high and low intelligence form a part of the same spectrum of variation as the normal range of intelligence[52-55], and so the “cases” and “controls” in this sample are expected to differ only quantitatively and not qualitatively in the genetic variants that influence intelligence.

We note that while differences in assessment methods across cohorts may reduce power to detect true associations in meta-analysis, it simultaneously reduces type I error by removing sources of measurement error that may be correlated between assessments when they are derived from the same method. Results therefore will more likely reflect genetic variants with robust associations to the common latent factor underlying general cognitive test performance across multiple methods and not those confounded by correlated measurement error.

2. Supplementary Results

2.1 Cohort-Specific Analyses

2.1.1 GWAS results per cohort

For each cohort, an individual GWAS was run after cohort-specific quality control was performed, as described in detail in **Supplementary Information 1.1**. Results were inspected for inflation possibly due to insufficiently corrected population stratification, by calculating the lambda inflation factor as well as the LD score intercept using LD score regression[56]. A lambda > 1 suggests an inflation of the genetic effects which can be due to both spurious and genuine effects. It is likely to increase with sample size and degree of polygenicity of the trait, as the distribution of effect sizes begins to differ substantially from a null distribution when more variants have true associations. An LD score intercept > 1 suggests that there is spurious association, and an intercept <1.10 is generally considered to suggest that the signal is mostly due to genuine association effects. For all cohorts except the HiQ/HRS cohort the LD intercept was < 1.04, and the intercept for the meta-analysis was 1.08 (compared to inflation of λ_{GC}=1.92), suggesting the genetic signal is mostly due to polygenicity and unlikely to be driven by population stratification (**Supplementary Table 4**).

For HiQ/HRS, the intercept was a bit higher at 1.14, although this is not outside the range reported for methodologically sound GWAS studies[56]. The HiQ/HRS was unusual among the meta-analysis cohorts in that it used a case-control design with samples recruited from two quite different populations, and it is possible that this influenced how well the LD reference population was matched and thus inflated the intercept. However, we note that there was no

evidence of inflation in a similar analysis with this sample using hard-called genotypes[24]. Further, when excluding this sample from the meta-analysis, 78% of genome-wide significant (GWS; $P<1\times10^{-8}$) SNPs from the full meta-analysis retain a GWS association and 100% retain at least suggestive significance ($P<5\times10^{-5}$), indicating that this sample is not contributing any additional spurious association signals. We see that this follows an expected pattern in which the increase in sample size boosts existing signal beyond a GWS threshold rather than identifying wholly distinct loci, as 94% of GWS SNPs remained significant and 100% were suggestive when excluding the smaller S4S sample (N=2,818), and 56% and 98% were GWS and suggestive, respectively, when excluding the larger set of non-adult samples (N=65,639) in the age group-specific meta-analyses.

2.1.2 Genetic correlations between cohorts

Genetic correlations between the GWAS results from all cohorts included in the meta-analysis, except 6 cohorts with N<800, were calculated with LD Score regression (**Supplementary Table 2)**. This method does not limit estimates of correlations between −1 and 1, to avoid bias in standard errors, but an r_g that is out of bounds (< −1 or > +1) usually means that one of the h^2 estimates was very close to zero[56,57]. Estimates of r_g ranged from −0.309 to 2.033, with lower or out of bound r_g's coming almost exclusively from cohorts with small sample size (N< 3,000). LD score regression estimates are highly variable and subject to chance fluctuation in small samples, thus a low or inconsistent r_g in these samples is not necessarily troubling on its own. No single cohort showed consistent low r_g's with all of the other cohorts, and thus we kept all cohorts in the meta-analysis. Capping the out-of-bound estimates at [-1,1], the average

correlation between cohorts was 0.67. The three largest cohorts (N >10,000: UKB-ts, UKB-wb, COGENT) were all very highly correlated, r_g >0.90, and each of the smaller cohorts showed a correlation of at least 0.56 with one of these three. Of the 21 pairwise correlations involving cohorts with N>3000, 20 were statistically significant (at P<.05) and all were positively correlated (r^2>0.56), indicating that substantial genetic overlap exists between cohorts so long as the sample size is large enough to obtain stable standard errors. Notably, despite having a different type of phenotype than the rest of the quantitative samples, the case/control HiQ/HRS sample was highly genetically correlated with the other cohorts (r_g >0.72 with UKB-ts, UKB-wb, and COGENT; mean r_g =0.71 with all cohorts).

2.1.3 SNP heterogeneity between cohorts

In addition to the primary fixed-effects meta-analysis, we conducted a random-effects meta-analysis to test for heterogeneity in the SNP association effects between cohorts. In the phase 1 meta-analysis (all cohorts except HiQ/HRS), no SNP had a heterogeneity test *P*-value below the GWS threshold, and 5.1% of all SNPs had a *P*-value less than a nominal threshold of 0.05. Among the 242 GWS lead SNPs reported here (see **Supplementary Results 2.3** below), none had a heterogeneity test *P*-value less than 0.05/242. In the phase 2 meta-analysis (phase 1 plus HiQ/HRS), a single SNP (rs7670286; meta-analysis *P*=0.59) had a heterogeneity test *P*-value below the GWS threshold, and 4.3% of all SNPs had a *P*-value less than a nominal threshold of 0.05. Among the 242 GWS lead SNPs reported here, none had a heterogeneity test *P*-value less than 0.05/242. Further, 15.5% of all meta-analysis SNPs, 29.3% of GWS SNPs, and 33.0% of lead SNPs were sign-concordant in more than 80% of available cohorts. Assuming that 10 cohorts

were available for meta-analysis (this number differs per SNP but is conservative when the number is larger), the expectation of observing >80% sign concordance by chance in a binomial distribution is 1.1%. Based on this expected proportion, the observed sign concordance rates for all SNPs, GWS SNPs, and lead SNPs were all significantly higher than that expected by chance (χ^2 test *P*s < 3×10^{-20}). Although a visual comparison of the individual cohort GWAS results (**Supplementary Figure 1**) shows clear differences in the associations detected in each cohort, these results are consistent with such effects being driven by differences in sample size/power rather than qualitative differences in the associated variants between cohorts. As noted in **Supplementary Results 2.1.1**, the consistency of significant/suggestive loci when excluding various subsets of the meta-analytic sample also corroborates to the homogeneity of genetic association effects between cohorts.

2.2 Age Group Comparisons

The vast majority of subjects included in the meta-analysis were adults, yet the relatively small child (N=9,814), young adult (N=6,033), and older adult (N=8,323) sub-samples were highly genetically correlated with the adult sample (**Supplementary Table 3**) suggesting that the same genetic effects are important across age groups. Although the point estimates of heritability were slightly larger in children (0.22), young adults (0.21), and older adults (0.23) versus adults (0.20), standard errors of the estimates in these subsets were overlapping, indicating that these could not be differentiated from equality.

2.3 Meta-analysis Results

2.3.1 Genomic risk loci

The meta-analysis resulted in 12,110 SNPs with a GWS association (P<5×10^{-8}), representing 524 *independent significant SNPs* in distinct LD regions (r^2 <0.6). These signals were indexed by 242 *lead SNPs* (r^2 <0.1) in 213 independent *genomic risk loci* after merging regions < 250 KB apart into a single locus (**Supplementary Table 5**). The borders of the genomic risk loci were defined by taking all independent GWS SNPs in a locus and then identifying all SNPs that were in LD with one of these SNPs in the reference panel, as detailed in the **Online Methods**. Annotated *candidate SNPs* (N=21,403) used for the follow-up analyses detailed below are all SNPs that are located in a risk locus and that are in LD with one of the independent GWS SNPs. Of the 12,110 GWS SNPs, 30 SNPs were not available in the UKB reference panel; 17 of these were within the positional boundaries of a risk locus and were included as such, and 13 were outside of bounds of defined risk loci (**Supplementary Table 6**).

Close visual examination of the regional association plots for each GWS locus (**Supplementary Figure 4**), which provide a zoomed in view of the SNPs contributing to the association signal in a linkage disequilibrium block, indicated 8 loci (#s 8, 40, 66, 82, 124, 134, 197, and 200) for which the patterns of association appeared suspicious (**Supplementary Figure 5**). Specifically, these regions had only one or two SNPs whose association was GWS, in contrast to a pattern of broad enrichment for SNPs in a local region that is seen in the majority of credibly associated loci due to the correlation of test statistics between SNPs in linkage disequilibrium. We investigated these loci further (**Supplementary Table 7**) and did not find obvious signs that would indicate genotyping errors or other statistical artefacts, such as poor imputation quality, inconsistent association statistics between cohorts (comparing our three largest cohorts, UKB-ts, UKB-wb,

COGENT, since test statistics can fluctuate substantially in small samples), major allele frequency differences from a reference panel, or patterns of regional LD that differed from a known reference panel. These SNPs generally also did not have low MAF, which might have explained the absence of LD proxies. These 8 loci appeared to be regions for which there were simply few other SNPs in strong LD with the lead SNP, and so there is a lack of information available to infer whether these represent credible association signals. None of the SNPs in these regions have been previously reported to have an association with intelligence or other cognitive phenotypes, and none were implicated in the proxy replication. We therefore have less confidence that these loci represent true genetic associations until they are replicated in future research, and so we report the total number of robustly associated genomic loci for intelligence as 205 (213-8). We do, however, include these eight more tentative loci in all tables for reference and future replication, with a notation of any results that stem from their inclusion.

These loci were mapped to genes *TTLL7* (locus 8, positional mapping), *ZNF385B* (locus 40, positional mapping), *ANAPC4* (locus 66, eQTL mapping), *CCDC149* (locus 66, positional and eQTL mapping), *SREK1IP1* (locus 82, positional mapping), *RIMS2* (locus 124, positional mapping), *DCC* (locus 197, positional mapping, although this gene also overlapped a second nearby locus, 198, that included many GWS SNPs), and *MAN2B1* (locus 200, eQTL mapping but also implicated via eQTL mapping from nearby locus 201). Of these genes, three genes (*ANAPC4, DCC, RIMS2*) also had a significant MAGMA gene-based *P*-value (P=1.67×10^{-7}, P=9.17×10^{-23}, and P=8.08×10^{-9} respectively, **Supplementary Table 15**) as they included multiple SNPs with *P*-values around the suggestive threshold of association, even though only a single SNP in these genes was GWS by

itself. *CCDC149* and *DCC* have been previously linked to intelligence in gene-based tests, although no SNPs had individually been associated[20,58]. None of these genes were found in the set of 92 genes implicated by all four mapping strategies (**Supplementary Table 16**). *RIMS2* was included in the *neurogenesis, regulation of nervous system development*, and *positive regulation of nervous system development* gene-sets that showed significant enrichment, and *DCC* was included in the *neurogenesis, neuron differentiation, central nervous system neuron differentiation,* and *regulation of nervous system development* gene-sets (**Supplementary Table 18**).

The 205 (i.e. 213-8) identified genomic risk loci were spread throughout the genome, with one or more risk loci found on every autosome. Of these, 190 were novel, which we defined as loci for which no SNPs within the positional bounds of the genomic risk locus (including SNPs that were not analyzed in the meta-analysis) had been previously linked to “intelligence”, “cognition”, or “cognitive ability” in the NHGRI-EBI catalog or in our manual lookup of recently published GWAS for these phenotypes (**Supplementary Table 24**). The genomic risk loci and their functional consequences are described further below in **Supplementary Results 2.3.5**.

Although chromosome X was included in the meta-analysis, we only had genotyped SNPs (N=14,847) on this chromosome available in the two UKB cohorts (N=195,653) (**Online Methods**). We did not find any GWS association on chromosome X with the currently available N for X, yet, given the difference in sample size and number of SNPs examined in comparison with the autosomes, their results are not directly comparable. We note that the current lack of GWS results does not necessarily imply there are no relevant genetic variants located on chromosome X. Current challenges with genotyping, imputation, and quality control of the X

chromosome often result in a disproportionately small number of high quality SNPs available for analysis, making its potential genetic contributions difficult to uncover[59].

2.3.2 Proxy replication

We tested for replication of the identified SNPs/loci using the proxy phenotype of educational attainment. Although replication would be more direct with the same measured intelligence phenotype, there are not, to our knowledge, other equally large independent cohorts with well-characterized assessments of intelligence. A sample size on par with that of the discovery sample is necessary to independently validate variants with very small effects, especially given the common occurrence of larger-than-expected associations being identified in the first discovery sample in a phenomenon known as “winner’s curse”.

Educational attainment is an easily and reliably measured phenotype and so has been collected in many hundreds of thousands of available samples. The genetic correlation between educational attainment and intelligence is among the largest observed between any pair of complex cognitive/psychiatric traits (rg > .70)[9,20], and there is evidence that intelligence directly impacts later educational attainment[60] and that (some of) the genetic effects on educational attainment are mediated through intelligence[61]. Educational attainment is therefore well-suited for use in validating genetic associations with intelligence and has been successfully applied as a proxy phenotype in several previous reports[17,20].

SNP-based proxy replication was conducted in a GWAS of educational attainment using a large, non-overlapping subset of the UKB sample (N=188,435; **Online Methods**), which included UKB subjects for which data on intelligence was not available and who were not genetically related

to any of the subjects for whom intelligence data was available. The GWAS results on educational attainment in UKB showed a high genetic correlation with previously published results of a GWAS meta-analysis of educational attainment[62] (LDscore regression r_g=0.93, SE=0.013), as well as a robust genetic correlation with the intelligence meta-analysis results r_g=0.73, SE=0.020). Out of the 12,110 GWS SNPs for intelligence, 12,093 were available for look-up, as were all of the 242 independent lead SNPs. We found that the effects of 11,280 out of 12,093 total SNPs (93%; exact binomial $P=5\times10^{-324}$) and the effects of 226 out of 242 independent lead SNPs (93%; exact binomial $P=1\times10^{-48}$) were sign concordant between educational attainment and intelligence (**Supplementary Table 8**). This approach resulted in 48 proxy-replicated loci (with $P<0.05/242$ for educational attainment). In 38 of these loci, the lead SNP for intelligence in the locus was significantly associated with educational attainment, while in the other 10 an LD proxy of the intelligence lead SNP was associated with educational attainment (**Supplementary Table 8)**.

2.3.3 Polygenic score validation

To evaluate the replicability and predictive utility of our SNP-based results, we estimated the variation in intelligence that could be explained by the current GWAS meta-analysis by calculating a polygenic score (PGS) in four independent cohorts using LDpred[63] and PRSice[64] (**Online Methods**). The four cohorts were each separately excluded from the meta-analysis, which was then re-run so the results could be used to calculate PGS in each independent hold-out sample.

There was good concordance between the two methods, with LDpred PGS explaining 2.4% - 4.7% ($P < 1\times10^{-21}$) of the variance in intelligence in each sample at the best prior of p=1.0/infinitesimal and PRSice PGS explaining 2.0% - 5.2% of the variance in each sample ($P < 2\times10^{-19}$) when filtering the included SNPs by meta-analysis *p* thresholds of < .11 (**Supplementary Table 9**), an increase in maximum explained variance of 0.4% compared to the previous largest GWAS meta-analysis[20]. Surprisingly, the proportional increases in number of associated loci found with this sample size relative to previous studies (205 versus 18 loci with sample N=269,867 versus 78,303) did not translate to a similarly large increase in variance explained by PGS (r^2=5.2% versus 4.8%).

2.3.4 Stratified heritability

Stratified heritability analyses showed enrichment of several functional genomic categories (**Supplementary Table 10**), including conserved regions of the genome (Enrichment of 16.44, proportion h^2=.428, P=2.01×10^{-12}), coding regions (Enrichment of 8.19, proportion h^2=.120, P=1.67×10^{-6}), H3K9ac histone regions (Enrichment of 2.44, proportion h^2=.307, P=6.25×10^{-5}), and the narrow H3K9ac peaks in these regions (Enrichment of 6.18, proportion h^2=.239, P=2.04×10^{-5}). H3K9ac is a histone mark that indicates an active promoter or enhancer region[65]. These annotations are not mutually exclusive, and in fact there is high evolutionary conservation for both protein-coding regions and regions of distal regulatory activity[66,67]. These results suggest that biological processes relevant for intelligence include those that are common across species, those that directly affect protein structure/function, as well as those that have indirect effects via regulatory mechanisms. These genetic effects come

disproportionately from variants that are very common, with greatest enrichment for SNPs with a minor allele frequency (MAF) between 0.4 and 0.5 (P=1.34×10^{-12}) in human populations (**Supplementary Figure 6**) but they are spread proportionally across the genome (**Supplementary Figure 7**), likely reflecting a high degree of polygenicity and the involvement of a large proportion of the genome in variation in intelligence. However, we note that our MAF filtering criteria during the quality control stage reduces the number of variants available with low (mostly <.01) frequency, and so may not be an accurate representation of the effects of these variants.

2.3.5 Description and functional annotation of genomic risk loci

Using FUMA, we annotated all 21,403 SNPs that were in LD (>=0.6) with one of the independent significant SNPs, allowing further inspection of these variants. Functional annotations included ANNOVAR[68], which identifies the SNP's genic position (e.g. intron, exon, intergenic), Combined Annotation Dependent Depletion (CADD)[69] scores, which predict how deleterious the effect of a SNP is on protein structure/function, RegulomeDB[70] (RDB) scores, which predict likelihood of regulatory functionality, and chromatin states from the Roadmap ChromHMM model[67,71], which predict transcription/regulatory effects from chromatin states at the SNP locus.

Of the 21,403 candidate SNPs, 21,368 were available for lookup in the annotation databases. These SNPs were mostly located in intronic (n=10,961; 51.3%) and intergenic areas (n=7,149; 33.4%) **(Supplementary Table 6; Figure 1c)**, yet 5.2% (1,103 SNPs) were annotated to functional genic regions, with 1.4% (293 SNPs) being exonic. The most likely genetic variants to have a substantial impact on a phenotype are those that have a direct consequence on a protein,

specifically variants located in coding exons that have a nonsynonymous change resulting in a change to the protein's amino acid sequence (ExNS SNPs). Of the annotated SNPs in GWS loci, we identified 146 ExNS located in 113 unique genes (**Supplementary Table 11**). Eighteen genes included more than 1 ExNS: *AKAP6, ALMS1, BSN, BTN2A1*, *CELSR3, DDX27, GNL3, ITHIH1, LINC02089, MST1R, MTMR4, PCDHA1, PCDHA3, RECQL4, RNF43, STAB1, TRIOBP,* and *ZNF638*. Sixty-four ExNS had CADD scores above 12.37 (the threshold suggested by Kircher et al.[69] to be deleterious) and 18 had a RDB score of 1d or 1f, suggesting they were likely affecting binding sites and gene expression. Apart from the protein consequences of ExNS variants, the rest of the implicated SNPs also showed evidence of indirect effects on gene products: 2.9% had a RegulomeDB[70] score of 1a-1f **(Figure 1d**), suggesting a regulatory function, and the majority of candidate SNPs (80.0%) were in open chromatin regions[67,71], as indicated by a minimum chromatin state of 1-7 (**Figure 1e**).

From the full GWAS results, the strongest signal (P=7.48^{-31}) was on chromosome 6 (risk locus 104), with the most associated lead SNP rs1906252. There were two other independent lead SNPs in the same locus (rs77418166 and rs6928545), indicative of multiple strong signals co-localized in this region of the genome (**Supplementary Tables 5** and **6; Supplementary Figure 4**). rs1906252 is located in a non-coding gene (*RP11-436D23.1*), which is specifically expressed in brain[72]. This specific SNP showed a GWS association with intelligence in two previous studies[20,73], and has also been linked to educational attainment[62] and bipolar disorder[74].

The second most associated locus (locus 50, **Supplementary Table 6**) on chromosome 3 includes 4 lead SNPs. The top lead SNP is rs2352974 (P=3.69×10^{-29}), which is in an intronic region of the *TRAIP* gene. The other three lead SNPs are rs73078367 (P=1.81×10^{-9}, in an intronic

region of *NCKIPSD*), rs13096357 (P=4.52×10^{-8}, in an intronic region of *CELSR3*), and rs1540293 (P=2.46×10^{-9}, in an intronic region of *CACNA2D2*). However, in this locus, there are also 5 exonic SNPs with CADD scores >20 (**Supplementary Table 11**). The highest CADD score is for rs13324142 (CADD score 25.6, GWAS P=1.98×10^{-87}, r^2 = 0.98 with lead SNP rs13096357 in this locus), which is a missense mutation in SLC26A6; the ancestral C allele (allele frequency = 0.90 in HRC) is associated with lower intelligence scores. rs3197999 (CADD score = 25.5; GWAS P=1.47×10^{-22}; r^2 = 0.48 with lead SNP rs73078367), is a missense mutation in the *MST1* gene, where the ancestral allele (G) is associated with lower intelligence scores and has an allele frequency of 0.71 (in HRC). rs11552724 (CADD score 25.4; GWAS P=4.63×10^{-11}; r^2 = 0.80 with lead SNP rs13096357) is a missense mutation in *USP19*, and the ancestral allele (G, allele frequency of 0.11 in HRC) is associated with higher intelligence scores. rs34759087 (CADD 23.1; GWAS P=3.22×10^{-8}; r^2 = 0.73 with lead SNP rs73078367) is a missense mutation in *LAMB2* with the ancestral allele associated with lower intelligence scores (C, allele frequency = 0.89 in HRC). And finally, rs3821875 (CADD score = 21.9; GWAS P=1.12×10^{-7}; r^2 = 0.91 with lead SNP rs13096357) is a missense mutation in *CELSR3* where the ancestral allele G (allele frequency 0.11 in HRC) is associated with lower intelligence scores. Full details of all genomic risk loci and their putative functional implications are listed in **Supplementary Tables 5 and 6**.

2.3.6 Gene mapping of GWS SNPs in genomic risk loci

We used three gene-mapping strategies implemented in FUMA[75] to select genes of interest based on SNPs in the significantly associated risk loci. For positional mapping, we mapped SNPs in the risk loci to genes using a window of 10 Kb, resulting in 522 mapped genes. eQTL mapping

resulted in 684 genes, of which 315 were located outside of the positional boundaries of the genomic risk loci, and chromatin interaction mapping resulted in 227 genes, of which 41 were located outside of the genomic risk loci. These 859 genes were mapped by FUMA based on the parameter settings as described in the methods and annotating all SNPs that are in LD ($r2 \geqq 0.6$) with an independent significant SNP. All of these genes include GWS SNPs within their genic boundaries and/or are influenced by GWS SNPs via eQTL and/or chromatin interactions. We note that they are not necessarily all relevant to intelligence: this is something that still needs to be validated in functional experiments. However, these 859 genes are starting points for generating hypotheses that can be functionally tested, because for annotated SNPs we now know the direction of effect (e.g. an allele that is associated with higher intelligence is also associated with higher expression of gene X; or an allele associated with higher intelligence leads to an altered protein structure). From this gene list, we can use the FUMA results to further select the genes that are most interesting for functional follow-up. For example, of the 859 unique genes mapped by FUMA, 189 had a probability of loss-of-function intolerance[76] (pLI) >0.90, indicating that these were extremely sensitive to mutations within the gene that result in truncation or loss of function of the protein product (**Supplementary Table 12).** In addition, for some genes we obtained converging evidence from multiple sources, such as two independent loci that point to the same gene, or that both eQTL associations outside a gene and GWS SNPs within the gene were present. Information like this may guide the order of priority for functional follow-up of these numerous reported genes.

As described in the main text, fifteen genes are particularly interesting because they are linked via chromatin interactions in one or more tissue types between two independent genomic risk

loci (**Figure 2**): *SATB2* in locus 45, *MEF2C* in locus 83, *FBXL17* and *MAN2A1* in locus 87/88, 8 genes (*ZNF184, ZNF311, ZNF322, HIST1H2AG, HIST1H2AH, HIST1H2BJ, HIST1H2BK*, and *HIST1H4I*) in locus 101/102, *ELAVL2* in locus 129, *PTCH1* in locus 130, and *ATF4* in locus 212. These interactions involved 7 pairs of independent genomic regions in linkage equilibrium and separated by >250kb. Given the prevalence of chromatin interactions found between similarly independent loci defined by our gene-mapping parameters (25,924 out of 73,499 regions as mapped in the HiC database), the probability of finding 7 or more pairs of interacting loci in our set of genomic risk loci (213 regions, or 22,578 possible pairs) was significantly higher than expected by chance (binomial test P=3.16×10^{-11}). Additional evidence supports several of these genes: all except *SATB2* and *FBXL17* also demonstrated multiple intra-locus chromatin interactions, and *FBXL17*, *MAN2A1*, *ZNF322, PTCH1,* and *ATF4* were mapped via eQTL associations in multiple tissues (**Supplementary Table 12).**

Despite the strong enrichment for GWAS association signal in genes specifically expressed in the brain (see below), these cross-locus chromatin interactions were mostly found in non-brain tissue types. Of the 55 total cross-locus interactions involving these 15 genes (the same interaction regions were frequently linked in multiple tissues), 31 were in the heart (aorta or ventricle), 11 were in the liver, and only 2 were in the brain (hippocampus); an additional 6 involved the spleen, and 5 the pancreas. While Sniekers et al.[20] found that genes significantly associated with intelligence in a previous study were most strongly enriched for expression in brain tissues, there was also some enrichment of certain genes in whole blood, lympohcytes, liver, and skeletal muscle. It is possible that this reflects the high oxygen and metabolic demands of cognitive processes, and that disruption in genetic functions that affect such

processes are consequently linked to individual differences in intelligence. However, we also note that currently available HiC data is still limited and may not be equally representative of all different tissues. For example, there are over ten-fold more interactions observed in some tissues (like ventricle and liver) than there are in brain tissues in the HiC interaction database[77]. The extent to which this is a result of true biological differences in the frequency of chromatin interactions between tissues or simply a methodological artefact due to differences in sample size, methods, and accuracy in generating this data for different tissues is currently uncertain.

We further investigated the FUMA-mapped genes for enrichment using hypergeometric tests on 7,246 pre-defined gene-sets derived from MSigDB[78] and gene expression profiles in 53 tissue types obtained from the GTEx Project[79] (**Online Methods**). We found significant differential expression of these genes in multiple brain tissues (cerebellar hemisphere, cerebellum, cortex, frontal cortex) (**Supplementary Figure 9**). These genes were also overrepresented among 15 sets of microRNA targets, 4 transcription factor binding targets, and cell adhesion and synaptic regulation pathways, among other curated gene-sets (**Supplementary Table 14**).

2.4 Gene-based (GWGAS) and Gene-set Association

2.4.1 Genome-wide gene-based association (GWGAS)

We tested 18,128 protein-coding genes for association with intelligence using MAGMA's gene-based test and detected 507 genes significantly associated at the Bonferroni corrected ($P<2.76\times10^{-6}$) threshold. Of these, 452 were novel genes which have not been previously implicated by either GWAS or GWGAS (**Supplementary Table 25**), and 157 genes were located outside the SNP-based genomic risk loci (**Supplementary Table 15**). The top gene *RNF123*

(P=1.63×10^{-33}) on chromosome 3 in locus 50 was also mapped by positional and eQTL mapping and has a pLI score of 0.97 (**Supplementary Table 12**), which is an extremely high probability of being intolerant to loss of function mutations. The protein encoded by this gene contains a C-terminal RING finger domain, a motif present in a variety of functionally distinct proteins and known to be involved in protein-protein and protein-DNA interactions, and an N-terminal SPRY domain. This protein displays E3 ubiquitin ligase activity toward the cyclin-dependent kinase inhibitor 1B which is also known as p27 or KIP1. Alternative splicing results in multiple transcript variants (description from http://www.genecards.org, provided by RefSeq, Feb 2016).

2.4.2 Gene-set association

We conducted gene-set analysis on a total of 7,323 gene-sets: 7,246 sets derived from the MSigDB, 53 tissue specific gene-sets and 24 cell specific gene-sets. The threshold for significance of gene-sets was thus set at 0.05/7,323=6.83×$10^{-6,}$ and competitive testing was used throughout (**Online Methods**).

Analysis of gene-sets from MSigDB resulted in six significantly associated gene-sets: *neurogenesis*, *neuron differentiation*, *central nervous system neuron differentiation*, *regulation of nervous system development*, *positive regulation of nervous system development*, and *regulation of synapse structure or activity* (**Supplementary Table 17**). These gene-sets are all involved in neuronal/nervous system processes and include many overlapping genes. Of 3,861 total genes in these 6 sets, only 1,509 are unique, and three sets are wholly encompassed by other sets: *central nervous system neuron differentiation* is a subset of *neuron differentiation*, which is in turn a subset of *neurogenesis*, and *positive regulation of nervous system*

development is a subset of *regulation of nervous system development*. Conditional gene-set testing was therefore used to test which of these sets was most responsible for the association signal (**Online Methods**). The strongest signal came from the *neurogenesis* gene-set, but *regulation of nervous system development* accounted for more of the variance in the other significant gene-sets than did *neurogenesis* (i.e. their association signal declined more when conditioning on this gene-set). Together, the *regulation of nervous system development, central nervous system neuron differentiation,* and *regulation of synapse structure or activity,* gene-sets accounted for the observed association signal of the other three sets (**Supplementary Table 17)**.

Of the 750 genes in the *regulation of nervous system development* gene-set, 41 genes had a significant GWGAS *P*-value and 6 of these (*CPNE1, IST1, MAN2A1, MTMR2, NEGR1,* and *RHOA*) were implicated by positional, eQTL, and chromatin interaction mapping in addition to GWGAS (**Supplementary Table 18**). Of the 166 genes in the *central nervous system neuron differentiation* set, 8 genes had a significant GWGAS *P*-value (*DCC, FGF8, PLXNA4, PTCH1, RORA, SHANK3, SZT2, TAL1*) and *PTCH1* was implicated by all four gene-mapping strategies. Of the 232 genes in the *regulation of synapse structure or activity* set, 16 genes had a significant GWGAS P-value (*STAU1, DBN1, NCAM1, SYNGR1, FLRT1, LRRC24, MEF2C, GRIN2A, NTRK3, SHISA9, BAIAP2, CTNNA2, EFNA5, SHANK3, SLC8A3, SORCS3*), and one of these (*STAU1*) was also implicated by all three other gene mapping strategies (**Supplementary Table 18**).

We additionally conducted gene-set analysis on gene-expression values from 53 GTEx tissues (**Online Methods**). We found Bonferroni-corrected significant associations for 11 tissue specific gene-sets, all of which were brain tissues, with the strongest association in cortical and

cerebellar regions (**Supplementary Table 19)**. Conditional gene-set analysis indicated that these associations were driven by a single source, either the cortex or specifically the frontal cortex (Brodmann's area 9), as the effect of all other regions disappeared when conditioning on either of these two tissues (**Supplementary Table 19**).

Cell-type specific gene-set analysis showed significant association with three cell types: medium spiny neurons, pyramidal CA1 neurons, pyramidal SS neurons, (**Supplementary Table 20**). The association with the pyramidal neurons from the somatosensory cortex likely explains the tissue specific association with the cortical areas as these neurons are enriched in cortical tissue. Conditional gene-set analysis showed that the association signal from the single-cell sets tested were driven by three independent cell types: medium spiny neurons, neuroblasts, and pyramidal CA1 neurons (**Supplementary Table 20**). Neuroblasts were not individually significant (P=4.73×10^{-4}) after applying a Bonferroni threshold, but they did account for the signal seen in other cell types such as dopaminergic neuroblasts.

The results in this section are parallel to the gene-set enrichment tests (i.e. 2x2 tests) based on FUMA-mapped genes described briefly in **Supplementary Results 2.3.6**. Both analyses concurrently indicated the strongest gene-set enrichment for genes expressed in the brain, but implicated different curated gene-sets. However, the FUMA annotated results are based on genes that are selected by their mapping from individually significant GWAS SNPs (which may be few or many SNPs linking a particular gene), while the MAGMA gene-set results are based on the aggregate gene-level association signal of all SNPs within a gene. We focus on the MAGMA gene-set results as they provide a more formal test of the association of gene-sets whose definition is based on the genes they comprise rather than specific SNPs.

2.5 Associations Between Intelligence and Other Phenotypes

2.5.1 Genetic correlations

We calculated genetic correlations (r_g) with 38 traits and found an r_g significantly different from 0 for 17 (using a corrected significance level of 0.05/38=.0013). Ten traits showed a significant negative r_g with intelligence, while seven traits showed a significant positive genetic correlation. For example, the r_g of intelligence with attention deficit hyperactivity disorder (ADHD) was −0.360 (**Supplementary Table 21; Supplementary Figure 10)**, which should be interpreted as that on average, -0.360^2=13% of genetic effects associated with a higher intelligence score are also associated with a lower risk for ADHD. This thus does not suggest that *all* genetic variants associated with higher intelligence are necessarily associated with lower risk for ADHD or are necessarily relevant for ADHD. The genetic variants associated with our measure of intelligence were negatively correlated with the genetic variants involved in a number of diseases and negative health-related outcomes (ADHD, depressive symptoms, Alzheimer's disease, schizophrenia, neuroticism, coronary artery disease, waist-hip ratio, body mass index [BMI], and waist circumference), and positively correlated with indicators of better health (longevity, former [vs. current] smoker status). Unlike the other neurocognitive and neurodevelopmental disorders examined, autism and intelligence had a positive rather than negative genetic correlation, which has been observed previously[20] and is consistent with some theories that autism involves a dysregulation of the systems governing cognitive development[80]. The genetic variants associated with intelligence were also positively correlated with those linked to the anthropometric traits of infant head circumference and intracranial volume. As has been

commonly observed, genetic variants associated with higher intelligence were also related to genetic influences on socioeconomic outcomes, being correlated strongly with higher educational attainment and with delayed reproductive age (higher age of first birth) and having fewer children.

2.5.2 Overlap of intelligence risk loci with risk loci implicated in other phenotypes

Comparing the GWS loci identified by this study with those in the previously reported literature using the NHGRI-EBI catalog (**Online Methods**), we found that loci significantly associated with intelligence in the SNP meta-analysis have also been linked to psychiatric phenotypes including schizophrenia (20 loci), mood disorders (5 loci), and neuroticism (5 loci), as well as educational attainment (24 loci), brain volume (2 loci), Alzheimer's disease (1 locus) and a number of physiological traits and diseases (**Supplementary Table 22**). Genes implicated by GWS SNP mapping in FUMA were most strongly enriched for sets of genes linked to schizophrenia (P=1.35×10^{-53}), educational attainment (P=9.91×10^{-37}), and cognitive function (P=1.78×10^{-24}) (**Supplementary Table 23**). These results indicate a common biological pathway implicating both normal variation in cognitive functioning and neurocognitive dysfunction.

2.5.3 Mendelian randomization with correlated traits

We performed Mendelian Randomization (MR) using Generalized Summary-data-based Mendelian Randomization[81] (GSMR, see **URLs**; **Online Methods**) to test for credible causal associations between intelligence and traits that were significantly genetically correlated with intelligence (described in **Supplementary Results 2.5.1**; **Supplementary Table 21;**

Supplementary Figure 10). We excluded traits for which there was significant sample overlap with the current intelligence meta-analysis, as known or as indicated by the intercept of the bivariate LD score regression. This was the case for age of first child, coronary artery disease, longevity, infant head circumference, number of children, neuroticism and depressive symptoms. For educational attainment, Mendelian randomization was performed on GWAS summary statistics based on the non-overlapping sample used in the proxy replication analyses **(Supplementary Results 2.3.2).** In the reverse causation analysis (i.e. testing the opposite hypothesis that the other trait causes intelligence), there were 3 traits with less than 10 independent lead SNPs. Relaxing this threshold to $P < 1\times10^{-5}$ allowed for a sufficient number of SNPs to include autism, former smoker status, and intracranial volume.

We performed analyses in two ways in order to test bidirectionality: forward GSMR was performed using GWS SNPs associated with intelligence as the instrumental variables (exposure) and correlated traits from non-overlapping samples as the outcome, while reverse GSMR was performed using GWS SNPs from the other traits as instrumental variables and intelligence as the outcome. All analyses were run before and after removing SNPs detected by the HEIDI outlier tool as showing pleiotropic effects. Results are shown in **Supplementary Table 26** and **Supplementary Figures 11-12.** A large drop in significance of the estimated *b* after removing SNPs with pleiotropic effects indicates that a substantial part of the observed association between two traits is due to pleiotropic SNPs.

The GSMR bidirectional analyses for intelligence and educational attainment showed weak evidence for pleiotropy: HEIDI detected 12 suspected pleiotropic SNPs (out of 239) in the intelligence -> educational attainment analysis and a somewhat larger proportion (7 SNPs out

of 47) in the reverse analysis. Also, the *P*-value of the estimated associations were similar before and after removal of pleiotropic SNPs. Both directions of causation were highly significant, and the causal effect of intelligence on educational attainment ($\hat{b}_{xy}$ = 0.549, $P<1\times10^{-320}$) was similar in magnitude to the effect of educational attainment on intelligence ($\hat{b}_{yx}$ = 0.480, $P=6.85\times10^{-82}$), suggesting a bidirectional effect. Similarly, a bidirectional effect was observed with intracranial volume, with a somewhat stronger positive effect of intelligence on intracranial volume ($\hat{b}_{xy}$ = 0.246, $P=2.97\times10^{-13}$) than the reverse ($\hat{b}_{yx}$ = 0.068, $P=8.44\times10^{-20}$).

We also observed a protective effect of intelligence on several neurological and psychiatric disorders. Intelligence was associated with lower risk for ADHD (OR=0.48, $\hat{b}_{xy}$=−0.734, $P=2.57\times10^{-46}$), with little evidence for pleiotropic effects. The OR of 0.48 can be interpreted such that individuals whose intelligence scores are 1 SD above the population mean have 52% lower risk of ADHD compared to the population prevalence. The reverse test (that the presence of ADHD causes lower intelligence) was also statistically significant, but with a smaller effect size ($\hat{b}_{yx}$=−0.084, $P=4.41\times10^{-19}$). A modest association was also observed between higher intelligence and higher risk for autism (OR=1.38, *bxy*=0.321, $P=1.12\times10^{-3}$), with no effect in the opposite direction (although it should be noted that there were few instrumental SNPs available to index genetic influences on autism).

Intelligence also had a protective effect on Alzheimer's disease (OR=0.65, $\hat{b}_{xy}$=−0.435, $P=3.59\times10^{-14}$), with only two SNPs (out of 233) showing pleiotropic effects, and no evidence for reverse causation. This association is potentially complicated by the fact that the sample for intelligence included older adults whose cognitive test scores may be influenced by genetic effects on cognitive decline (rather than ability), even after screening procedures to exclude

observable instances of dementia or Alzheimer's. We therefore re-ran the meta-analysis and corresponding GSMR analysis using a subset of participants from UKB aged <50 and the other cohorts under this age (GenR, IMAGEN, BLTS-C, BLTS-A, S4S, STR, and TEDS), with a total N = 59,590. The genetic correlations for this subset, calculated with LD score regression, were 0.99 with the full meta-analysis sample and 0.71 with the older adult (M_{age}>60) subset. Although the pattern of results appeared similar to the full meta-analysis, only 6 independent GWS lead SNPs were found for intelligence in this smaller subset, therefore we reduced the significance threshold to $P < 1\times10^{-5}$ for the purposes of GSMR. The GSMR results remained similar, with intelligence demonstrating a somewhat attenuated protective effect on Alzheimer's (OR=0.83, $\hat{b}_{xy}$=−0.191, $P=2.35\times10^{-5}$) and zero out of 189 variants showing pleiotropic effects. These results are indicative of a phenotypic causal effect of intelligence on Alzheimer's that is most likely independent of any pleiotropic SNPs affecting both traits through impacts on cognitive decline.

The association between intelligence and schizophrenia was more complex. GSMR detected a strong protective effect of intelligence on schizophrenia (OR=0.50, *bxy*=−0.685, $P=2.02\times10^{-57}$) and reverse GSMR analysis showed evidence of a bidirectional association, indicating that schizophrenia may cause impaired cognitive functioning, although this effect was relatively weaker (*bxy*= −0.214, $P=4.19\times10^{-52}$). There was also strong evidence for pleiotropic effects of 31 SNPs (out of 240) identified in the intelligence -> schizophrenia analysis and 18 SNPs (out of 111) in the reverse, with a substantial decrease in the effect of intelligence on schizophrenia after filtering these pleiotropic SNPs.

We further found potentially causal relationships with several anthropomorphic and health-related outcomes. Waist circumference had a modest effect on lower intelligence, while

conversely it was found that intelligence had a modest impact on lower waist-hip ratio and body mass index. The opposite causal directions were not significantly supported for any of these three phenotypes. Finally, an association was observed between higher intelligence and higher likelihood of being a former (vs. current) smoker, with no significant reverse effect, indicating that individuals with higher intelligence are more likely to successfully quit smoking.

The precise mechanisms underlying the putative causal relationships suggested here cannot be determined from the available data. Higher intelligence is prospectively predictive of better socioeconomic outcomes[82] and lower mortality/morbidity later in life[83] so it is possible that the protective effects observed for some outcomes are mediated through better social/economic resources such as access to preventative care and healthier environments, or engagement in lifestyle choices (diet, exercise, social activities, substance use) that prevent cognitive and/or physical decline. Alternatively, these associations may be a function of direct biological effects by which genetic variants that increase intelligence simultaneously lower risk for psychiatric illness, perhaps due to their effects on brain structure/function or overall health.

It is also possible that these associations (particularly for Alzheimer's disease) were observed because the variants identified in our meta-analysis contained a mixture of genetic effects impacting intelligence as well as cognitive decline since our meta-analyzed sample covered a wide age range. However, the high genetic correlation across age groups and the consistency of the GSMR results in the younger age subset suggests that this is not likely the case. Further, we saw no evidence of GWAS association with the *APOE* gene, which massively impacts Alzheimer's disease risk and general cognitive decline[84] and would be evident in the association results if the phenotypes indexed cognitive decline rather than general intelligence. The *APOE*

gene was not identified by any of our gene mapping strategies or gene/gene-set analyses, and none of the GWS SNPs identified in the most recent Alzheimer's GWAS[85] were among the GWS SNPs for intelligence. Although one identified genomic locus overlapped a region previously linked to Alzheimer's (**Supplementary Table 22**), there was not a significant enrichment for genes associated with both phenotypes (**Supplementary Table 23**). This suggests that the causal pathway between intelligence and Alzheimer's may be more likely to emerge through protective lifestyle factors correlated with higher intelligence than through direct biological pathways between the phenotypes. We speculate that other observed causal relationships implied by the GSMR results (e.g., height, BMI, smoking) are also likely to come about through lifestyle factors associated with higher intelligence or its associated higher socioeconomic status, but these mechanisms remain to be tested.

Our results are in line with evidence from other epidemiological, longitudinal, and co-twin control study designs which support these putative causal relationships, although true experimental research is necessarily lacking for these phenotypes due to ethical and practical constraints. For example, evidence exists for both a bidirectional relationship and a shared genetic etiology between intelligence and educational attainment[60,86-90], with numerous studies finding genetic overlap between them but also prospective evidence that intelligence predicts later educational outcomes and vice versa. Empirical research also supports the existence of a shared genetic etiology between better cognitive function and lower risk for schizophrenia, in addition to direct causal links between lower intelligence and schizophrenia (though not the bidirectional causal effects observed here)[91-95]. There is less literature to support the other findings, but they are consistent with the well-replicated link between higher intelligence and

better health-related outcomes across multiple domains[83]. While our results are limited by the availability of GWS SNPs for various phenotypes used to conduct the GSMR analyses, they provide incremental molecular genetic support to the existing literature and point to potential causal mechanisms for the commonly observed correlational relationships that merit in-depth investigation.

3. Supplementary Figures

UK Biobank (web-based test)

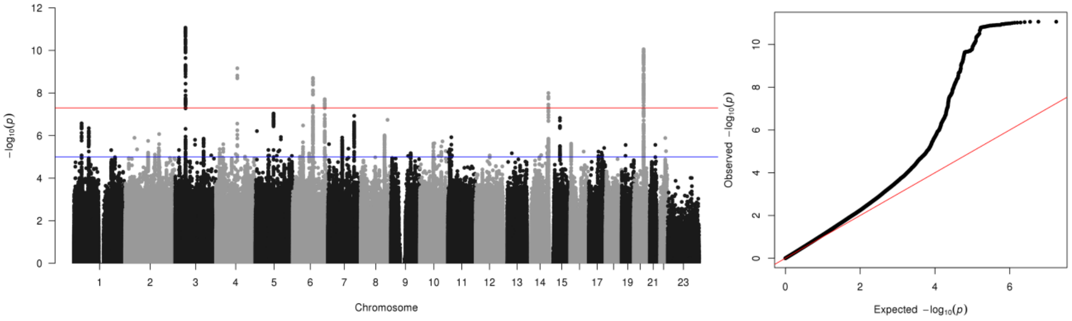

UK Biobank (touch-screen test)

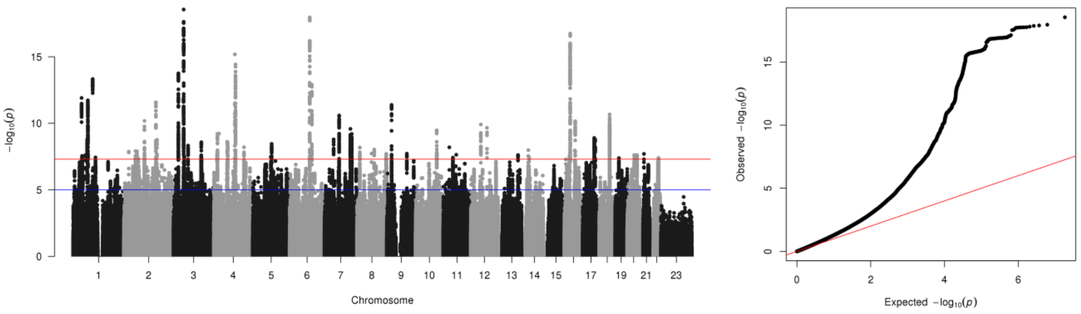

Cognitive Genomics Consortium

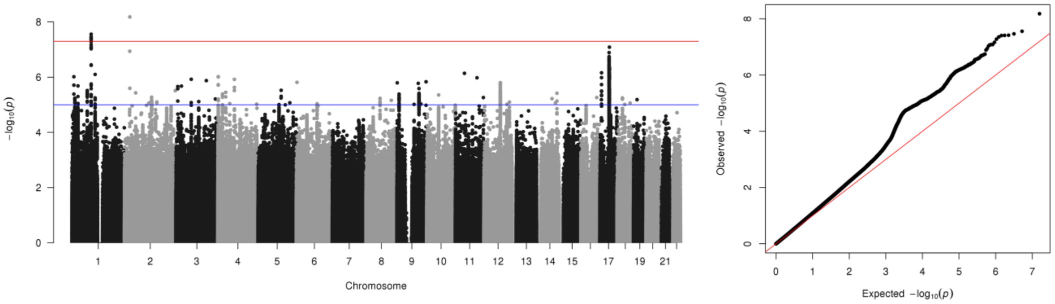

Rotterdam Study

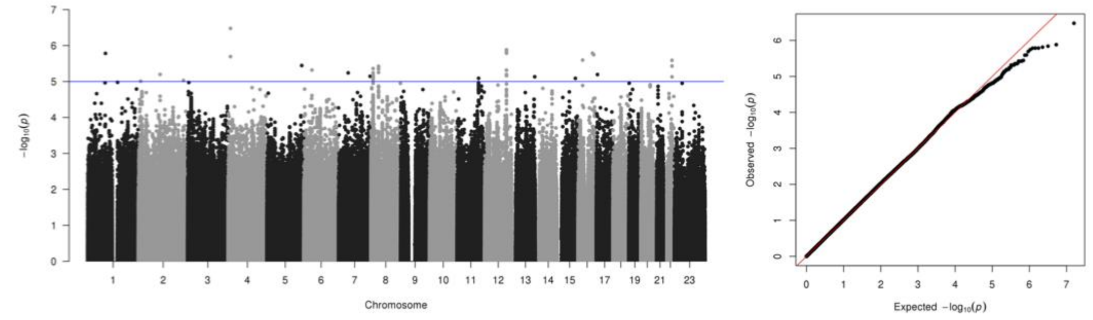

Generation R

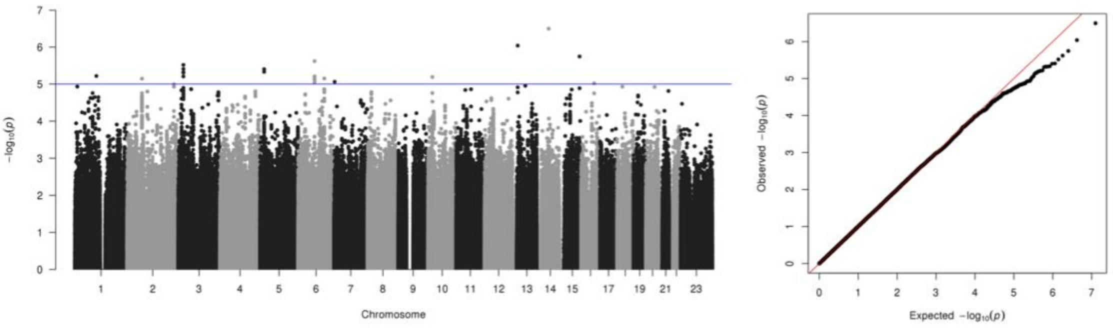

Spit for Science

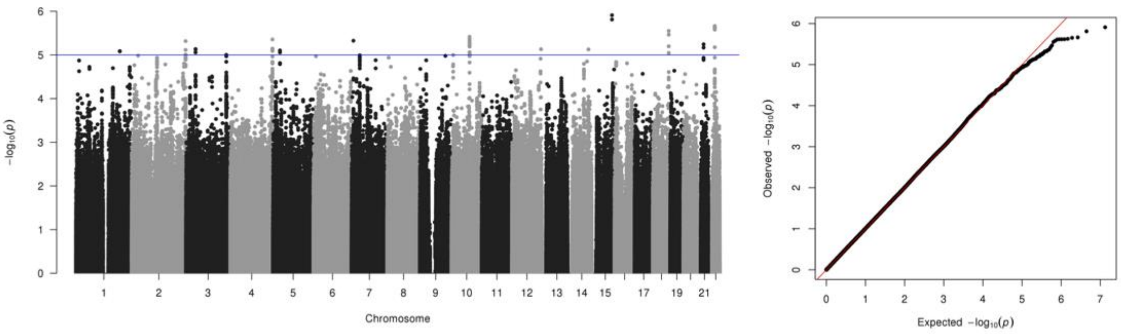

High IQ / Health and Retirement Study

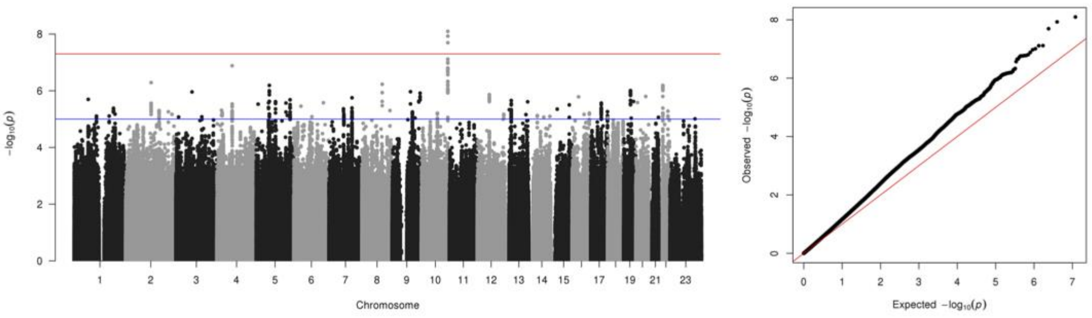

Twins Early Development Study 1

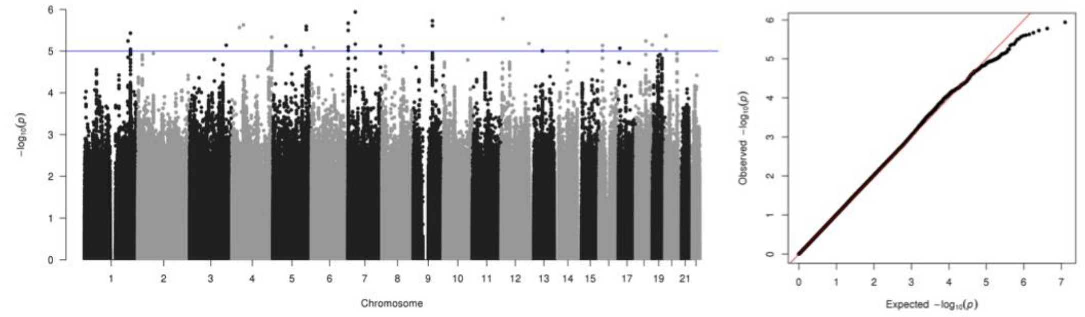

Twins Early Development Study 2

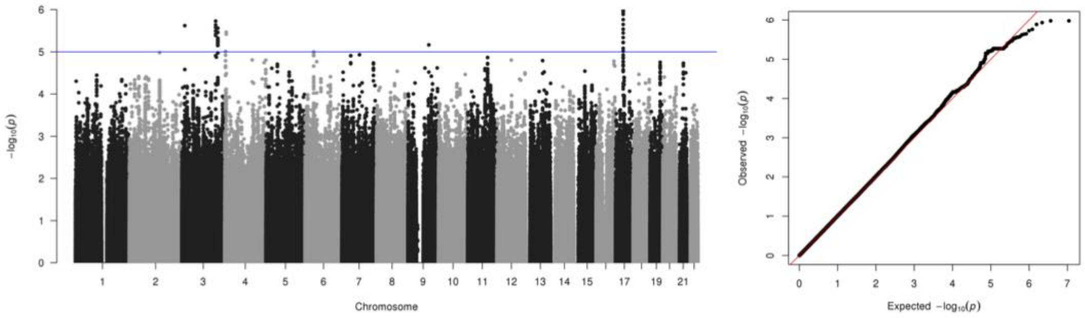

Middle-Aged Danish Twins study

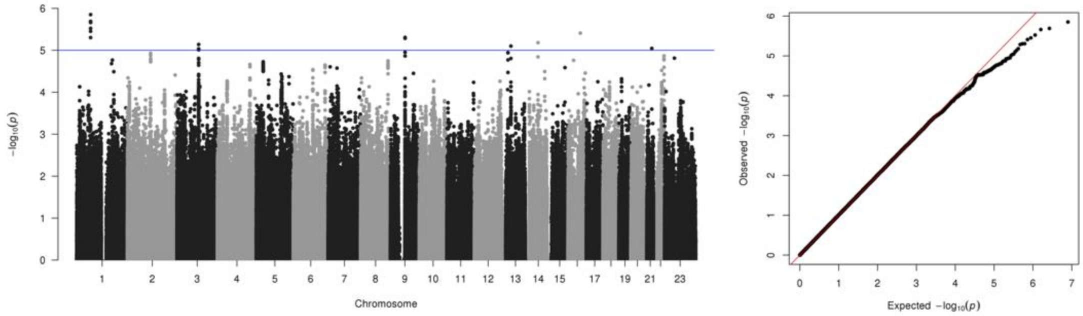

Longitudinal Study of Aging Danish Twins

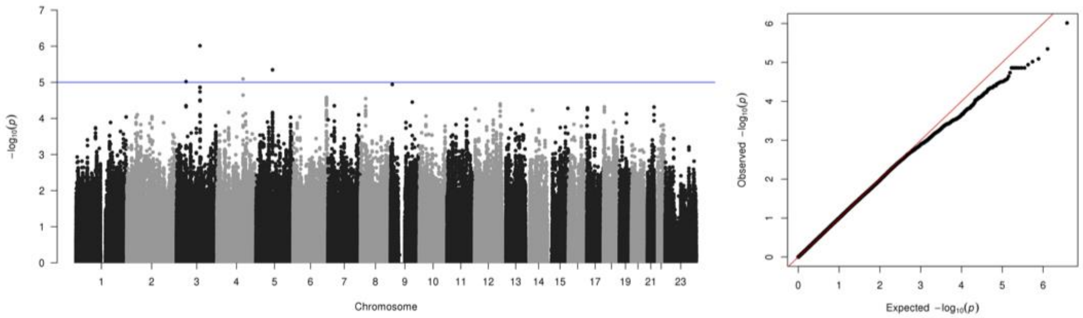

IMAGEN

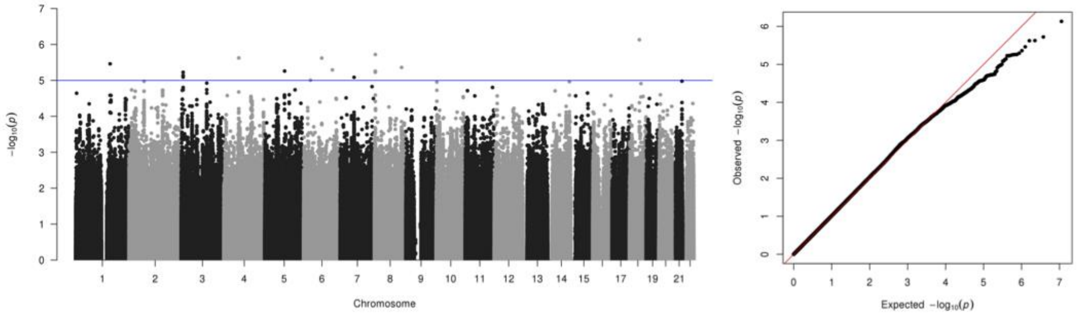

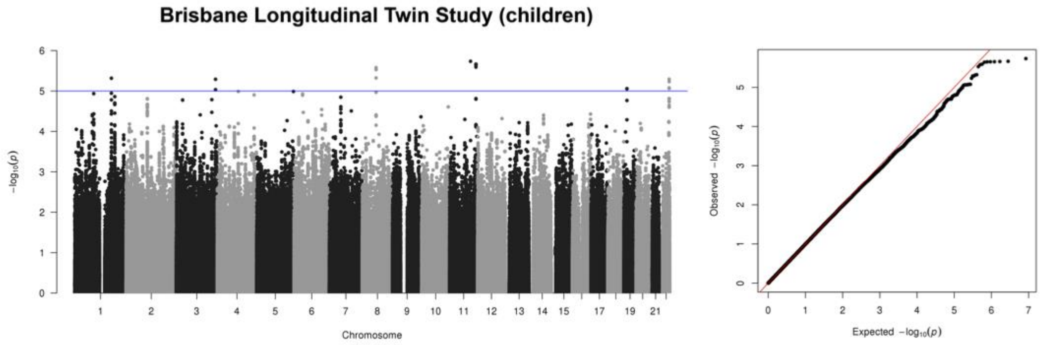
Brisbane Longitudinal Twin Study (children)
$-\log_{10}(p)$
Chromosome
Observed $-\log_{10}(p)$
Expected $-\log_{10}(p)$

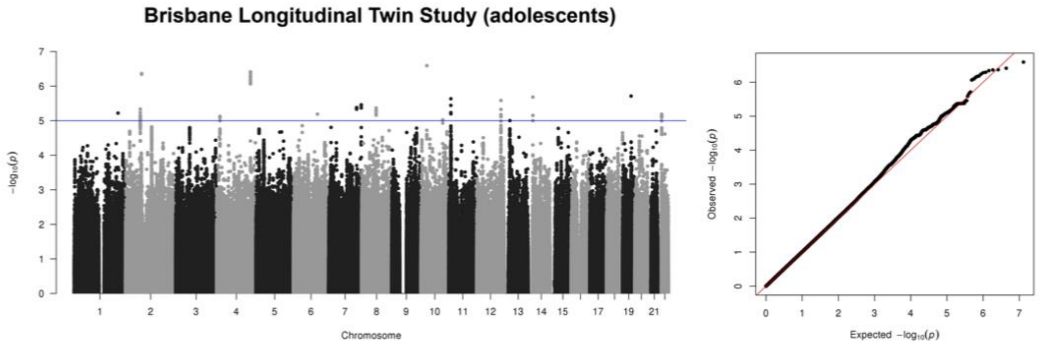
Brisbane Longitudinal Twin Study (adolescents)
$-\log_{10}(p)$
Chromosome
Observed $-\log_{10}(p)$
Expected $-\log_{10}(p)$

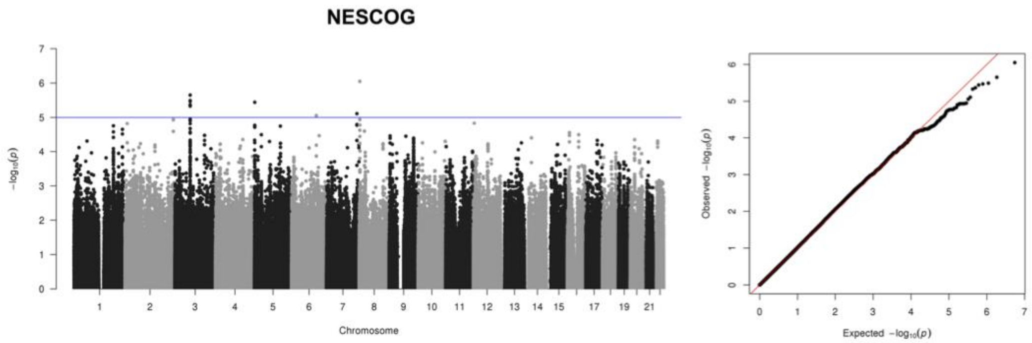
NESCOG
$-\log_{10}(p)$
Chromosome
Observed $-\log_{10}(p)$
Expected $-\log_{10}(p)$

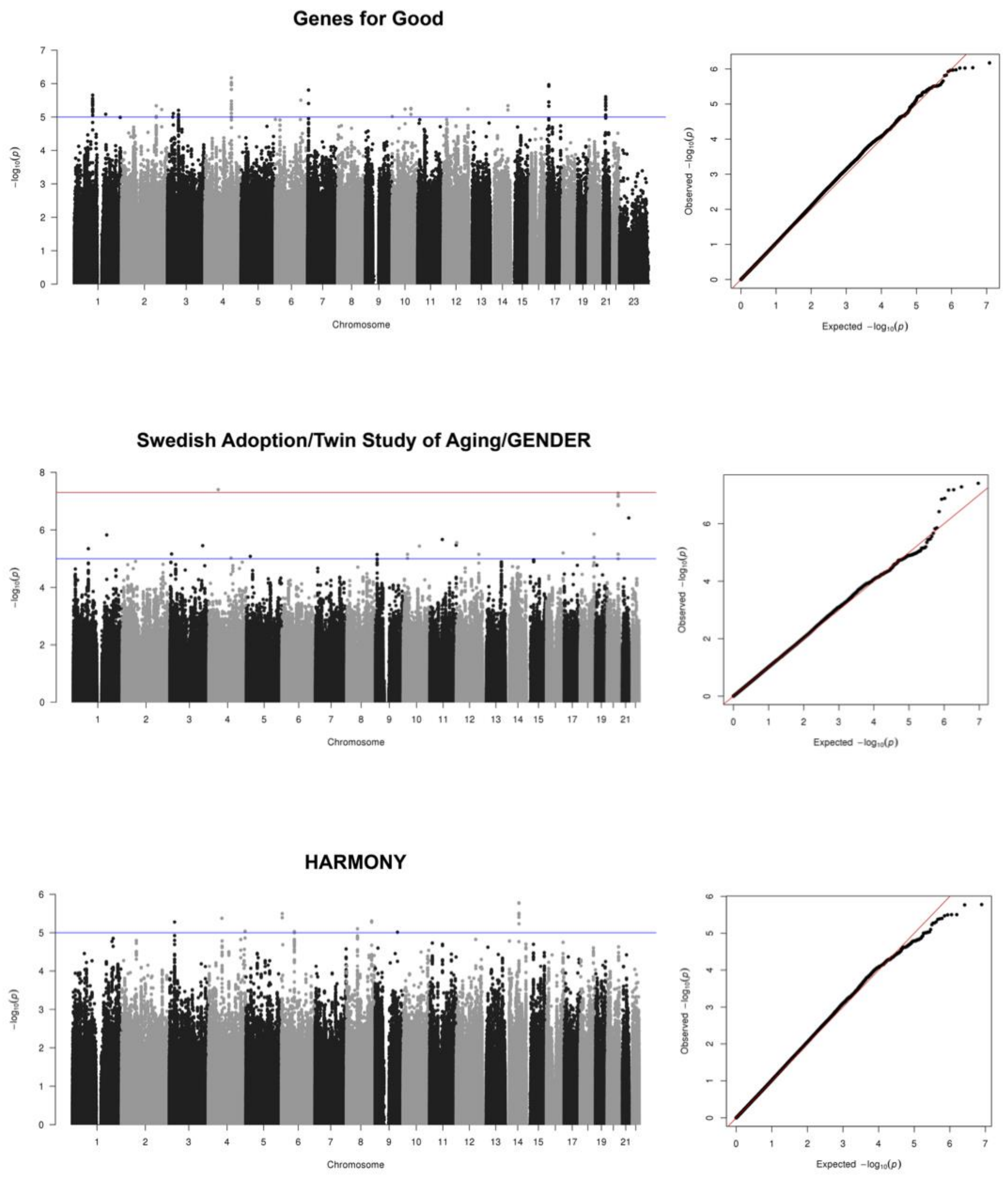

Supplementary Figure 1. Manhattan and QQ plots of the individual cohort GWAS results included in a meta-analysis of intelligence in 269,867 independent individuals. For each of 14 cohorts included in the GWAS meta-analysis, -log10 transformed two-tailed *P*-values of SNP associations with intelligence measures in a linear or logistic regression model are presented against their chromosomal position in a Manhattan plot (left) and against expected null *P*-values in a QQ plot (right). Sample sizes and details of the statistical analyses for each cohort are presented in Supplementary Information 1.1. The dotted red line indicates Bonferroni-corrected genome-wide significance ($P<5\times10^{-8}$), the blue line the threshold for suggestive associations ($P<1\times10^{-5}$).

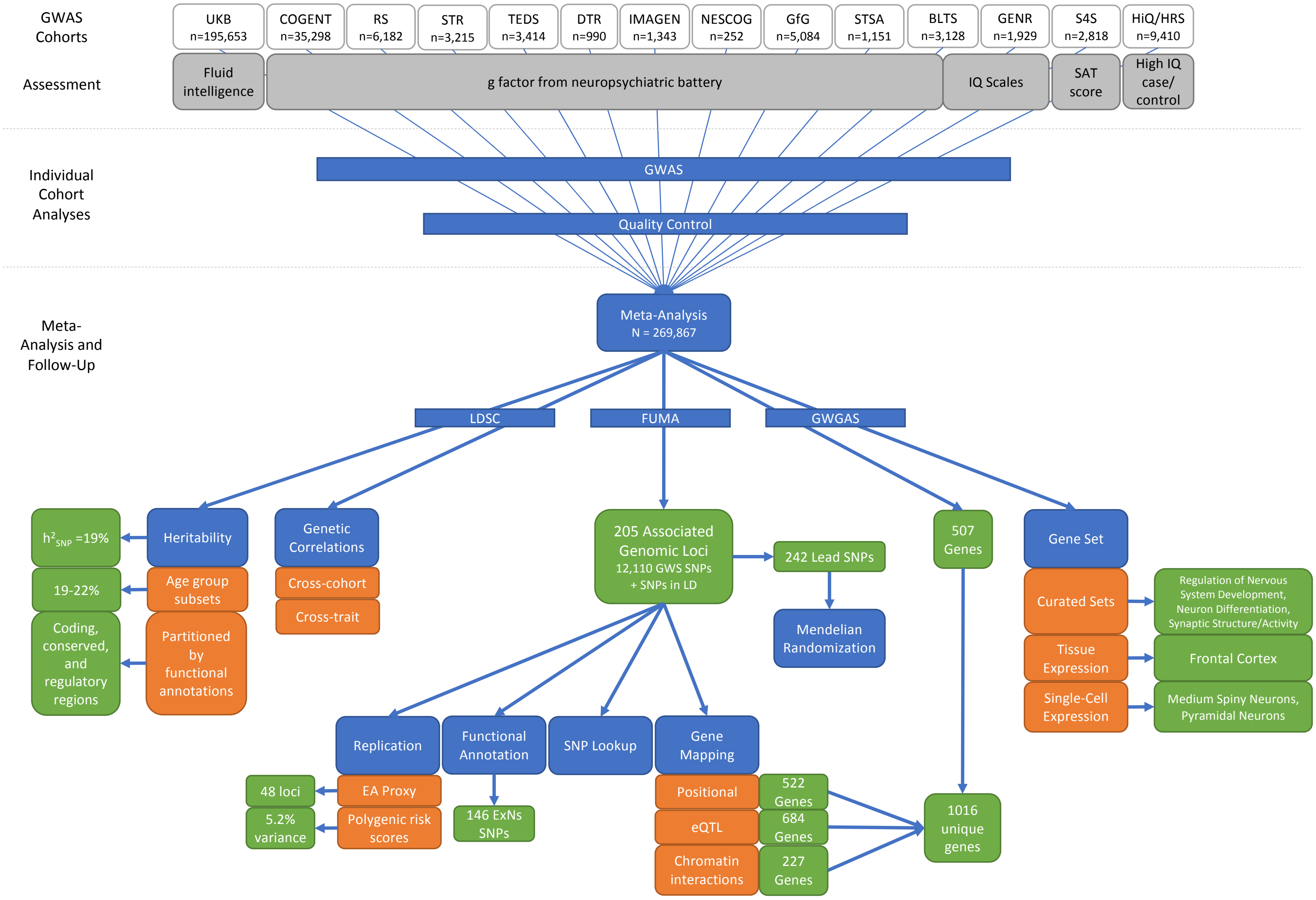

Supplementary Figure 2. Flowchart of study methodology and results of a GWAS meta-analysis of intelligence in 269,867

independent individuals. White boxes indicate study cohorts, grey boxes indicate assessment measures, blue boxes indicate groups of analytic procedures, orange boxes indicate sub-analyses, green boxes indicate results. GWAS = genome-wide association; LDSC = linkage disequilibrium (LD) score regression; FUMA = functional mapping and annotation; GWGAS = genome-wide gene-based association; SNP = single nucleotide polymorphism; EA = educational attainment; ExNS = exonic non-synonymous; eQTL = expression quantitative trait locus.

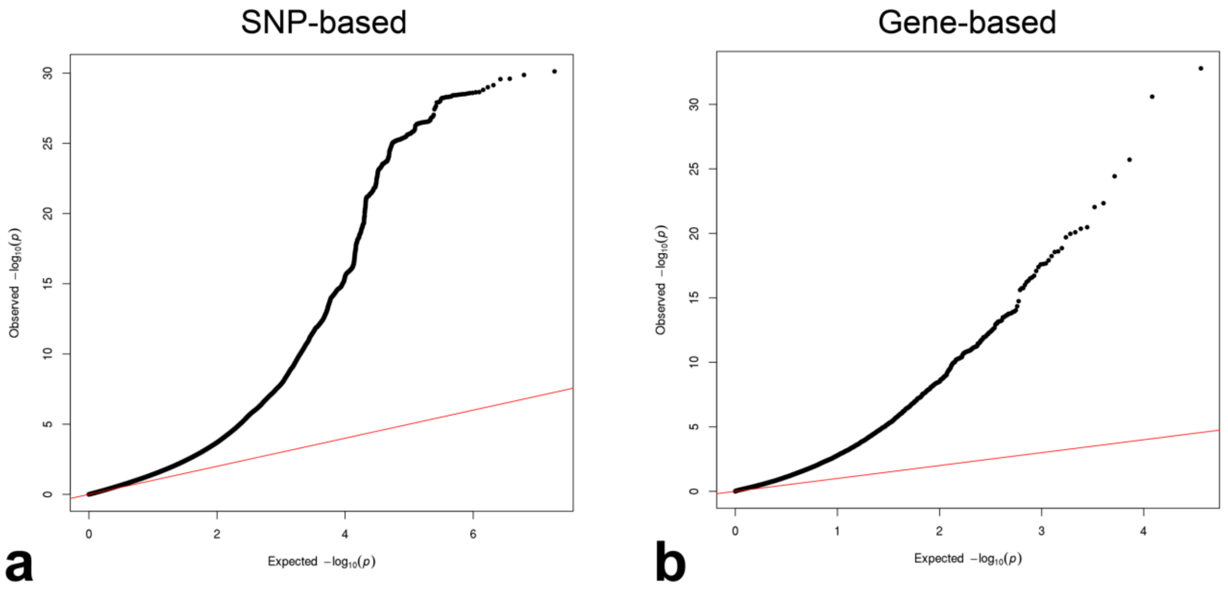

Supplementary Figure 3. QQ-plots of SNP and gene association results in a meta-analysis of intelligence in 269,867 independent individuals. Observed -log10 transformed two-tailed *P*-values of associations with intelligence measures are plotted against expected null *P*-values for **a)** all SNPs in the GWAS meta-analysis, and **b)** all genes in the gene-based meta-analysis.

Locus 1

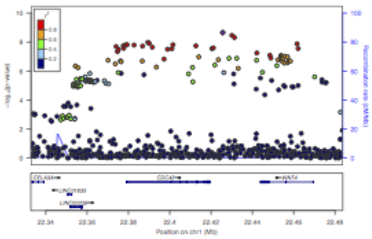

Locus 2

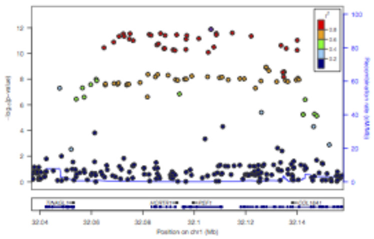

Locus 3

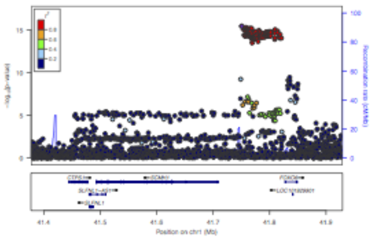

Locus 4

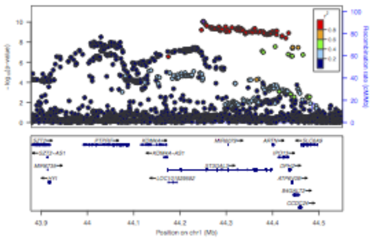

Locus 5

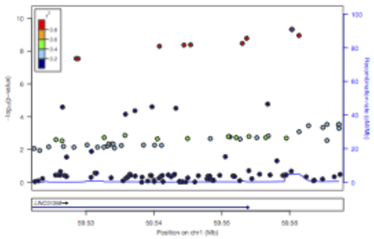

Locus 6

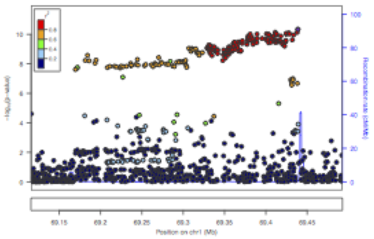

Locus 7

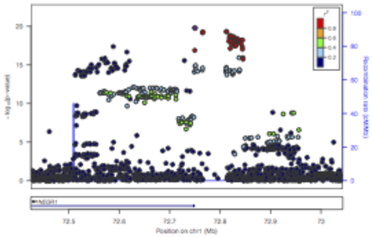

Locus 9

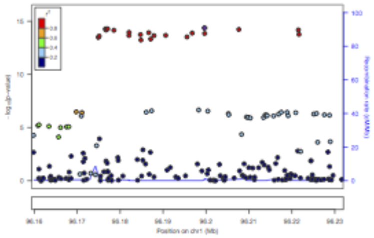

Locus 10

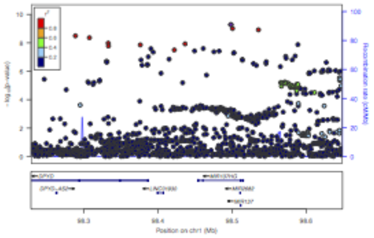

Locus 11

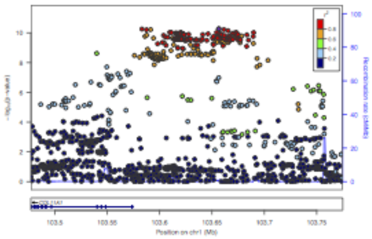

Locus 12

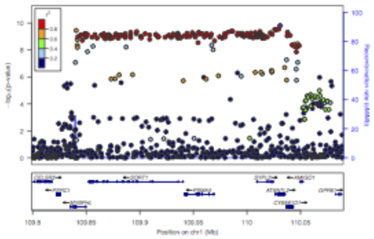

Locus 13

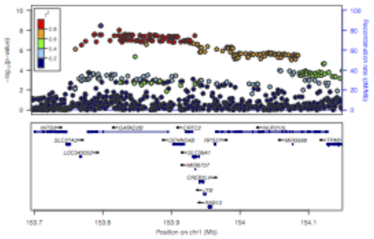

Locus 14

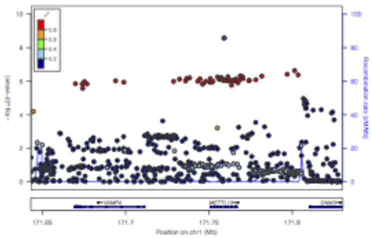

Locus 15

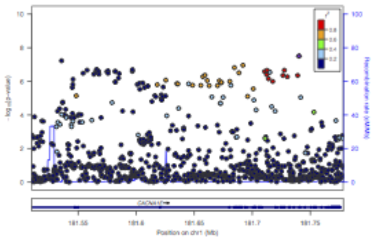

Locus 16

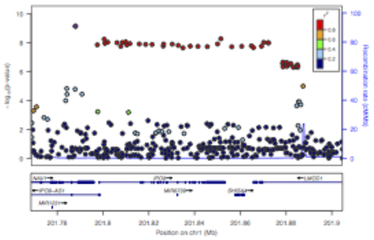

Locus 17

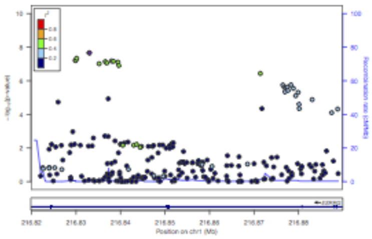

Locus 18

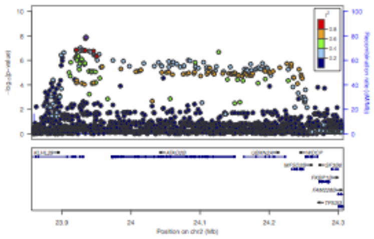

Locus 19

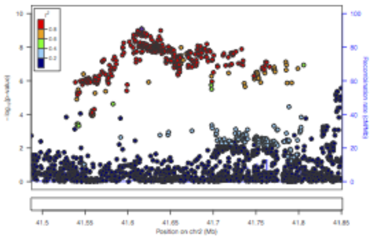

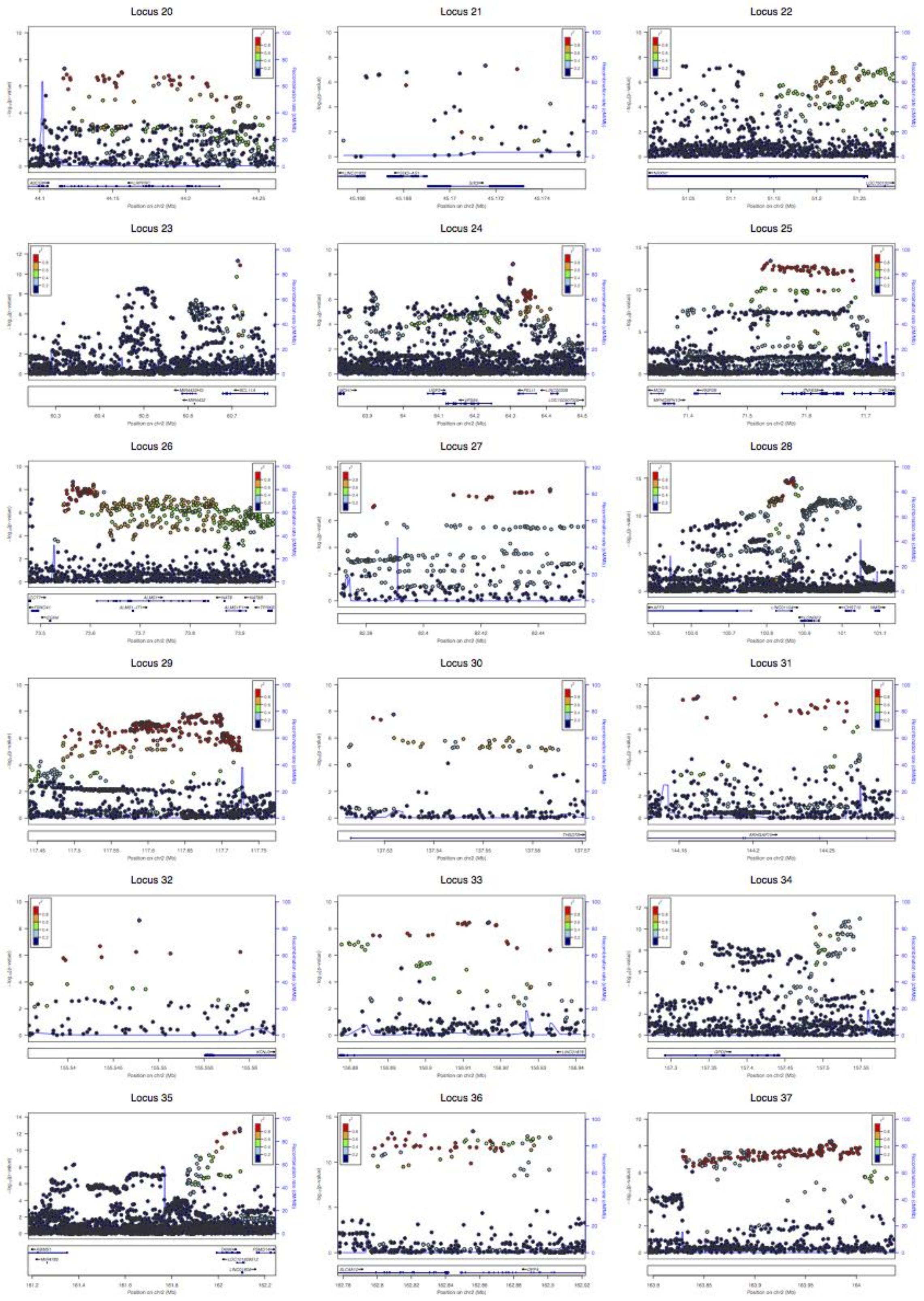
Locus 20
Locus 21
Locus 22
Locus 23
Locus 24
Locus 25
Locus 26
Locus 27
Locus 28
Locus 29
Locus 30
Locus 31
Locus 32
Locus 33
Locus 34
Locus 35
Locus 36
Locus 37

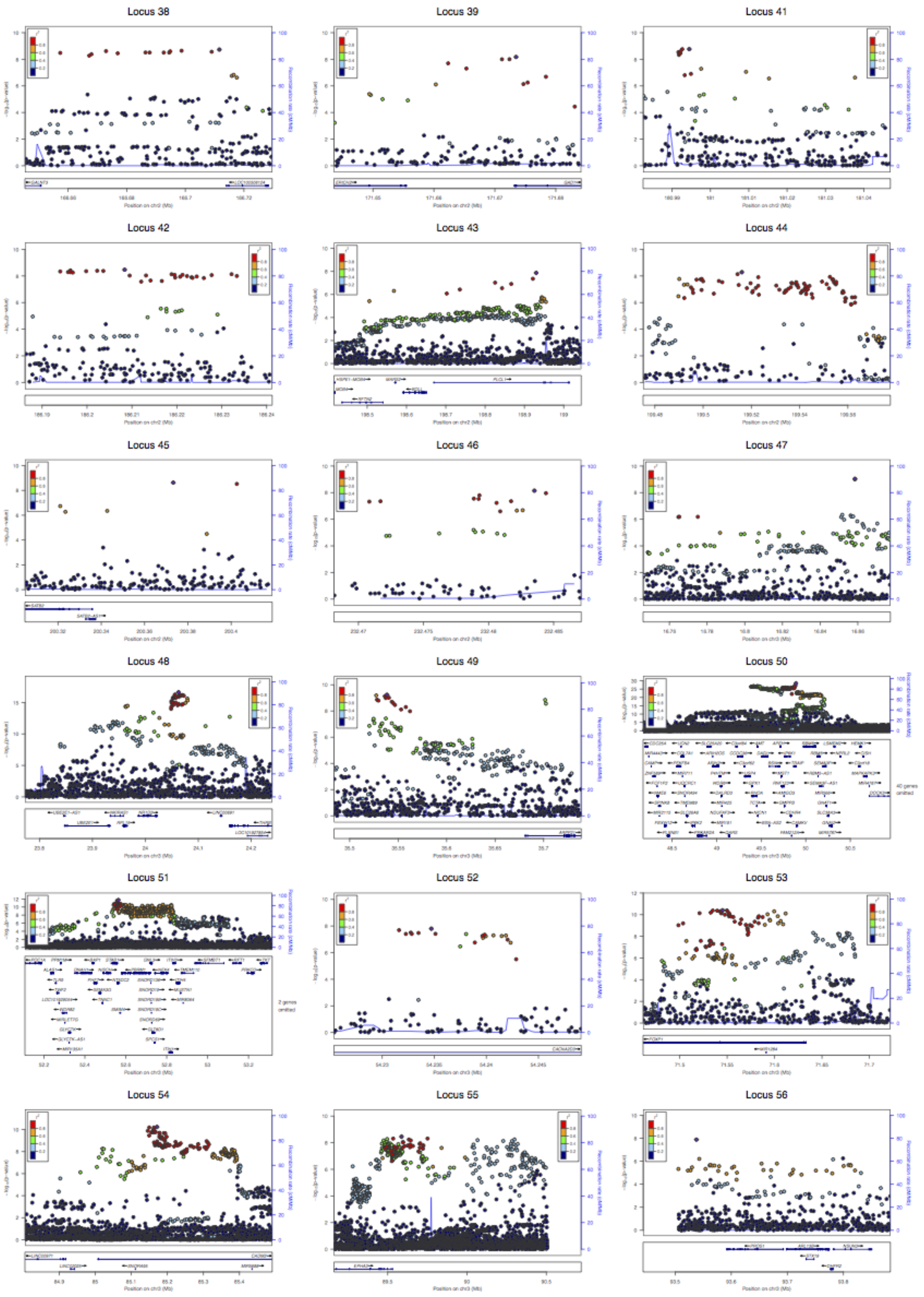
Locus 38
Locus 39
Locus 41
Locus 42
Locus 43
Locus 44
Locus 45
Locus 46
Locus 47
Locus 48
Locus 49
Locus 50
Locus 51
Locus 52
Locus 53
Locus 54
Locus 55
Locus 56

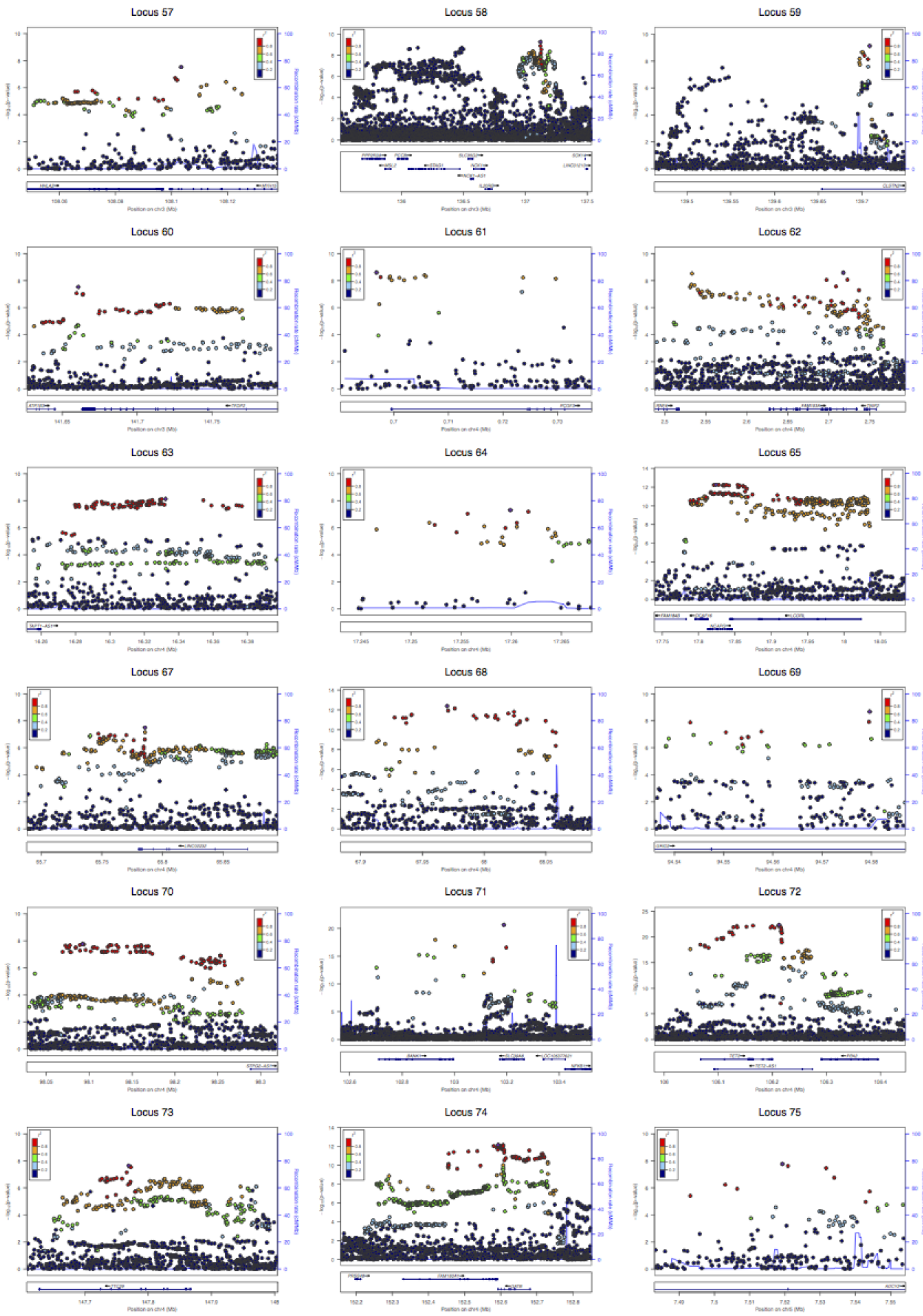
Locus 57
Locus 58
Locus 59
Locus 60
Locus 61
Locus 62
Locus 63
Locus 64
Locus 65
Locus 67
Locus 68
Locus 69
Locus 70
Locus 71
Locus 72
Locus 73
Locus 74
Locus 75

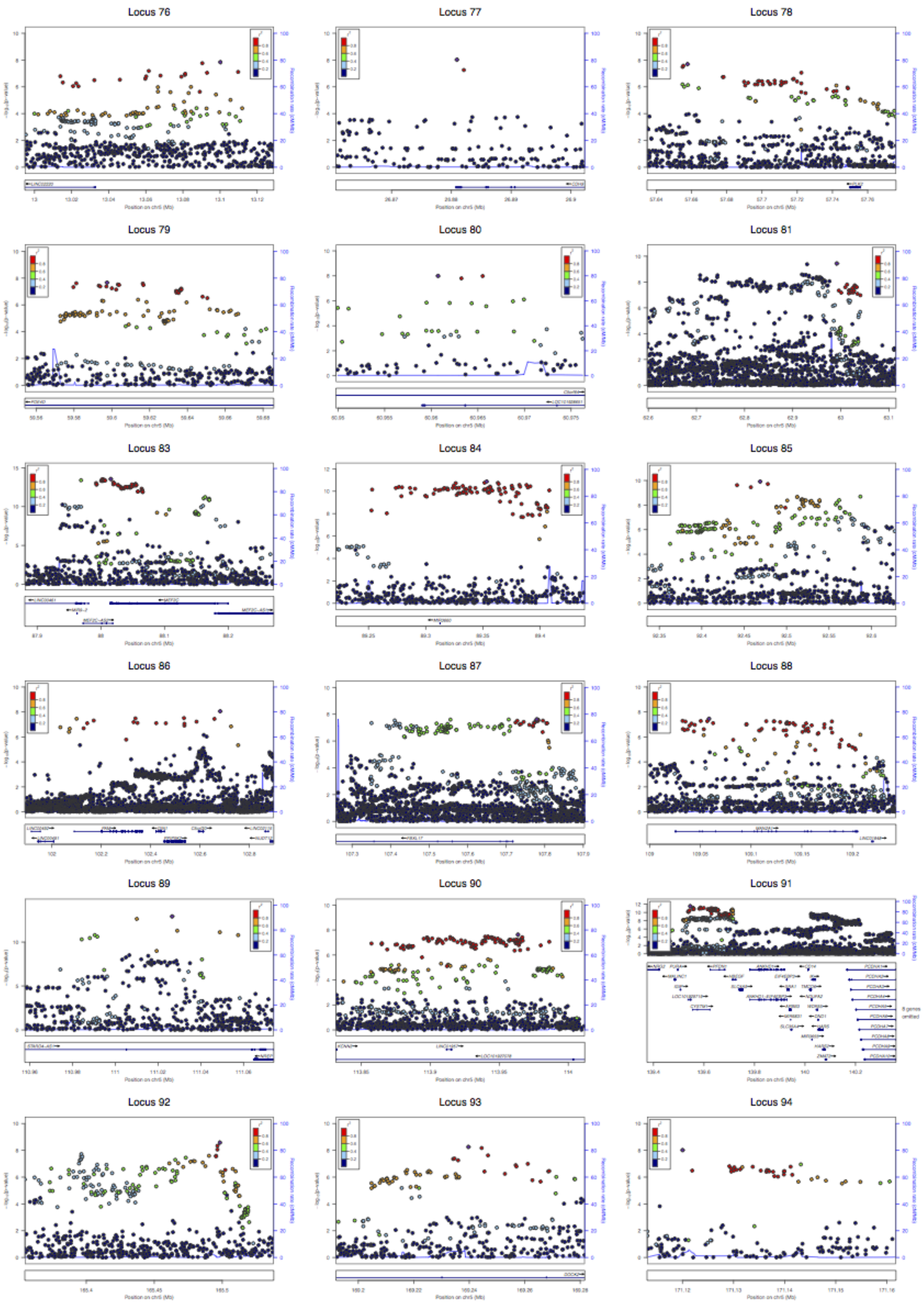
Locus 76
Locus 77
Locus 78
Locus 79
Locus 80
Locus 81
Locus 83
Locus 84
Locus 85
Locus 86
Locus 87
Locus 88
Locus 89
Locus 90
Locus 91
Locus 92
Locus 93
Locus 94

Locus 95

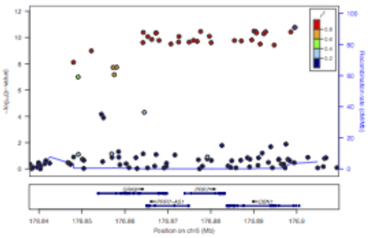

Locus 96

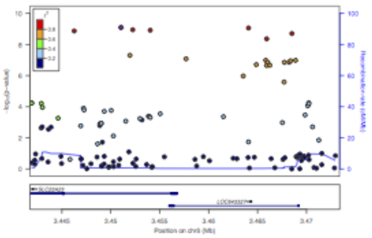

Locus 97

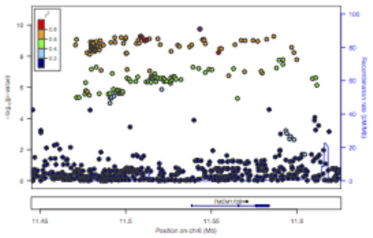

Locus 98

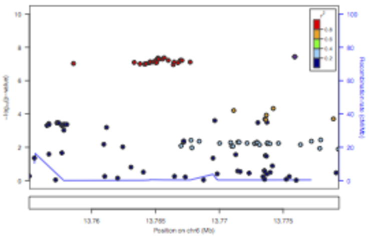

Locus 99

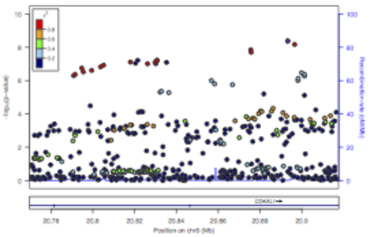

Locus 100

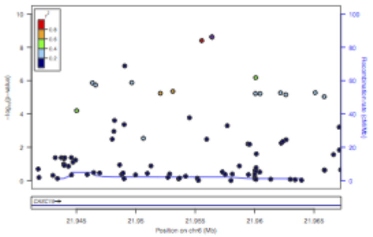

Locus 101

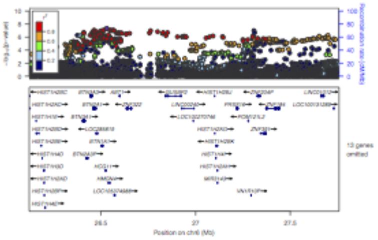

Locus 102

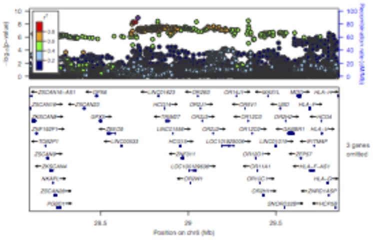

Locus 103

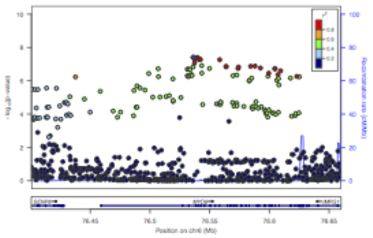

Locus 104

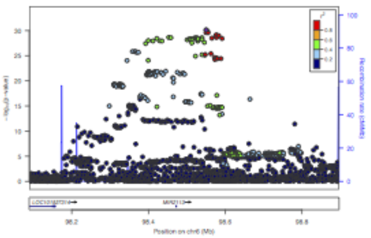

Locus 105

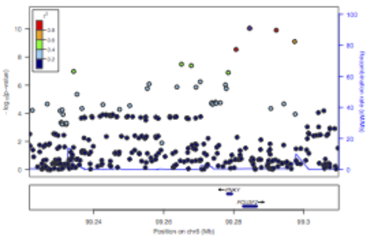

Locus 106

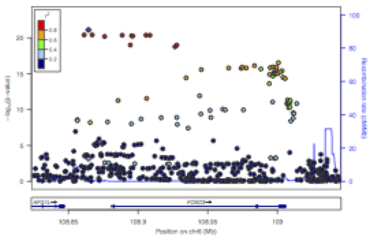

Locus 107

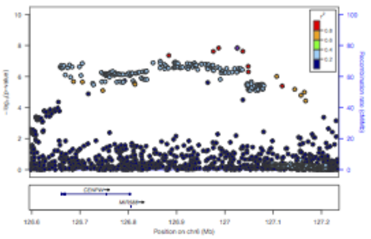

Locus 108

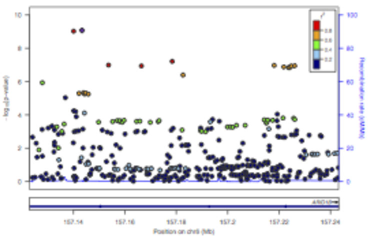

Locus 109

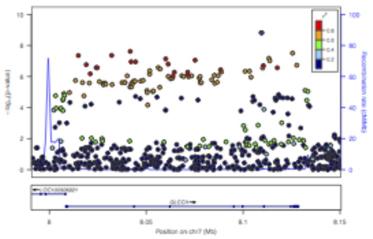

Locus 110

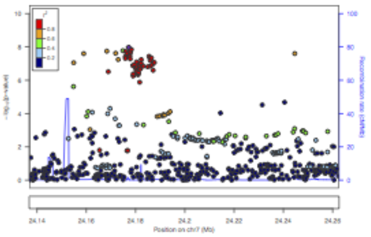

Locus 111

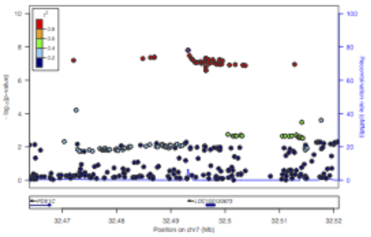

Locus 112

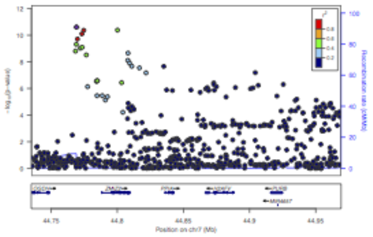

Locus 113

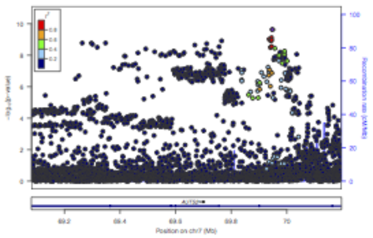

Locus 114

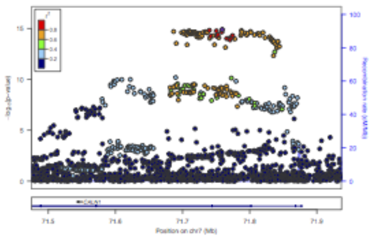

Locus 115

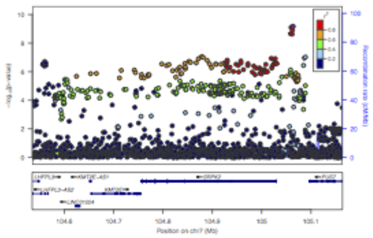

Locus 116

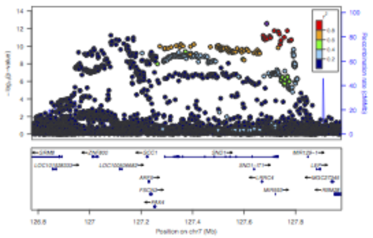

Locus 117

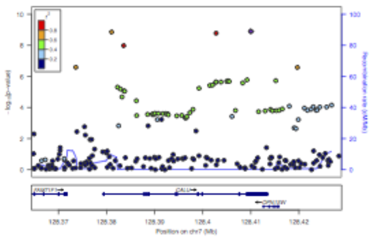

Locus 118

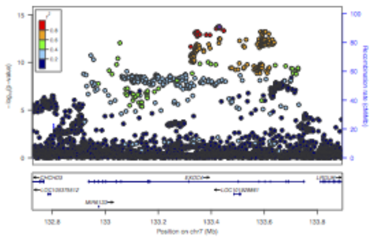

Locus 119

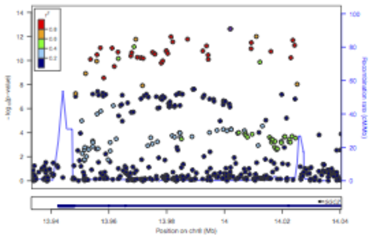

Locus 120

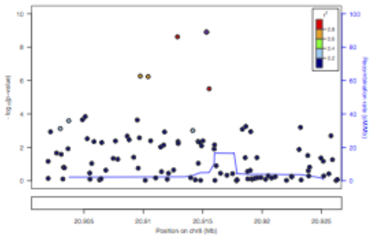

Locus 121

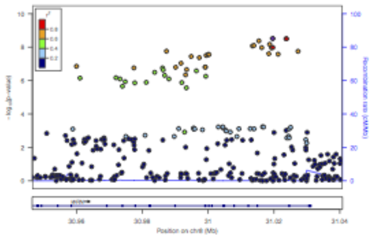

Locus 122

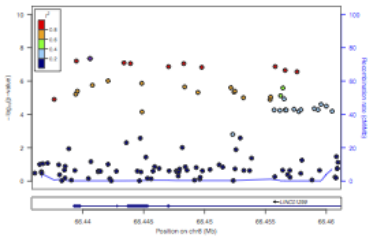

Locus 123

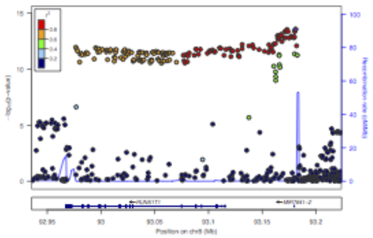

Locus 125

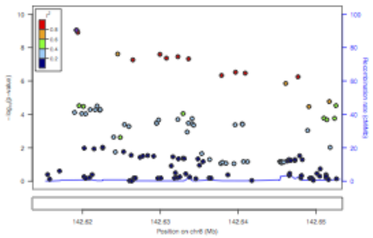

Locus 126

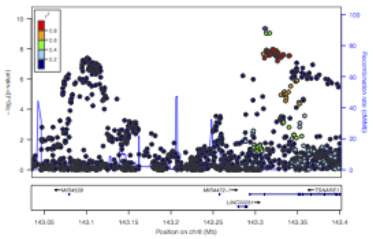

Locus 127

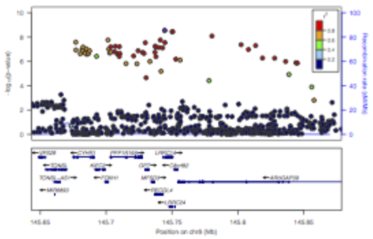

Locus 128

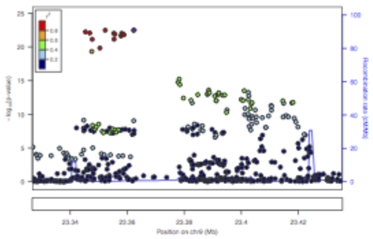

Locus 129

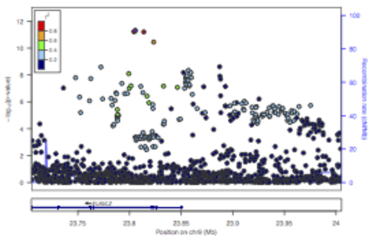

Locus 130

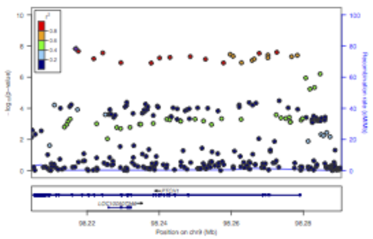

Locus 131

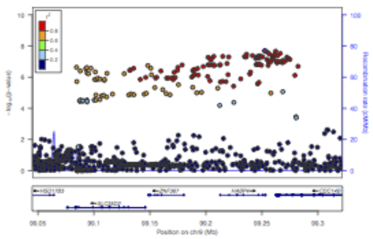

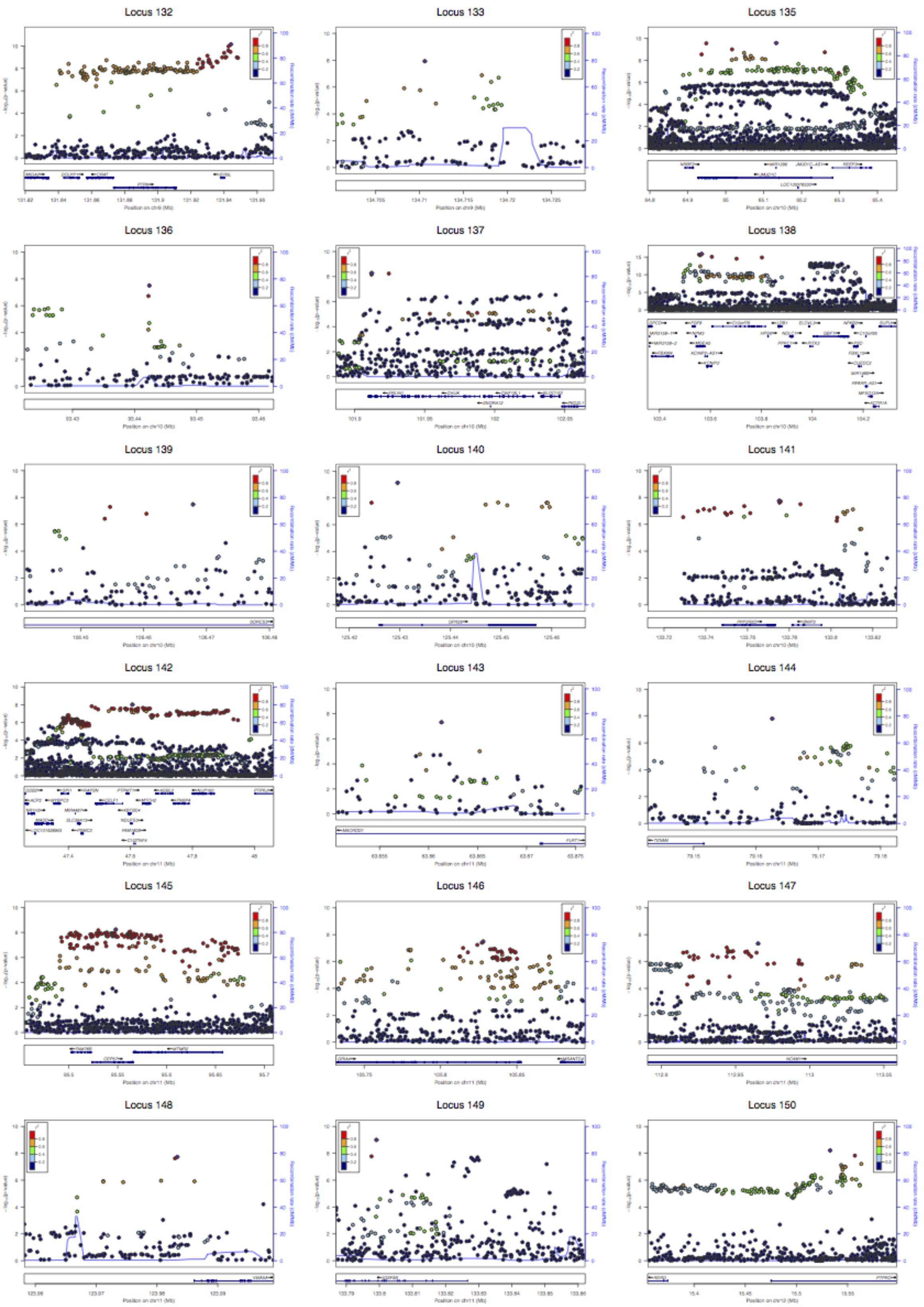
Locus 132
Locus 133
Locus 135
Locus 136
Locus 137
Locus 138
Locus 139
Locus 140
Locus 141
Locus 142
Locus 143
Locus 144
Locus 145
Locus 146
Locus 147
Locus 148
Locus 149
Locus 150

Locus 151

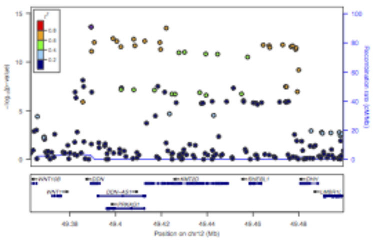

Locus 152

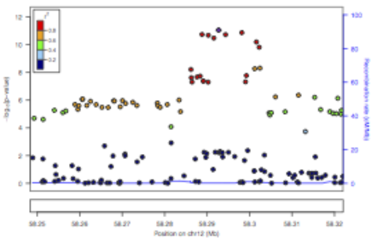

Locus 153

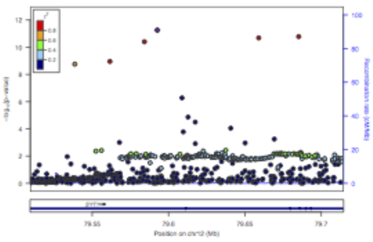

Locus 154

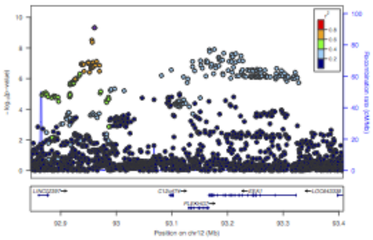

Locus 155

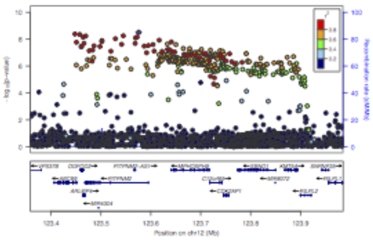

Locus 156

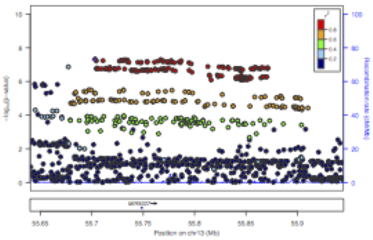

Locus 157

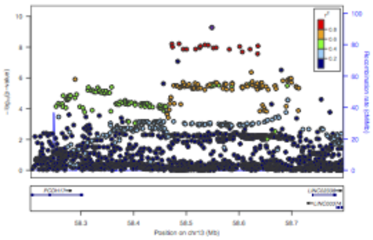

Locus 158

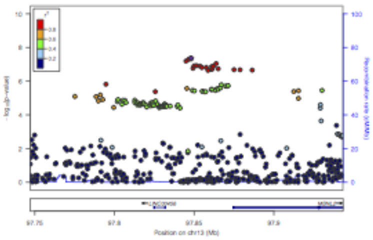

Locus 159

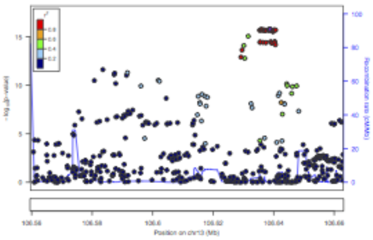

Locus 160

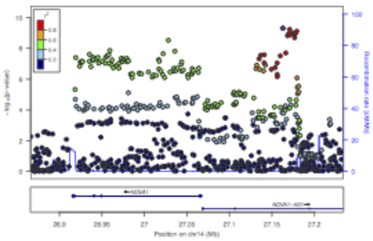

Locus 161

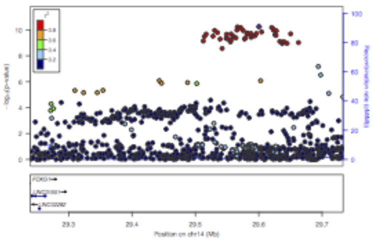

Locus 162

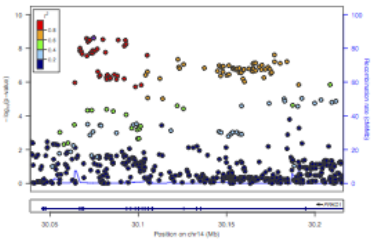

Locus 163

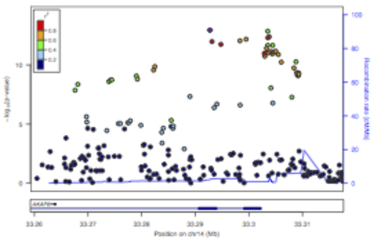

Locus 164

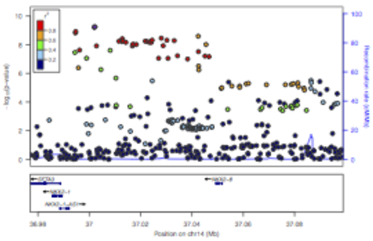

Locus 165

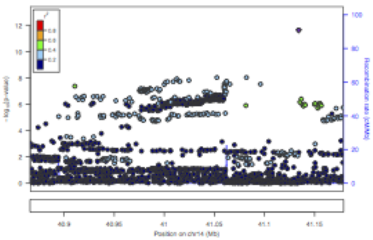

Locus 166

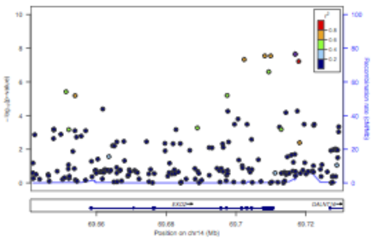

Locus 167

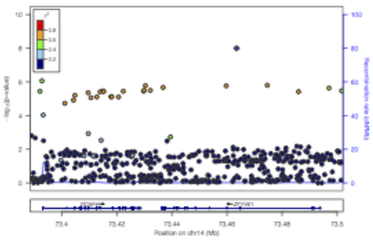

Locus 168

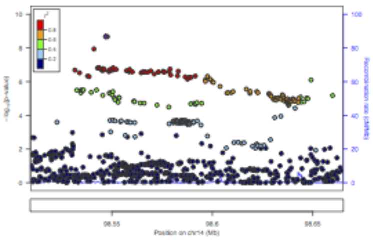

Locus 169

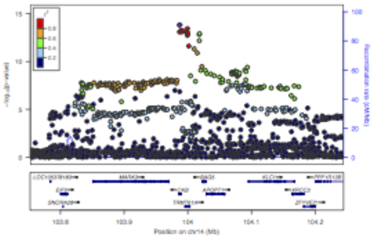

Locus 170

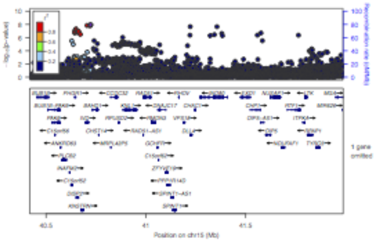

Locus 171

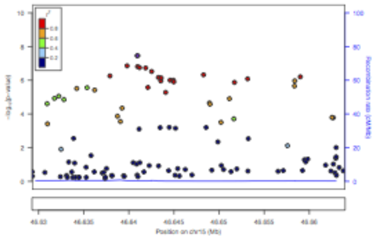

Locus 172

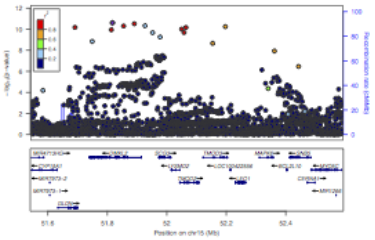

Locus 173

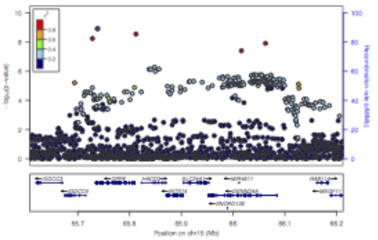

Locus 174

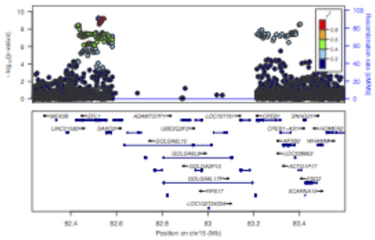

Locus 175

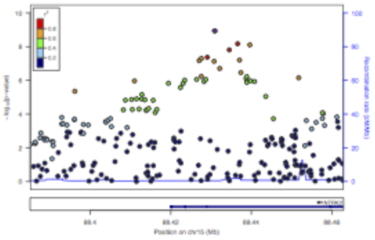

Locus 176

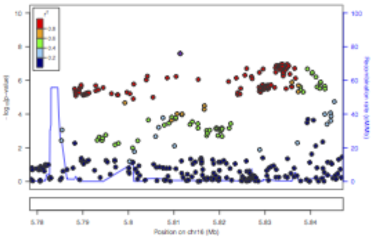

Locus 177

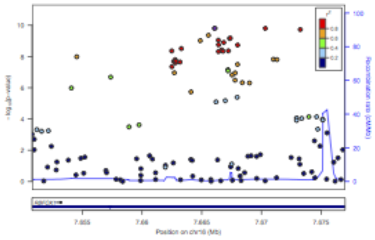

Locus 178

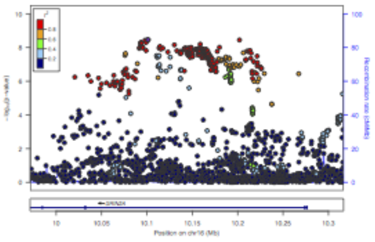

Locus 179

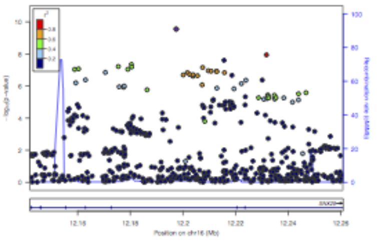

Locus 180

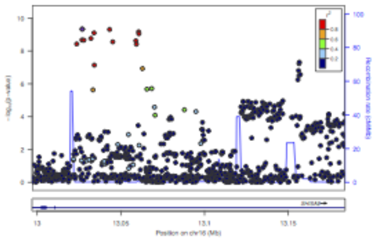

Locus 181

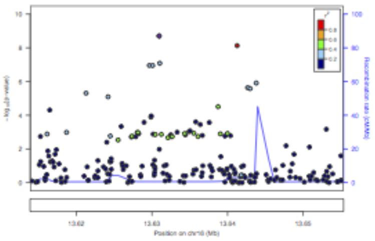

Locus 182

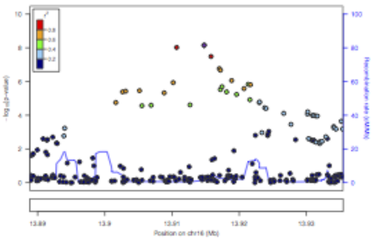

Locus 183

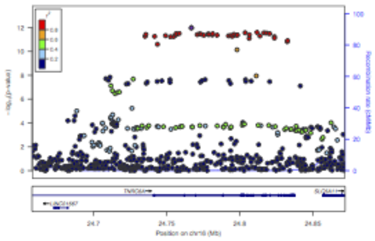

Locus 184

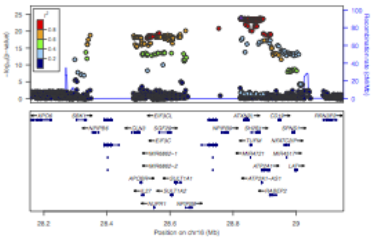

Locus 185

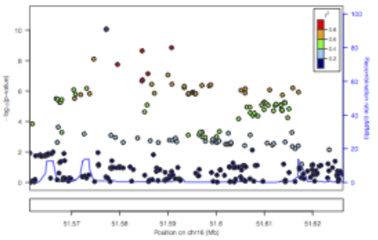

Locus 186

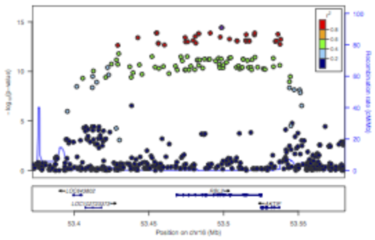

Locus 187

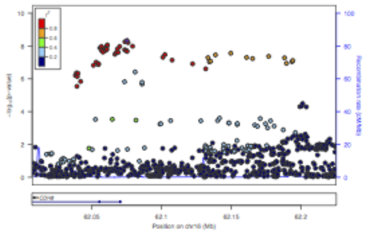

Locus 188

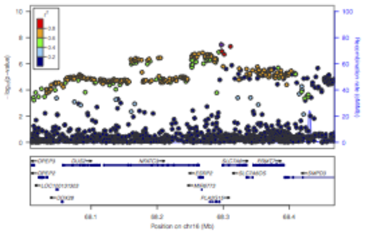

Locus 189

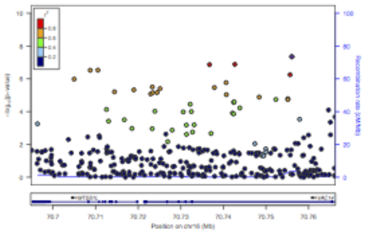

Locus 190

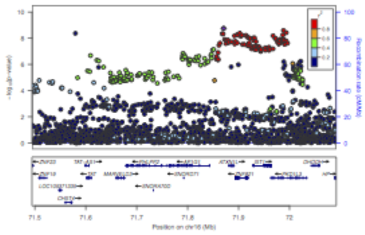

Locus 191

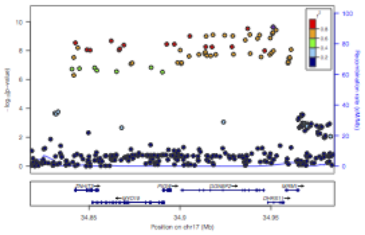

Locus 192

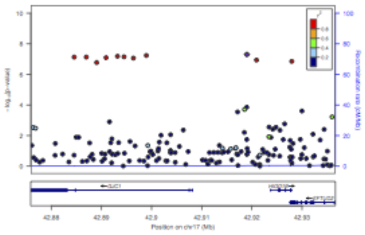

Locus 193

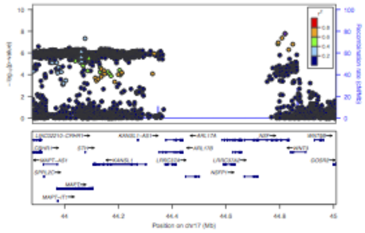

Locus 194

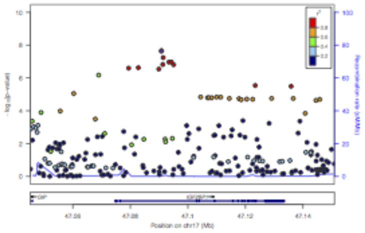

Locus 195

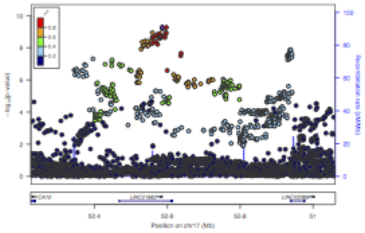

Locus 196

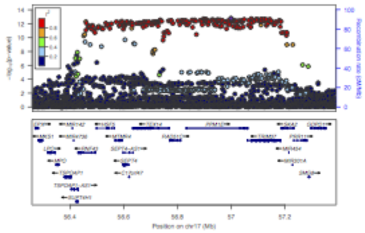

Locus 198

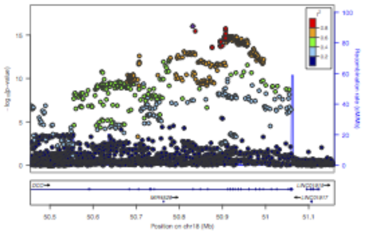

Locus 199

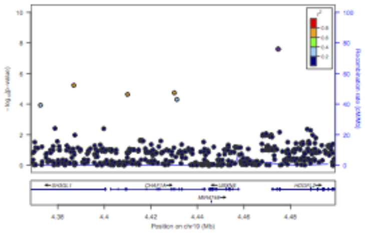

Locus 201

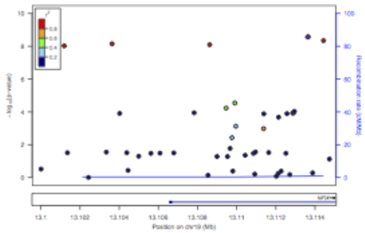

Locus 202

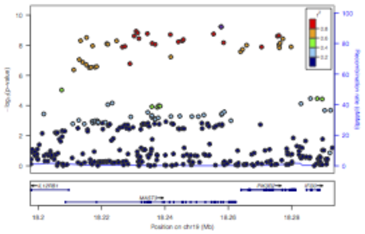

Locus 203

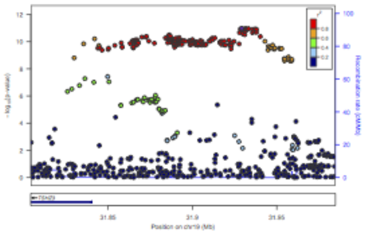

Locus 204

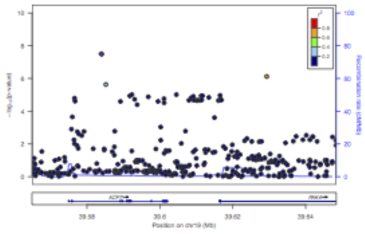

Locus 205

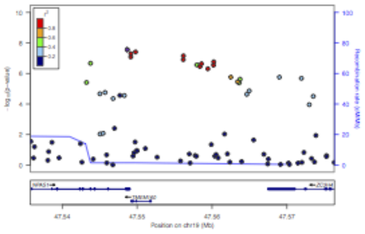

Locus 206

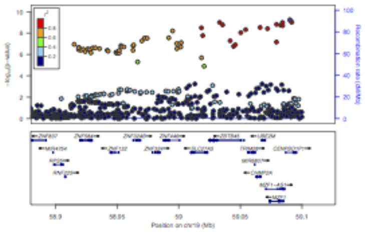

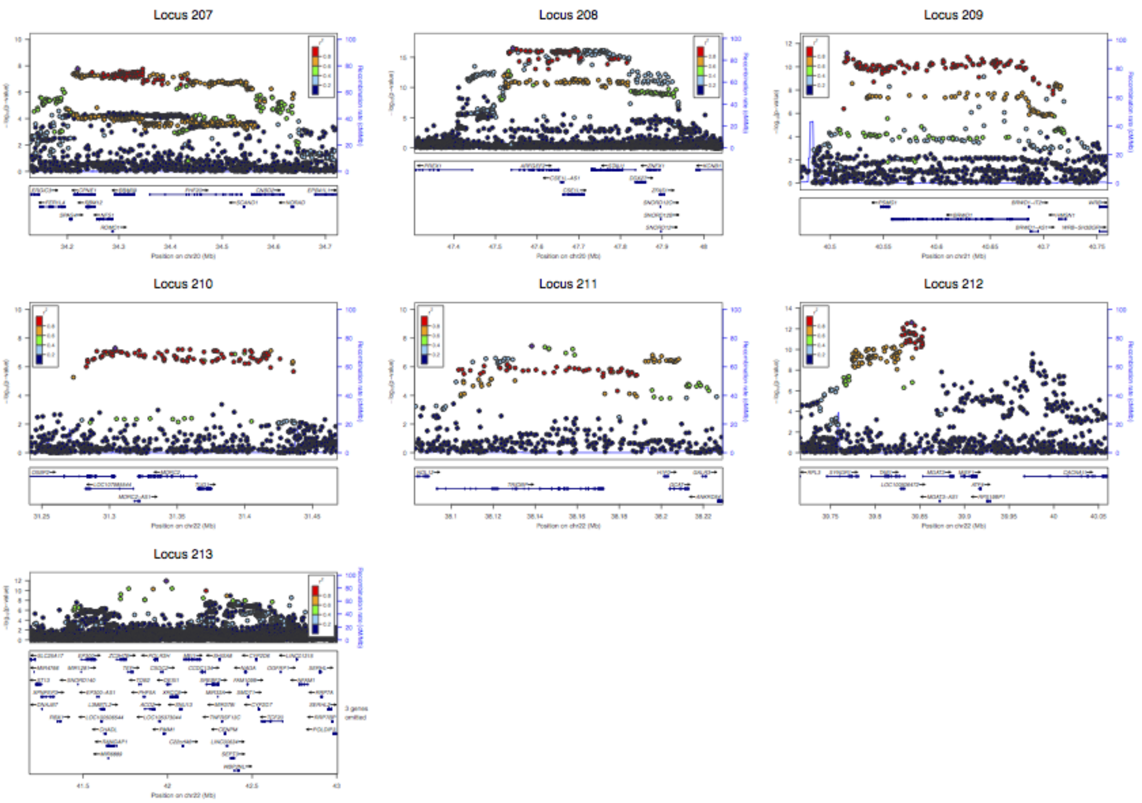

Supplementary Figure 4. Regional association plots of genomic risk loci identified in a GWAS meta-analysis of intelligence in 269,867 independent individuals. Distinct genomic risk loci were defined as LD-independent regions (r^2<0.1) separated by 250 kb and containing one or more SNPs with a Bonferroni-corrected genome-wide significant association (two-tailed P<5×10^{-8}) with intelligence in a meta-analysis of linear and logistic regression statistics. For each locus, the regional association plot shows the –log10 transformed two-tailed *P*-value of each SNP from the meta-analysis on the y-axis and base pair positions along the chromosomes on the x-axis. Genes overlapping the locus are displayed below the plot. Independent lead SNPs are indicated by a purple diamond, and each SNP is colored by its LD value with the lead SNP in the region.

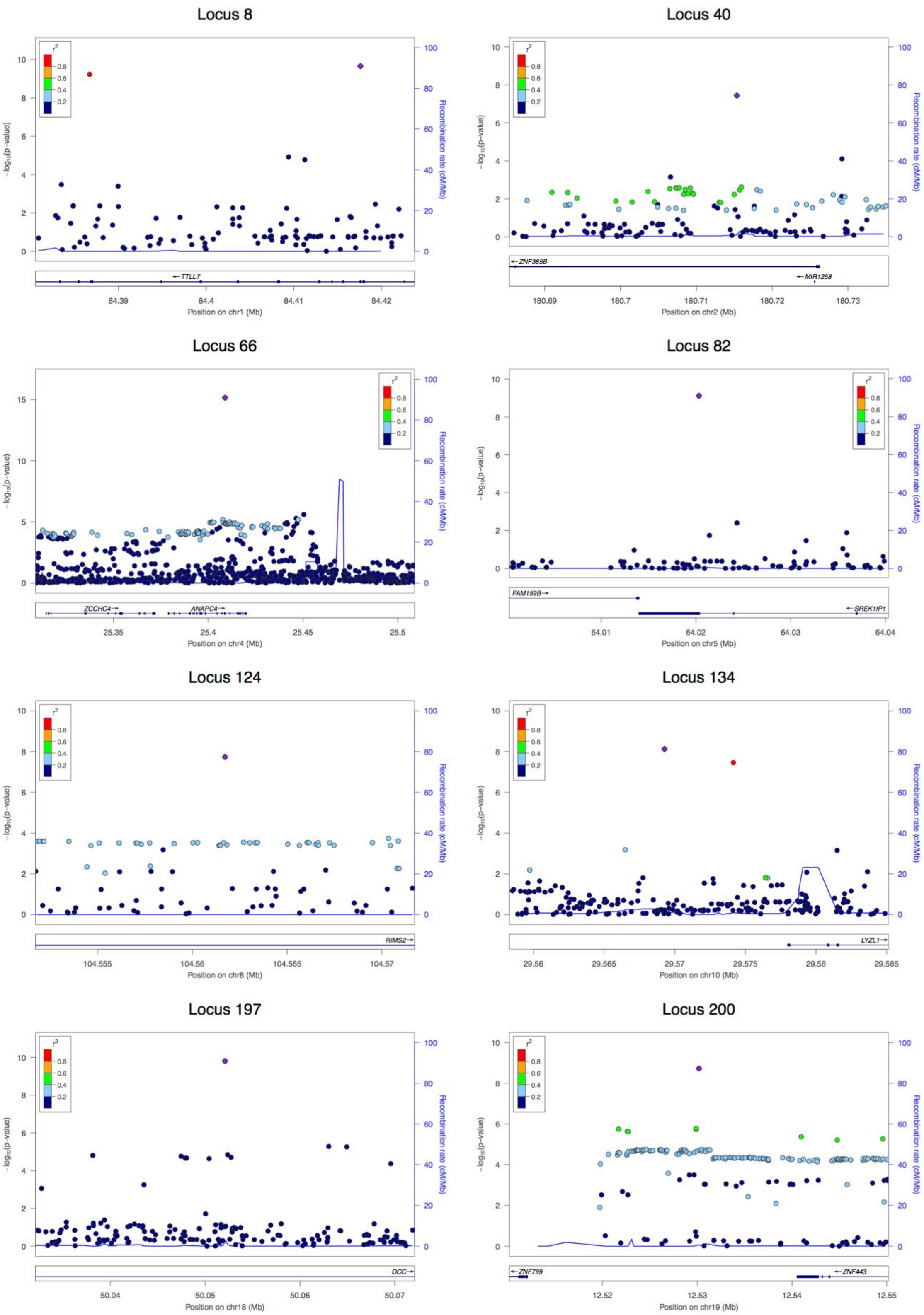

Supplementary Figure 5. Regional association plots for eight genome-wide significant loci with suspicious patterns of linkage disequilibrium in a GWAS meta-analysis of intelligence in

269,867 independent individuals. Distinct genomic risk loci were defined as LD-independent regions (r^2<0.1) separated by 250 kb and containing one or more SNPs with a Bonferroni-corrected genome-wide significant association (two-tailed $P<5\times10^{-8}$) with intelligence in a meta-analysis of linear and logistic regression statistics. For each locus, the regional association plot shows the –log10 transformed two-tailed *P*-value of each SNP from the meta-analysis on the y-axis and base pair positions along the chromosomes on the x-axis. Genes overlapping the locus are displayed below the plot. Independent lead SNPs are indicated by a purple diamond, and each SNP is colored by its LD value with the lead SNP in the region**.** Lead SNPs were isolated GWS signals in a region; these did not show obvious quality issues but were excluded from the number of reported risk loci (**Supplementary Results 2.3.1**).

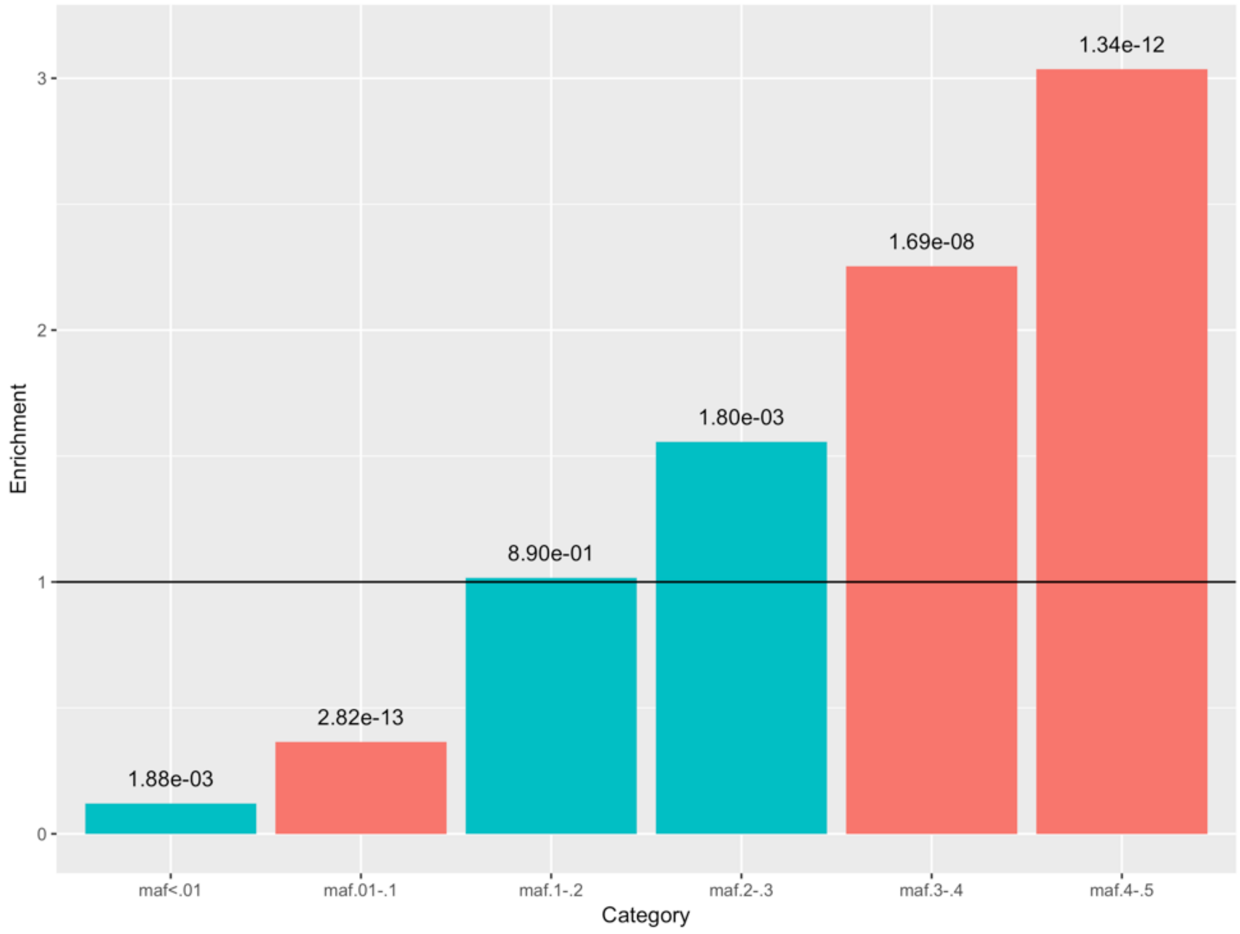

Supplementary Figure 6. Heritability of intelligence in a GWAS meta-analysis of 269,867 independent individuals, stratified by minor allele frequency (MAF) bins. Tests of enrichment/depletion of SNP heritability within a bin relative to the proportion of SNPs in the bin were conducted with LD score regression. Two-tailed *P*-values are presented above the bins; red bins are significant after Bonferroni correction for 56 total strata. Horizontal line (=1.0) indicates no enrichment.

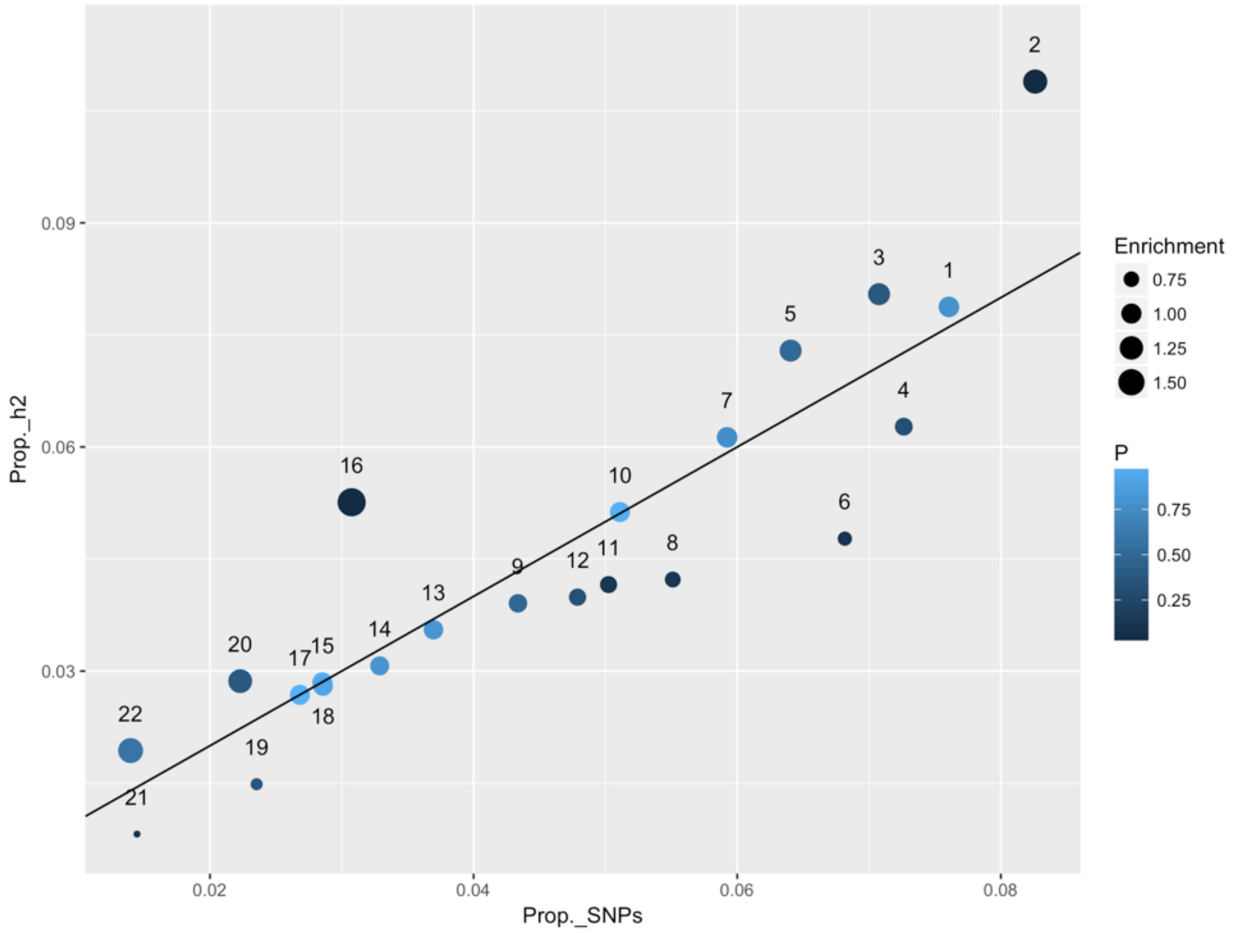

Supplementary Figure 7. Heritability of intelligence in a GWAS meta-analysis of 269,867 independent individuals, stratified by chromosome. Tests of enrichment/depletion of SNP heritability within a chromosome relative to the proportion of SNPs on the chromosome were conducted with LD score regression. The chromosome size (proportion of total SNPs) is on the x-axis and the proportion of total heritability (h^2) attributable to each chromosome is on the y-axis. The X chromosome was not available in the reference panel for LD scores. Chromosomes are colored by two-tailed *P*-values; none were significant after Bonferroni correction for 56 total strata. Diagonal line indicates no enrichment.

Chromosome 1

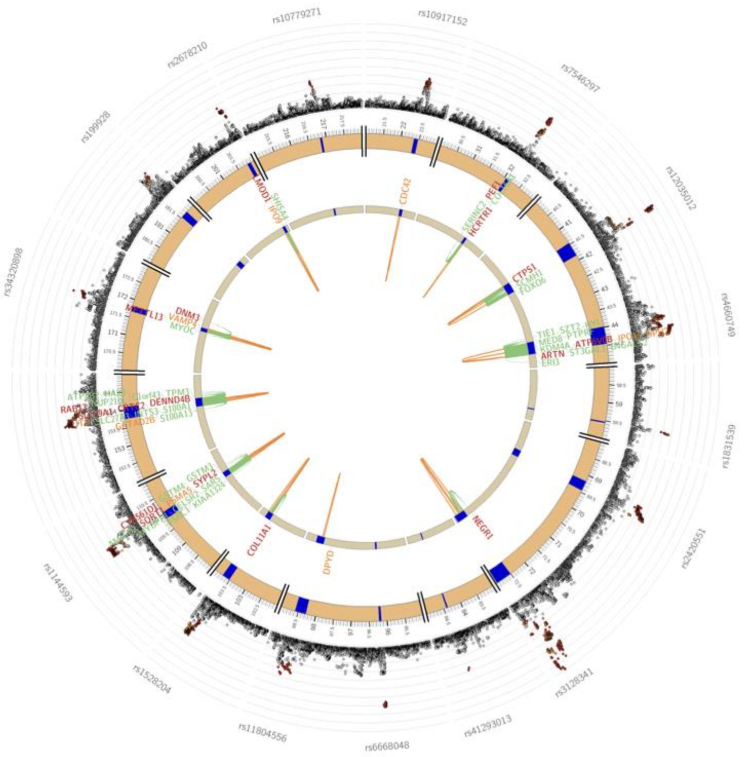

Chromosome 2

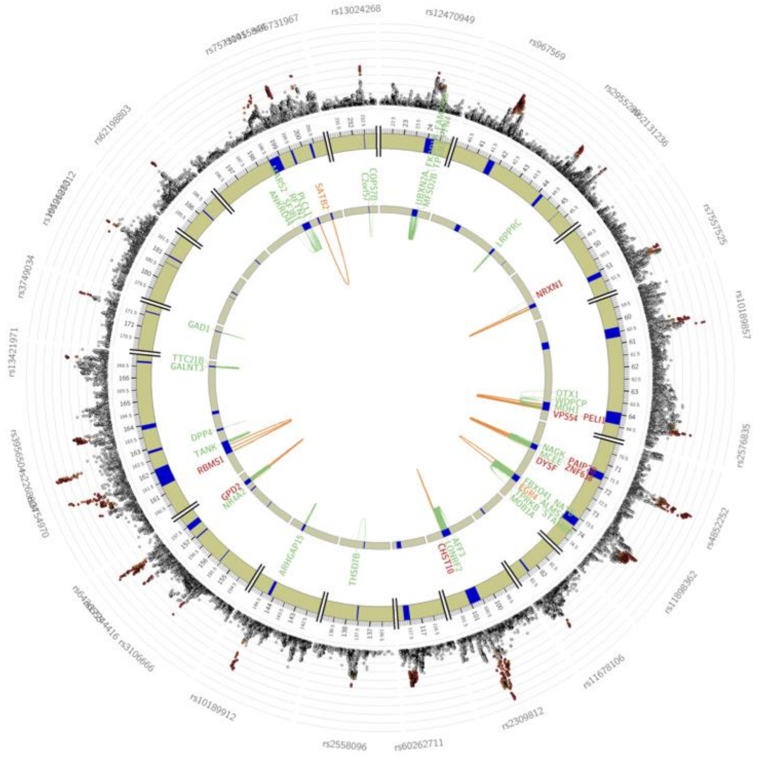

Chromosome 3

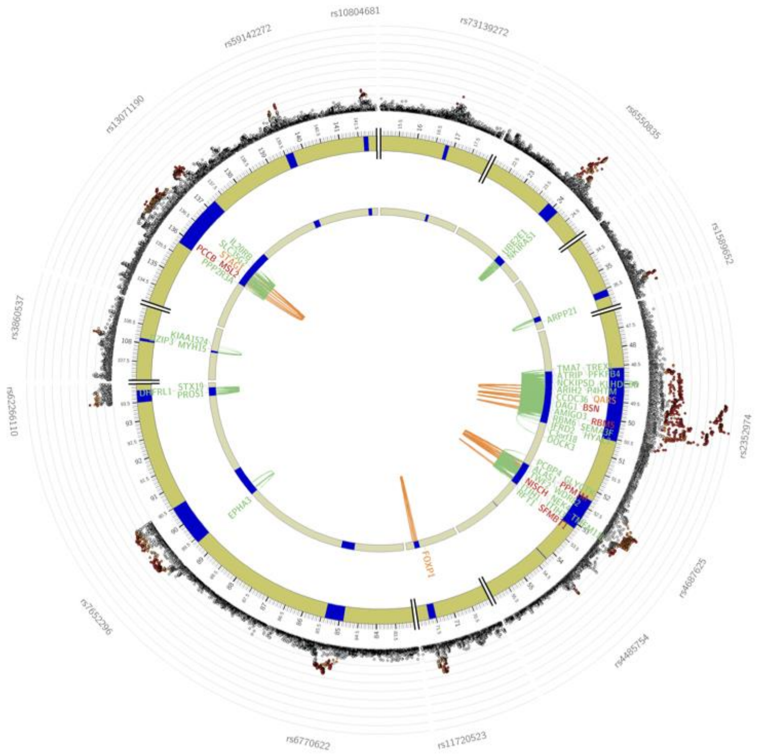

Chromosome 4

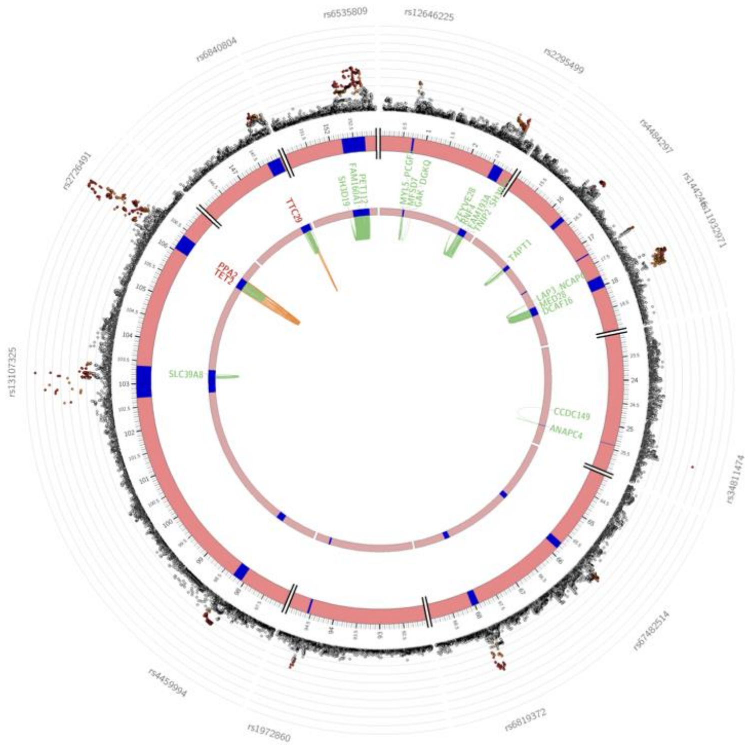

Chromosome 5

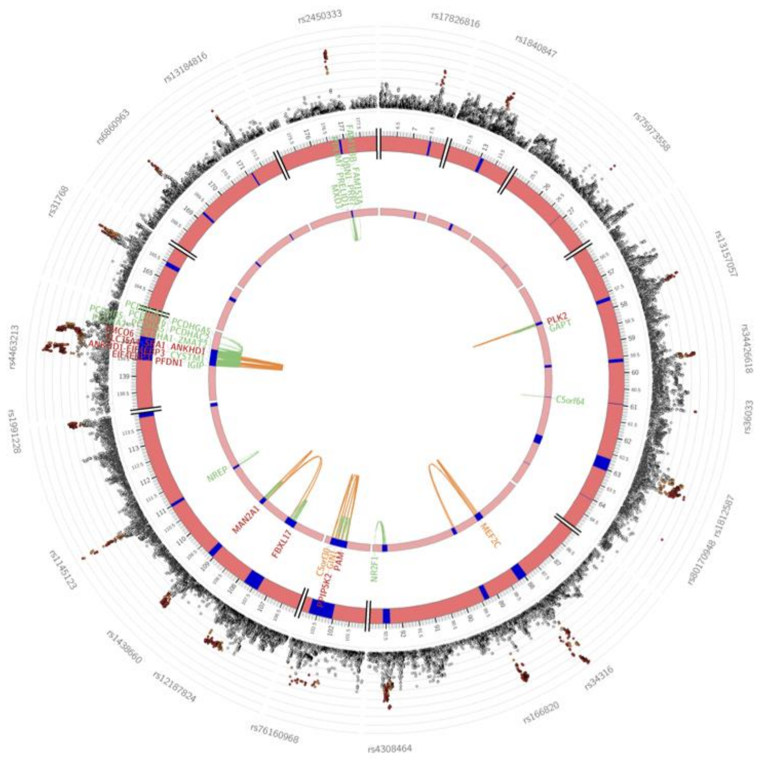

Chromosome 6

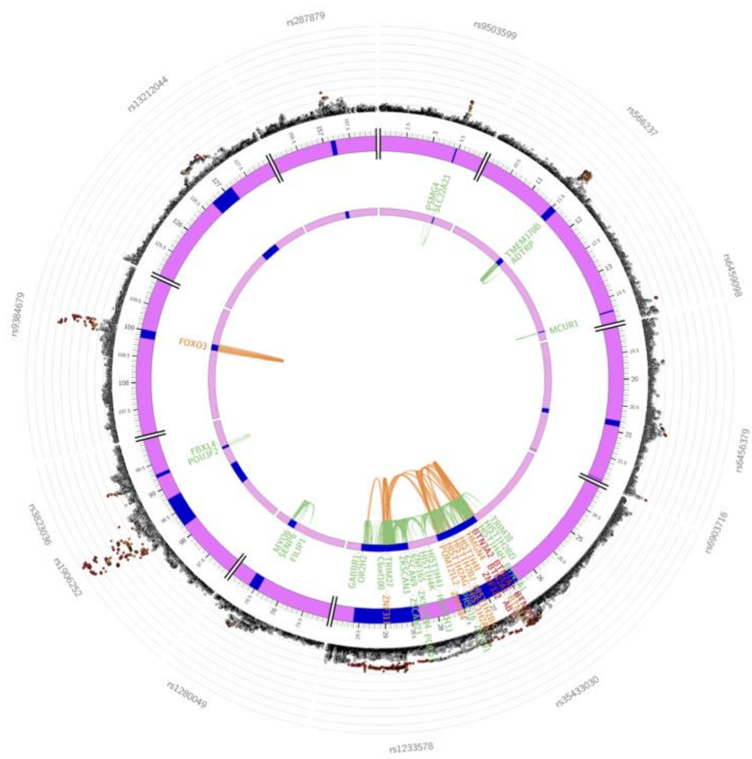

Chromosome 7

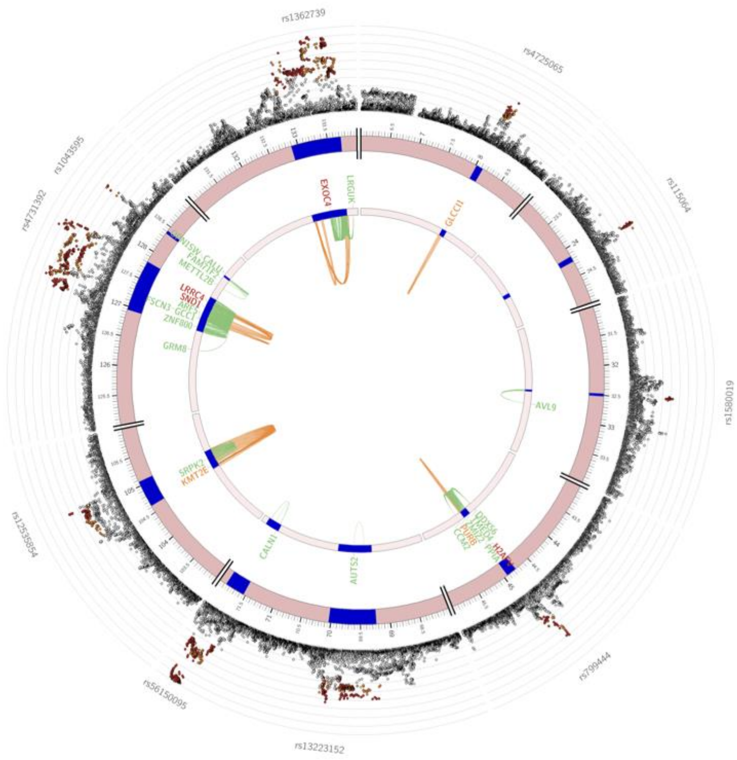

Chromosome 8

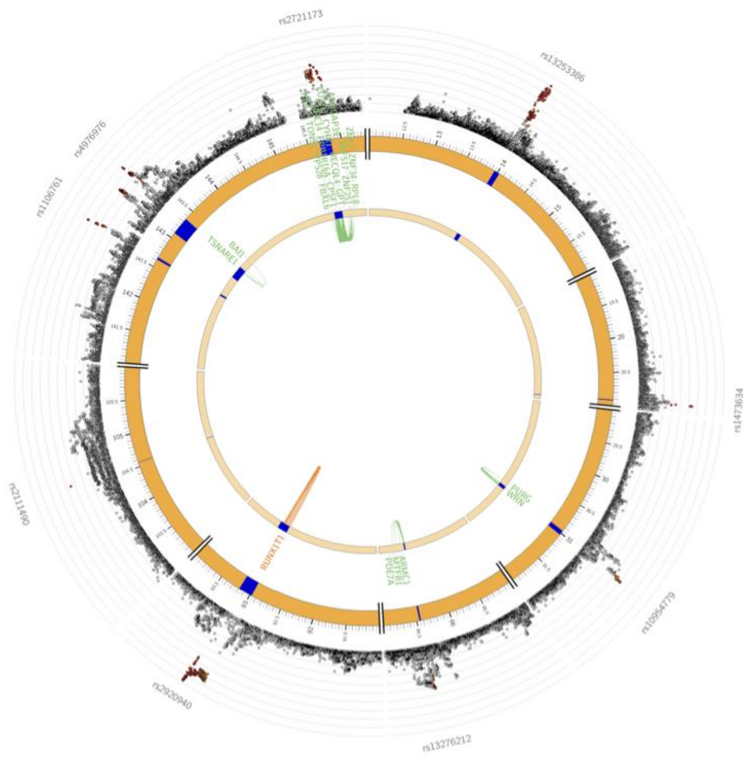

Chromosome 9

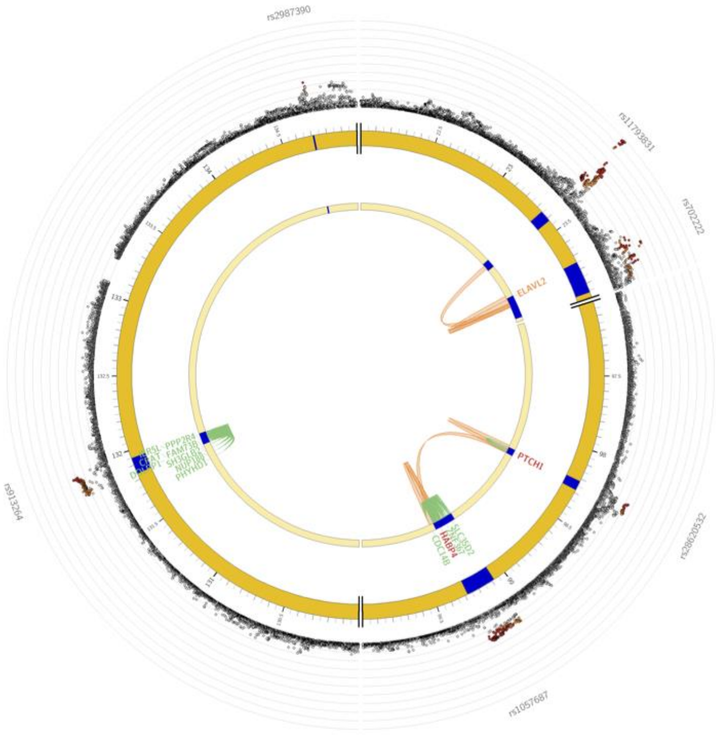

Chromosome 10

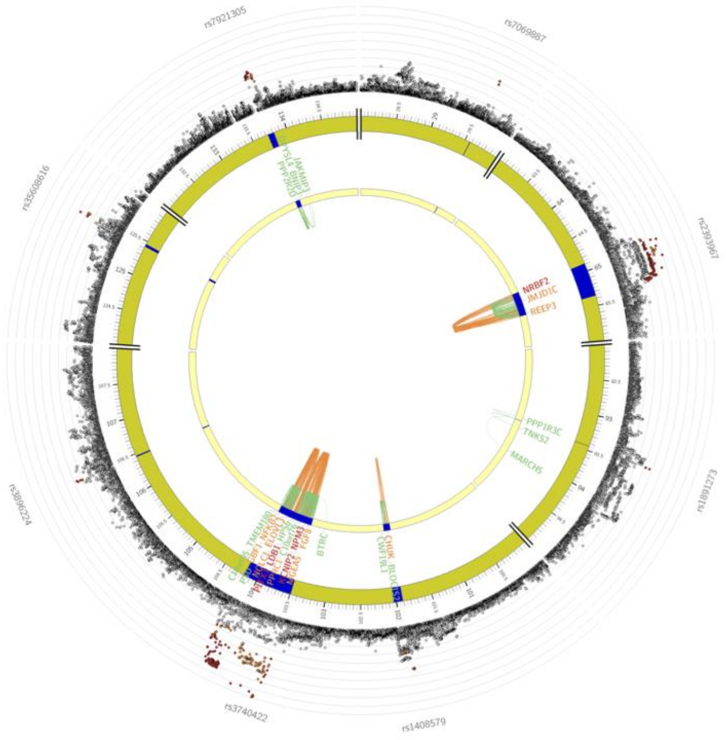

Chromosome 11

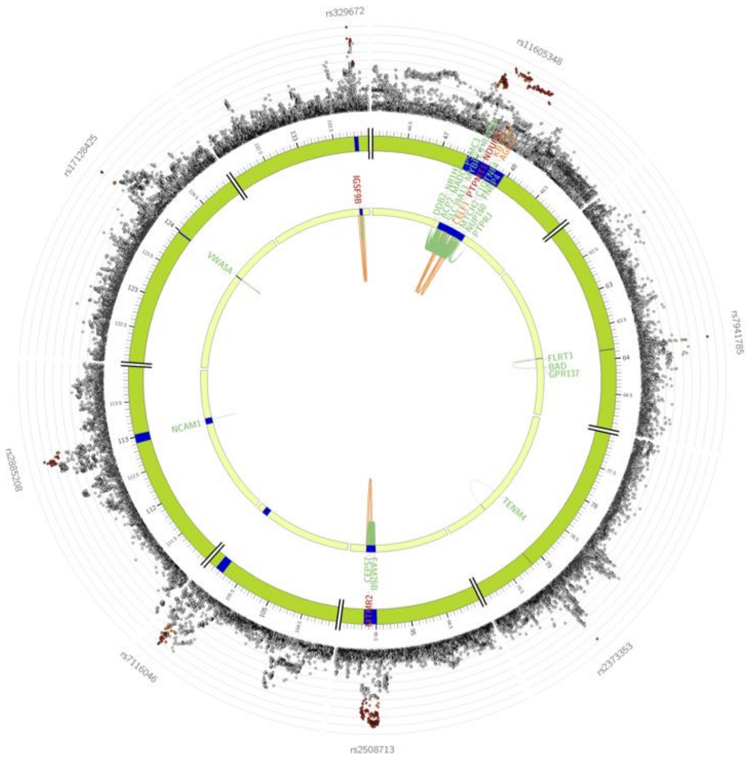

Chromosome 12

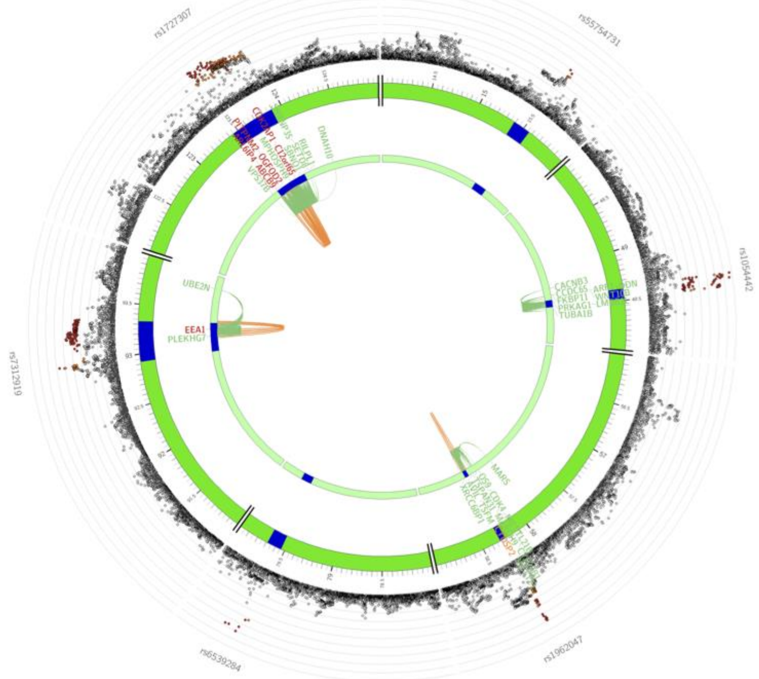

Chromosome 13

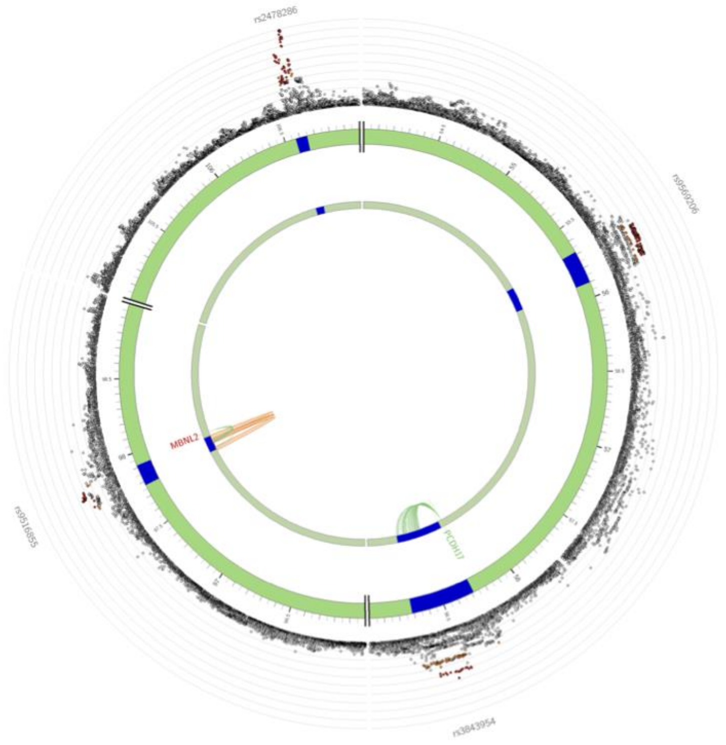

Chromosome 14

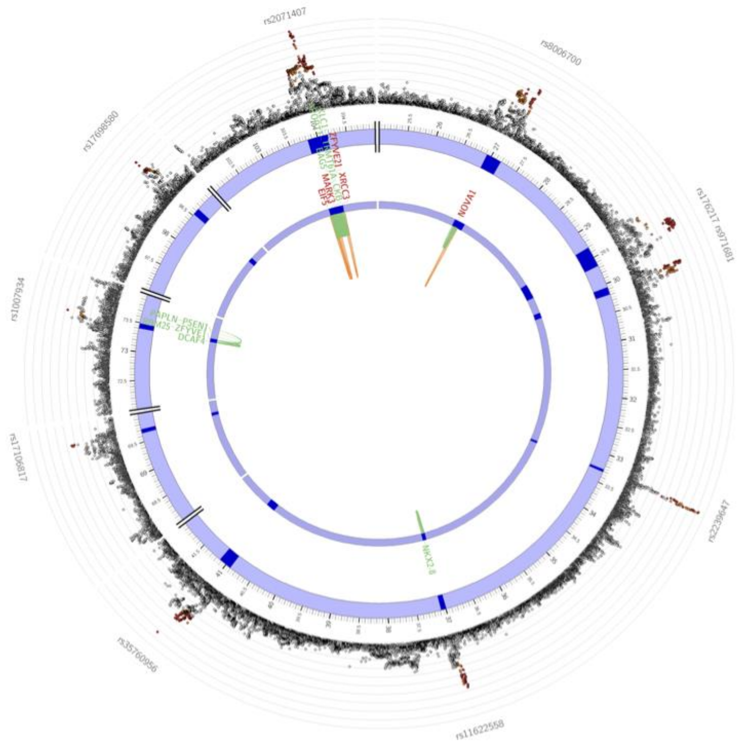

Chromosome 15

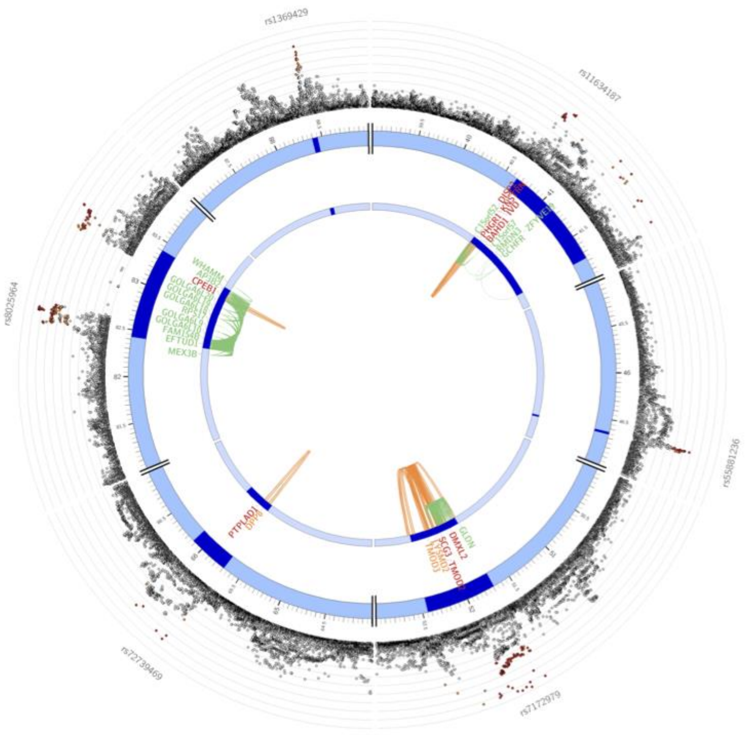

Chromosome 16

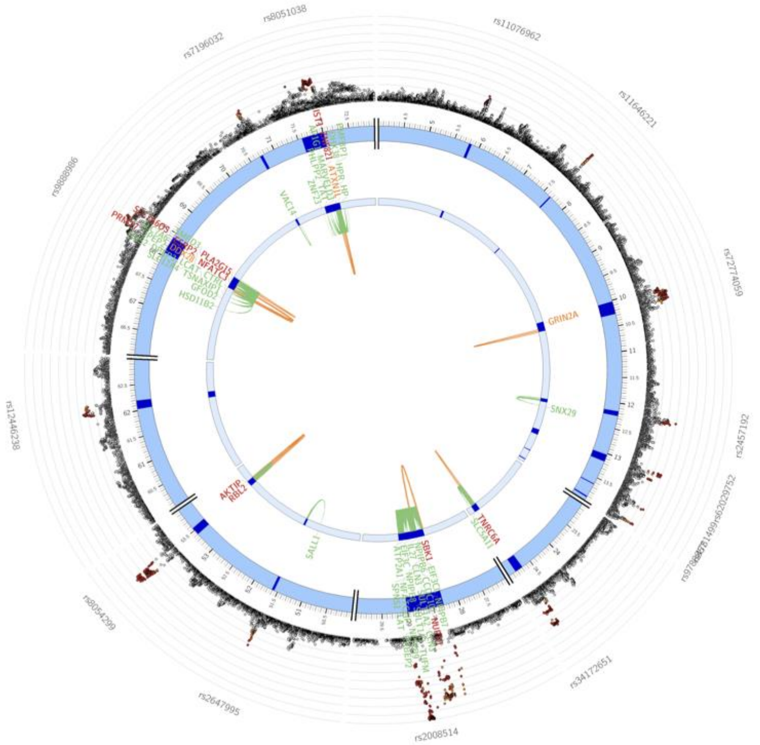

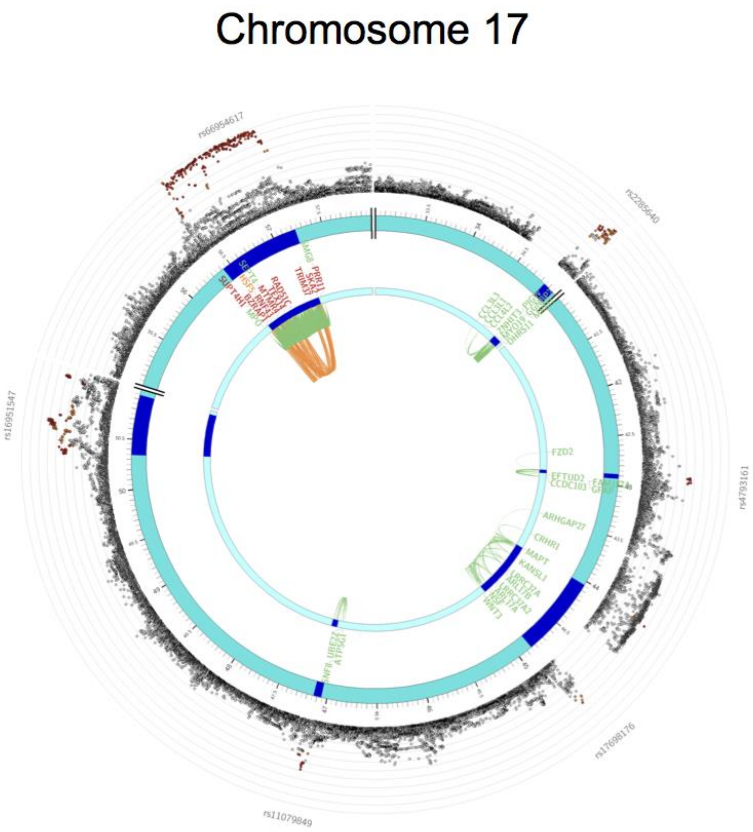
Chromosome 17
rs66954617
rs2285640
rs16951547
rs4793161
rs17698176
rs11079849

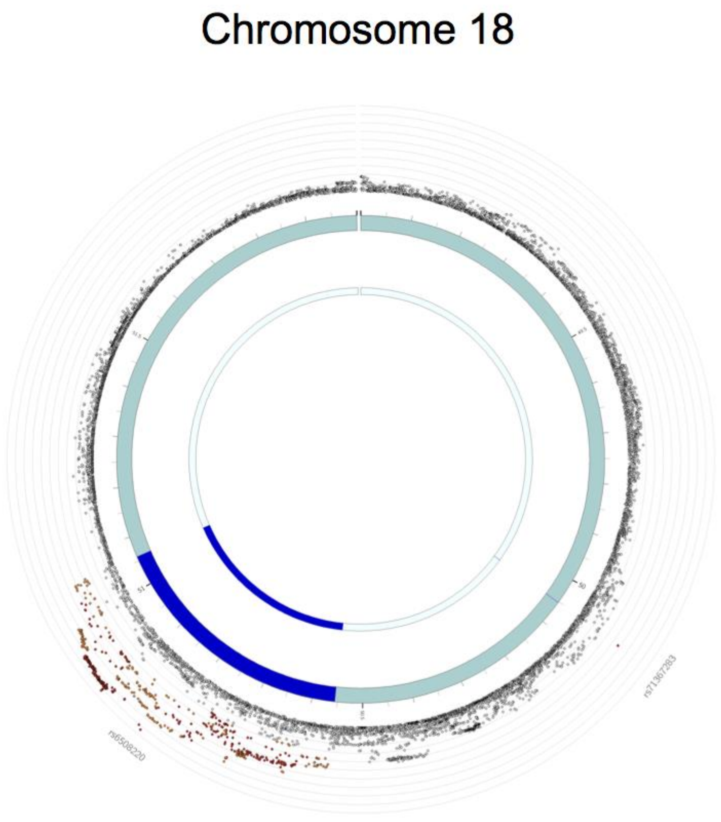
Chromosome 18
rs6508220
rs71367283

Chromosome 19

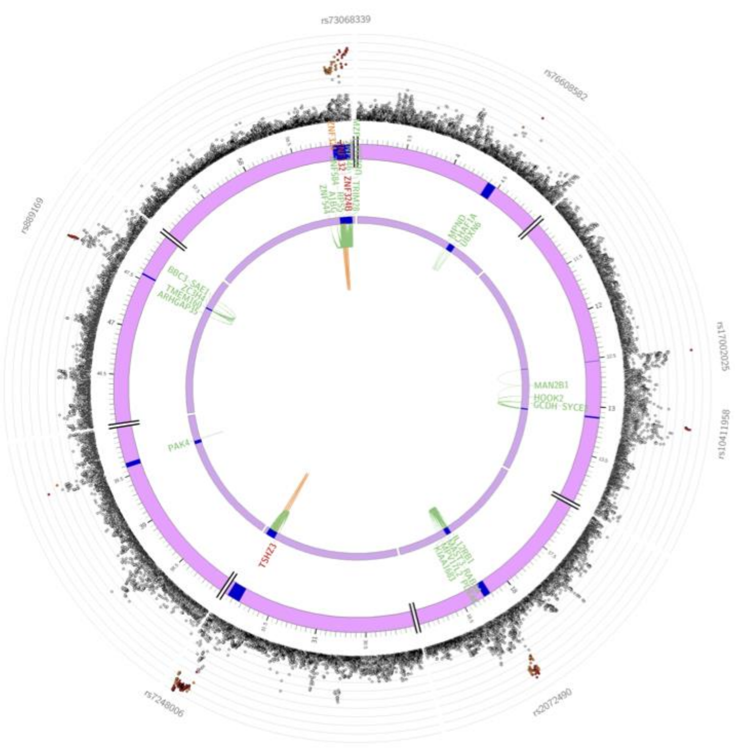

Chromosome 20

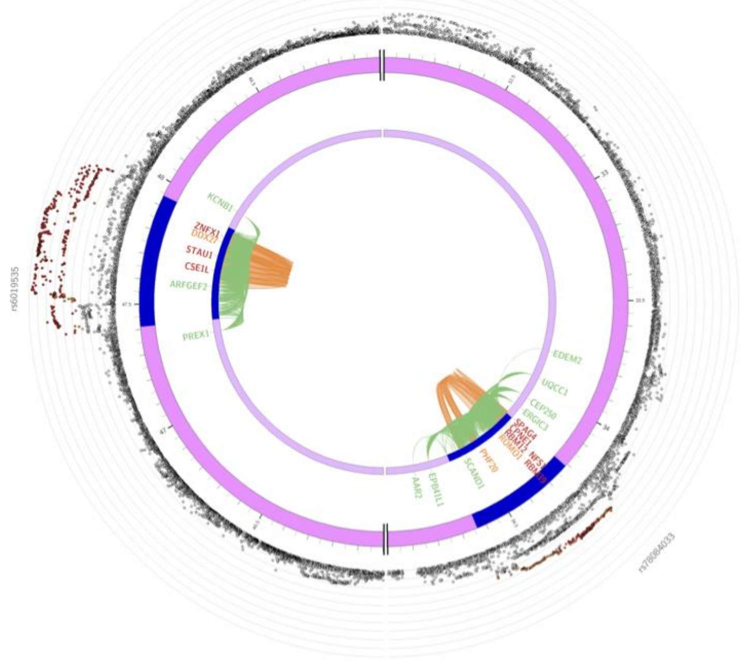

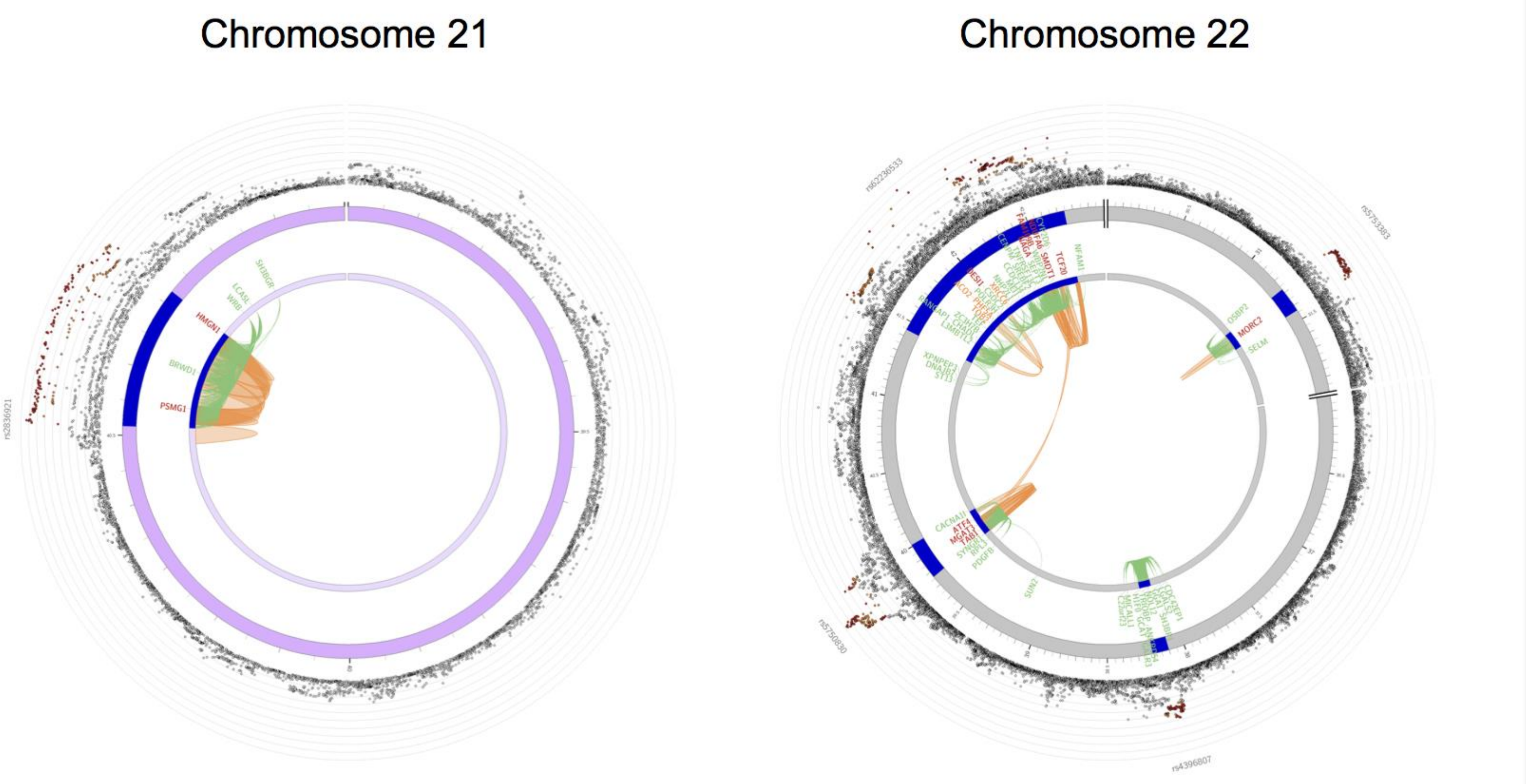

Supplementary Figure 8. Genomic risk loci, eQTL associations and chromatin interactions identified in a GWAS meta-analysis of intelligence in 269,867 independent individuals. For each chromosome, circos plots show genes that were implicated from genomic risk loci (blue regions) in the SNP meta-analysis by eQTL mapping (green lines connecting an eQTL SNP to its associated gene), and/or chromatin interaction (orange lines connecting two interacting regions). Genes implicated by both eQTL and chromatin interactions mapping are in red. The outer layer shows a Manhattan plot containing the –log10 transformed two-tailed *P*-value of each SNP in the GWAS meta-analysis (of linear and logistic association statistics), with genome-wide significant SNPs colored by their linkage disequilibrium patterns with the lead SNP.

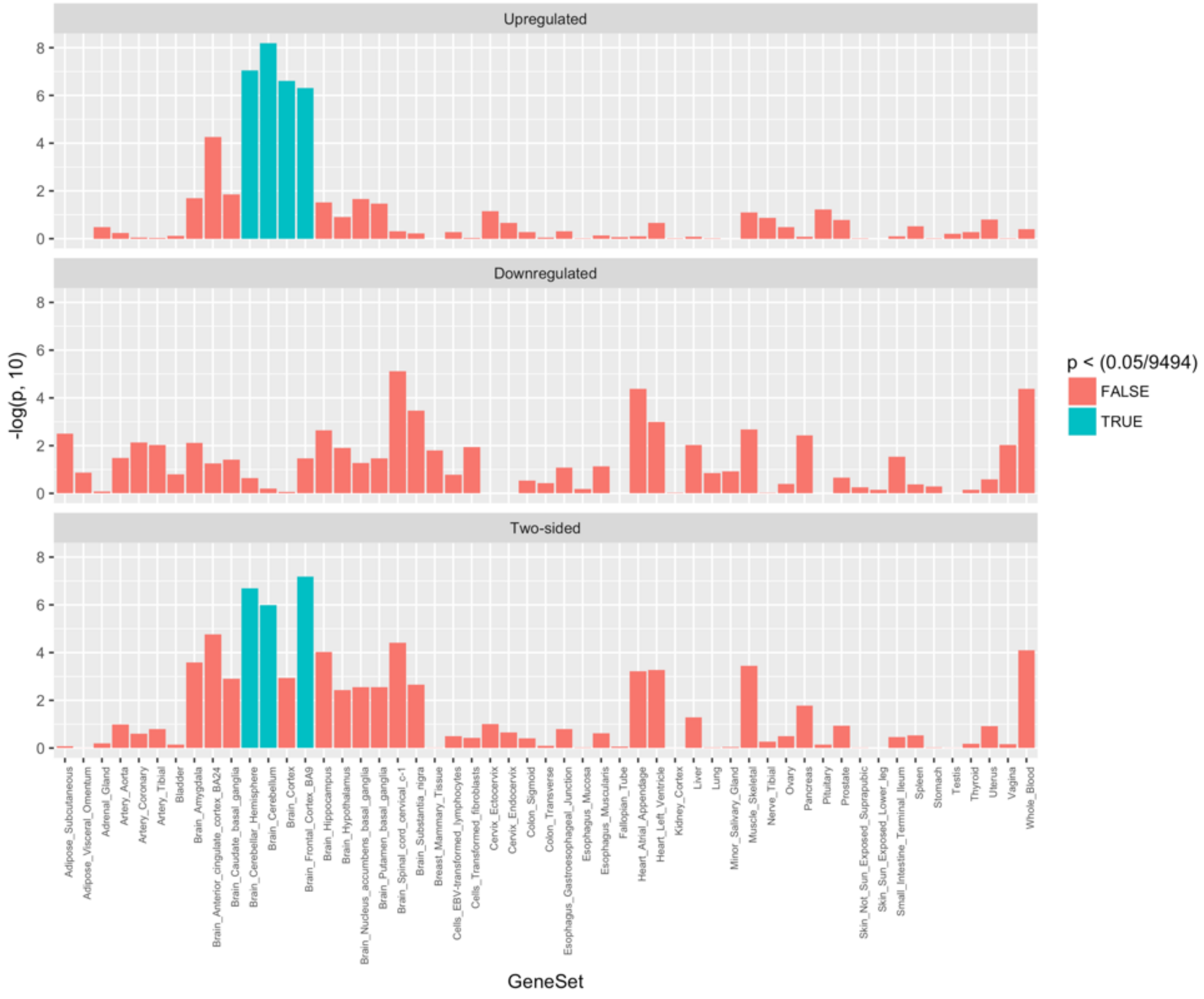

Supplementary Figure 9. Tissue enrichment for differential gene expression in genes associated with intelligence in a GWAS meta-analysis of 269,867 independent individuals. Hypergeometric tests of enrichment were conducted for 53 GTEx tissue types for 872 genes implicated by positional, eQTL, or chromatin interaction mapping of GWS SNPs in LD ($r^2 \geqq 0.6$) with one of the independent GWS SNPs. Enrichment difference are shown for higher (Upregulated), lower (Downregulated), or two-sided differences in gene expression. The tests do not include genes implicated only by GWGAS. Two-tailed *P*-values are presented; categories in blue are significant after Bonferroni correction for 9,494 total gene-sets.

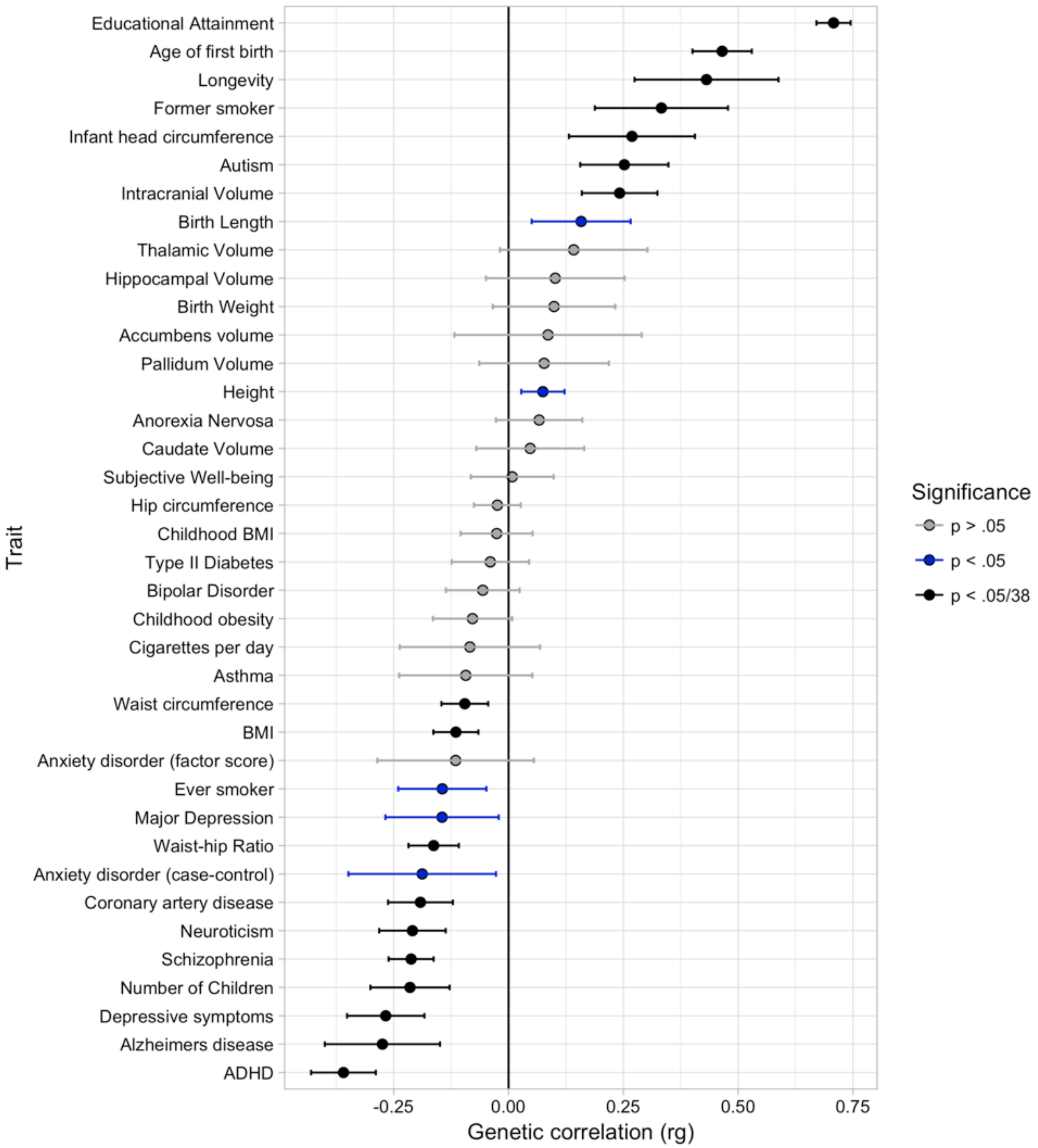

Supplementary Figure 10. Genetic correlations between intelligence and other traits previously investigated with GWAS. Correlations were calculated with LD score regression using SNP summary statistics from the GWAS meta-analysis of intelligence in 269,867 individuals and publically available summary statistics for other traits (**Supplementary Table 21**). Point estimates for correlations and 95% confidence intervals are shown; black dots indicate significant two-tailed *P*-values after Bonferroni correction for 38 pairs of traits.

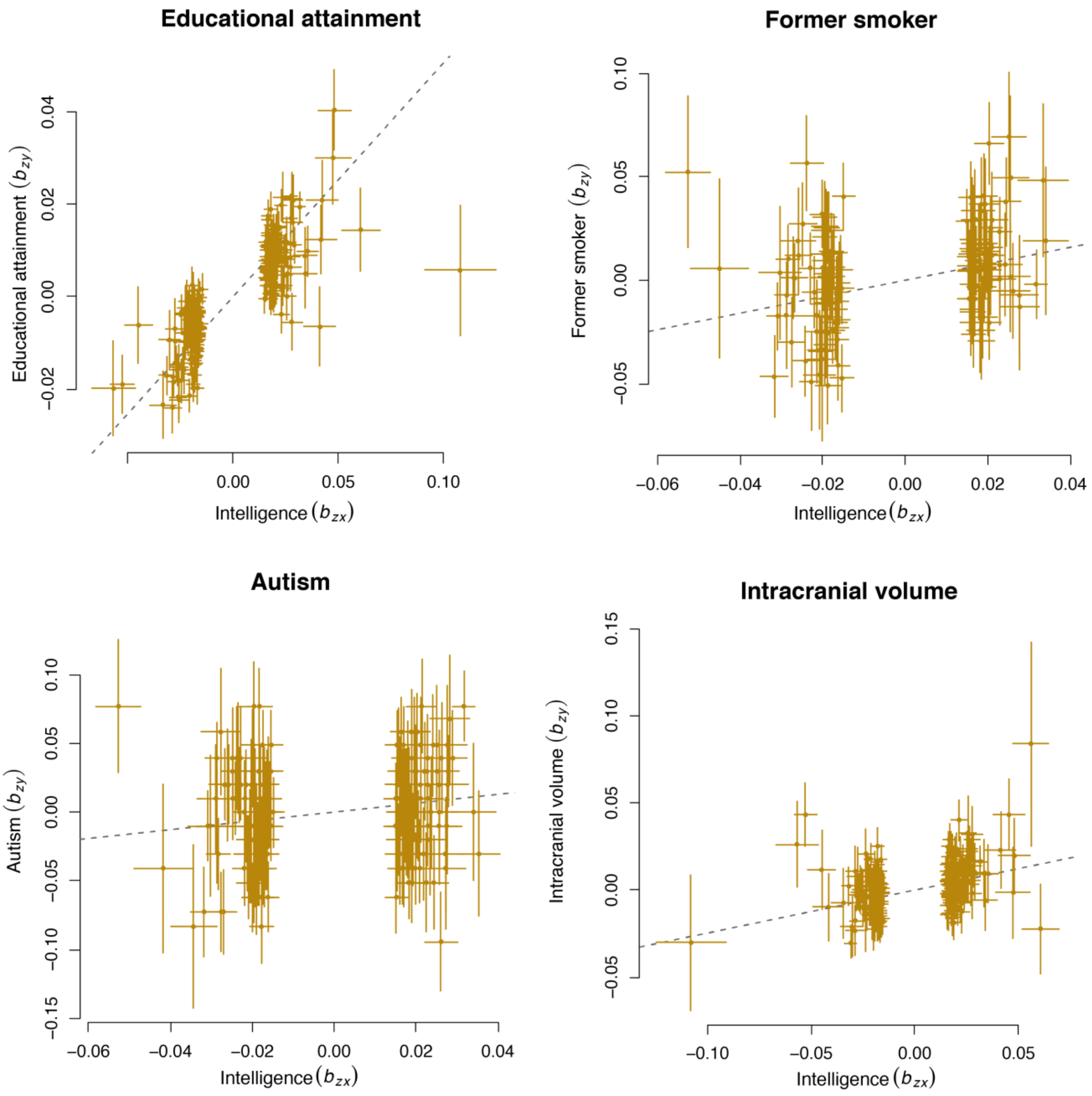
Educational attainment
Educational attainment (b_{zy})
−0.02
0.00
0.02
0.04
0.00
0.05
0.10
Intelligence (b_{zx})
Former smoker
Former smoker (b_{zy})
−0.05
0.00
0.05
0.10
−0.06
−0.04
−0.02
0.00
0.02
0.04
Intelligence (b_{zx})
Autism
Autism (b_{zy})
−0.15
−0.10
−0.05
0.00
0.05
0.10
−0.06
−0.04
−0.02
0.00
0.02
0.04
Intelligence (b_{zx})
Intracranial volume
Intracranial volume (b_{zy})
−0.05
0.00
0.05
0.10
0.15
−0.10
−0.05
0.00
0.05
Intelligence (b_{zx})

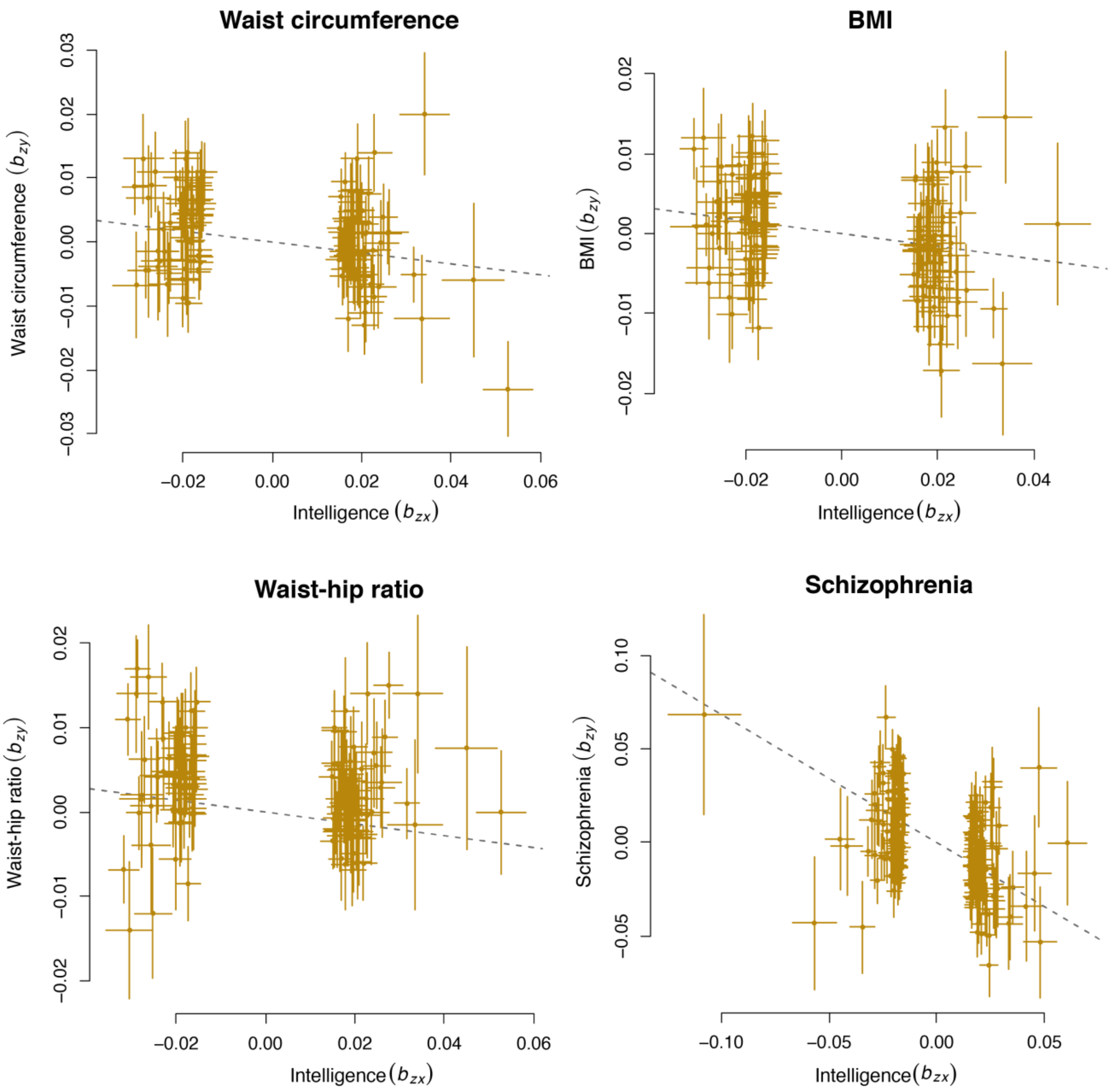
Waist circumference
Waist circumference (b_{zy})
0.03
0.02
0.01
0.00
−0.01
−0.02
−0.03
−0.02
0.00
0.02
0.04
0.06
Intelligence (b_{zx})
BMI
BMI (b_{zy})
0.02
0.01
0.00
−0.01
−0.02
−0.02
0.00
0.02
0.04
Intelligence (b_{zx})
Waist-hip ratio
Waist-hip ratio (b_{zy})
0.02
0.01
0.00
−0.01
−0.02
−0.02
0.00
0.02
0.04
0.06
Intelligence (b_{zx})
Schizophrenia
Schizophrenia (b_{zy})
0.10
0.05
0.00
−0.05
−0.10
−0.05
0.00
0.05
Intelligence (b_{zx})

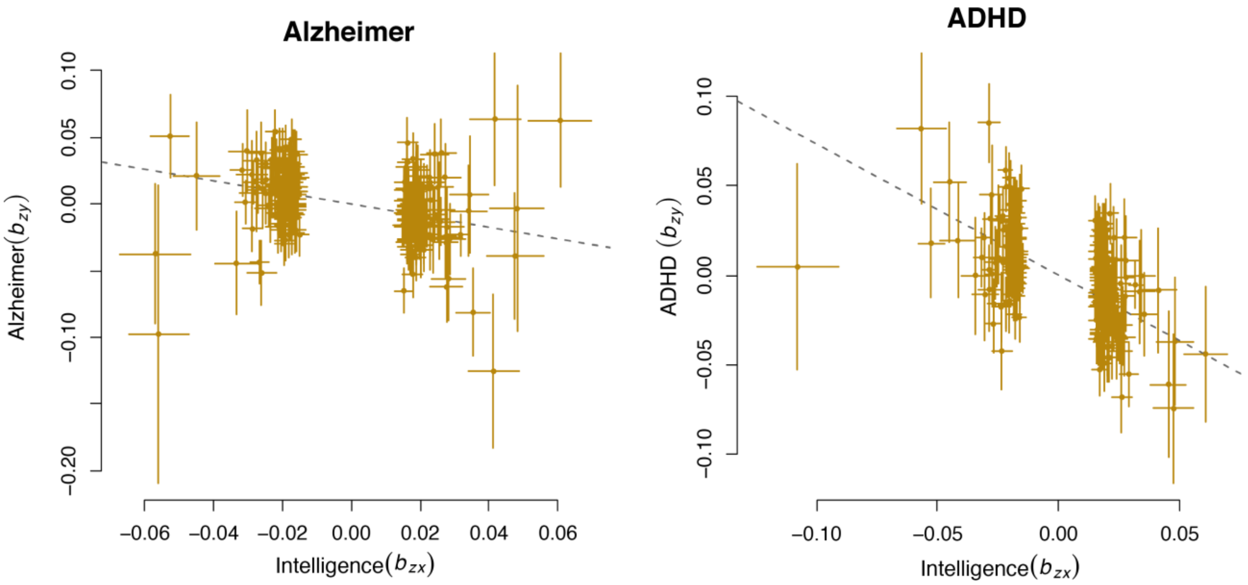

Supplementary Figure 11. Mendelian Randomization tests for the effect of intelligence on other correlated traits. Plots of effect sizes of independent lead SNPs from a GWAS meta-analysis of intelligence in 269,867 independent individuals (b_{zx}) on the x-axis and SNP GWAS effect sizes for correlated traits on the y-axis (b_{zx}). The dotted line represents a line with slope of (b_{xy}) and an intercept of 0. Error bars show 95% confidence intervals for the effect sizes for each trait.

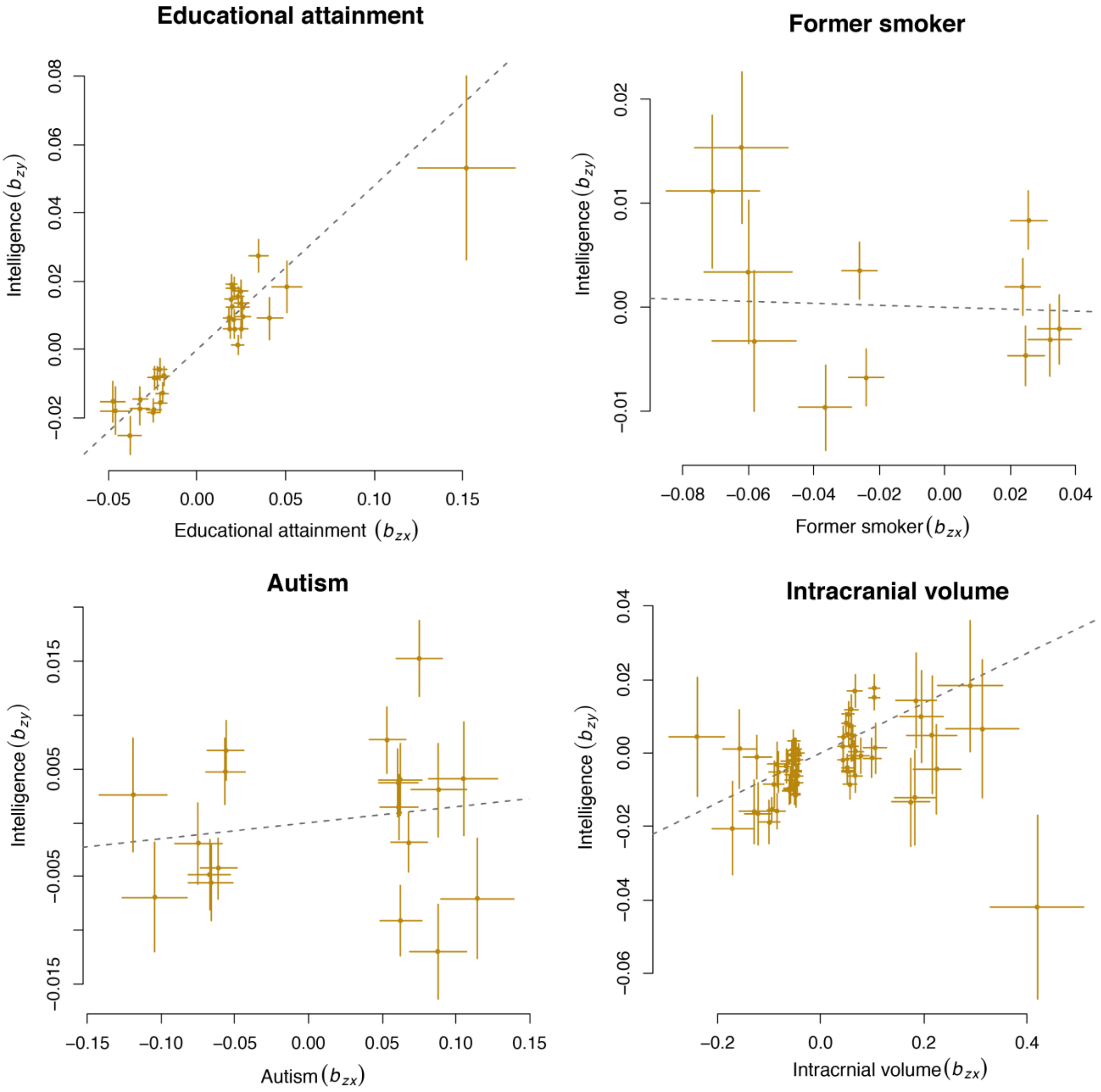
Educational attainment
Intelligence (b_{zy})
Educational attainment (b_{zx})
Former smoker
Intelligence (b_{zy})
Former smoker (b_{zx})
Autism
Intelligence (b_{zy})
Autism (b_{zx})
Intracranial volume
Intelligence (b_{zy})
Intracrnial volume (b_{zx})

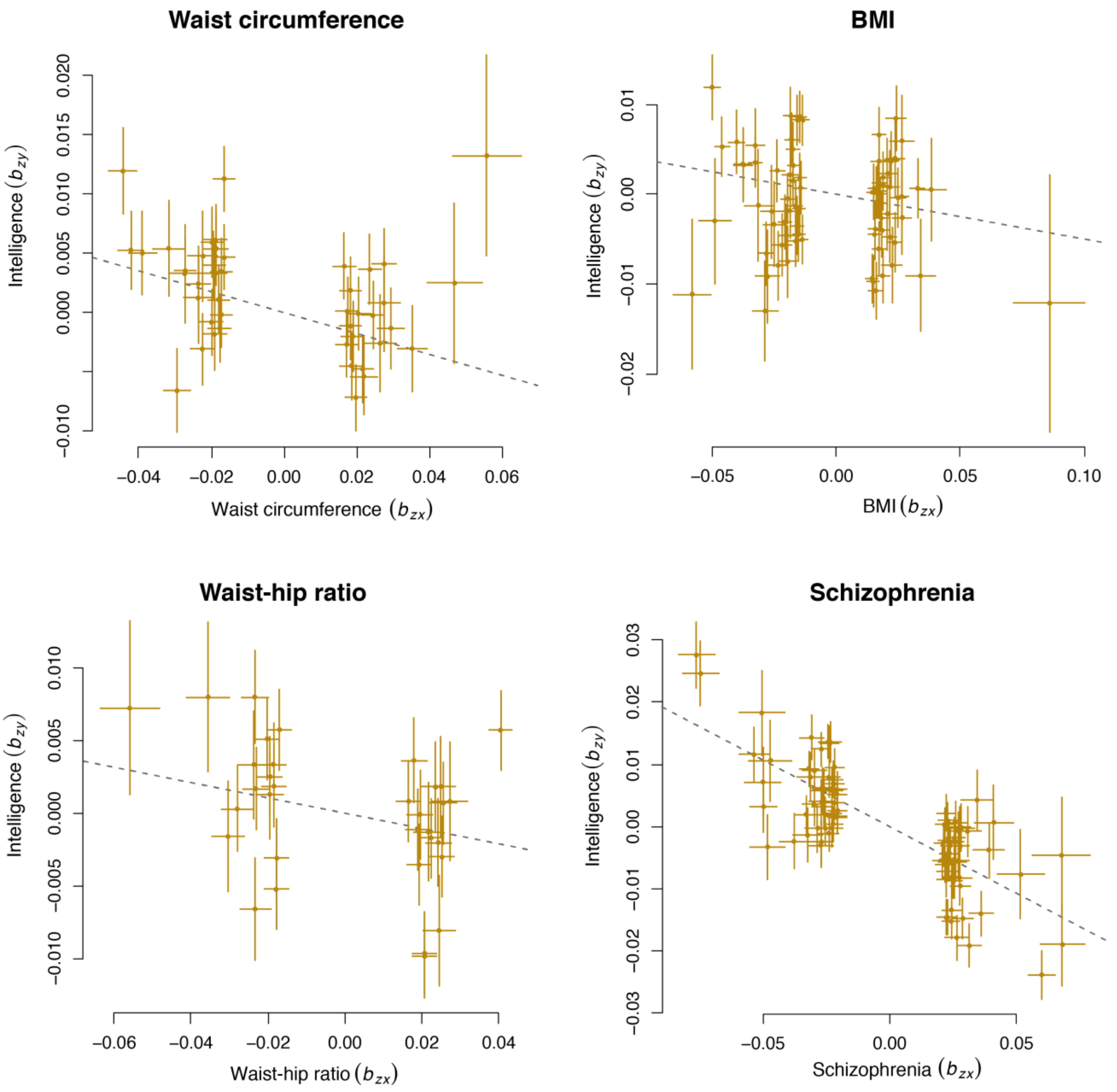
Waist circumference
Intelligence (b_{zy})
0.020
0.015
0.010
0.005
0.000
−0.010
−0.04
−0.02
0.00
0.02
0.04
0.06
Waist circumference (b_{zx})
BMI
Intelligence (b_{zy})
0.01
0.00
−0.01
−0.02
−0.05
0.00
0.05
0.10
BMI (b_{zx})
Waist-hip ratio
Intelligence (b_{zy})
0.010
0.005
0.000
−0.005
−0.010
−0.06
−0.04
−0.02
0.00
0.02
0.04
Waist-hip ratio (b_{zx})
Schizophrenia
Intelligence (b_{zy})
0.03
0.02
0.01
0.00
−0.01
−0.02
−0.03
−0.05
0.00
0.05
Schizophrenia (b_{zx})

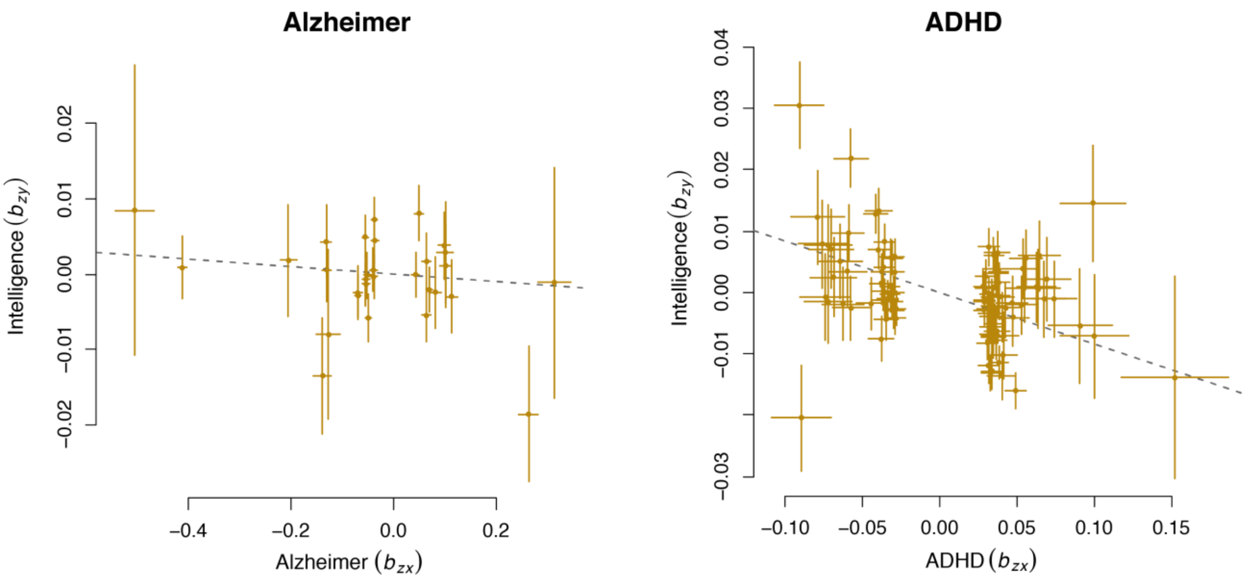

Supplementary Figure 12. Mendelian Randomization reverse tests for the effect of other correlated traits on intelligence. Plots of effect sizes of independent lead SNPs from a GWAS meta-analysis of intelligence in 269,867 independent individuals (b_{zy}) on the y-axis and SNP GWAS effect sizes for correlated traits on the x-axis (b_{zx}). The dotted line represents a line with slope of (b_{xy}) and an intercept of 0. Error bars show 95% confidence intervals for the effect sizes for each trait.

4. List of Supplementary Tables (separate Excel file)

Supplementary Table 1. Overview of the 14 cohorts included in a GWAS meta-analysis of intelligence in 269,867 independent individuals.

Supplementary Table 2. Genetic correlations between cohorts in a GWAS meta-analysis of intelligence in 269,867 independent individuals.

Supplementary Table 3. Heritability and genetic correlations across age subgroups of cohorts in a GWAS meta-analysis of intelligence in 269,867 independent individuals.

Supplementary Table 4. Test statistic inflation and heritability estimates in GWAS and meta-analysis of intelligence in 269,867 independent individuals from 14 cohorts.

Supplementary Table 5. Summary statistics for 242 independent lead SNPs in distinct genomic loci associated with intelligence in a meta-analysis of 269,867 independent individuals.

Supplementary Table 6. Summary statistics and functional annotation for SNPs reaching genome-wide significance in a meta-analysis of intelligence in 269,867 independent individuals.

Supplementary Table 7. Additional information on eight low confidence genome-wide significant loci which showed suspicious patterns of regional linkage disequilibrium (LD) in a GWAS meta-analysis of 269,867 independent individuals.

Supplementary Table 8. Proxy replication for SNPs associated with intelligence in a GWAS of educational attainment (EA) in 188,435 independent individuals.

Supplementary Table 9. Polygenic scores derived from GWAS meta-analysis results predicting intelligence in independent samples.

Supplementary Table 10. Heritability of intelligence in a GWAS meta-analysis of 269,867 independent individuals, stratified by genomic annotation categories.

Supplementary Table 11. Exonic non-synonymous (ExNS) variants in the genomic loci associated with intelligence in a GWAS meta-analysis of 269,867 independent individuals.

Supplementary Table 12. Genes implicated by positional, eQTL, or chromatin interaction mapping of SNPs associated with intelligence in a GWAS meta-analysis of 269,867 independent individuals.

Supplementary Table 13. Chromatin interaction regions linking significant GWAS loci from a meta-analysis of intelligence in 269,867 individuals to implicated genes.

Supplementary Table 14. Gene-sets with significant enrichment for genes associated with intelligence based on positional, eQTL, or chromatin interaction of significant GWAS SNPs.

Supplementary Table 15. Genes significantly associated with intelligence in gene-based association tests (GWGAS) for 269,867 independent individuals.

Supplementary Table 16. Details of 105 genes implicated by four strategies: positional, eQTL, and chromatin interaction mapping of significant GWAS SNPs, and gene-based association testing.

Supplementary Table 17. Gene-set association results for curated gene-sets in a GWAS meta-analysis of 269,867 independent individuals.

Supplementary Table 18. Supporting evidence for individual genes in gene-sets with significant associations with intelligence in 279,867 independent individuals.

Supplementary Table 19. Gene-set association results for tissue-specific gene expression in 269,867 independent individuals.

Supplementary Table 20. Gene-set association results for single cell-specific gene expression in brain cells in 269,867 independent individuals.

Supplementary Table 21. Genetic correlations (rg) between intelligence and 38 traits based on previously published GWAS results.

Supplementary Table 22. Catalogue of previously reported GWAS associations from the NCBI database for SNPs identified in a meta-analysis of intelligence in 269,867 independent individuals.

Supplementary Table 23. Tests for overrepresentation of genes mapped by significant GWAS SNPs in sets of genes with previously identified associations with human traits and diseases.

Supplementary Table 24. Overlap of genome-wide significant SNPs identified by the present study and previous GWAS of intelligence.

Supplementary Table 25. Genes implicated in intelligence using various association methods in the present and previous studies.

Supplementary Table 26. Results of Mendelian randomization tests for traits genetically correlated with intelligence.

5. Additional Acknowledgements

Acknowledgements for each of the study cohorts follow below:

UKB: This research has been conducted using the UK Biobank Resource (application 16406). We thank the participants and researchers who collected and contributed to the data.

COGENT: This work has been supported by grants from the National Institutes of Health (R01MH079800 and P50MH080173 to AKM; R01MH080912 to DCG; K23MH077807 to KEB; K01MH085812 to MCK). Data collection for the TOP cohort was supported by the Research Council of Norway, South-East Norway Health Authority, and KG Jebsen Foundation. The NCNG study was supported by Research Council of Norway Grants 154313/V50 and 177458/V50. The NCNG GWAS was financed by grants from the Bergen Research Foundation, the University of Bergen, the Research Council of Norway (FUGE, Psykisk Helse), Helse Vest RHF and Dr Einar Martens Fund. The Helsinki Birth Cohort Study has been supported by grants from the Academy of Finland, the Finnish Diabetes Research Society, Folkhälsan Research Foundation, Novo Nordisk Foundation, Finska Läkaresällskapet, Signe and Ane Gyllenberg Foundation, University of Helsinki, Ministry of Education, Ahokas Foundation, Emil Aaltonen Foundation. For the LBC1936 cohort, phenotype collection was supported by The Disconnected Mind project. Genotyping was funded by the UK Biotechnology and Biological Sciences Research Council (BBSRC grant No. BB/F019394/1). The work was undertaken by The University of Edinburgh Centre for Cognitive Ageing and Cognitive Epidemiology, part of the cross council Lifelong Health and Wellbeing Initiative, which is funded by the Medical Research Council and the Biotechnology and Biological Sciences Research Council (MR/K026992/1). The CAMH work was supported by the CAMH Foundation and the Canadian Institutes of Health Research. The Duke

Cognition Cohort (DCC) acknowledges K. Linney, J.M. McEvoy, P. Hunt, V. Dixon, T. Pennuto, K. Cornett, D. Swilling, L. Phillips, M. Silver, J. Covington, N. Walley, J. Dawson, H. Onabanjo, P. Nicoletti, A. Wagoner, J. Elmore, L. Bevan, J. Hunkin and R. Wilson for recruitment and testing of subjects. DCC also acknowledges the Ellison Medical Foundation New Scholar award AG-NS-0441-08 for partial funding of this study as well as the National Institute of Mental Health of the National Institutes of Health under award number K01MH098126. The UCLA Consortium for Neuropsychiatric Phenomics (CNP) study acknowledges the following sources of funding from the NIH: Grants UL1DE019580 and PL1MH083271 (RMB), RL1MH083269 (TDC), RL1DA024853 (EL) and PL1NS062410. The ASPIS study was supported by National Institute of Mental Health research grants R01MH085018 and R01MH092515 to Dr. Dimitrios Avramopoulos. Support for the Duke Neurogenetics Study was provided by the National Institutes of Health (R01 DA033369 and R01 AG049789 to A.R.Har) and by a National Science Foundation Graduate Research Fellowship to MAS. Recruitment, genotyping and analysis of the TCD healthy control samples were supported by Science Foundation Ireland (grants 12/IP/1670, 12/IP/1359 and 08/IN.1/B1916). Genotyping of the Manchester Cognitive Ageing Cohort was supported by the UK's Biotechnology and Biological Sciences Research Council (BBSRC; grant id: F01256X/1).

Data access for several cohorts used in this study was provided by the National Center for Biotechnology Information (NCBI) database of Genotypes and Phenotypes (dbGaP). dbGaP accession numbers for these cohorts were:

Cardiovascular Health Study (CHS): phs000287.v4.p1, phs000377.v5.p1, and phs000226.v3.p1

Framingham Heart Study (FHS): phs000007.v23.p8 and phs000342.v11.p8

Multi-Site Collaborative Study for Genotype-Phenotype Associations in Alzheimer's Disease (GENADA): phs000219.v1.p1

Long Life Family Study (LLFS): phs000397.v1.p1

Genetics of Late Onset Alzheimer's Disease Study (LOAD): phs000168.v1.p1

Minnesota Center for Twin and Family Research (MCTFR): phs000620.v1.p1

Philadelphia Neurodevelopmental Cohort (PNC): phs000607.v1.p1

The acknowledgment statements for these cohorts are found below:

Framingham Heart Study: The Framingham Heart Study is conducted and supported by the National Heart, Lung, and Blood Institute (NHLBI) in collaboration with Boston University (Contract No. N01-HC-25195 and HHSN268201500001I). This manuscript was not prepared in collaboration with investigators of the Framingham Heart Study and does not necessarily reflect the opinions or views of the Framingham Heart Study, Boston University, or NHLBI. Funding for SHARe Affymetrix genotyping was provided by NHLBI Contract N02-HL-64278. SHARe Illumina genotyping was provided under an agreement between Illumina and Boston University.

Cardiovascular Health Study: This research was supported by contracts HHSN268201200036C, HHSN268200800007C, N01HC85079, N01HC85080, N01HC85081, N01HC85082, N01HC85083, N01HC85084, N01HC85085, N01HC85086, N01HC35129, N01HC15103, N01HC55222, N01HC75150, N01HC45133, and N01HC85239; grant numbers U01HL080295 and U01HL130014 from the National Heart, Lung, and Blood Institute, and R01AG023629 from the National Institute on Aging, with additional contribution from the National Institute of Neurological Disorders and Stroke. A full list of principal CHS investigators and institutions can be found at https://chs-nhlbi.org/pi. This manuscript was not prepared in collaboration with CHS

investigators and does not necessarily reflect the opinions or views of CHS, or the NHLBI. Support for the genotyping through the CARe Study was provided by NHLBI Contract N01HC65226. Support for the Cardiovascular Health Study Whole Genome Study was provided by NHLBI grant HL087652. Additional support for infrastructure was provided by HL105756 and additional genotyping among the African-American cohort was supported in part by HL085251, DNA handling and genotyping at Cedars-Sinai Medical Center was supported in part by National Center for Research Resources grant UL1RR033176, now at the National Center for Advancing Translational Technologies CTSI grant UL1TR000124; in addition to the National Institute of Diabetes and Digestive and Kidney Diseases grant DK063491 to the Southern California Diabetes Endocrinology Research Center.

Multi-Site Collaborative Study for Genotype-Phenotype Associations in Alzheimer's Disease: The genotypic and associated phenotypic data used in the study were provided by the GlaxoSmithKline, R&D Limited. Details on data acquisition have been published previously in: Li H, Wetten S, Li L, St Jean PL, Upmanyu R, Surh L, Hosford D, Barnes MR, Briley JD, Borrie M, Coletta N, Delisle R, Dhalla D, Ehm MG, Feldman HH, Fornazzari L, Gauthier S, Goodgame N, Guzman D, Hammond S, Hollingworth P, Hsiung GY, Johnson J, Kelly DD, Keren R, Kertesz A, King KS, Lovestone S, Loy-English I, Matthews PM, Owen MJ, Plumpton M, Pryse-Phillips W, Prinjha RK, Richardson JC, Saunders A, Slater AJ, St George-Hyslop PH, Stinnett SW, Swartz JE, Taylor RL, Wherrett J, Williams J, Yarnall DP, Gibson RA, Irizarry MC, Middleton LT, Roses AD. Candidate single-nucleotide polymorphisms from a genomewide association study of Alzheimer disease. Arch Neurol., Jan;65(1):45-53, 2008 (PMID: 17998437).

Filippini N, Rao A, Wetten S, Gibson RA, Borrie M, Guzman D, Kertesz A, Loy-English I,

Williams J, Nichols T, Whitcher B, Matthews PM. Anatomically-distinct genetic associations of APOE epsilon4 allele load with regional cortical atrophy in Alzheimer's disease. Neuroimage, Feb 1;44(3):724-8, 2009. (PMID: 19013250).

Genetics of Late Onset Alzheimer's Disease Study: Funding support for the "Genetic Consortium for Late Onset Alzheimer's Disease" was provided through the Division of Neuroscience, NIA. The Genetic Consortium for Late Onset Alzheimer's Disease includes a genome-wide association study funded as part of the Division of Neuroscience, NIA. Assistance with phenotype harmonization and genotype cleaning, as well as with general study coordination, was provided by Genetic Consortium for Late Onset Alzheimer's Disease. A list of contributing investigators is available at https://www.ncbi.nlm.nih.gov/projects/gap/cgi-bin/study.cgi?study_id=phs000168.v1.p1

Long Life Family Study: Funding support for the Long Life Family Study was provided by the Division of Geriatrics and Clinical Gerontology, National Institute on Aging. The Long Life Family Study includes GWAS analyses for factors that contribute to long and healthy life. Assistance with phenotype harmonization and genotype cleaning as well as with general study coordination, was provided by the Division of Geriatrics and Clinical Gerontology, National Institute on Aging. Support for the collection of datasets and samples were provided by Multicenter Cooperative Agreement support by the Division of Geriatrics and Clinical Gerontology, National Institute on Aging (UO1AG023746; UO1AG023755; UO1AG023749; UO1AG023744; UO1AG023712). Funding support for the genotyping which was performed at the Johns Hopkins University Center for Inherited Disease Research was provided by the National Institute on Aging, National Institutes of Health.

Minnesota Center for Twin and Family Research: This project was led by William G. Iacono, PhD. And Matthew K. McGue, PhD (Co-Principal Investigators) at the University of Minnesota, Minneapolis, MN, USA. Co-investigators from the same institution included: Irene J. Elkins, Margaret A. Keyes, Lisa N. Legrand, Stephen M. Malone, William S. Oetting, Michael B. Miller, and Saonli Basu. Funding support for this project was provided through NIDA (U01DA024417). Other support for sample ascertainment and data collection came from several grants: R37DA05147, R01AA09367, R01AA11886, R01DA13240, R01MH66140.

Philadelphia Neurodevelopmental Cohort: Support for the collection of the data sets was provided by grant RC2MH089983 awarded to Raquel Gur, MD, and RC2MH089924 awarded to Hakon Hakonarson, MD, PhD. All subjects were recruited through the Center for Applied Genomics at The Children's Hospital in Philadelphia.

RS: The Rotterdam Study is supported by the Erasmus Medical Center and Erasmus University Rotterdam, the Netherlands Organization for Scientific Research (NWO), the Netherlands Organization for Health Research and Development (ZonMw), the Research Institute for Diseases in the Elderly (RIDE), the Ministry of Education, Culture and Science, the Ministry of Health, Welfare and Sports, the European Commission (DG XII), and the Municipality of Rotterdam. The contribution of inhabitants, general practitioners and pharmacists of the Ommoord district to the Rotterdam Study is greatly acknowledged.

GENR: The Generation R Study is conducted by the Erasmus Medical Center, Rotterdam in close collaboration with the Erasmus University Rotterdam, the Municipal Health Service Rotterdam area, the Rotterdam Homecare Foundation and the Stichting Trombosedienst & Artsenlaboratorium Rijnmond (STAR), Rotterdam. The authors wish to thank the parents and

children that participate in the Generation R Study. The Generation R Study is made possible by financial support from the Erasmus Medical Center, Rotterdam, the Erasmus University Rotterdam, the Simons Foundation Autism Research Initiative (SFARI - 307280), and the Netherlands Organization for Health Research and Development ZonMw grant number 10.000.1003 and ZonMw TOP grant number 91211021. HT was funded by a ZonMW VICI grant (016.VICI.170.200).

STR: The authors thank the STR cohort for making summary statistics available. The study was funded by the Jan Wallander and Tom Hedelius Foundation, the Ragnar Söderberg Foundation (E9/11), the Swedish Council for Working Life and Social Research, the Ministry for Higher Education, Karolinska Institutet, the Swedish Research Council (421-2013-1061; M-2005-1112), GenomEUtwin (EU/QLRT- 2001-01254; QLG2-CT-2002-01254), NIH DK U01-066134, The Swedish Foundation for Strategic Research (SSF).

S4S: Spit for Science: The VCU Student Survey has been supported by Virginia Commonwealth University, P20AA017828, R37AA011408, K02AA018755, and P50AA022537 from the National Institute on Alcohol Abuse and Alcoholism, and UL1RR031990 from the National Center for Research Resources and National Institutes of Health Roadmap for Medical Research. We would like to thank the VCU students for making this study a success, as well as the many VCU faculty, students, and staff who contributed to the design and implementation of the project.

HiQ/HRS: Analyses in this paper represent independent research funded by the National Institute for Health Research (NIHR) Biomedical Research Centre at South London and Maudsley NHS Foundation Trust and King's College London. The views expressed are those of the authors and not necessarily those of the NHS, the NIHR or the Department of Health.

Analyses were performed using high performance computing facilities funded with capital equipment grants from the GSTT Charity (TR130505) and Maudsley Charity (980). Research on the HiQ cohort was supported by a European Research Council Advanced Investigator award (295366) and an award from the John Templeton Foundation (13575) to Robert Plomin. The controls for this study were from The University of Michigan Health and Retirement Study, obtained through dbGaP, accession# phs000428.v2.p2, which is funded by the National Institute on Aging (grant numbers 01AG009740, RC2AG036495, and RC4AG039029) and conducted by the University of Michigan, Ann Arbor, MI.

TEDS: We gratefully acknowledge the ongoing contribution of the participants in the Twins Early Development Study (TEDS) and their families. TEDS is supported by a program grant to RP from the UK Medical Research Council (MR/M021475/1 and previously G0901245), with additional support from the US National Institutes of Health (AG046938). The research leading to these results has also received funding from the European Research Council under the European Union's Seventh Framework Programme (FP7/2007-2013)/ grant agreement no. 602768 and ERC grant agreement no. 295366. RP is supported by a Medical Research Council Professorship award (G19/2). EK is supported by the MRC/IoPPN Excellence Award.

DTR: The DTR is supported by grants from The National Program for Research Infrastructure 2007 from the Danish Agency for Science, Technology and Innovation and the US National Institutes of Health (P01 AG08761). The Danish Aging Research Center is supported by a grant from the VELUX Foundation. Genotyping was supported by NIH R01 AG037985 (Pedersen).

IMAGEN: The IMAGEN consortium received support from the following sources: the European Union-funded FP6 Integrated Project IMAGEN (Reinforcement-related behaviour in normal

brain function and psychopathology) (LSHM-CT- 2007-037286), the Horizon 2020 funded ERC Advanced Grant 'STRATIFY' (Brain network based stratification of reinforcement-related disorders) (695313), ERANID (Understanding the Interplay between Cultural, Biological and Subjective Factors in Drug Use Pathways) (PR-ST-0416-10004), BRIDGET (JPND: BRain Imaging, cognition Dementia and next generation GEnomics) (MR/N027558/1), the FP7 projects IMAGEMEND(602450; IMAging GEnetics for MENtal Disorders) and MATRICS (603016), the Innovative Medicine Initiative Project EU-AIMS (115300-2), the Medical Research Council Grant 'c-VEDA' (Consortium on Vulnerability to Externalizing Disorders and Addictions) (MR/N000390/1), the Swedish Research Council FORMAS, the Medical Research Council, the National Institute for Health Research (NIHR) Biomedical Research Centre at South London and Maudsley NHS Foundation Trust and King's College London, the Bundesministeriumfür Bildung und Forschung (BMBF grants 01GS08152; 01EV0711; eMED SysAlc01ZX1311A; Forschungsnetz AERIAL), the Deutsche Forschungsgemeinschaft (DFG grants SM 80/7-1, SM 80/7-2, SFB 940/1). Further support was provided by grants from: ANR (project AF12-NEUR0008-01 - WM2NA, and ANR-12-SAMA-0004), the Fondation de France, the Fondation pour la Recherche Médicale, the Mission Interministérielle de Lutte-contre-les-Drogues-et-les-Conduites-Addictives (MILDECA), the Assistance-Publique-Hôpitaux-de-Paris and INSERM (interface grant), Paris Sud University IDEX 2012; the National Institutes of Health, Science Foundation Ireland (16/ERCD/3797), U.S.A. (Axon, Testosterone and Mental Health during Adolescence; RO1 MH085772-01A1), and by NIH Consortium grant U54 EB020403, supported by a cross-NIH alliance that funds Big Data to Knowledge Centres of Excellence. Consortium contributors include Lisa Albrecht (Charité), Chris Andrew (IoP), Mercedes Arroyo (Cambridge University), Eric Artiges (INSERM), Semiha Aydin

(PTB), Christine Bach (Central Institute of Mental Health), Tobias Banaschewski (Central Institute of Mental Health), Alexis Barbot (Commissariat à l'Energie Atomique), Gareth Barker (IoP), Nathalie Boddaert (INSERM), Arun Bokde (Trinity College Dublin), Zuleima Bricaud (INSERM), Uli Bromberg (University of Hamburg), Ruediger Bruehl (PTB), Christian Büchel (University of Hamburg), Arnaud Cachia (INSERM), Anna Cattrell (IoP), Patricia Conrod (IoP), Patrick Constant (PERTIMM), Hans Crombag (University of Sussex), Katharina Czech (Charité), Jeffrey Dalley (Cambridge University), Benjamin Decideur (Commissariat à l'Energie Atomique), Sylvane Desrivieres (IoP), Tahmine Fadai (University of Hamburg), Herta Flor (Central Institute of Mental Health), Vincent Frouin (Commissariat à l'Energie Atomique), Birgit Fuchs (GABO:milliarium mbH & Co. KG), Jürgen Gallinat (Charité), Hugh Garavan (Trinity College Dublin), Fanny Gollier Briand (INSERM), Penny Gowland (University of Nottingham), Kay Head (University of Nottingham), Bert Heinrichs (Deutsches Referenzzentrum für Ethik), Andreas Heinz (Charité), Nadja Heym (University of Nottingham), Thomas Hübner (Technische Universität Dresden), Albrecht Ihlenfeld (PTB), James Ireland (Delosis), Bernd Ittermann (PTB), Nikolay Ivanov (Charité), Tianye Jia (IoP), Jennifer Jones (Trinity College Dublin), Arno Klaassen (Scito), Christophe Lalanne (Commissariat à l'Energie Atomique), Mark Lathrop (CNG), Dirk Lanzerath (Deutsches Referenzzentrum für Ethik), Hervé Lemaitre (INSERM), Katharina Lüdemann (Charité), Christine Macare (IoP), Catherine Mallik (IoP), Jean-François Mangin (INSERM), Karl Mann (Central Institute of Mental Health), Adam Mar (Cambridge University), Jean-Luc Martinot (INSERM), Jessica Massicotte (INSERM), Eva Mennigen (Technische Universität Dresden), Fabiana Mesquita de Carvahlo (IoP), Xavier Mignon (PERTIMM), Ruben Miranda (INSERM), Kathrin Müller (Technische Universität Dresden), Frauke Nees (Central

Institute of Mental Health), Charlotte Nymberg (IoP), Marie-Laure Paillere (INSERM), Tomas Paus (University of Toronto), Zdenka Pausova (University of Toronto), Yolanda Pena-Oliver (University of Sussex), Jean-Baptiste Poline (Commissariat à l'Energie Atomique), Luise Poustka (Central Institute of Mental Health), Michael Rapp (Charité), Laurence Reed (IoP), Gabriel Robert (IoP), Jan Reuter (Charité), Marcella Rietschel (Central Institute of Mental Health), Stephan Ripke (Technische Universität Dresden), Tamzin Ripley (University of Sussex), Trevor Robbins (Cambridge University), Sarah Rodehacke (Technische Universität Dresden), John Rogers (Delosis), Alexander Romanowski (Charité), Barbara Ruggeri (IoP), Christina Schilling (Charité), Christine Schmäl (Central Institute of Mental Health), Dirk Schmidt (Technische Universität Dresden), Sophia Schneider (University of Hamburg), Markus Schroeder (Tembit), Florian Schubert (PTB), Yannick Schwartz (Commissariat à l'Energie Atomique), Michael Smolka (Technische Universität Dresden), Wolfgang Sommer (Central Institute of Mental Health), Rainer Spanagel (Central Institute of Mental Health), Claudia Speiser (GABO:milliarium mbH & Co. KG), Tade Spranger (Deutsches Referenzzentrum für Ethik / Institut of Science and Ethics), Alicia Stedman (University of Nottingham), Sabina Steiner (Central Institute of Mental Health), Dai Stephens (University of Sussex), Nicole Strache (Charité), Andreas Ströhle (Charité), Maren Struve (Central Institute of Mental Health), Naresh Subramaniam (Cambridge University), David Theobald (Cambridge University), Lauren Topper (IoP), Sabine Vollstaedt-Klein (Central Institute of Mental Health), Bernadeta Walaszek (PTB), Henrik Walter (Charité), Katharina Weiß (Charité), Helen Werts (IoP), Robert Whelan (Trinity College Dublin), Steve Williams (IoP), Juliana Yacubian (University of Hamburg), Veronika Ziesch (Technische Universität Dresden),

Monica Zilbovicius (INSERM), C Peng Wong (IoP), Steven Lubbe (IoP), Lourdes Martinez-Medina (IoP), Agnes Kepa (IoP), Alinda Fernandes (IoP), Amir Tahmasebi (University of Toronto).

BLTS: These studies have been supported from multiple sources: National Health and Medical Research Council (389891, 552485, 1009064, 1031119, 1049894), Australian Research Council (A79600334, A79801419, A79906588, DP0212016, DP0664638, DP1093900), and Human Frontiers Science Program (RG0154/1998-B). SEM is supported by an Australian National Health and Medical Research Council fellowship (SRFB-1103623). We acknowledge the work and support of our collaborators and students, without which this work would not have been possible. We also are greatly appreciative of the assistance of our long-serving research assistants Natalie Garden, Marlene Grace, and Ann Eldridge; Project coordinators Kerrie McAloney, Alison MacKenzie and Romana Leisser; IT support from Harry Beeby, Daniel Park, and David Smyth; DNA sample preparation from Anjali Henders and the Molecular Genetics Laboratory; as well as many other research assistants and support staff in the Genetic Epidemiology Unit at QIMR Berghofer. Thanks go to the Education Board and school principals and staff for help in contacting twins. Finally, we warmly thank the twins and their families for their continued support, generosity of time, and for their interest in this research. G.W.M. is supported by an NHMRC Fellowship (GNT1078399).

NESCOG: We thank all participating subjects. This research was part of Science Live, the innovative research program of science center NEMO that enables scientists to carry out peer-reviewed research using NEMO visitors as volunteers. Collaboration between P.F.S. and the CNCR was facilitated by the Royal Academy of Arts and Sciences of The Netherlands, Visiting Professor Program (ISK/5913/VPP). The Netherlands Organization for Scientific Research (NWO)

Division for the Social Sciences (MaGW) provided funding for this research through grant VENI-451-08-025 to S.vdS. and VIDI 016-065-318 to D.P.

GfG: The Genes for Good study acknowledges funding from the University of Michigan Genomics Initiative, DA037904, AA023974, and HG008983.

STSA: The SATSA study included the following support (PI: N.L. Pedersen): NIH grants R01 AG04563, R01 AG10175, the MacArthur Foundation Research Network on Successful Aging, the Swedish Council For Working Life and Social Research (FAS) (97:0147:1B, 2009-0795) and Swedish Research Council (825-2007-7460, 825-2009-6141). Support for DNA extraction and genotyping was in part from NIH R01 AG028555 (Reynolds) and NIH R01 AG037985 (Pedersen).

The GENDER study included the following support: MacArthur Foundation Research Network on Successful Aging, The Axel and Margaret Ax:son Johnson's Foundation, The Swedish Council for Social Research, and the Swedish Foundation for Health Care Sciences and Allergy Research [PI: B. Malberg; A. Dahl Aslan]. Support for DNA extraction and genotyping was in part from NIH R01 AG028555 (Reynolds) and NIH R01 AG037985 (Pedersen).

The HARMONY study was supported by NIH grant R01 AG08724, (PI's: M. Gatz, N.L. Pedersen). Support for DNA extraction and genotyping was in part from NIH R01 AG028555 (Reynolds) and NIH R01 AG037985 (Pedersen).

6. References

1 Sudlow, C. *et al.* UK biobank: an open access resource for identifying the causes of a wide range of complex diseases of middle and old age. *PLoS Med* **12**, e1001779, doi:10.1371/journal.pmed.1001779 (2015).

2 Bycroft, C. *et al.* Genome-wide genetic data on ~500,000 UK Biobank participants. *bioRxiv*, doi:10.1101/166298 (2017).

3 McCarthy, S. *et al.* A reference panel of 64,976 haplotypes for genotype imputation. *Nat Genet* **48**, 1279-1283, doi:10.1038/ng.3643 (2016).

4 1000 Genomes Project Consortium *et al.* A global reference for human genetic variation. *Nature* **526**, 68-74, doi:10.1038/nature15393 (2015).

5 Webb, B. T. *et al.* Molecular Genetic Influences on Normative and Problematic Alcohol Use in a Population-Based Sample of College Students. *Frontiers in genetics* **8**, 30, doi:10.3389/fgene.2017.00030 (2017).

6 Abraham, G., Qiu, Y. & Inouye, M. FlashPCA2: principal component analysis of biobank-scale genotype datasets. *Bioinformatics*, doi:10.1093/bioinformatics/btx299 (2017).

7 Chang, C. C. *et al.* Second-generation PLINK: rising to the challenge of larger and richer datasets. *Gigascience* **4**, 7, doi:10.1186/s13742-015-0047-8 (2015).

8 Messer, L. C. *et al.* The development of a standardized neighborhood deprivation index. *Journal of urban health : bulletin of the New York Academy of Medicine* **83**, 1041-1062, doi:10.1007/s11524-006-9094-x (2006).

9 Trampush, J. W. *et al.* GWAS meta-analysis reveals novel loci and genetic correlates for general cognitive function: a report from the COGENT consortium. *Mol Psychiatry* **22**, 336-345, doi:10.1038/mp.2016.244 (2017).

10 Loh, P. R. *et al.* Efficient Bayesian mixed-model analysis increases association power in large cohorts. *Nat Genet* **47**, 284-290, doi:10.1038/ng.3190 (2015).

11 Willer, C. J., Li, Y. & Abecasis, G. R. METAL: fast and efficient meta-analysis of genomewide association scans. *Bioinformatics* **26**, 2190-2191, doi:10.1093/bioinformatics/btq340 (2010).

12 Hofman, A. *et al.* The Rotterdam Study: 2016 objectives and design update. *European journal of epidemiology* **30**, 661-708, doi:10.1007/s10654-015-0082-x (2015).

13 Kooijman, M. N. *et al.* The Generation R Study: design and cohort update 2017. *European journal of epidemiology* **31**, 1243-1264, doi:10.1007/s10654-016-0224-9 (2016).

14 Tellegen P, Winkel M, Wijnberg-Williams B & J., L. *Snijders-Oomen Niet-Verbale Intelligentietest: SON-R 2½-7.*, (Boom Testuitgevers, 2005).

15 Medina-Gomez, C. *et al.* Challenges in conducting genome-wide association studies in highly admixed multi-ethnic populations: the Generation R Study. *European journal of epidemiology* **30**, 317-330, doi:10.1007/s10654-015-9998-4 (2015).

16 Lichtenstein, P. *et al.* The Swedish Twin Registry: a unique resource for clinical, epidemiological and genetic studies. *Journal of internal medicine* **252**, 184-205 (2002).

17 Rietveld, C. A. *et al.* Common genetic variants associated with cognitive performance identified using the proxy-phenotype method. *Proc Natl Acad Sci U S A* **111**, 13790-13794, doi:10.1073/pnas.1404623111 (2014).
18 Howie, B. N., Donnelly, P. & Marchini, J. A flexible and accurate genotype imputation method for the next generation of genome-wide association studies. *PLoS Genet* **5**, e1000529, doi:10.1371/journal.pgen.1000529 (2009).
19 Abecasis, G. R., Cherny, S. S., Cookson, W. O. & Cardon, L. R. Merlin--rapid analysis of dense genetic maps using sparse gene flow trees. *Nat Genet* **30**, 97-101, doi:10.1038/ng786 (2002).
20 Sniekers, S. *et al.* Genome-wide association meta-analysis of 78,308 individuals identifies new loci and genes influencing human intelligence. *Nat Genet* **49**, 1107-1112, doi:10.1038/ng.3869 (2017).
21 Dick, D. M. *et al.* Spit for Science: launching a longitudinal study of genetic and environmental influences on substance use and emotional health at a large US university. *Front Genet* **5**, 47, doi:10.3389/fgene.2014.00047 (2014).
22 Frey, M. C. & Detterman, D. K. Scholastic Assessment or g? The relationship between the Scholastic Assessment Test and general cognitive ability. *Psychol Sci* **15**, 373-378, doi:10.1111/j.0956-7976.2004.00687.x (2004).
23 Marchini, J., Howie, B., Myers, S., McVean, G. & Donnelly, P. A new multipoint method for genome-wide association studies by imputation of genotypes. *Nat Genet* **39**, 906-913, doi:10.1038/ng2088 (2007).
24 Zabaneh, D. *et al.* A genome-wide association study for extremely high intelligence. *Mol Psychiatry*, doi:10.1038/mp.2017.121 (2017).
25 Trouton, A., Spinath, F. M. & Plomin, R. Twins early development study (TEDS): a multivariate, longitudinal genetic investigation of language, cognition and behavior problems in childhood. *Twin Res* **5**, 444-448, doi:10.1375/136905202320906255 (2002).
26 Wechsler, D. *Wechsler intelligence scale for children*. (The Psychological Corporation, 1949).
27 Raven J, Court J & J, R. *Manual for Raven's progressive matrices and vocabulary scales*. (Oxford University Press, 1996).
28 Raven, J. C. *Guide to using the Mill Hill vocabulary scale with the progressive matrices scale*. (H.K. Lewis, 1965).
29 Haworth, C. M. *et al.* Internet cognitive testing of large samples needed in genetic research. *Twin Res Hum Genet* **10**, 554-563, doi:10.1375/twin.10.4.554 (2007).
30 Gaist, D. *et al.* Strength and anthropometric measures in identical and fraternal twins: no evidence of masculinization of females with male co-twins. *Epidemiology* **11**, 340-343 (2000).
31 Skytthe, A. *et al.* The Danish Twin Registry: linking surveys, national registers, and biological information. *Twin Res Hum Genet* **16**, 104-111, doi:10.1017/thg.2012.77 (2013).
32 Skytthe, A., Kyvik, K., Holm, N. V., Vaupel, J. W. & Christensen, K. The Danish Twin Registry: 127 birth cohorts of twins. *Twin Res* **5**, 352-357, doi:10.1375/136905202320906084 (2002).

33 McGue, M. & Christensen, K. The heritability of cognitive functioning in very old adults: evidence from Danish twins aged 75 years and older. *Psychology and aging* **16**, 272-280 (2001).
34 Schumann, G. *et al.* The IMAGEN study: reinforcement-related behaviour in normal brain function and psychopathology. *Mol Psychiatry* **15**, 1128-1139, doi:10.1038/mp.2010.4 (2010).
35 Robbins, T. W. *et al.* Cambridge Neuropsychological Test Automated Battery (CANTAB): a factor analytic study of a large sample of normal elderly volunteers. *Dementia (Basel, Switzerland)* **5**, 266-281 (1994).
36 Delaneau, O., Zagury, J. F. & Marchini, J. Improved whole-chromosome phasing for disease and population genetic studies. *Nat Methods* **10**, 5-6, doi:10.1038/nmeth.2307 (2013).
37 Gillespie, N. A. *et al.* The Brisbane Longitudinal Twin Study: Pathways to Cannabis Use, Abuse, and Dependence project-current status, preliminary results, and future directions. *Twin Res Hum Genet* **16**, 21-33, doi:10.1017/thg.2012.111 (2013).
38 Jackson, D. *Multidimensional aptitude battery II*. (Sigma Assessment Systems, 1998).
39 Mellanby J & Langdon, D. *Verbal and spatial reasoning test for children*. (Cambridge Assessment, 2010).
40 Liu, D. J. *et al.* Meta-analysis of gene-level tests for rare variant association. *Nat Genet* **46**, 200-204, doi:10.1038/ng.2852 (2014).
41 Polderman, T. J. *et al.* Attentional switching forms a genetic link between attention problems and autistic traits in adults. *Psychol Med* **43**, 1985-1996, doi:10.1017/s0033291712002863 (2013).
42 Wechsler, D. & De Lemos, M. M. *Wechsler adult intelligence scale - revised*. (1981).
43 Condon, D. M. & Revelle, W. The international cognitive ability resource: Development and initial validation of a public-domain measure. *Intelligence* **43**, 52-64, doi:http://dx.doi.org/10.1016/j.intell.2014.01.004 (2014).
44 Finkel, D. & Pedersen, N. L. Processing Speed and Longitudinal Trajectories of Change for Cognitive Abilities: The Swedish Adoption/Twin Study of Aging. *Aging, Neuropsychology, and Cognition* **11**, 325-345, doi:10.1080/13825580490511152 (2004).
45 Gold, C. H., Malmberg, B., McClearn, G. E., Pedersen, N. L. & Berg, S. Gender and health: a study of older unlike-sex twins. *J Gerontol B Psychol Sci Soc Sci* **57**, S168-176 (2002).
46 Purcell, S. *et al.* PLINK: a tool set for whole-genome association and population-based linkage analyses. *Am J Hum Genet* **81**, 559-575, doi:10.1086/519795 (2007).
47 Gatz, M. & Pedersen, N. L. Study of Dementia in Swedish Twins. *Twin Res Hum Genet* **16**, 313-316, doi:10.1017/thg.2012.68 (2013).
48 Spearman, C. "General Intelligence," Objectively Determined and Measured. *The American Journal of Psychology* **15**, 201-292, doi:10.2307/1412107 (1904).
49 Deary, I. J., Penke, L. & Johnson, W. The neuroscience of human intelligence differences. *Nat Rev Neurosci* **11**, 201-211, doi:10.1038/nrn2793 (2010).
50 Johnson, W., Bouchard, T. J., Krueger, R. F., McGue, M. & Gottesman, I. I. Just one g: consistent results from three test batteries. *Intelligence* **32**, 95-107, doi:https://doi.org/10.1016/S0160-2896(03)00062-X (2004).

51 Panizzon, M. S. *et al.* Genetic and Environmental Influences of General Cognitive Ability: Is g a valid latent construct? *Intelligence* **43**, 65-76, doi:10.1016/j.intell.2014.01.008 (2014).

52 Spain, S. L. *et al.* A genome-wide analysis of putative functional and exonic variation associated with extremely high intelligence. *Mol Psychiatry* **21**, 1145-1151, doi:10.1038/mp.2015.108 (2016).

53 Selzam, S. *et al.* Predicting educational achievement from DNA. *Mol Psychiatry* **22**, 267-272, doi:10.1038/mp.2016.107 (2017).

54 Plomin, R. & Kovas, Y. Generalist genes and learning disabilities. *Psychol Bull* **131**, 592-617, doi:10.1037/0033-2909.131.4.592 (2005).

55 Plomin, R. & von Stumm, S. The new genetics of intelligence. *Nat Rev Genet*, doi:10.1038/nrg.2017.104 (2018).

56 Bulik-Sullivan, B. K. *et al.* LD Score regression distinguishes confounding from polygenicity in genome-wide association studies. *Nat Genet* **47**, 291-295, doi:10.1038/ng.3211 (2015).

57 Bulik-Sullivan, B. *et al.* An atlas of genetic correlations across human diseases and traits. *Nat Genet* **47**, 1236-1241, doi:10.1038/ng.3406 (2015).

58 Davies, G. *et al.* Genome-wide association study of cognitive functions and educational attainment in UK Biobank (N=112 151). *Mol Psychiatry* **21**, 758-767, doi:10.1038/mp.2016.45 (2016).

59 Konig, I. R., Loley, C., Erdmann, J. & Ziegler, A. How to include chromosome X in your genome-wide association study. *Genet Epidemiol* **38**, 97-103, doi:10.1002/gepi.21782 (2014).

60 Deary, I. J., Strand, S., Smith, P. & Fernandes, C. Intelligence and educational achievement. *Intelligence* **35**, 13-21, doi:https://doi.org/10.1016/j.intell.2006.02.001 (2007).

61 Rietveld, C. A. *et al.* GWAS of 126,559 individuals identifies genetic variants associated with educational attainment. *Science* **340**, 1467-1471, doi:10.1126/science.1235488 (2013).

62 Okbay, A. *et al.* Genome-wide association study identifies 74 loci associated with educational attainment. *Nature* **533**, 539-542, doi:10.1038/nature17671 (2016).

63 Vilhjalmsson, B. J. *et al.* Modeling Linkage Disequilibrium Increases Accuracy of Polygenic Risk Scores. *Am J Hum Genet* **97**, 576-592, doi:10.1016/j.ajhg.2015.09.001 (2015).

64 Euesden, J., Lewis, C. M. & O'Reilly, P. F. PRSice: Polygenic Risk Score software. *Bioinformatics* **31**, 1466-1468, doi:10.1093/bioinformatics/btu848 (2015).

65 Karmodiya, K., Krebs, A. R., Oulad-Abdelghani, M., Kimura, H. & Tora, L. H3K9 and H3K14 acetylation co-occur at many gene regulatory elements, while H3K14ac marks a subset of inactive inducible promoters in mouse embryonic stem cells. *BMC Genomics* **13**, 424, doi:10.1186/1471-2164-13-424 (2012).

66 Mendizabal, I., Keller, T. E., Zeng, J. & Yi, S. V. Epigenetics and Evolution. *Integrative and Comparative Biology* **54**, 31-42, doi:10.1093/icb/icu040 (2014).

67 Roadmap Epigenomics Consortium *et al.* Integrative analysis of 111 reference human epigenomes. *Nature* **518**, 317-330, doi:10.1038/nature14248 (2015).

68 Wang, K., Li, M. & Hakonarson, H. ANNOVAR: functional annotation of genetic variants from high-throughput sequencing data. *Nucleic Acids Res* **38**, e164, doi:10.1093/nar/gkq603 (2010).

69 Kircher, M. *et al.* A general framework for estimating the relative pathogenicity of human genetic variants. *Nat Genet* **46**, 310-315, doi:10.1038/ng.2892 (2014).

70 Boyle, A. P. *et al.* Annotation of functional variation in personal genomes using RegulomeDB. *Genome Res* **22**, 1790-1797, doi:10.1101/gr.137323.112 (2012).

71 Ernst, J. & Kellis, M. ChromHMM: automating chromatin-state discovery and characterization. *Nat Methods* **9**, 215-216, doi:10.1038/nmeth.1906 (2012).

72 Fagerberg, L. *et al.* Analysis of the human tissue-specific expression by genome-wide integration of transcriptomics and antibody-based proteomics. *Molecular & Cellular Proteomics : MCP* **13**, 397-406, doi:10.1074/mcp.M113.035600 (2014).

73 Davies, G. *et al.* Genetic contributions to variation in general cognitive function: a meta-analysis of genome-wide association studies in the CHARGE consortium (N=53949). *Mol Psychiatry* **20**, 183-192, doi:10.1038/mp.2014.188 (2015).

74 Hou, L. *et al.* Genome-wide association study of 40,000 individuals identifies two novel loci associated with bipolar disorder. *Hum Mol Genet* **25**, 3383-3394, doi:10.1093/hmg/ddw181 (2016).

75 Watanabe, K., Taskesen, E., van Bochoven, A. & Posthuma, D. FUMA: Functional mapping and annotation of genetic associations. *bioRxiv*, doi:10.1101/110023 (2017).

76 Lek, M. *et al.* Analysis of protein-coding genetic variation in 60,706 humans. *Nature* **536**, 285-291, doi:10.1038/nature19057 (2016).

77 Schmitt, A. D. *et al.* A Compendium of Chromatin Contact Maps Reveals Spatially Active Regions in the Human Genome. *Cell reports* **17**, 2042-2059, doi:10.1016/j.celrep.2016.10.061 (2016).

78 Liberzon, A. *et al.* Molecular signatures database (MSigDB) 3.0. *Bioinformatics* **27**, 1739-1740, doi:10.1093/bioinformatics/btr260 (2011).

79 GTEx Consortium. Human genomics. The Genotype-Tissue Expression (GTEx) pilot analysis: multitissue gene regulation in humans. *Science* **348**, 648-660, doi:10.1126/science.1262110 (2015).

80 Crespi, B. J. Autism As a Disorder of High Intelligence. *Front Neurosci* **10**, 300, doi:10.3389/fnins.2016.00300 (2016).

81 Zhu, Z. *et al.* Causal associations between risk factors and common diseases inferred from GWAS summary data. *bioRxiv*, doi:10.1101/168674 (2017).

82 Strenze, T. Intelligence and socioeconomic success: A meta-analytic review of longitudinal research. *Intelligence* **35**, 401-426, doi:https://doi.org/10.1016/j.intell.2006.09.004 (2007).

83 Batty, G. D., Deary, I. J. & Gottfredson, L. S. Premorbid (early life) IQ and later mortality risk: systematic review. *Annals of epidemiology* **17**, 278-288, doi:10.1016/j.annepidem.2006.07.010 (2007).

84 Davies, G. *et al.* A genome-wide association study implicates the APOE locus in nonpathological cognitive ageing. *Mol Psychiatry* **19**, 76-87, doi:10.1038/mp.2012.159 (2014).

85 Lambert, J. C. *et al.* Meta-analysis of 74,046 individuals identifies 11 new susceptibility loci for Alzheimer's disease. *Nat Genet* **45**, 1452-1458, doi:10.1038/ng.2802 (2013).

86 Stanek, K. C., Iacono, W. G. & McGue, M. Returns to Education: What Do Twin Studies Control? *Twin Res Hum Genet* **14**, 509-515 (2011).

87 Richards, M. & Sacker, A. Is education causal? Yes. *Int J Epidemiol* **40**, 516-518, doi:10.1093/ije/dyq166 (2011).

88 Johnson, W., Deary, I. J. & Iacono, W. G. Genetic and environmental transactions underlying educational attainment. *Intelligence* **37**, 466-478, doi:https://doi.org/10.1016/j.intell.2009.05.006 (2009).

89 Calvin, C. M. *et al.* Multivariate genetic analyses of cognition and academic achievement from two population samples of 174,000 and 166,000 school children. *Behav Genet* **42**, 699-710, doi:10.1007/s10519-012-9549-7 (2012).

90 Krapohl, E. *et al.* The high heritability of educational achievement reflects many genetically influenced traits, not just intelligence. *Proc Natl Acad Sci U S A* **111**, 15273-15278, doi:10.1073/pnas.1408777111 (2014).

91 Hedman, A. M. *et al.* Is there change in intelligence quotient in chronically ill schizophrenia patients? A longitudinal study in twins discordant for schizophrenia. *Psychol Med* **42**, 2535-2541, doi:10.1017/S0033291712000694 (2012).

92 Le Hellard, S. *et al.* Identification of Gene Loci That Overlap Between Schizophrenia and Educational Attainment. *Schizophr Bull* **43**, 654-664 (2017).

93 Lencz, T. *et al.* Molecular Genetic Evidence for Genetic Overlap between General Cognitive Ability and Risk for Schizophrenia: A Report from the Cognitive Genomics Consortium (COGENT). *Mol Psychiatry* **19**, 168-174, doi:10.1038/mp.2013.166 (2014).

94 Hoe, M., Nakagami, E., Green, M. F. & Brekke, J. S. The causal relationships between neurocognition, social cognition and functional outcome over time in schizophrenia: a latent difference score approach. *Psychol Med* **42**, 2287-2299, doi:10.1017/S0033291712000578 (2012).

95 Kendler, K. S., Ohlsson, H., Sundquist, J. & Sundquist, K. IQ and Schizophrenia in a Swedish National Sample: Their Causal Relationship and the Interaction of IQ with Genetic Risk. *Am J Psychiatry* **172**, 259-265, doi:10.1176/appi.ajp.2014.14040516 (2015).
